# Supplementary material for: Cost-effectiveness of HPV-based cervical cancer screening in the public health system in Nicaragua
Source: BMJ Open. 2017 Jun 15;7(6):e015048. doi: 10.1136/bmjopen-2016-015048 (PMC5623348; doi:10.1136/bmjopen-2016-015048)
Supplement: Supplementary Appendix [file bmjopen-2016-015048supp001.pdf]

Supplemental Appendix:
The Cost-Effectiveness of HPV-Based Screening in the Public Health System in Nicaragua

MODEL CALIBRATION

Overview of the calibration process

Details of model development for the U.S. and Nicaragua models, including initial parameterization and calibration, have been published elsewhere [1, 2]. Baseline HPV incidence rates, as a function of genotype and age, were derived from a published prospective study of sexually active women aged 15-85 years in Bogota, Colombia [3]. Because HPV incidence is not necessarily similar in Colombia and Nicaragua due to differences in sexual behavior, we considered age-specific HPV incidence and natural immunity following initial infection as candidate parameters for calibration. Type-specific data on CIN2 and CIN3 regression and progression are limited [4-9], so these highly uncertain parameters were also candidates for calibration. Time-dependent rates of HPV clearance and progression by genotype were informed by primary longitudinal data from the control arm of the Costa Rica Vaccine Trial [10], assuming that rates of HPV clearance and progression to precancer are similar across populations and vary only by time since infection and HPV genotype.

To calibrate the model, we selected empirical epidemiologic data from Nicaragua, including age-specific prevalence of high-risk HPV and age-specific cancer incidence (see below for further detail). We then set plausible search ranges around baseline model input values for age- and type-specific HPV incidence; natural immunity following initial infection; and progression and regression of CIN. We performed repeated model simulations in the absence of any preventive intervention (i.e., natural history simulations). For each simulation, we randomly selected a single value for each of the uncertain parameters from the identified search range, creating a unique vector of parameter values (i.e., parameter "set"). From over 1,475,000 repeated samplings, we identified the parameter sets with the highest correspondence to the empirical calibration target data from Nicaragua by calculating and aggregating the log-likelihood of model-projected outcomes. We used the 50 parameter sets with the highest likelihood scores (i.e., best overall fit to the empirical data from Nicaragua) for analysis to capture uncertainty in the model parameters as a form of probabilistic sensitivity analysis. We report results as a mean of outcomes across these top 50 parameter sets; incremental cost-effectiveness ratios are reported as the ratio of the mean costs divided by the mean effects of one strategy versus another across sets.

Calibration targets

We assessed model fit by comparing projected model outcomes of age-specific prevalence of high-risk HPV and age-specific cancer incidence relative to empirical data from Nicaragua. The scoring algorithm included age-specific prevalence of high-risk HPV and age-specific cervical cancer incidence.

Age-specific prevalence of high-risk HPV was drawn from the Screening Technologies to Advance Rapid Testing for Cervical Cancer Prevention–Utility and Program Planning (START-UP) project data on *care*HPV positivity using a cut-off ratio cut-point of 0.5 relative light units (**Table A.1**). For each age group, we derived a 95% binomial confidence interval around the point prevalence, which comprised the calibration target. The likelihood function for each age group was assumed to follow a binomial distribution.

Age-specific cancer incidence was drawn from Globocan due to the lack of a local cancer registry [11] (**Table A.2**). The likelihood function for each age group was assumed to follow a normal distribution.

Composite goodness-of-fit scores for each input parameter set were generated by summing the log likelihood of each model outcome (i.e., age-specific HPV prevalence, age-specific cancer incidence). The 50 input parameter sets with the highest goodness-of-fit scores yielded the model outputs that were simultaneously closest to all calibration targets, and these were selected for analysis. **Figures A.1** and **A.2** display model fit to epidemiologic data on age-specific prevalence of high-risk HPV and age-specific cancer incidence.

Transition probabilities for the calibrated natural history model are presented in **Table A.3.**

COST DATA

Direct Medical Costs: Screening, Diagnosis, and Treatment of Precancerous Lesions
The direct medical costs of screening, diagnosis, and treatment of precancerous lesions were drawn from the START-UP demonstration study in Masaya department and from the Scale-Up Nicaragua Project in the departments of Carazo, Chontales, and Chinandega. Direct medical costs included clinical staff time, clinical supplies, drugs, clinical equipment, laboratory staff time, laboratory supplies, and laboratory equipment. Costs from the START-UP study were collected in 2010 local currency units and inflated to year 2015 levels using Nicaragua GDP deflators and then converted to US$ using the official exchange rate [12]; the exception was for equipment, which was generally procured in the United States and was inflated to year 2015 levels using US GDP deflators. Costs from the Scale-Up Nicaragua project (including equipment) were collected in 2015 local currency units and converted to US$ using the official exchange rate [12].

Costs are reported in **Table A.4** (screening procedures) and **Table A.5** (precancer diagnostic and treatment procedures).

Women's Time and Transportation Costs
To account for the opportunity cost of women's time spent traveling to, waiting for, or receiving care, we valued women's time using Nicaragua's 2013 monthly minimum wage [13], converted to 2015 US$. While the minimum wage may be a conservative estimate for valuation of women's time if they are formally employed, it can serve as a proxy for the societal value of women's time spent working in the informal sector or at home. We converted this to an hourly wage rate assuming 171 hours of work per month (40 hours per week).

Estimates for time spent traveling to, waiting for, and receiving care were dependent upon the facility level where care was assumed to take place (**Table A.6**). While screening and delivery of primary screening results were assumed to take place at basic primary health facilities, we assumed cryotherapy and LEEP treatment were only provided at referral facilities. Estimates of women's time spent receiving a procedure were based on site-specific data from the START-UP demonstration projects, with staff time spent on the procedure (excluding preparation and registration time, which we assumed were built into patient waiting time) used as a proxy for women's procedure time. Estimates of women's wait time for screening and delivery of primary screening results was based on the average wait time for screening at facilities in the Chinandega department; wait time for cryotherapy, colposcopy, and LEEP was based on the average wait time at referral centers in the Chontales department. Round-trip transportation time was based on Scale-Up project staff estimates of the typical amount of time women spent traveling to screening or referral facilities, using a sample of 9 facilities the Carazo department and 10 facilities in the Chontales and Chinandega departments. We averaged across screening and referral facilities within

each department to obtain a department average, and then averaged across departments to obtain average round-trip transportation times based on facility level. Estimates of women's time and time costs are presented in **Table A.7.**

Women's transportation costs were based on Scale-Up project staff reports of the typical mode of transportation to a particular facility by women in the catchment area, using a sample of 9 facilities in Carazo and 10 facilities each in Chontales or Chinandega. We used the most commonly reported mode of transportation for each department (for screening facilities: walking for Carazo; walking for Chontales; bus for Chinandega; for referral facilities: bus in all three departments). The cost of a bus trip was based on typical bus fare for the distance to the health facility, as reported by Scale-Up project staff. We averaged the typical transportation costs across departments to obtain an estimate of women's round-trip transportation costs to and from a health facility, assuming the same mode of transport was used for the trip to and from the health facility. Women's transportation costs are presented in **Table A.8.**

Programmatic Costs

We included the programmatic cost of the following types of training sessions that were conducted during the Scale-Up project: 1) for health care personnel to offer careHPV screening and counsel women on results; 2) for laboratory technicians to run the careHPV assay; 3) for community outreach workers and auxiliary nurses to promote careHPV screening and offer self-collection of HPV samples; and 4) for health care providers to offer VIA and cryotherapy. We included the cost of trainer time and trainee time (valued using average monthly salaries converted to an hourly rate; volunteer outreach worker time was valued using the minimum wage), as well as the cost of supplies, materials, transportation, and refreshments. To derive an average health care personnel training cost per woman screened, we divided the total training cost for health care personnel by 100,000, corresponding to the number of women screened during the Scale-Up project. To derive an average laboratory technician training cost per woman screened, we divided the total training cost for laboratory technicians by 100,000. To derive an average community outreach worker training cost per woman screened, we divided the total training cost for outreach workers by 80,000, corresponding to the number of women who participated in self-collection of HPV samples. To derive an average health care provider training cost per woman receiving VIA and cryotherapy, we divided the total VIA/cryotherapy training cost for health care providers by 16,000, corresponding to the approximate number of women who tested HPV-positive in Scale-Up and would thus require visual assessment (to determine eligibility for cryotherapy) and, if eligible, cryotherapy.

All women screened with HPV testing incurred the per-woman training costs for health care personnel and laboratory technicians. Only women screened with HPV self-collection incurred the per-woman cost of training outreach workers to offer self-collection (due to lack of data on self-collection in a community setting, in the base case analysis we assumed self-collection took place at the clinic; however, we assumed outreach workers would be involved in mobilizing and educating women about self-collection, and conservatively included the cost of training so as to represent a known programmatic cost as self-collection efforts shift to also include community settings). Only women who received either VIA (as triage) or screen-and-treat cryotherapy incurred the per-woman cost of training health providers to perform VIA and cryotherapy. Training costs are presented in **Table A.9.**

Cost of Cancer Care by Stage

Costs associated with cancer care by stage (Local versus Regional or Distant), including direct medical costs, direct non-medical costs (i.e., transport to a cancer center, and meals and temporary housing

during the course of treatment), and patient and support person time were derived from a previous analysis in El Salvador [14]. Based on expert opinion in El Salvador, we assumed 50% of local cancer patients presented with FIGO stage 1a, requiring radical hysterectomy, and 50% of local cancer patients presented with FIGO stage 1b/2a, requiring cobalt therapy followed by 5 chemotherapy sessions followed by brachytherapy. We assumed all women presenting with FIGO stage 2b or higher received cobalt therapy followed by 5 chemotherapy sessions followed by brachytherapy.

To adjust the direct medical costs of cancer treatment in El Salvador to the setting of Nicaragua, we multiplied by the ratio of WHO-CHOICE inpatient bed-day costs at a teaching hospital in Nicaragua relative to El Salvador. We assumed each patient and a support person spent the same amount of time traveling, waiting for, and receiving care as in El Salvador, and valued this time at the 2013 minimum wage rate in Nicaragua (inflated and converted to 2015 US$). To adjust temporary housing and transportation costs to the setting of Nicaragua, we multiplied the Salvadoran costs by the ratio of GDP per capita in Nicaragua relative to El Salvador. Costs are reported in **Table A.10**.

SUPPLEMENTARY RESULTS

Additional results described in the main manuscript are presented in **Figures A3** to **A6.**

Table A.1. Age-specific prevalence of high-risk HPV, Nicaragua [15].[a]

Age group	Number of women	Number of women with high-risk HPV	Prevalence (95% CI)
30 – 34 years	1,693	310	0.18 (0.17, 0.20)
35 – 39 years	1,141	184	0.16 (0.14, 0.18)
40 – 44 years	933	125	0.13 (0.11, 0.16)
45 – 49 years	878	121	0.14 (0.12, 0.16)

[a] HPV positivity was based on a cut-off of 0.5 relative light units.

Table A.2. Age-specific cervical cancer incidence, Nicaragua (GLOBOCAN 2012) [11].

Age group	Cases	Rate per 100,000 women (95% CI)
40 – 44 years	123	78.7 (64.8, 92.6)
45 – 49 years	112	85.4 (69.6, 101.2)
50 – 54 years	102	88.4 (71.2, 105.6)
55 – 59 years	85	88.1 (69.4, 106.8)
60 – 64 years	51	84.0 (61.0, 107.1)
65 – 69 years	37	80.8 (54.8, 106.8)
70 – 74 years	30	74.6 (47.9, 101.3)
≥75 years	45	70.3 (49.8, 90.8)

Table A.3. Monthly transition probabilities in the calibrated natural history model of HPV infection and cervical carcinogenesis.

HPV Infection

HPV Type and Age Group, years	Baseline Value		Range of Multiplier Values Among Top 50 Parameter Sets[a]	
	Min	Max	Min	Max
**HPV 16**				
<21	0	0.00186	3.15278	7.90861
21-24	0.000903	0.00125	0.670187	6.75291
25-29	0.000782	0.00087	0.604518	7.39154
30-49	0.000602	0.00078	0.677505	7.86645
>= 50	0.000217	0.00059	0.363714	8.9736
**HPV 18**				
<21	0	0.00116	0.365305	9.94514
21-24	0.0011	0.001173	0.304444	8.18583
25-29	0.0006	0.001	0.305103	8.23571
30-49	0.000301	0.00058	0.336658	9.81842
>= 50	0.000109	0.000295	0.347223	9.86684
**HPV 31**				
<21	0	0.001428	0.432486	7.54208
21-24	0.0009	0.001364	0.332414	5.86815
25-29	0.0006	0.000805	0.312983	6.63163
30-49	0.000301	0.00055	0.381602	7.86096
>= 50	0.000109	0.000295	0.445801	7.91394
**HPV 33**				
<21	0	0.000786	0.337263	5.98918
21-24	0.000412	0.00069	0.321392	5.21972
25-29	0.00025	0.00036	0.321163	4.52998
30-49	0.000131	0.00022	0.315971	5.91849
>= 50	0.000054	0.000128	0.341468	5.73381
**HPV 45**				
<21	0	0.001	0.838185	7.90205
21-24	0.00041	0.0008	0.303553	6.97879
25-29	0.000315	0.00037	0.373551	7.22544
30-49	0.000161	0.000305	0.320699	7.65339
>= 50	0.000054	0.00015	0.313646	7.97911

**HPV 52**				
<21	0	0.001186	0.339479	5.92479
21-24	0.00027	0.0008	0.328541	4.11892
25-29	0.000184	0.000254	0.301478	4.73227
30-49	0.00015	0.000173	0.365365	5.61621
>= 50	0.000054	0.000148	0.333456	11.406
**HPV 58**				
<21	0	0.001189	0.586648	7.78762
21-24	0.00059	0.0011	0.30793	7.02402
25-29	0.000465	0.00055	0.376855	6.55248
30-49	0.000244	0.00045	0.300592	7.20446
>= 50	0.000054	0.000239	0.42182	7.99349
**Other Oncogenic Types**				
<21	0	0.00247	0.324069	5.82435
21-24	0.001804	0.0023	0.301534	5.30746
25-29	0.0012	0.0017	0.311474	4.99753
30-49	0.000602	0.0011	0.318774	5.86776
>= 50	0.000217	0.00059	0.313403	5.86636
**Nononcogenic Types**				
<21	0	0.00261	0.361451	14.721
21-24	0.00255	0.00262	0.327572	10.6641
25-29	0.002	0.0025	0.327932	13.399
30-49	0.00085	0.00186	0.326549	14.4844
>= 50	0.000217	0.0008	0.324229	14.05

HPV clearance

**HPV Type and time since infection, months**	Baseline Value	Multiplier Value Among Top 50 Parameter Sets[a]
**HPV 16**		
1-15	0.041886	1
16-27	0.040754	1
28-39	0.033905	1
40-51	0.031888	1
52+	0.019846	1
**HPV 18**		
1-15	0.073342	1
16-27	0.063235	1
28-39	0.053605	1
40-51	0.020616	1

52+	0.020616	1
**HPV 31**		
1-15	0.063447	1
16-27	0.033826	1
28-39	0.033826	1
40-51	0.033826	1
52+	0.033826	1
**HPV 33**		
1-15	0.083452	1
16-27	0.044955	1
28-39	0.036156	1
40-51	0.036156	1
52+	0.036156	1
**HPV 45**		
1-15	0.078517	1
16-27	0.042579	1
28-39	0.041675	1
40-51	0.030133	1
52+	0.030133	1
**HPV 52**		
1-15	0.062999	1
16-27	0.044401	1
28-39	0.044401	1
40-51	0.039325	1
52+	0.039325	1
**HPV 58**		
1-15	0.065572	1
16-27	0.05443	1
28-39	0.053968	1
40-51	0.033319	1
52+	0.033319	1
**High Risk HPV**		
1-15	0.080766	1
16-27	0.066633	1
28-39	0.053972	1
40-51	0.049229	1
52+	0.005094	1

**Low Risk HPV**		
1-15	0.051888	1
16-27	0.050005	1
28-39	0.034649	1
40-51	0.034649	1
52-63	0.034649	1
64+	0.028608	1

HPV Progression to CIN2

HPV Type and time since infection, months	Baseline Value	Multiplier Value Among Top 50 Parameter Sets[a]
**HPV 16**		
1-15	0.001707	1
16-27	0.002422	1
28-39	0.002577	1
40-51	0.005518	1
52+	0.014998	1
**HPV 18**		
1-15	4.27E-05	1
16-27	0.000189	1
28-39	0.000189	1
40-51	0.007733	1
52+	0.007733	1
**HPV 31**		
1-15	0.000262	1
16-27	0.00278	1
28-39	0.003091	1
40-51	0.006931	1
52+	0.006931	1
**HPV 33**		
1-15	0.000719	1
16-27	0.000719	1
28-39	0.004939	1
40-51	0.004939	1
52+	0.004939	1
**HPV 45**		
1-15	0	1
16-27	0	1

28-39	0.002258	1
40-51	0.005333	1
52+	0.005333	1
**HPV 52**		
1-15	0.000884	1
16-27	0.001681	1
28-39	0.001681	1
40-51	0.001978	1
52+	0.005678	1
**HPV 58**		
1-15	0.000587	1
16-27	0.002468	1
28-39	0.002468	1
40-51	0.004617	1
52+	0.010246	1
**High Risk HPV**		
1-15	0.000126	1
16-27	0.000373	1
28-39	0.00196	1
40-51	0.00196	1
52+	0.00196	1
**Low Risk HPV**		
1-15	0.000205	1
16-27	0.000291	1
28-39	0.00031	1
40-51	0.000663	1
52+	0.000663	1

HPV progression to CIN3

HPV Type and time since infection, months	Baseline Value	Multiplier Value Among Top 50 Parameter Sets[a]
**HPV 16**		
1-15	0.000569	1
16-27	0.000808	1
28-39	0.00086	1
40-51	0.001843	1
52+	0.005024	1
**HPV 18**		

1-15	1.07E-05	1
16-27	4.72E-05	1
28-39	4.72E-05	1
40-51	0.001939	1
52+	0.001939	1
**HPV 31**		
1-15	6.55E-05	1
16-27	0.000696	1
28-39	0.000774	1
40-51	0.001737	1
52+	0.001737	1
**HPV 33**		
1-15	0.00018	1
16-27	0.00018	1
28-39	0.001237	1
40-51	0.001237	1
52+	0.001237	1
**HPV 45**		
1-15	0	1
16-27	0	1
28-39	0.000565	1
40-51	0.001336	1
52+	0.001336	1
**HPV 52**		
1-15	0.000221	1
16-27	0.000421	1
28-39	0.000421	1
40-51	0.000495	1
52+	0.001423	1
**HPV 58**		
1-15	0.000147	1
16-27	0.000618	1
28-39	0.000618	1
40-51	0.001156	1
52+	0.002571	1
**High Risk HPV**		
1-15	3.16E-05	1

16-27	9.32E-05	1
28-39	0.00049	1
40-51	0.00049	1
52+	0.00049	1
**Low Risk HPV**		
1-15	2.28E-05	1
16-27	3.23E-05	1
28-39	3.44E-05	1
40-51	7.37E-05	1
52+	7.37E-05	1

Regression of CIN2 to NL

**HPV Type and time since infection, years**	Baseline Value		Range of Multiplier Values Among Top 50 Parameter Sets[a]	
	Min	Max	Min	Max
**HPV 16**				
1-5	0.05	0.523578	0.500863	0.687389
6-10	0.035	0.523578	0.500863	0.687389
11-20	0.013	0.523578	0.500863	0.687389
21-29	0.0005	0.523578	0.500863	0.687389
30-39	0.0001	0.523578	0.500863	0.687389
40+	0.00005	0.523578	0.500863	0.687389
**All Other HPV Types**				
1-5	0.05	0.523578	0.527319	3.70898
6-10	0.035	0.523578	0.527319	3.70898
11-20	0.013	0.523578	0.527319	3.70898
21-29	0.0005	0.523578	0.527319	3.70898
30-39	0.0001	0.523578	0.527319	3.70898
40+	0.00005	0.523578	0.527319	3.70898

Regression of CIN3 to NL

**HPV Type and time since infection, years**	Baseline Value		Range of Multiplier Values Among Top 50 Parameter Sets[a]	
	Min	Max	Min	Max
**HPV 16**				
1-5	0.025	0.639221	0.50664	1.67845
6-10	0.0175	0.639221	0.50664	1.67845
11-20	0.0065	0.639221	0.50664	1.67845

21-29	0.00025	0.639221	0.50664	1.67845
30-39	0.00005	0.639221	0.50664	1.67845
40+	0.000025	0.639221	0.50664	1.67845
**All Other HPV Types**				
1-5	0.025	0.639221	0.577736	3.9781
6-10	0.0175	0.639221	0.577736	3.9781
11-20	0.0065	0.639221	0.577736	3.9781
21-29	0.00025	0.639221	0.577736	3.9781
30-39	0.00005	0.639221	0.577736	3.9781
40+	0.000025	0.639221	0.577736	3.9781

Progression of CIN2 to CA

**HPV Type and time since infection, years**	Baseline Value	Range of Multiplier Values Among Top 50 Parameter Sets[a]	
		Min	Max
**HPV16**			
1-5	3.29E-05	1.00901	1.49572
6-10	3.56E-05	1.00901	1.49572
11-20	0.000857	1.00901	1.49572
21-29	0.002506	1.00901	1.49572
30-34	0.005011	1.00901	1.49572
35-39	0.005429	1.00901	1.49572
40-44	0.011693	1.00901	1.49572
45-49	0.012528	1.00901	1.49572
50+	0.33408	1.00901	1.49572
**HPV 18**			
1-5	3.29E-05	1.00901	1.49572
6-10	3.56E-05	1.00901	1.49572
11-20	0.000571	1.00901	1.49572
21-29	0.002506	1.00901	1.49572
30-34	0.005011	1.00901	1.49572
35-39	0.005429	1.00901	1.49572
40-44	0.011693	1.00901	1.49572
45-49	0.012528	1.00901	1.49572
50+	0.33408	1.00901	1.49572
**HPV 31**			
1-5	2.2E-05	1.00901	1.49572
6-10	2.38E-05	1.00901	1.49572
11-20	0.000857	1.00901	1.49572

21-29	0.00167	1.00901	1.49572
30-34	0.003341	1.00901	1.49572
35-39	0.003619	1.00901	1.49572
40-44	0.007795	1.00901	1.49572
45-49	0.008352	1.00901	1.49572
50+	0.22272	1.00901	1.49572
**HPV 33**			
1-5	3.29E-05	1.00901	1.49572
6-10	3.56E-05	1.00901	1.49572
11-20	0.000571	1.00901	1.49572
21-29	0.002506	1.00901	1.49572
30-34	0.005011	1.00901	1.49572
35-39	0.005429	1.00901	1.49572
40-44	0.011693	1.00901	1.49572
45-49	0.012528	1.00901	1.49572
50+	0.33408	1.00901	1.49572
**HPV 45**			
1-5	2.2E-05	1.00901	1.49572
6-10	2.38E-05	1.00901	1.49572
11-20	0.000571	1.00901	1.49572
21-29	0.00167	1.00901	1.49572
30-34	0.003341	1.00901	1.49572
35-39	0.003619	1.00901	1.49572
40-44	0.007795	1.00901	1.49572
45-49	0.008352	1.00901	1.49572
50+	0.22272	1.00901	1.49572
**HPV 52**			
1-5	2.2E-05	1.00901	1.49572
6-10	2.38E-05	1.00901	1.49572
11-20	0.000571	1.00901	1.49572
21-29	0.00167	1.00901	1.49572
30-34	0.003341	1.00901	1.49572
35-39	0.003619	1.00901	1.49572
40-44	0.007795	1.00901	1.49572
45-49	0.008352	1.00901	1.49572
50+	0.22272	1.00901	1.49572
**HPV 58**			
1-5	2.2E-05	1.00901	1.49572
6-10	2.38E-05	1.00901	1.49572

11-20	0	1.00901	1.49572
21-29	0.00167	1.00901	1.49572
30-34	0.003341	1.00901	1.49572
35-39	0.003619	1.00901	1.49572
40-44	0.007795	1.00901	1.49572
45-49	0.008352	1.00901	1.49572
50+	0.22272	1.00901	1.49572
**High Risk HPV**			
1-5	2.2E-05	1.00901	1.49572
6-10	2.38E-05	1.00901	1.49572
11-20	0.000571	1.00901	1.49572
21-29	0.00167	1.00901	1.49572
30-34	0.003341	1.00901	1.49572
35-39	0.003619	1.00901	1.49572
40-44	0.007795	1.00901	1.49572
45-49	0.008352	1.00901	1.49572
50+	0.22272	1.00901	1.49572

Progression of CIN3 to CA

**HPV Type and time since infection, years**	Baseline Value	Range of Multiplier Values Among Top 50 Parameter Sets[a]	
		Min	Max
**HPV 16**			
1-5	0.000165	1.00199	1.48007
6-10	0.000178	1.00199	1.48007
11-20	0.004284	1.00199	1.48007
21-29	0.012528	1.00199	1.48007
30-34	0.025056	1.00199	1.48007
35-39	0.027144	1.00199	1.48007
40-44	0.058464	1.00199	1.48007
45-49	0.06264	1.00199	1.48007
50+	0.33408	1.00199	1.48007
**HPV 18**			
1-5	0.000165	1.00199	1.48007
6-10	0.000178	1.00199	1.48007
11-20	0.004284	1.00199	1.48007
21-29	0.012528	1.00199	1.48007
30-34	0.025056	1.00199	1.48007
35-39	0.027144	1.00199	1.48007
40-44	0.058464	1.00199	1.48007
45-49	0.06264	1.00199	1.48007

50+	0.33408	1.00199	1.48007
**HPV 31**			
1-5	0.00011	1.00199	1.48007
6-10	0.000119	1.00199	1.48007
11-20	0.002856	1.00199	1.48007
21-29	0.008352	1.00199	1.48007
30-34	0.016704	1.00199	1.48007
35-39	0.018096	1.00199	1.48007
40-44	0.038976	1.00199	1.48007
45-49	0.04176	1.00199	1.48007
50+	0.22272	1.00199	1.48007
**HPV 33**			
1-5	0.000165	1.00199	1.48007
6-10	0.000178	1.00199	1.48007
11-20	0.004284	1.00199	1.48007
21-29	0.012528	1.00199	1.48007
30-34	0.025056	1.00199	1.48007
35-39	0.027144	1.00199	1.48007
40-44	0.058464	1.00199	1.48007
45-49	0.06264	1.00199	1.48007
50+	0.33408	1.00199	1.48007
**HPV 45**			
1-5	0.00011	1.00199	1.48007
6-10	0.000119	1.00199	1.48007
11-20	0.002856	1.00199	1.48007
21-29	0.008352	1.00199	1.48007
30-34	0.016704	1.00199	1.48007
35-39	0.018096	1.00199	1.48007
40-44	0.038976	1.00199	1.48007
45-49	0.04176	1.00199	1.48007
50+	0.22272	1.00199	1.48007
**HPV 52**			
1-5	0.00011	1.00199	1.48007
6-10	0.000119	1.00199	1.48007
11-20	0.002856	1.00199	1.48007
21-29	0.008352	1.00199	1.48007
30-34	0.016704	1.00199	1.48007
35-39	0.018096	1.00199	1.48007
40-44	0.038976	1.00199	1.48007

45-49	0.04176	1.00199	1.48007
50+	0.22272	1.00199	1.48007
**HPV 58**			
1-5	0.00011	1.00199	1.48007
6-10	0.000119	1.00199	1.48007
11-20	0.002856	1.00199	1.48007
21-29	0.008352	1.00199	1.48007
30-34	0.016704	1.00199	1.48007
35-39	0.018096	1.00199	1.48007
40-44	0.038976	1.00199	1.48007
45-49	0.04176	1.00199	1.48007
50+	0.22272	1.00199	1.48007
**High Risk HPV**			
1-5	0.00011	1.00199	1.48007
6-10	0.000119	1.00199	1.48007
11-20	0.002856	1.00199	1.48007
21-29	0.008352	1.00199	1.48007
30-34	0.016704	1.00199	1.48007
35-39	0.018096	1.00199	1.48007
40-44	0.038976	1.00199	1.48007
45-49	0.04176	1.00199	1.48007
50+	0.22272	1.00199	1.48007

Progression of invasive cancer stages

	Baseline Value
Local to regional	0.02
Regional to distant	0.025

Invasive cancer mortality

Local	0.005876
Regional	0.015137
Distant	0.065519

Probability of symptom detection

Local	0.0039
Regional	0.1333
Distant	0.1746

3

[a] Multiplier values were drawn from the identified plausible search ranges and applied to baseline input probabilities; the values presented represent the multiplier values that yielded the 50 best fits to the epidemiologic calibration targets.

Table A.4. Screening: Direct medical costs per procedure (2015 US$).[a]

Cost category	HPV test (provider-collection)[b]	HPV test (self-collection)[b]	Cytology	VIA triage	Source
Clinical staff	3.81	2.88	3.81	3.81	START-UP
Clinical supplies	6.72	6.72	0.82	0.36	Scale-Up (HPV);[c] START-UP (Cytology/VIA)
Clinical equipment	0.01	0.01	0.01	0.02	
Lab staff	0.41	0.41	1.37	0.00	Scale-Up (transport personnel);[d] START-UP (other)
Lab supplies	0.81	0.81	0.92	0.00	Scale-Up (transport fuel);[e] START-UP (other)
Lab equipment	0.21	0.21	0.32	0.00	START-UP
**Total direct medical cost**	**11.96**	**11.04**	**7.26**	**4.19**	

[a] HPV: human papillomavirus; Scale-Up: Scale-Up demonstration project (2015); START-UP: Screening Technologies to Advance Rapid Testing for Cervical Cancer Prevention–Utility and Program Planning demonstration project (2010); US$: United States dollars; VIA: visual inspection with acetic acid.

[b] HPV testing with both provider-collection of cervical samples and self-collection of vaginal samples was assumed to take place at the clinic. The difference in costs between collection modalities is due to reduced clinical staff time when a woman self-collects her own sample.

[c] The cost of clinical supplies for HPV testing was drawn from the Scale-Up project and includes the test kit, plate sealers, brush, collection medium, pipette tips (3 types), gloves, and towels to decant and dry.

[d] The cost of laboratory staff time was drawn from the START-UP project, with the exception of preparing and transporting samples to the laboratory, which was drawn from the Scale-Up project. Staff time spent preparing samples for transport in the Scale-Up project was based on project staff estimates in each of the three departments (Carazo, Chontales, and Chinandega), which ranged from 5 to 7 minutes per sample. Staff time for round-trip transport of samples was averaged across 9 to 10 facilities in each of the three departments, and valued based on the average monthly wage of the personnel responsible for transporting the samples in each department. We derived an average cost per sample prepared and transported for each department (assuming the cost of staff time per sample was equivalent for HPV and cytology specimens), and then averaged these costs across the three departments.

[e] For HPV testing, all lab supplies are bundled in the category of clinical supplies, with the exception of fuel for laboratory transport, which is included under lab supplies. For both HPV and cytology testing, we assumed the same cost of fuel per sample. Using Scale-Up project staff estimates of the number of samples transported per load, the distance to the lab from 9 to 10 facilities in each department, and the usual type of vehicle used in each department, we derived the average fuel cost per sample using the average fuel economy of major vehicle types [16] and the average gas price per liter in Nicaragua [12].

Table A.5. Diagnosis and Treatment of Precancer: Direct medical costs per procedure (2015 US$).[a]

Cost category	Colposcopy	Colposcopy and biopsy	Cryotherapy	LEEP	Source
Clinical staff	5.92	6.29	7.54	7.34	START-UP
Clinical supplies	0.38	1.39	3.54	36.05	Scale-Up (cryotherapy gas);[b] START-UP (other)
Drugs	0.13	0.13	0.30	0.10	START-UP
Clinical equipment	0.49	0.53	9.13	13.29	Scale-Up (cryotherapy unit);[c] START-UP (other)
Lab staff	0.00	4.09	0.00	4.09	START-UP
Lab supplies	0.00	3.35	0.00	3.35	START-UP
Lab equipment	0.00	4.12	0.00	4.12	START-UP
**Total direct medical cost**	**6.91**	**19.91**	**18.16**	**68.36**	

[a] LEEP: loop electrosurgical excision procedure; Scale-Up: Scale-Up demonstration project (2015); START-UP: Screening Technologies to Advance Rapid Testing for Cervical Cancer Prevention–Utility and Program Planning demonstration project (2010); US$: United States dollars.

[b] We used estimates from Scale-Up project staff to inform the cost of gas used per cryotherapy procedure, based on the cost of 1 cylinder and the number of women treated per tank. We did not include the cost of the tank deposit.

[c] To estimate the cost per procedure of a cryotherapy unit, we assumed this equipment item was a tradable good, and accordingly converted the purchase price from local currency units to US$ using the official exchange rate. We annualized the cost with a 3% interest rate and assumed an economic life of 5 years. To derive the number of procedures per machine per year across the three departments considered, we divided the annual number of cryotherapy procedures by the number of cryotherapy units. We then divided the annualized cryotherapy unit cost by the number of procedures per machine per year.

Table A.6. Location of service delivery for screening, diagnosis, and treatment of precancerous lesions and cancer.[a]

Procedure	Location of services
HPV DNA test	Screening facility
Cytology test	Screening facility
VIA test	Referral facility
Colposcopy/biopsy	Referral facility
Cryotherapy	Referral facility
LEEP	Referral facility
Follow-up visits (after cryotherapy or LEEP)	Primary facility
Cancer treatment	Tertiary facility

[a] HPV: human papillomavirus; LEEP: loop electrosurgical excision procedure; VIA: visual inspection with acetic acid.

Tabe A.7. Women's time estimates.[a]

Visit	Minutes	Cost (2015 US$)[b]	Source
**Screening**[c]			
Wait time	33	0.48	Scale-Up
Procedure time	20	0.29	START-UP
Transport time (round-trip)	57	0.82	Scale-Up
**Results**[c]			
Wait time	33	0.48	Scale-Up
Procedure time	2	0.03	START-UP
Transport time (round-trip)	57	0.82	Scale-Up
**Cryotherapy**[c]			
Wait time	120	1.75	Scale-Up
Procedure time	35	0.51	START-UP
Transport time (round-trip)	213	3.10	Scale-Up
**Colposcopy/biopsy**			
Wait time	120	1.75	Scale-Up
Procedure time	37	0.54	START-UP
Transport time (round-trip)	213	3.10	Scale-Up
**LEEP**			
Wait time	120	1.75	Scale-Up
Procedure time	25	0.36	START-UP
Transport time (round-trip)	213	3.10	Scale-Up

[a] LEEP: loop electrosurgical excision procedure; Scale-Up: Scale-Up demonstration project (2015); START-UP: Screening Technologies to Advance Rapid Testing for Cervical Cancer Prevention–Utility and Program Planning demonstration project (2010); US$: United States dollars.

[b] Women's time was valued using Nicaragua's 2013 monthly minimum wage [13], converted to 2015 US$. We converted this to an hourly wage rate assuming 171 hours of work per month (40 hours per week).

[c] Wait and transport time costs were accrued for each visit to a health facility, but we did not double-count wait and transport times when multiple procedures were performed in the same visit (e.g., VIA triage of screen-positive women and cryotherapy of VIA+ women accrued the wait time and transport time costs associated with a single visit to a referral facility, but accrued the time costs of both procedures.)

Table A.8. Women's transportation costs.[a]

Facility[b]	Round-trip transportation cost (2015 US$)	Source
Screening facility	0.41	Scale-Up
Referral facility	2.81	Scale-Up

[a] Scale-Up: Scale-Up demonstration project (2015); US$: United States dollar.
[b] Screening with cytology or HPV testing was assumed to take place at a screening facility (i.e., a primary level health facility). Triage testing with visual inspection with acetic acid (VIA), cryotherapy, colposcopy/biopsy, and loop electrosurgical excision procedure (LEEP) were assumed to take place at referral facilities.

Table A.9. Programmatic training costs for HPV-based screening.[a]

Type of training	Total cost of supplies/materials[b] (2015 US$)	Total cost of personnel time[c] (2015 US$)	Per woman cost[d] (2015 US$)
Health care personnel offering careHPV	2,758	5,784	0.09
Laboratory technicians processing careHPV assays	3,503	526	0.04
Community outreach workers promoting and offering self-collection	2,662	3,618	0.08
Health care providers offering VIA and cryotherapy	8,103	16,069	1.51

[a] HPV: human papillomavirus; US$: United States dollars; VIA: visual inspection with acetic acid.

[b] The total cost of supplies, materials, transportation, and refreshments for all training sessions of each type was converted from 2015 local currency units using the official exchange rate.

[c] The total cost of personnel time includes time costs for both the trainer and the trainees, valued using average monthly salary rates of each type of personnel present (and the proportion of trainees of each personnel type) and based on the length of the training session.

[d] To derive an average health care personnel training cost per woman screened, we divided the total training cost for health care personnel by 100,000, corresponding to the number of women screened during the Scale-Up project. To derive an average laboratory technician training cost per woman screened, we divided the total training cost for laboratory technicians by 100,000. To derive an average community outreach worker training cost per woman screened with self-collection, we divided the total training cost for outreach workers by 80,000, corresponding to the number of women who participated in self-collection of HPV samples (due to lack of data on self-collection in a community setting, in the base case analysis we assumed self-collection took place at the clinic; however, we assumed outreach workers would be involved in mobilizing and educating women about self-collection, and conservatively included the cost of training so as to represent a known programmatic cost as self-collection efforts shift to also include community settings). To derive an average health care provider training cost per woman receiving VIA or cryotherapy, we divided the total VIA/cryotherapy training cost for health care providers by 16,000, corresponding to the approximate number of women who tested HPV-positive in Scale-Up and would thus require visual assessment and cryotherapy.

Table A.10. Cancer treatment costs (2015 US$).

Cancer stage	Local cancer	Regional cancer	Distant cancer
Direct medical costs[a]	943.62	917.83	917.83
Woman and support person time	345.77	639.14	639.14
Transportation, temporary housing, and meals	196.78	389.50	389.50
**Total cancer treatment cost**	**1486**	**1946**	**1946**

[a] Direct medical costs are based on the premise that 50% of local cancer patients presented with FIGO stage 1a, requiring radical hysterectomy, and 50% of local cancer patients presented with FIGO stage 1b/2a, requiring cobalt therapy followed by 5 chemotherapy sessions followed by brachytherapy. We assumed all women presenting with FIGO stage 2b or higher received cobalt therapy followed by 5 chemotherapy sessions followed by brachytherapy.

[b] Patient and support person time costs value time using the minimum wage in Nicaragua, as described in the text.

[c] Direct non-medical costs include round-trip transportation for visits to a cancer center and meals and temporary housing during the course of treatment.

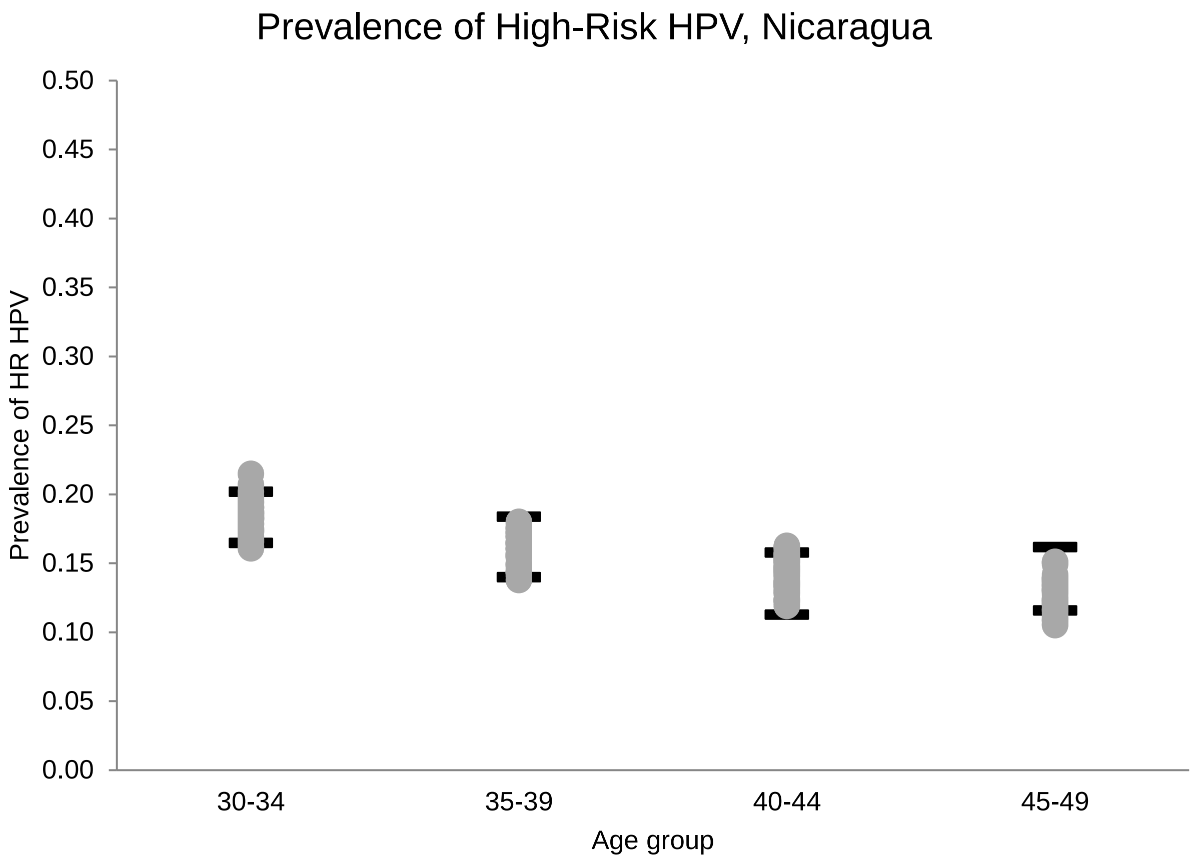

Figure A1. Selected model output from the top 50 input parameter sets compared with empirical data (i.e., calibration targets) on age-specific prevalence of high-risk HPV in Nicaragua, based on a relative light unit cut-off value of 0.5 in the START-UP studies [2, 15]. Bold lines represent the 95% confidence intervals around the empirical data, and gray circles represent model output from each of the top 50 input parameter sets.

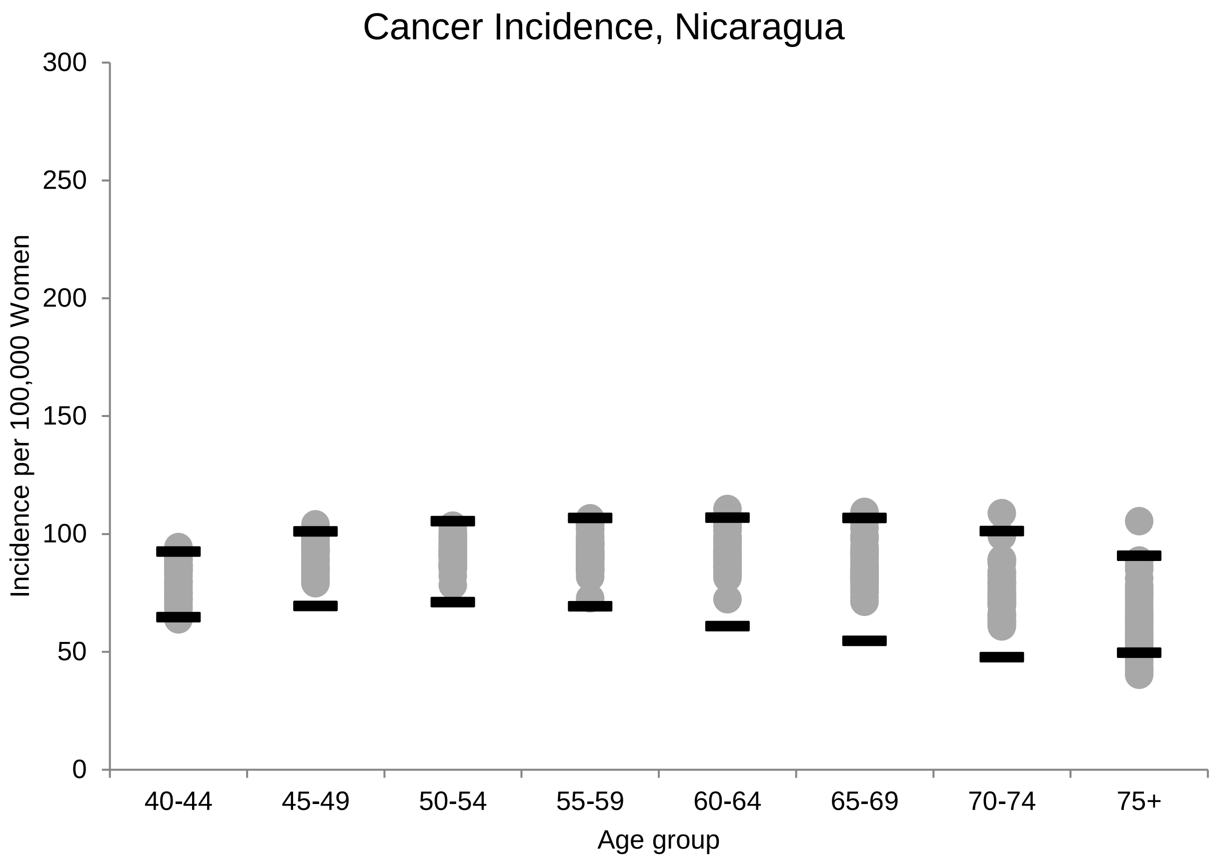

Figure A2. Selected model output from the top 50 input parameter sets compared with empirical data (i.e., calibration targets) on age-specific cancer incidence in Nicaragua [2, 11]. Bold lines represent the 95% confidence intervals around the empirical data, and gray circles represent model output from each of the top 50 input parameter sets.

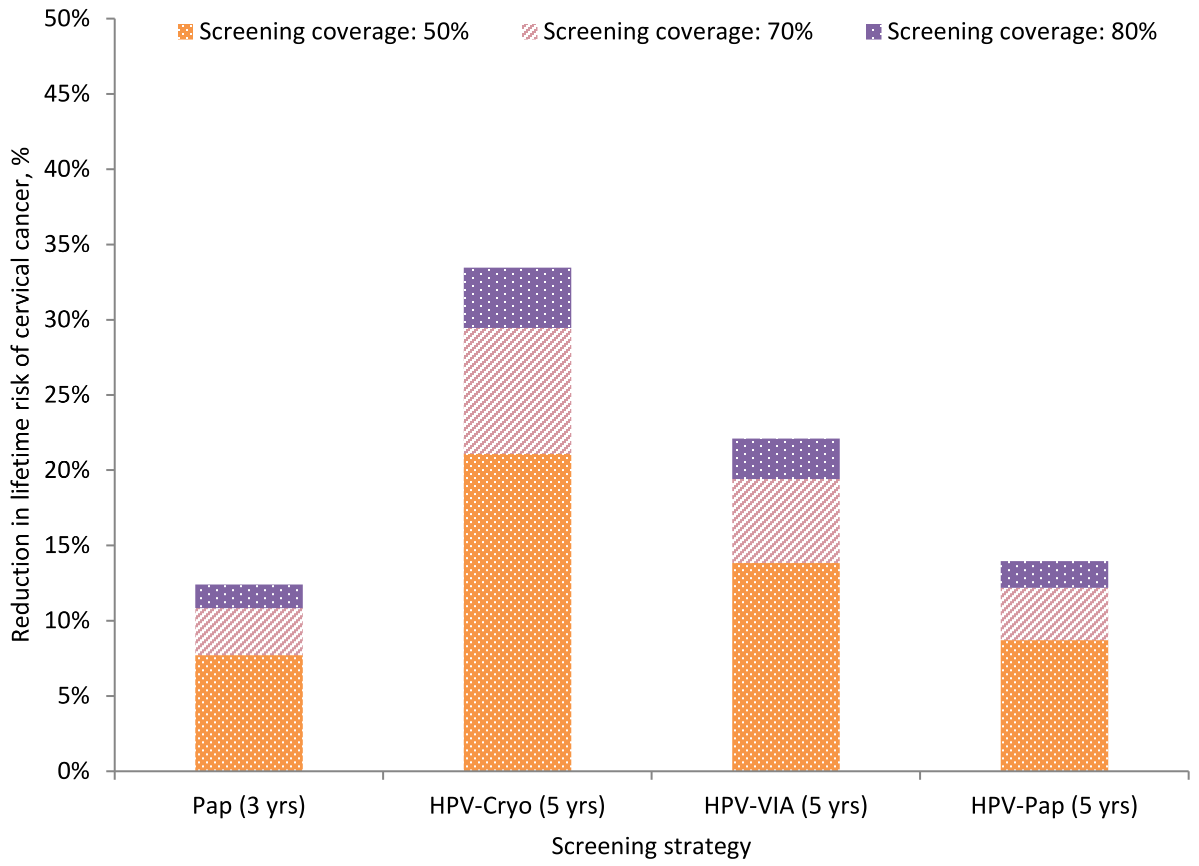

Figure A3. Reduction in lifetime risk of cervical cancer, by screening coverage level. Bars indicate the percent reduction in lifetime risk of cervical cancer for each screening strategy (Pap testing every 3 years; careHPV with cryotherapy for HPV-positive women every 5 years [HPV-Cryo]; careHPV every 5 years with visual inspection with acetic acid [VIA] triage of HPV-positive women [HPV-VIA]; and careHPV every 5 years with Pap triage of HPV-positive women [HPV-Pap]) as screening coverage of the target population increases.

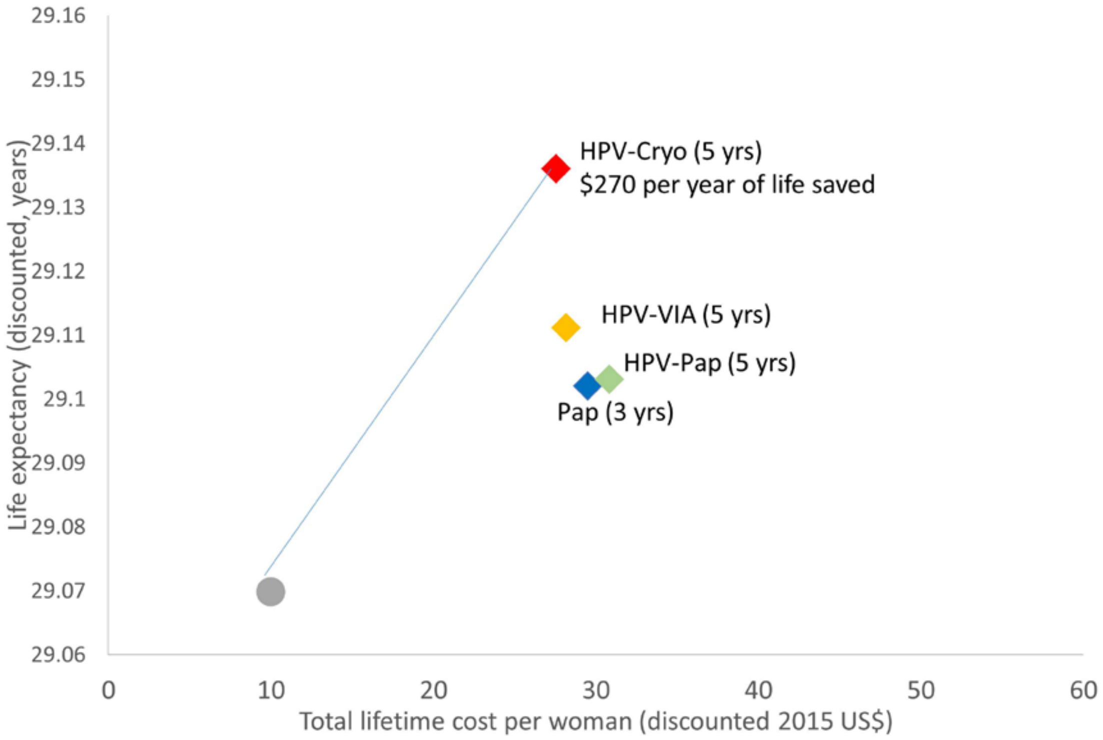

Figure A4. Cost-effectiveness analysis: Payer cost only. The graph displays the discounted lifetime costs (x-axis; in 2015 US$) and life expectancy (y-axis) associated with each screening strategy (Pap testing every 3 years; careHPV with cryotherapy for HPV-positive women every 5 years [HPV-Cryo]; careHPV every 5 years with visual inspection with acetic acid [VIA] triage of HPV-positive women [HPV-VIA]; and careHPV every 5 years with Pap triage of HPV-positive women [HPV-Pap]), when only costs to the payer are included (i.e., women's time and transportation costs are excluded). The cost-effectiveness associated with a change from one strategy to a more costly alternative is represented by the difference in cost divided by the difference in life expectancy associated with the two strategies. The curve indicates the strategies that are efficient because they are more effective and either 1) cost less; or 2) have a more attractive cost-effectiveness ratio than less effective options. The incremental cost-effectiveness ratio (ICER) is the reciprocal of the slope of the line connecting the two strategies under comparison. In this sensitivity analysis focused on costs to the payer, HPV-Cryo every 5 years was less costly and more effective than other screening strategies considered, and was thus a dominant strategy with an ICER of US$270 per year of life saved.

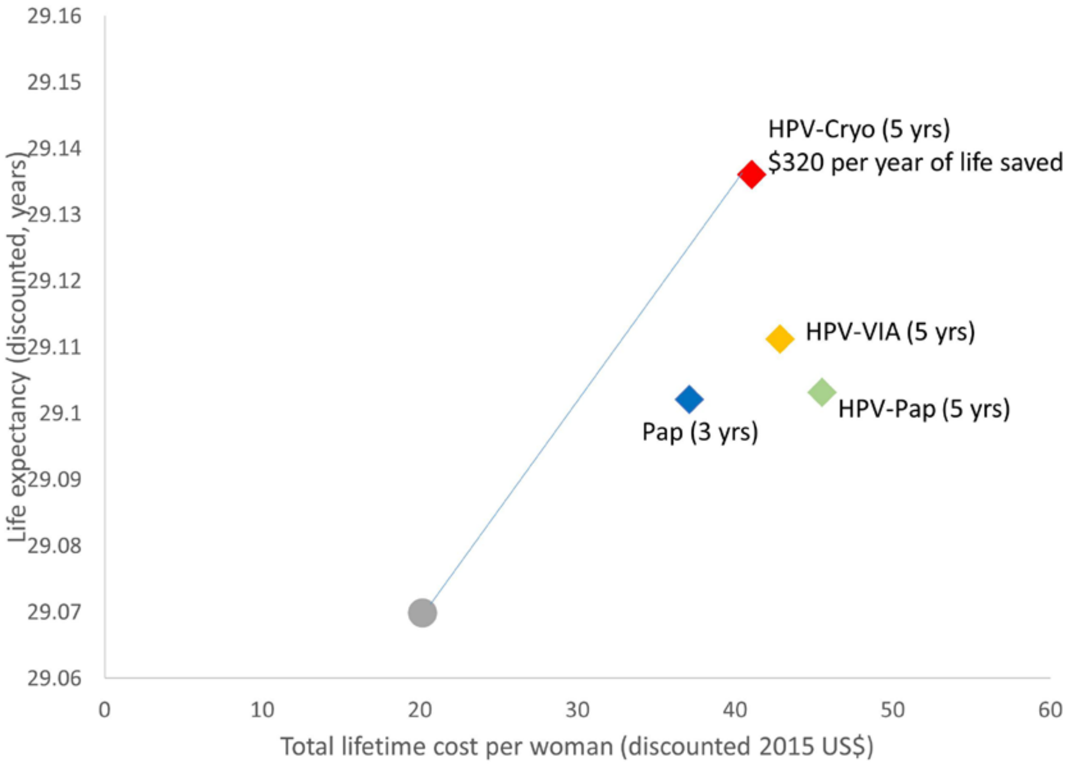

Figure A5. Cost-effectiveness analysis: Direct medical cost of Pap testing, US$3. The graph displays the discounted lifetime costs (x-axis; in 2015 US$) and life expectancy (y-axis) associated with each screening strategy (Pap testing every 3 years; careHPV with cryotherapy for HPV-positive women every 5 years [HPV-Cryo]; careHPV every 5 years with visual inspection with acetic acid [VIA] triage of HPV-positive women [HPV-VIA]; and careHPV every 5 years with Pap triage of HPV-positive women [HPV-Pap]), when the direct medical cost of Pap testing was assumed to be as low as US$3 (base case: US$7.26). The cost-effectiveness associated with a change from one strategy to a more costly alternative is represented by the difference in cost divided by the difference in life expectancy associated with the two strategies. The curve indicates the strategies that are efficient because they are more effective and either 1) cost less; or 2) have a more attractive cost-effectiveness ratio than less effective options. The incremental cost-effectiveness ratio (ICER) is the reciprocal of the slope of the line connecting the two strategies under comparison. In this sensitivity analysis examining the impact of a lower Pap test cost, HPV-Cryo every 5 years was more effective and had a more attractive cost-effectiveness ratio than Pap testing every 3 years; furthermore, HPV-Cryo was more effective and less costly than HPV-VIA and HPV-Pap, and was thus a dominant strategy with an ICER of US$320 per year of life saved.

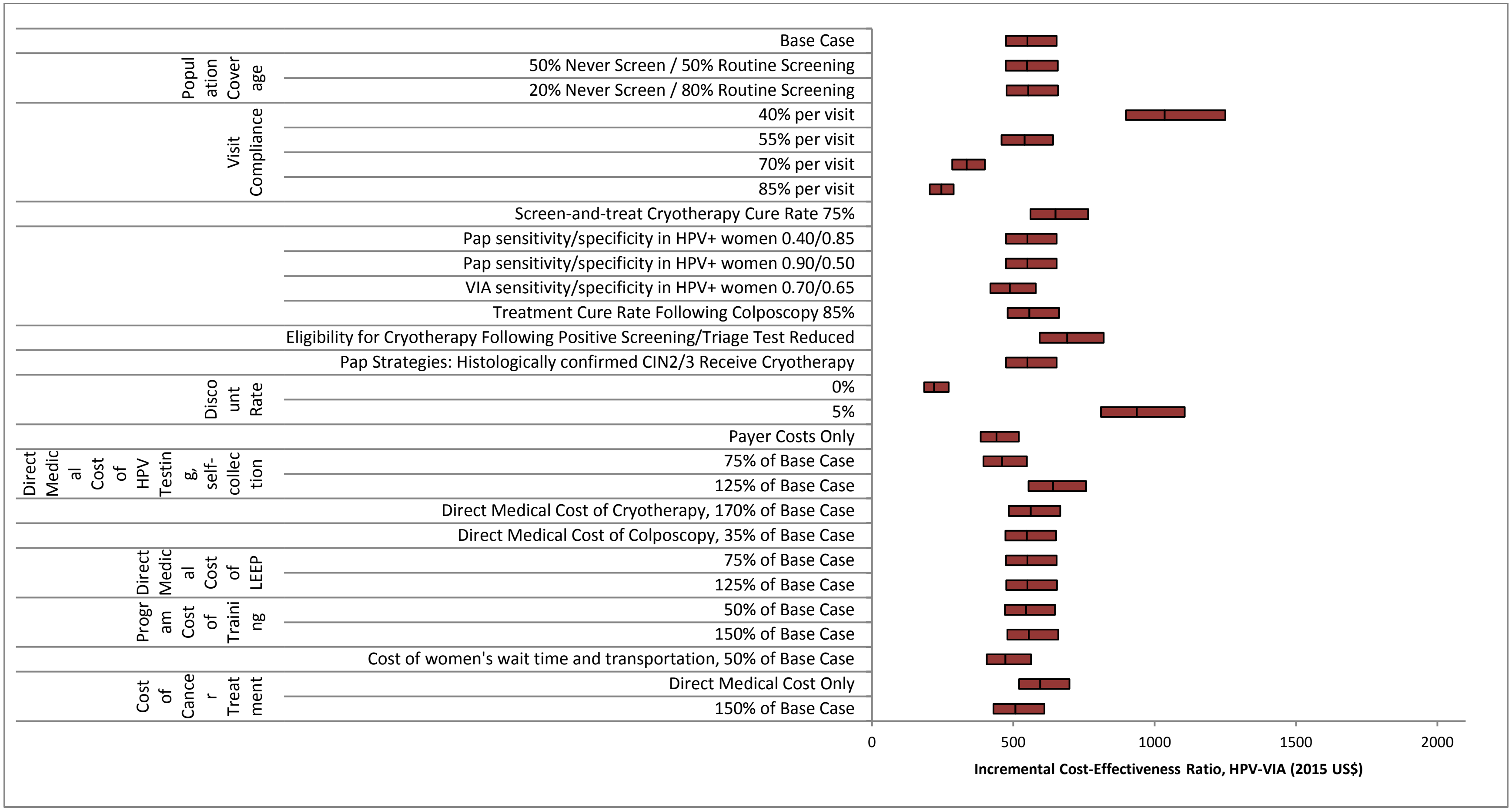

Figure A6. Base case and sensitivity analysis when HPV-Cryo is assumed to be unavailable: Incremental cost-effectiveness ratios, HPV-VIA strategy. Incremental cost-effectiveness ratios (ICERs) are presented (x-axis, 2015 US$ per year of life saved) for the base case and sensitivity analyses (y-axis), assuming HPV-Cryo is not available but Pap alone, HPV-VIA, and HPV-Pap are available. The orange bars represent the range of the ICERs for HPV-VIA every 5 years across the 50 input parameter sets, with the ICER of the mean costs divided by the mean effects demarcated by a black line. Nicaragua’s per capita gross domestic product (GDP) is US$2,090, as a possible threshold that designates interventions as “very cost-effective”.

References

1. Campos, N.G., et al., *An updated natural history model of cervical cancer: derivation of model parameters.* Am J Epidemiol, 2014. **180**(5): p. 545-55.
2. Campos, N.G., Tsu, V., Jeronimo, J., Mvundura, M., Lee, K., Kim, J.J., *When and how often to screen for cervical cancer in three low- and middle-income countries: A cost-effectiveness analysis.* Papillomavirus Research, 2015.
3. Munoz, N., et al., *Incidence, duration, and determinants of cervical human papillomavirus infection in a cohort of Colombian women with normal cytological results.* J Infect Dis, 2004. **190**(12): p. 2077-87.
4. McCredie, M.R., et al., *Natural history of cervical neoplasia and risk of invasive cancer in women with cervical intraepithelial neoplasia 3: a retrospective cohort study.* Lancet Oncol, 2008. **9**(5): p. 425-34.
5. Meyskens, F.L., Jr., et al., *Enhancement of regression of cervical intraepithelial neoplasia II (moderate dysplasia) with topically applied all-trans-retinoic acid: a randomized trial.* J Natl Cancer Inst, 1994. **86**(7): p. 539-43.
6. Keefe, K.A., et al., *A randomized, double blind, Phase III trial using oral beta-carotene supplementation for women with high-grade cervical intraepithelial neoplasia.* Cancer Epidemiol Biomarkers Prev, 2001. **10**(10): p. 1029-35.
7. Castle, P.E., et al., *Evidence for frequent regression of cervical intraepithelial neoplasia-grade 2.* Obstet Gynecol, 2009. **113**(1): p. 18-25.
8. Wang, S.M., et al., *Six-year regression and progression of cervical lesions of different human papillomavirus viral loads in varied histological diagnoses.* Int J Gynecol Cancer, 2013. **23**(4): p. 716-23.
9. Moscicki, A.B., et al., *Rate of and risks for regression of cervical intraepithelial neoplasia 2 in adolescents and young women.* Obstet Gynecol, 2010. **116**(6): p. 1373-80.
10. Herrero, R., et al., *Rationale and design of a community-based double-blind randomized clinical trial of an HPV 16 and 18 vaccine in Guanacaste, Costa Rica.* Vaccine, 2008. **26**(37): p. 4795-808.
11. Ferlay, J., et al., *GLOBOCAN 2012 v1.0, Cancer Incidence and Mortality Worldwide: IARC CancerBase No. 11 [Internet]*, 2013, International Agency for Research on Cancer: Lyon, France.
12. *World Development Indicators*, 2016, World Bank.
13. International Labour Organization. *Country Profiles: Nicaragua*, 2016.
14. Campos, N.G., et al., *The comparative and cost-effectiveness of HPV-based cervical cancer screening algorithms in El Salvador.* Int J Cancer, 2015.
15. Jeronimo, J., et al., *A multicountry evaluation of careHPV testing, visual inspection with acetic acid, and papanicolaou testing for the detection of cervical cancer.* Int J Gynecol Cancer, 2014. **24**(3): p. 576-85.
16. United States Department of Energy. *Average Fuel Economy of Major Vehicle Categories*. August 1, 2016]; Available from: http://www.afdc.energy.gov/data/10310.
