# Supplementary material for: Cost-effectiveness of HPV-based cervical cancer screening in the public health system in Nicaragua
Source: BMJ Open. 2017 Jun 15;7(6):e015048. doi: 10.1136/bmjopen-2016-015048 (PMC5623348; doi:10.1136/bmjopen-2016-015048)
Supplement: Author's manuscript [file bmjopen-2016-015048.draft_revisions.pdf]

BMJ Open

The Cost-Effectiveness of HPV-Based Cervical Cancer Screening in the Public Health System in Nicaragua

Journal:	*BMJ Open*
Manuscript ID	bmjopen-2016-015048
Article Type:	Research
Date Submitted by the Author:	04-Nov-2016
Complete List of Authors:	Campos, Nicole; Harvard T.H. Chan School of Public Health, Health Policy and Management<br>Mvundura, Mercy; PATH, Devices and Tools Program<br>Jeronimo, Jose; PATH, Reproductive Global Health Program<br>Holme, Francesca; PATH, Reproductive Health Global Program<br>Vodicka, Elisabeth; University of Washington, School of Pharmacy<br>Kim, Jane; Harvard School of Public Health, Health Policy and Management
**Primary Subject Heading**:	Health economics
Secondary Subject Heading:	Global health, Health economics, Health policy, Obstetrics and gynaecology, Public health
Keywords:	HEALTH ECONOMICS, Epidemiology < ONCOLOGY, PUBLIC HEALTH

SCHOLARONE™
Manuscripts

The Cost-Effectiveness of HPV-Based Cervical Cancer Screening in the Public Health System in Nicaragua

Nicole G. Campos, PhD, [a] Mercy Mvundura, PhD,[b] Jose Jeronimo, MD,[c] Francesca Holme, MPH,[c] Elisabeth Vodicka, MHA,[d] Jane J. Kim, PhD[a]

[a] Center for Health Decision Science, Harvard T.H. Chan School of Public Health, 718 Huntington Avenue, Boston, Massachusetts, USA

[b] PATH, Devices and Tools Program, P.O. Box 900922, Seattle, Washington, USA

[c] PATH, Reproductive Health Global Program, P.O. Box 900922, Seattle, Washington, USA

[d] University of Washington, School of Pharmacy, Seattle, Washington, USA

Corresponding author: Nicole G. Campos, 718 Huntington Avenue, Boston, MA 02115; e-mail: ncampos@hsph.harvard.edu; phone: 617-432-2019; fax: 617-432-0190

Word count (abstract): 300

Word count (text): 4,068

For peer review only - http://bmjopen.bmj.com/site/about/guidelines.xhtml

For peer review only

Abstract

Objectives: To evaluate the cost-effectiveness of HPV DNA testing (versus Pap-based screening) for cervical cancer screening in Nicaragua.

Design: A previously developed Monte Carlo simulation model of the natural history of HPV infection and cervical cancer was calibrated to epidemiologic data from Nicaragua. Cost data inputs were derived using a micro-costing approach in Carazo, Chontales, and Chinandega departments; test performance data were from a demonstration project in Masaya department.

Setting: Nicaragua's public health sector facilities.

Participants: Women aged 30 to 59 years.

Interventions: Screening strategies included 1) Pap testing every 3 years, with referral to colposcopy for women with an ASCUS+ result ("Pap"); 2) HPV testing every 5 years, with referral to cryotherapy for HPV-positive eligible women (HPV screen-and-treat, or "HPV-ST"); 3) HPV testing every 5 years, with referral to triage with visual inspection with acetic acid (VIA) for HPV-positive women ("HPV-VIA"); and 4) HPV testing every 5 years, with referral to Pap testing for HPV-positive women ("HPV-Pap").

Outcome measures: Reduction in lifetime risk of cancer; incremental cost-effectiveness ratios (ICER; 2015 US$ per year of life saved [YLS]).

Results: HPV-based screening strategies were more effective than Pap testing. HPV-ST was the least costly and most effective strategy, reducing lifetime cancer risk by 29.5% and outperforming HPV-VIA, HPV-Pap, and Pap only, which reduced cancer risk by 19.4%, 12.2%, and 10.8%, respectively. With an ICER of US$320/YLS, HPV-ST every 5 years would be very cost-effective using a threshold based on Nicaragua's per capita GDP of US$2,090. Findings were robust across sensitivity analyses on test performance, coverage, compliance, and cost parameters.

For peer review only - http://bmjopen.bmj.com/site/about/guidelines.xhtml

Conclusions: HPV testing is very cost-effective compared to Pap testing in Nicaragua, due to higher test sensitivity and the relatively lower number of visits required. Increasing compliance with recommended follow-up will further improve the health benefits and value for public health dollars.

For peer review only

For peer review only - http://bmjopen.bmj.com/site/about/guidelines.xhtml

Strengths and limitations of this study:

- Using implementation data from the Scale-Up project— which aims to facilitate institutionalization of HPV testing at the national level in Guatemala, Honduras, and Nicaragua—we estimated the cost-effectiveness of HPV testing in Nicaragua's public health system.
- The screening algorithms, as modeled, reflect the prototypical structure of a screening episode and the type of facility at which visits usually take place, but do not capture variation due to geography or health facility capacity.
- While we adopted a micro-costing approach to leverage data from implementation in Nicaragua, individual-level data for each woman were not available; furthermore, we did not have cost data associated with HPV self-collection in community settings, where most self-collection takes place.
- Despite these limitations, extensive sensitivity analyses on cost and screening assumptions indicate that HPV-ST is robustly the most efficient strategy.

Funding statement: This work was supported by the Bill & Melinda Gates Foundation.

Disclaimer: The findings and conclusions contained within are those of the authors and do not necessarily reflect positions or policies of the Bill & Melinda Gates Foundation. The funders had no role in study design; data collection, analysis, and interpretation; preparation of the manuscript; or decision to submit the article for publication.

Competing interests disclosed: All authors have completed the ICMJE uniform disclosure form at www.icmje.org/coi_disclosure.pdf and declare financial support from the Bill and Melinda Gates Foundation for the submitted work; JJ was the director of the START-UP demonstration projects and

For peer review only - http://bmjopen.bmj.com/site/about/guidelines.xhtml

received all tests used in the study as a donation from Qiagen; no other relationships or activities that could appear to have influenced the submitted work.

Contributors: All authors developed the analysis plan and interpreted the data. NC, MM, JJ, FH, and JK conceptualized the study. MM, JJ, FH, and EV collected implementation data. NC conducted data analysis and wrote the first draft of the report and revised subsequent drafts. JJ was the principal investigator of the START-UP and Scale-Up projects. JK was the principal investigator overseeing microsimulation model development. All authors contributed to and approved the final report.

Data sharing statement: A supplementary appendix is available.

For peer review only

For peer review only - http://bmjopen.bmj.com/site/about/guidelines.xhtml

Background

Cervical cancer is the leading cause of cancer among women in Nicaragua, with an estimated 934 cases and 424 deaths each year [1]. Yet cervical cancer is preventable through screening that allows for early detection and subsequent treatment of precancerous lesions caused by sexually transmitted infection with human papillomavirus (HPV). While most HPV infections clear spontaneously within 1 to 2 years, a persistent infection with one of approximately 15 oncogenic HPV genotypes may progress to precancer, which, if untreated, may become invasive cancer [2, 3].

In most high-income countries, routine screening with cervical cytology (i.e., Pap) testing has substantially reduced the incidence of cervical cancer [4]. However, due to its low sensitivity to detect precancer, Pap testing must be performed at regular frequent intervals in women of screening age. In low- and middle-income settings, where many women do not have access to routine primary health care and even fewer have access to higher-level facilities that offer diagnostic testing and treatment, Pap testing has not been effective at reducing cervical cancer incidence and mortality [5]. In Nicaragua, an estimated 31.5% of women aged 15 to 49 years have been screened within the last year, and nearly 30% of women in this age group have never been screened [6]. One recent survey found that 87% of women in León, Nicaragua, were informed of their Pap results, but of those who were referred to follow-up, only 67% received further care [7].

HPV DNA tests are highly sensitive to detect potentially oncogenic HPV infections and present an alternative to Pap-based screening. Because HPV-negative women are at very low risk for developing cervical cancer within the next 10 years [8], the interval between screenings can be extended to at least 5 years for this subset of women [9]. An additional advantage of HPV testing is that samples can be collected by a provider or by the woman herself, reducing the burden on health workers and time women spend seeking care and potentially increasing screening uptake [10-13]. Furthermore, a lower-cost HPV DNA test known as careHPV has been clinically validated [14, 15] and is now commercialized.

For peer review only - http://bmjopen.bmj.com/site/about/guidelines.xhtml

97 Given these potential benefits, the World Health Organization (WHO) recommends HPV testing for countries with sufficient resources [9].

In 2011, the Screening Technologies to Advance Rapid Testing for Cervical Cancer Prevention−Utility and Program Planning (START-UP) project in Nicaragua's Masaya department demonstrated that screening with careHPV could be effectively implemented in public sector health facilities [14]. The Ministry of Health of Nicaragua subsequently built on these initial efforts, incorporating HPV testing into public health care systems in three departments with technical assistance from PATH under the Scale-Up project. Adoption of HPV testing within Nicaragua's public health care system is taking place in three phases [16]. In Phase 1, partner organizations worked with the Ministry of Health to prepare for introduction of HPV screening into public health facilities by developing screening and treatment algorithms; creating educational materials; organizing training sessions for health workers and laboratory technicians; and bolstering referral and treatment systems for follow-up of screen-positive women. Phase 2 piloted screening with 10,000 HPV tests in order to identify and address barriers to implementation. Phase 3 will expand coverage to over 50,000 women within one year.

To inform decision makers considering the national adoption and scale-up of HPV testing within Nicaragua's public health sector, this study aimed to 1) estimate the economic cost of cervical cancer screening with careHPV testing; and 2) project the long-term health and economic impact and value (i.e., cost-effectiveness) of careHPV testing in Nicaragua relative to existing Pap-based screening.

Methods

Analytic overview

We used a micro-costing approach to measure and aggregate the cost of all resources used to provide cervical cancer screening at the level of the individual patient within the public health sector in Nicaragua. We considered direct medical costs (i.e., medical resources required for the intervention), direct non-medical costs (i.e., other resources consumed as part of the intervention, such as patient

For peer review only - http://bmjopen.bmj.com/site/about/guidelines.xhtml

transportation costs), and patient time costs (i.e., time spent traveling and waiting for or receiving care). These cost data were input into a previously developed Monte Carlo simulation model of the natural history of HPV infection and cervical cancer that was calibrated to epidemiologic data from Nicaragua [17, 18]. We then used the model to project the lifetime health and economic outcomes associated with careHPV testing, using three different algorithms for the management of women who test HPV-positive, and Pap-based screening for women aged 30 to 59 years.

Model outcomes included the lifetime risk of cervical cancer, total lifetime costs per woman (in 2015 United States dollars [US$]), and life expectancy. Incremental cost-effectiveness ratios (ICERs) were calculated by dividing the additional cost of a particular strategy by its additional health benefit, compared with the next most costly strategy. Dominated strategies (defined as more costly and either less effective or having a higher cost-effectiveness ratio than more effective strategies) were eliminated. There is no universal criterion that defines a threshold cost-effectiveness ratio, below which an intervention is considered good value for money; we considered an intervention with an ICER less than Nicaragua's 2015 per capita GDP of US$2,090 to be "very cost-effective", and an intervention with an ICER less than three times per capita GDP as "cost-effective" [19]. We followed guidelines for cost-effectiveness by adopting a societal perspective, including costs irrespective of the payer in order to capture the opportunity cost of resources used for the screening intervention. We discounted future costs and life-years at a rate of 3% per year to account for time preferences [20, 21].

Mathematical simulation model

Descriptions of the natural history model of HPV infection and cervical carcinogenesis and model parameterization process have been previously published [17, 18], but we summarize model features here. Individual girls enter the model at age 9 years, prior to initiating sexual activity, and face monthly transitions between mutually exclusive health states that reflect disease progression, including

For peer review only - http://bmjopen.bmj.com/site/about/guidelines.xhtml

type-specific HPV infection, grade of precancer (i.e., cervical intraepithelial neoplasia [CIN] grade 2 or 3), and stage of invasive cancer. Transition probabilities may vary by age, HPV type, duration of infection or precancerous lesion status, prior HPV infection, and exposure to screening and treatment of HPV or precancer. Cervical cancer can be detected through symptoms or screening. Death can occur from non-cervical causes or from cervical cancer after its onset. The model tracks each individual woman's health status, clinical events, and economic outcomes over her lifetime, and aggregates outcomes to estimate the expected costs and health outcomes over the lifetime of the cohort.

The model was calibrated to epidemiologic data on age-specific HPV prevalence and cervical cancer incidence from Nicaragua [1, 14, 17]. We estimated baseline "prior" input parameter values for natural history transitions using available longitudinal data, including age- and type-specific HPV incidence data from Colombia [22-25]. To reflect potential differences in parameters that may vary by setting (i.e., age- and type-specific HPV incidence, natural immunity following initial infection) and uncertainty in progression and regression of precancer, we set plausible bounds around these input values and performed repeated model simulations of disease natural history in the absence of any intervention. Each model simulation selected one random value within the bounds for each uncertain parameter, creating a unique natural history input parameter set. By summing the log-likelihood of model-projected outcomes for each parameter set relative to the epidemiologic data from Nicaragua, we computed a goodness-of-fit score. We selected the 50 top-fitting input parameter sets to use in analysis. Results are reported as the mean across the top 50 parameter sets, and ICERs are reported as the ratio of the mean costs divided by the mean effects of one strategy versus another across sets [26]. Further details on model parameterization, including calibration, are available in the **Appendix**.

Cervical cancer screening strategies

For peer review only

For peer review only - http://bmjopen.bmj.com/site/about/guidelines.xhtml

We considered the following screening strategies for women aged 30 to 59 years (**Figure 1**): 1) Pap testing every 3 years, with referral to colposcopy for all women with an atypical squamous cells of undetermined significance or worse (ASCUS+) result (hereafter referred to as "Pap"); 2) careHPV testing every 5 years, with referral to cryotherapy for all HPV-positive eligible women (HPV screen-and-treat, or "HPV-ST"); 3) careHPV testing every 5 years, with referral to triage with visual inspection with acetic acid (VIA) for all HPV-positive women ("HPV-VIA"); and 4) careHPV testing every 5 years, with referral to Pap testing for all HPV-positive women ("HPV-Pap"). The pathway of care for each strategy was based on patterns of care in the Scale-Up project, national screening guidelines, and WHO recommendations. We optimistically assumed 70% of women had access to routine screening and attended an initial visit at a screening facility (i.e., a primary health care facility). Women could then return to receive screening results and recommendations for any necessary follow-up care. Follow-up could include colposcopy, cryotherapy, or triage testing at a referral (i.e., higher level) facility, with the exception of Pap triage testing, which was assumed to take place at the screening clinic. At each encounter after the initial screening visit, we assumed 85% of women complied with each subsequent visit to a screening facility, while 40% complied with each subsequent visit to a referral facility, consistent with data from Phase 2 of the Scale-Up project. The minimum number of visits required for treatment in a single screening episode was 4 for Pap, 3 for HPV-ST, 3 for HPV-VIA, and 5 for HPV-Pap. In the HPV-VIA and HPV-Pap strategies, women who were HPV-positive but negative on the selected triage test were referred to repeat HPV testing in 1 year. In the HPV-ST and HPV-VIA strategies, women who were not eligible for treatment with cryotherapy based on visual assessment were referred to colposcopy with biopsy to rule out cancer; in the absence of cancer, these women were referred to treatment with loop electrosurgical excision procedure (LEEP) or cryotherapy.

Screening and treatment parameters are presented in **Table 1** [6, 14, 17, 27-32]. Screening test performance data were drawn from the START-UP project in Nicaragua to reflect local test

For peer review only - http://bmjopen.bmj.com/site/about/guidelines.xhtml

characteristics. While the START-UP project did not evaluate VIA and Pap as triage tests, we used the VIA and Pap positivity rates in HPV-positive women, along with published studies of triage test performance, to inform triage test sensitivity and specificity [33-38]; in the base case, we optimistically assumed high sensitivity of triage testing.

For all HPV testing strategies, we assumed 20% of women received provider-collection of cervical specimens and 80% of women self-collected vaginal specimens, consistent with the proportions in the Scale-Up project to date. We weighted cost and health outcomes for provider- and self-collection accordingly when aggregating results for the HPV strategies.

Cost data

All costs were converted to 2015 United States dollars (US$) using GDP deflators and the official exchange rate [39]. The direct medical costs of screening, diagnosis, and treatment of precancer were drawn from the START-UP study (Masaya department) and the Scale-Up project (Carazo, Chontales, and Chinandega departments). Direct medical costs included clinical staff time, clinical supplies, drugs, clinical equipment, laboratory staff time, laboratory supplies, and laboratory equipment. Direct non-medical costs included women's round-trip transportation costs to health facilities, and were based on estimates provided by Scale-Up project staff to represent average transportation costs in the Carazo, Chontales, and Chinandega departments. To account for the opportunity cost of women's time spent traveling to, waiting for, or receiving care, we used time estimates from the START-UP and Scale-Up projects, and valued women's time using Nicaragua's monthly minimum wage to serve as a proxy for the societal value of women's time. **Figure 2** displays the categorical breakdown of undiscounted costs for the screening visits over the course of a woman's screening-eligible years, with Pap versus careHPV testing.

For peer review only - http://bmjopen.bmj.com/site/about/guidelines.xhtml

Data on programmatic costs are limited, but for HPV strategies we included the cost of training sessions for health providers offering HPV-based screening, outreach workers, laboratory technicians, and providers offering VIA and cryotherapy. While women who self-collected HPV specimens in the Scale-Up project primarily did so in a community setting, micro-costing data were not available for self-collection performed outside of the clinic, so we assumed clinic-based self-collection. However, we conservatively included the cost of training outreach workers to represent a known programmatic cost as self-collection efforts are shifting to community settings.

Data on the costs of treating cervical cancer were unavailable for Nicaragua, so we estimated direct medical, direct non-medical, and patient and support person time costs using data from El Salvador [29].

Selected cost data are presented in **Table 1.** Further details on cost data are provided in the **Appendix**.

Sensitivity analyses

We performed sensitivity analysis to examine the impact of independently varying uncertain parameters, including Pap test performance, triage test performance in HPV-positive women, colposcopy performance, screening coverage, visit compliance, treatment effectiveness, and cost data. Ranges selected for sensitivity analysis are displayed in **Table 1**.

Results

Base case: Population-level health benefits and cost-effectiveness analysis

HPV-based screening strategies were more effective than Pap testing. Among the HPV strategies, HPV-ST (every 5 years) was the most effective strategy; under base case assumptions, it reduced the lifetime risk of cervical cancer by 29.5% on average (range: 25.2%−33.6%). HPV-VIA (every 5

For peer review only - http://bmjopen.bmj.com/site/about/guidelines.xhtml

years) reduced cancer risk by 19.4% (range: 16.2%–22.6%), while HPV-Pap reduced cancer risk by 12.2% (range: 10.2%–14.5%). Pap (every 3 years) reduced cancer risk by 10.8% (range: 8.7%–13.4%). Under base case assumptions, HPV-ST was both less costly and more effective than all other strategies, thus dominating HPV-VIA, HPV-Pap, and Pap alone. With an ICER of US$320 per year of life saved (YLS), HPV-ST every 5 years would be considered “very cost-effective” given Nicaragua’s per capita GDP of US$2,090. The total discounted lifetime cost per woman and life expectancy associated with each screening strategy is presented in **Figure 3**.

Sensitivity analysis

While HPV-ST remained the most effective strategy across all sensitivity analyses, the magnitude of reduction in lifetime risk of cancer was dependent upon screening coverage of the target population and compliance with recommended follow-up. When coverage was 50% and all other parameters were held constant at base case values, HPV-ST reduced cancer risk by an average of 21.1%; HPV-VIA, HPV-Pap, and Pap yielded average cancer risk reductions of 13.9%, 8.7%, and 7.7%, respectively. As coverage increased to 80%, all else being equal, HPV-ST reduced cancer risk by an average of 33.5%, while HPV-VIA, HPV-Pap, and Pap yielded average cancer risk reductions of 22.1%, 14.0%, and 12.4%, respectively (**Appendix**). **Figure 4** displays the impact of visit compliance on lifetime risk of cancer. When compliance with visits to all facilities (i.e., for both screening and referral) was low at 40%, HPV-ST remained the most effective strategy, but only reduced cancer risk by 16.2%; Pap had little health impact at this level of compliance, reducing cancer risk by only 5.4%. As compliance at all facilities rose to 85%, HPV-ST reduced cancer risk by 47.9%; HPV-VIA, HPV-Pap, and Pap reduced cancer risk by 42.0%, 40.7%, and 35.7% respectively.

In addition to remaining the most effective strategy across all sensitivity analyses, HPV-ST remained the most efficient strategy as well. HPV-ST remained the least costly and most effective

For peer review only - http://bmjopen.bmj.com/site/about/guidelines.xhtml

strategy with a stable ICER of US$320 per YLS when 1) Pap test performance (as a primary screening test) improved; 2) VIA and Pap triage test performance improved; and 3) colposcopy was assumed to be perfect. Despite slight fluctuation in the ICER, HPV-ST also remained the least costly and most effective strategy as 1) screening coverage varied from 50% to 80%; 2) visit compliance varied from 40% to 85% per visit; 3) the screen-and-treat cryotherapy cure rate was reduced to 75%; 4) the treatment cure rate following colposcopy was reduced to 85%; 5) only payer costs were considered, excluding women's time and transportation costs; 6) the direct medical cost of HPV self-collection was varied from 75% to 125% of the base case; 7) the direct medical cost of cryotherapy was increased to 170% of the base case; 8) the direct medical cost of colposcopy was reduced to 35% of the base case; 9) programmatic costs associated with HPV-based screening were varied from 50% to 150% of the base case; 10) women's time and transportation costs were reduced to 50% of the base case; and 11) the costs of cancer treatment ranged from including only direct medical costs to 150% of the base case (**Figure 5**). Among variables considered, compliance per visit appears to have the greatest impact on the ICER for HPV-ST, with 40% compliance yielding an ICER of US$580 per YLS and 85% compliance yielding an ICER of US$190 per YLS. Even when visit compliance is low, HPV-ST would be considered very cost-effective.

When only payer costs were considered (i.e., women's time and transportation costs were excluded), the total lifetime cost per woman was lower for all strategies, and Pap every 3 years was slightly less costly (although still slightly less effective) than HPV-Pap every 5 years. HPV-ST remained the most effective and efficient strategy, with an ICER of US$270 per YLS (**Appendix**).

The only scenario in which Pap testing every 3 years was the least costly strategy occurred when the direct medical cost of Pap testing was US$3 (base case: US$7.26), a value commonly cited for the cost of Pap in Nicaragua, though the source of this estimate is unknown. However, Pap remained the least effective strategy, and HPV-ST had a lower cost-effectiveness ratio, maintaining an ICER of US$320 per YLS (**Appendix**).

For peer review only

For peer review only - http://bmjopen.bmj.com/site/about/guidelines.xhtml

Discussion

Using implementation data from the Scale-Up project— which aims to facilitate institutionalization of HPV testing at the national level in Guatemala, Honduras, and Nicaragua— we estimated the long-term health impact and value of careHPV testing in Nicaragua's public health system. We found that screening algorithms consisting of HPV testing at 5-year intervals would be less costly and more effective than screening with Pap testing at 3-year intervals. Furthermore, HPV testing followed by treatment with cryotherapy for all eligible HPV-positive women would be less costly and more effective than HPV testing followed by triage testing with either VIA or Pap for HPV-positive women. A screen-and-treat HPV program would be a very cost-effective intervention in Nicaragua, with an ICER of US$320 per YLS under base case assumptions. These findings were robust across sensitivity analyses. The comparatively large health benefits and efficiency of HPV-ST can largely be attributed to the relatively low number of visits to health care facilities and the high sensitivity of the careHPV test to detect both CIN2+ and oncogenic HPV infections with the potential to develop into precancer.

We found that screening coverage of the target population had a considerable impact on achievable reductions in cervical cancer risk, with HPV-ST yielding the greatest risk reduction. Due to proportional increases in both costs and health benefits, the ICER for HPV-ST remained stable as coverage increased from 50% to 80%. Compliance with recommended follow-up was a key driver of both achievable reductions in cancer risk and the ICER of HPV-ST. As the proportion of women who returned for each clinical encounter (relative to the previous visit) increased from 40% to 85%, the cancer benefit associated with HPV-ST rose from 16.2% to 47.9%; the ICER fell from US$580 per YLS to US$190 per YLS. Thus, improved efforts to successfully navigate women to recommended follow-up will enhance screening program effectiveness and efficiency.

For peer review only - http://bmjopen.bmj.com/site/about/guidelines.xhtml

While substantially reducing the cost of Pap testing to US$3 (less than half of the base case value) made Pap the strategy with the lowest per-woman lifetime costs, the confluence of low test sensitivity and the high number of health facility visits needed to complete screening, diagnostic follow-up, and treatment made Pap the least effective strategy since women are lost to follow-up with each additional required visit. Even in this low-cost Pap scenario, HPV-ST remained the most effective and cost-effective strategy.

There are several limitations to this analysis. We did not consider alternative screening intervals or ages for each strategy, but rather restricted the analysis to the ages and intervals currently under consideration by the Nicaraguan Ministry of Health. The screening algorithms, as modeled, reflect the prototypical structure of a screening episode and the type of facility at which visits usually take place, but do not capture variation due to geography or health facility capacity. Furthermore, the modeled screening algorithms do not entirely reflect the complex downstream follow-up of screen-positive women that is embodied in the Ministry of Health's screening guidelines. In simplifying the downstream follow-up for modeled strategies, we may have underestimated the costs and overestimated the benefits relative to the national guidelines, which call for additional follow-up prior to treatment. However, the modeled strategies would likely bias the analysis in favor of Pap and HPV triage strategies.

While we adopted a micro-costing approach to leverage data from the START-UP and Scale-Up projects in Nicaragua, there remain limitations to our cost estimates. First, individual-level data for each woman were not available; thus, our estimates represent average costs in the project populations. Second, we did not have information on the costs associated with HPV self-collection in community settings, where most self-collection takes place. Instead, we assumed all self-collection took place at the clinic. Compared to clinic-based efforts, community-based self-collection may be associated with lower costs for women's time and travel, and higher direct medical and programmatic costs due to outreach worker involvement in facilitating screening and delivering results. Third, our estimates of programmatic

For peer review only - http://bmjopen.bmj.com/site/about/guidelines.xhtml

costs were restricted to training sessions, and we did not have information on the costs of social mobilization and outreach, patient navigation and support, or infrastructural improvements that would be required to successfully scale-up a screening program. Fourth, we valued women's time based on the minimum wage in Nicaragua. This may be a conservative estimate if most women attending screening are formally employed; conversely, it may overestimate the societal value of women's time spent working in the informal sector or at home. Finally, we extrapolated the cost of cancer treatment using data from El Salvador [29]. Despite these limitations, extensive sensitivity analyses on cost components indicate that HPV-ST is robustly the most efficient strategy.

As implementation of HPV testing continues, particularly without triage testing, the health system's capacity to provide cryotherapy will likely need to increase. While the use of triage testing (either with Pap or HPV) reduces the number of cryotherapy procedures performed, we found that the lower sensitivity of triage testing (resulting in more false negatives) led to a decline in health benefits as fewer women with persistent HPV infection and precancer received treatment. The cost savings associated with fewer cryotherapy procedures were outweighed by increased costs of additional follow-up and cancer treatment in triage-negative women. A sensitivity analysis on the cost of cryotherapy revealed that HPV-ST remained the dominant strategy even when costs increased to 170% of the base case. However, we did not explicitly consider the costs of increasing access to cryotherapy machines or the implications of gas stock-outs, which have been identified as barriers in some low- and middle-income countries [40]. New ablative technologies currently undergoing testing are smaller, portable, and do not require gas. Thermal-coagulation has been used in United Kingdom for more than 30 years, and now it is being used in several low- and middle-income countries, including as part of a "screen-and-treat" program in Malawi [41], and is currently undergoing testing in Latin America. If newer technologies demonstrate cure rates similar to cryotherapy, the cost-effectiveness of screen-and-treat algorithms may improve along with access to treatment.

For peer review only

For peer review only - http://bmjopen.bmj.com/site/about/guidelines.xhtml

359 In summary, using data from the Scale-Up implementation project in Nicaragua, we found that
360 HPV testing followed by cryotherapy for eligible HPV-positive women (a “screen-and-treat” approach)
361 was a very cost-effective intervention in Nicaragua. As the HPV-ST algorithm was not implemented in
362 Phase 2 of the Scale-Up project, compliance and cost estimates may need to be further honed to reflect
363 improvements in capacity for cryotherapy if HPV-ST is implemented going forward. Still, extensive
364 sensitivity analyses indicate the robustness of findings. An HPV-based screening algorithm involving a
365 similar “screen-and-treat” approach was recently found to be good value for public health dollars in El
366 Salvador [29], where national scale-up is underway. It is important to note that a favorable cost-
367 effectiveness profile does not guarantee that HPV-ST will be affordable or feasible in a lower-middle
368 income country like Nicaragua. Both the cost-effectiveness ratio, budgetary impact, and health systems
369 infrastructure need to be favorable for screening programs to be sustainable. We present these findings
370 to inform evidence-based decision making around national screening guidelines, program design and
371 implementation, and budgeting for infrastructural improvements and procurement of HPV tests in
372 Nicaragua.

For peer review only

References

1. Ferlay J, Soerjomataram I, Ervik M, et al. *GLOBOCAN 2012 v1.0, Cancer Incidence and Mortality Worldwide: IARC CancerBase No. 11 [Internet]*, 2013, International Agency for Research on Cancer: Lyon, France.
2. Walboomers JM, Jacobs MV, Manos MM, et al. Human papillomavirus is a necessary cause of invasive cervical cancer worldwide. *J Pathol* 1999;189(1):12-19.
3. Rodriguez AC, Schiffman M, Herrero R, et al. Longitudinal study of human papillomavirus persistence and cervical intraepithelial neoplasia grade 2/3: critical role of duration of infection. *J Natl Cancer Inst* 2010;102(5):315-324.
4. Kitchener HC, Castle PE, and Cox JT. Chapter 7: Achievements and limitations of cervical cytology screening. *Vaccine* 2006;24 Suppl 3:S3/63-70.
5. Denny L, Quinn M, and Sankaranarayanan R. Chapter 8: Screening for cervical cancer in developing countries. *Vaccine* 2006;24 Suppl 3:S3/71-77.
6. Institut Catala Oncologia. *HPV Information Centre*, 2014.
7. Vastbinder MB, Castillo C, and Bekkers RL. Barriers to follow-up of abnormal Papanicolaou smears among women in Leon, Nicaragua. *Trop Doct* 2010;40(1):22-26.
8. Khan MJ, Castle PE, Lorincz AT, et al. The elevated 10-year risk of cervical precancer and cancer in women with human papillomavirus (HPV) type 16 or 18 and the possible utility of type-specific HPV testing in clinical practice. *J Natl Cancer Inst* 2005;97(14):1072-1079.
9. World Health Organization. *WHO Guidelines for Screening and Treatment of Precancerous Lesions for Cervical Cancer Prevention.* World Health Organization, 2013: Geneva.
10. Arrossi S, Thouyaret L, Herrero R, et al. Effect of self-collection of HPV DNA offered by community health workers at home visits on uptake of screening for cervical cancer (the EMA study): a population-based cluster-randomised trial. *Lancet Glob Health* 2015;3(2):e85-94.
11. Moses E, Pedersen HN, Mitchell SM, et al. Uptake of community-based, self-collected HPV testing vs. visual inspection with acetic acid for cervical cancer screening in Kampala, Uganda: preliminary results of a randomised controlled trial. *Trop Med Int Health* 2015;20(10):1355-1367.
12. Lazcano-Ponce E, Lorincz AT, Cruz-Valdez A, et al. Self-collection of vaginal specimens for human papillomavirus testing in cervical cancer prevention (MARCH): a community-based randomised controlled trial. *Lancet* 2011;378(9806):1868-1873.
13. Racey CS, Withrow DR, and Gesink D. Self-collected HPV testing improves participation in cervical cancer screening: a systematic review and meta-analysis. *Can J Public Health* 2013;104(2):e159-166.
14. Jeronimo J, Bansil P, Lim J, et al. A multicountry evaluation of careHPV testing, visual inspection with acetic acid, and papanicolaou testing for the detection of cervical cancer. *Int J Gynecol Cancer* 2014;24(3):576-585.
15. Qiao YL, Sellors JW, Eder PS, et al. A new HPV-DNA test for cervical-cancer screening in developing regions: a cross-sectional study of clinical accuracy in rural China. *Lancet Oncol* 2008;9(10):929-936.
16. Jeronimo J, Holme F, Slavkovsky R, et al. Implementation of HPV testing in Latin America. *J Clin Virol* 2016;76 Suppl 1:S69-73.
17. Campos NG, Tsu, V., Jeronimo, J., Mvundura, M., Lee, K., Kim, J.J. When and how often to screen for cervical cancer in three low- and middle-income countries: A cost-effectiveness analysis. *Papillomavirus Research* 2015.

For peer review only - http://bmjopen.bmj.com/site/about/guidelines.xhtml

18. Campos NG, Burger EA, Sy S, et al. An updated natural history model of cervical cancer: derivation of model parameters. *Am J Epidemiol* 2014;180(5):545-555.
19. World Health Organization. *Macroeconomics and health: Investing in health for economic development: Report of the Commission on Macroeconomics and Health.* World Health Organization, 2001: Geneva.
20. Tan-Torres Edejer T, Baltussen, R., Adam, T., Hutubessy, R., Acharya, A., Evans, D.B., Murray, C.J.L., ed. *Making Choices in Health: WHO Guide to Cost-Effectiveness Analysis*. 2003, World Health Organization: Geneva, Switzerland.
21. Jamison DT, Breman, J.G., Measham, A.R., Alleyne, G., Claeson, M., Evans, D.B., Jha, P., Mills, A., Musgrove, P., ed. *Disease Control Priorities in Developing Countries*. Second edition ed. 2006, Oxford University Press and The World Bank: Washington, D.C.
22. Munoz N, Mendez F, Posso H, et al. Incidence, duration, and determinants of cervical human papillomavirus infection in a cohort of Colombian women with normal cytological results. *J Infect Dis* 2004;190(12):2077-2087.
23. Herrero R, Hildesheim A, Rodriguez AC, et al. Rationale and design of a community-based double-blind randomized clinical trial of an HPV 16 and 18 vaccine in Guanacaste, Costa Rica. *Vaccine* 2008;26(37):4795-4808.
24. Surveillance Epidemiology, and End Results (SEER) Program. *SEER*Stat Database: Incidence - SEER 18 Regs Research Data + Hurricane Katrina Impacted Louisiana Cases Nov 2011 Sub (1973-2009 varying) – Linked to County Attributes – Total U.S., 1969-2010 Counties*.
25. Sankaranarayanan R, Swaminathan R, Brenner H, et al. Cancer survival in Africa, Asia, and Central America: a population-based study. *Lancet Oncol* 2010;11(2):165-173.
26. Stinnett AA and Paltiel AD. Estimating CE ratios under second-order uncertainty: the mean ratio versus the ratio of means. *Med Decis Making* 1997;17(4):483-489.
27. Mayrand MH, Duarte-Franco E, Rodrigues I, et al. Human papillomavirus DNA versus Papanicolaou screening tests for cervical cancer. *N Engl J Med* 2007;357(16):1579-1588.
28. Ronco G, Dillner J, Elfstrom KM, et al. Efficacy of HPV-based screening for prevention of invasive cervical cancer: follow-up of four European randomised controlled trials. *Lancet* 2014;383(9916):524-532.
29. Campos NG, Maza M, Alfaro K, et al. The comparative and cost-effectiveness of HPV-based cervical cancer screening algorithms in El Salvador. *Int J Cancer* 2015.
30. Chirenje ZM, Rusakaniko S, Kirumbi L, et al. Situation analysis for cervical cancer diagnosis and treatment in east, central and southern African countries. *Bull World Health Organ* 2001;79(2):127-132.
31. Cremer M, Bullard K, Maza M, et al. Cytology versus visual inspection with acetic acid among women treated previously with cryotherapy in a low-resource setting. *Int J Gynaecol Obstet* 2010;111(3):249-252.
32. Sauvaget C, Muwonge R, and Sankaranarayanan R. Meta-analysis of the effectiveness of cryotherapy in the treatment of cervical intraepithelial neoplasia. *Int J Gynaecol Obstet* 2013;120(3):218-223.
33. Bigoni J, Gundar M, Tebeu PM, et al. Cervical cancer screening in sub-Saharan Africa: a randomized trial of VIA versus cytology for triage of HPV-positive women. *Int J Cancer* 2015;137(1):127-134.
34. Catarino R, Vassilakos P, Scaringella S, et al. Smartphone Use for Cervical Cancer Screening in Low-Resource Countries: A Pilot Study Conducted in Madagascar. *PLoS One* 2015;10(7):e0134309.

For peer review only - http://bmjopen.bmj.com/site/about/guidelines.xhtml

35. Pan QJ, Hu SY, Guo HQ, et al. Liquid-based cytology and human papillomavirus testing: a pooled analysis using the data from 13 population-based cervical cancer screening studies from China. *Gynecol Oncol* 2014;133(2):172-179.
36. Qiao YL, Jeronimo J, Zhao FH, et al. Lower cost strategies for triage of human papillomavirus DNA-positive women. *Int J Cancer* 2014;134(12):2891-2901.
37. Tebeu PM, Fokom-Domgue J, Crofts V, et al. Effectiveness of a two-stage strategy with HPV testing followed by visual inspection with acetic acid for cervical cancer screening in a low-income setting. *Int J Cancer* 2015;136(6):E743-750.
38. Richardson LA, El-Zein M, Ramanakumar AV, et al. HPV DNA testing with cytology triage in cervical cancer screening: Influence of revealing HPV infection status. *Cancer Cytopathol* 2015;123(12):745-754.
39. *World Development Indicators*, 2016, World Bank.
40. Msyamboza KP, Phiri T, Sichali W, et al. Cervical cancer screening uptake and challenges in Malawi from 2011 to 2015: retrospective cohort study. *BMC Public Health* 2016;16(1):806.
41. Campbell C, Kafwafwa S, Brown H, et al. Use of thermo-coagulation as an alternative treatment modality in a 'screen and treat' programme of cervical screening in rural Malawi. *Int J Cancer* 2016.
42. Ronco G, Dillner J, Elfstrom KM, et al. Efficacy of HPV-based screening for prevention of invasive cervical cancer: follow-up of four European randomised controlled trials. *Lancet* 2014;383(9916):524-532.
43. Starks D, Arriba LN, Enerson CL, et al. Mexican Cervical Cancer Screening Study II: 6-month and 2-year follow-up of HR-HPV women treated with cryotherapy in a low-resource setting. *J Low Genit Tract Dis* 2014;18(4):333-337.
44. McClung EC and Blumenthal PD. Efficacy, safety, acceptability and affordability of cryotherapy: a review of current literature. *Minerva Ginecol* 2012;64(2):149-171.

For peer review only - http://bmjopen.bmj.com/site/about/guidelines.xhtml

Table 1. Baseline values and ranges for model variables.[a]

Test/Parameter	Base Case:	Sensitivity Analysis:
Screening/triage test performance (sensitivity/specificity to detect CIN2+)		
Pap (primary) **[14, 27, 42]**	0.41/0.94	0.70/0.95
careHPV (primary), provider-collection of cervical samples **[14]**	0.78/0.89	--
careHPV (primary), provider-collection of vaginal samples **[14]**	0.67/0.86	--
VIA (triage of HPV+) **[14, 33, 34, 36, 37]**	0.60/0.75	0.40/0.85<br>0.70/0.65
Pap (triage of HPV+) **[14, 33, 35, 38]**	0.85/0.55	0.40/0.85<br>0.90/0.50
Colposcopy performance (sensitivity/specificity to detect CIN1+) **[14, 17]**[b]		
Colposcopy (ASCUS+ women)	0.95/0.68	1.0/1.0
Colposcopy (HPV+ women ineligible for cryotherapy)	Upon ruling out cancer, all referred to treatment	0.95/0.68, with <CIN1 sent back to routine screening intervals
Colposcopy (HPV+/VIA+ women ineligible for ST cryotherapy)	Upon ruling out cancer, all referred to treatment	0.95/0.68, with <CIN1 sent back to routine screening intervals
Colposcopy (HPV+/Pap+ women)	Upon ruling out cancer, all referred to treatment	0.95/0.68, with <CIN1 sent back to routine screening intervals
Coverage and compliance[c]		
Access to routine screening, % of the target population **[6]**	70%	50% - 80%
Visit compliance, screening facility[d]	85%	40% - 85%
Visit compliance, referral facility[d]	40%	40% - 85%
Treatment eligibility and efficacy		
Eligibility for screen-and-treat cryotherapy **[29]**	≤CIN1: 100%<br>CIN2: 85%<br>CIN3: 75%	--
Screen-and-treat cryotherapy cure rate (for HPV+ or HPV+/VIA+) **[30-32]**	92%	75%

For peer review only - http://bmjopen.bmj.com/site/about/guidelines.xhtml

For peer review only

Proportion of women maintaining an HPV infection following cryotherapy **[43]**	15%	--
Treatment cure rate following colposcopy (LEEP for CIN2+; else cryotherapy)**[30]**	96%	85%
Proportion of women maintaining an HPV infection following colposcopic diagnosis and treatment **[44]**	10%	--
Direct medical costs, screening and treatment of precancer (2015 US$)**[14]**[d]		
Pap test	7.26	3
careHPV test (provider-collection)	11.96	--
careHPV test (self-collection)	11.04	75% - 125% of base case
VIA triage test	4.19	--
Colposcopy/biopsy	19.91	6.91[e]
Cryotherapy	18.16	30.54[f]
LEEP	68.36	--
Women's time and transportation costs (2015 US$)[d]		
Transportation to screening facility (round-trip)	0.41	0% to 50% of base case
Transportation to referral facility (round trip)	2.81	0% to 50% of base case
Wait time, screening facility	0.48	0% to 50% of base case
Wait time, referral facility	1.75	0% to 50% of base case
Transport time, screening facility	0.82	0% to 50% of base case
Transport time, referral facility	3.10	0% to 50% of base case
Programmatic costs, training (2015 US$)[d]		
Health care personnel (careHPV)	0.09 per woman screened with careHPV	50% - 150% of base case
Laboratory technicians (careHPV)	0.04 per woman screened with careHPV	50% - 150% of base case
Outreach workers/auxiliary nurses (careHPV self-collection)	0.08 per woman screened with careHPV (self-collection)	50% - 150% of base case
Health care providers (VIA and cryotherapy)	1.51 per woman receiving VIA and/or cryotherapy	50% - 150% of base case
Cost of cancer treatment (2015 US$)**[29]**		
Local cancer		
- Direct medical	944	
- Direct non-medical[g]	197	
- Women's time[h]	346	

For peer review only - http://bmjopen.bmj.com/site/about/guidelines.xhtml

Total	1,486	944 - 2,2920 (direct medical only; 150% of base case)
Regional and distant cancer		
- Direct medical	918	
- Direct non-medical[g]	390	
- Women's time[h]	640	
Total	1,946	918 - 2,290 (direct medical only; 150% of base case)

[a] ASCUS+: atypical squamous cells of undetermined significance or higher; CIN 1+: cervical intraepithelial neoplasia grade 1 or higher; CIN2+: cervical intraepithelial neoplasia grade 2 or higher; HPV: human papillomavirus; LEEP: loop electrosurgical excision procedure; ST: screen-and-treat; US$: 2015 United States dollars; VIA: visual inspection with acetic acid.
[b] Test performance characteristics of colposcopy in the START-UP demonstration project were derived from the worst diagnosis of the local pathologist relative to the worst diagnosis by a quality control pathologist (gold standard); we applied the treatment threshold of CIN1+, although this was not the treatment threshold in START-UP. To derive test performance of colposcopy, we excluded histological classifications that were inadequate or with a histological classification other than negative, CIN1, CIN2, CIN3, or cancer. Because CIN1 is not a true underlying health state in the microsimulation model, performance of colposcopy in the model is based on the underlying health states of no lesion, HPV infection, CIN2, or CIN3. For a treatment threshold of CIN1, we weighted sensitivity of colposcopy for women with HPV based on the country-specific prevalence of CIN1 among women with HPV infections in the START-UP studies.
[c] Compliance is defined as the proportion of women who return for each clinical encounter, relative to the previous visit.
[d] Unpublished data from the Scale-Up Nicaragua project. Further details on costing data are provided in the Appendix.
[e] In sensitivity analysis, we considered the direct medical cost of colposcopy to be equivalent to the cost of colposcopy alone, without biopsy.
[f] In sensitivity analysis, we considered the direct medical cost of cryotherapy to include the upper bound of cryotherapy equipment costs (assuming the lowest number of women treated per year per facility, in Carazo, Chontales, or Chinandega) and the upper bound of cryotherapy supply costs (assuming the lowest number of women treated per gas tank in any facility in Carazo, Chontales, or Chinandega).
[g] Direct non-medical costs include transportation to a tertiary facility, temporary housing, and meals.
[h] Includes woman's time an support person's time.

For peer review only - http://bmjopen.bmj.com/site/about/guidelines.xhtml

Figure legends.

Figure 1. Pathways of care, by screening strategy. Each diagram indicates the flow of screening-eligible women (i.e., women aged 30 to 59 years) through each point of contact in a screening episode, conditional on visit compliance and test results, for a) Pap testing every 3 years (Pap), which requires 4 visits for screening, diagnosis, and treatment; b) HPV screen-and-treat every 5 years (HPV-ST), which requires 3 or more visits for screening and necessary treatment; c) HPV testing followed by visual inspection with acetic acid (VIA) triage of HPV-positive women every 5 years (HPV-VIA), which requires 3 or more visits for screening and necessary treatment; and d) HPV testing followed by Pap triage of HPV-positive women every 5 years (HPV-Pap), which requires 5 or more visits for screening and necessary treatment. CIN: cervical intraepithelial neoplasia; HPV: human papillomavirus; LEEP: loop electrosurgical excision procedure; VIA: visual inspection with acetic acid.

Figure 2. Cervical cancer screening cost per woman over duration of screening eligibility, by cost component: Pap testing (every 3 years) versus careHPV testing (every 5 years). Bars indicate the undiscounted cost (2015 US$) of screening with Pap testing (offered 10 times between ages 30 and 59 years) versus careHPV testing (offered 6 times between ages 30 and 59 years), by cost component. Only screening costs are shown; costs associated with recommended management following a positive screening test are not included. 6x: delivered 6 times over the course of screening eligible ages 30 to 59; 10x: delivered 10 times over the course of screening eligible ages.

Figure 3. Cost-effectiveness analysis: Base case. The graph displays the discounted lifetime costs (x-axis; in 2015 US$) and life expectancy (y-axis) associated with each screening strategy (Pap testing every 3 years; careHPV screen-and-treat every 5 years [HPV-ST]; careHPV every 5 years with visual inspection with acetic acid [VIA] triage of HPV-positive women [HPV-VIA]; and careHPV every 5 years with Pap triage of HPV-positive women [HPV-Pap]), under base case assumptions. The cost-effectiveness associated with a change from one strategy to a more costly alternative is represented by the difference

For peer review only - http://bmjopen.bmj.com/site/about/guidelines.xhtml

For peer review only

in cost divided by the difference in life expectancy associated with the two strategies. The curve indicates the strategies that are efficient because they are more effective and either 1) cost less; or 2) have a more attractive cost-effectiveness ratio than less effective options. The incremental cost-effectiveness ratio (ICER) is the reciprocal of the slope of the line connecting the two strategies under comparison. In the base case analysis, HPV-ST every 5 years was less costly and more effective than other screening strategies considered, and was thus a dominant strategy with an ICER of US$320 per year of life saved. HPV-Pap: HPV testing with Pap triage of HPV-positive women; HPV-ST: HPV screen-and-treat strategy; HPV-VIA: HPV testing with visual inspection with acetic acid (VIA) triage of HPV-positive women; yrs: years.

Figure 4. Reduction in lifetime risk of cervical cancer, by compliance level. Bars indicate the percent reduction in lifetime risk of cervical cancer for each screening strategy (Pap testing every 3 years; careHPV screen-and-treat every 5 years [HPV-ST]; careHPV every 5 years with visual inspection with acetic acid [VIA] triage of HPV-positive women [HPV-VIA]; and careHPV every 5 years with Pap triage of HPV-positive women [HPV-Pap]) as compliance per visit within a screening episode increases. Compliance is defined as the proportion of women who return for each clinical encounter, relative to the previous visit. Coverage of the target population is assumed to be 70%. While the base case analysis assumed 85% compliance for visits at screening facilities and 40% compliance for visits at referral facilities (for diagnosis and treatment), the graph displays cancer risk reduction assuming the specified compliance level at all visits, regardless of facility type.

Figure 5. Base case and sensitivity analysis: Incremental cost-effectiveness ratios, HPV screen-and-treat (HPV-ST) strategy. Incremental cost-effectiveness ratios (ICERs) are presented (x-axis, 2015 US$ per year of life saved) for the base case and sensitivity analyses (y-axis). The blue bars represent the range of the ICERs for HPV-ST every 5 years across the 50 input parameter sets, with the ICER of the mean costs divided by the mean effects demarcated by a black line. The dashed blue line indicates

For peer review only

Nicaragua's per capita gross domestic product (GDP), at US$2,090, assuming this is the threshold that designates interventions as "very cost-effective".

For peer review only

For peer review only - http://bmjopen.bmj.com/site/about/guidelines.xhtml

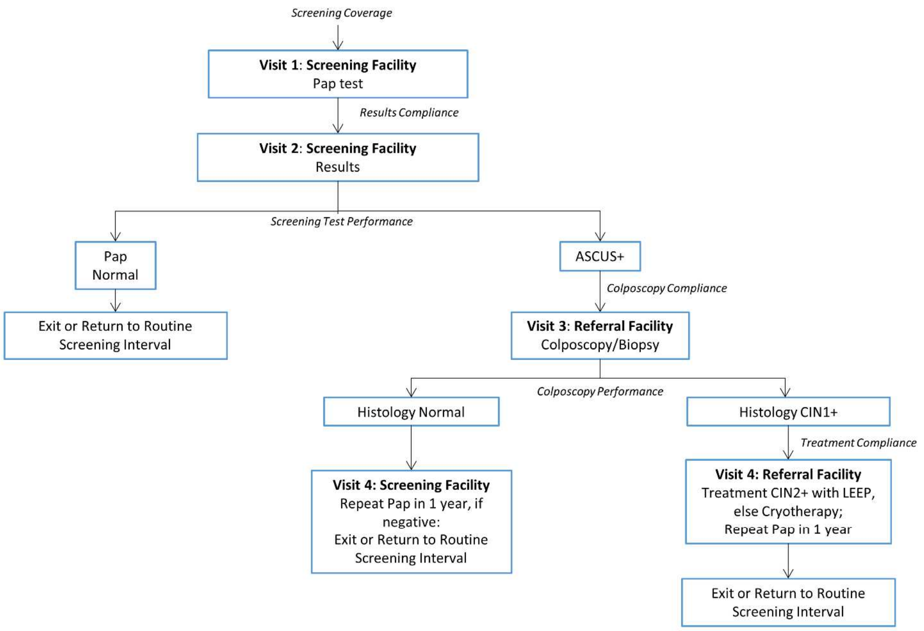

Figure 1A. Pathways of care, by screening strategy. Each diagram indicates the flow of screening-eligible women (i.e., women aged 30 to 59 years) through each point of contact in a screening episode, conditional on visit compliance and test results, for a) Pap testing every 3 years (Pap), which requires 4 visits for screening, diagnosis, and treatment; b) HPV screen-and-treat every 5 years (HPV-ST), which requires 3 or more visits for screening and necessary treatment; c) HPV testing followed by visual inspection with acetic acid (VIA) triage of HPV-positive women every 5 years (HPV-VIA), which requires 3 or more visits for screening and necessary treatment; and d) HPV testing followed by Pap triage of HPV-positive women every 5 years (HPV-Pap), which requires 5 or more visits for screening and necessary treatment. CIN: cervical intraepithelial neoplasia; HPV: human papillomavirus; LEEP: loop electrosurgical excision procedure; VIA: visual inspection with acetic acid.

254x190mm (300 x 300 DPI)

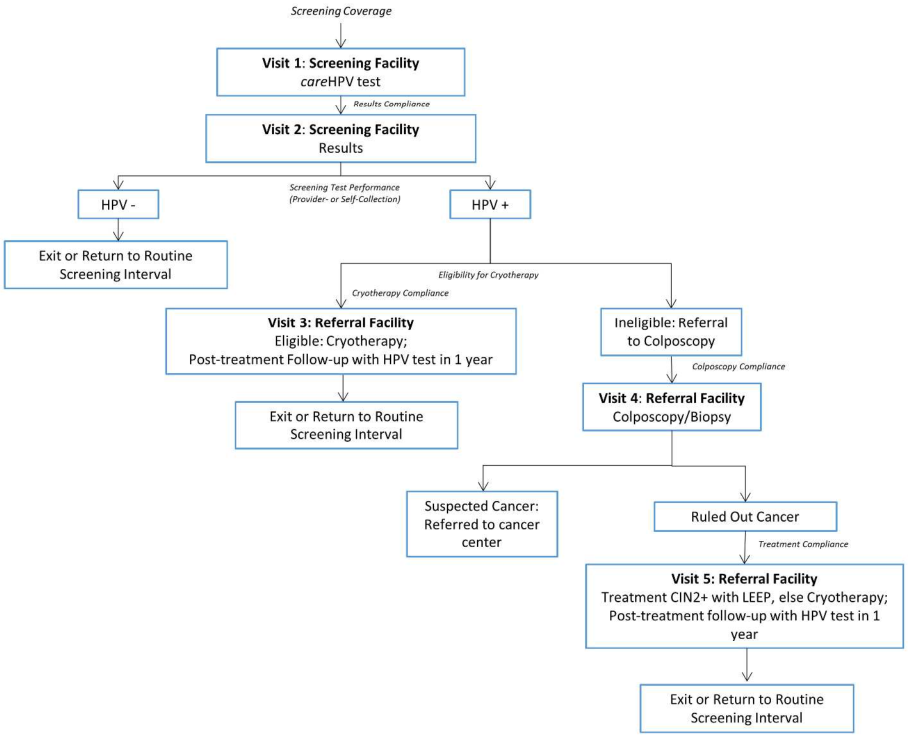

Figure 1B. Pathways of care, by screening strategy. Each diagram indicates the flow of screening-eligible women (i.e., women aged 30 to 59 years) through each point of contact in a screening episode, conditional on visit compliance and test results, for a) Pap testing every 3 years (Pap), which requires 4 visits for screening, diagnosis, and treatment; b) HPV screen-and-treat every 5 years (HPV-ST), which requires 3 or more visits for screening and necessary treatment; c) HPV testing followed by visual inspection with acetic acid (VIA) triage of HPV-positive women every 5 years (HPV-VIA), which requires 3 or more visits for screening and necessary treatment; and d) HPV testing followed by Pap triage of HPV-positive women every 5 years (HPV-Pap), which requires 5 or more visits for screening and necessary treatment. CIN: cervical intraepithelial neoplasia; HPV: human papillomavirus; LEEP: loop electrosurgical excision procedure; VIA: visual inspection with acetic acid.

254x190mm (300 x 300 DPI)

For peer review only - http://bmjopen.bmj.com/site/about/guidelines.xhtml

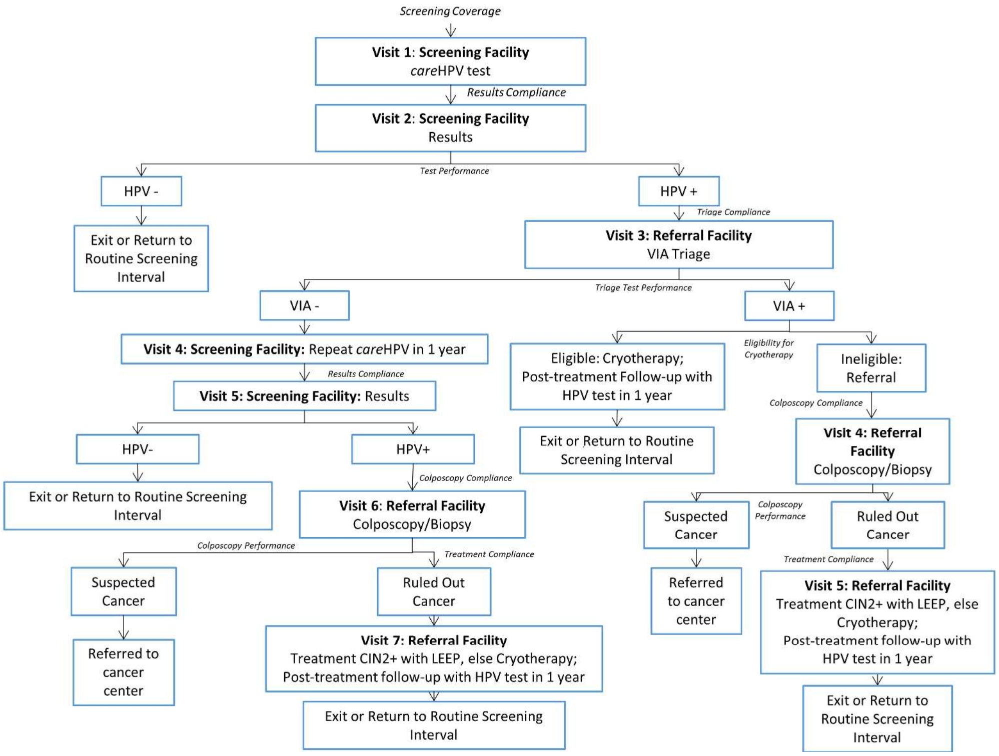

Figure 1C. Pathways of care, by screening strategy. Each diagram indicates the flow of screening-eligible women (i.e., women aged 30 to 59 years) through each point of contact in a screening episode, conditional on visit compliance and test results, for a) Pap testing every 3 years (Pap), which requires 4 visits for screening, diagnosis, and treatment; b) HPV screen-and-treat every 5 years (HPV-ST), which requires 3 or more visits for screening and necessary treatment; c) HPV testing followed by visual inspection with acetic acid (VIA) triage of HPV-positive women every 5 years (HPV-VIA), which requires 3 or more visits for screening and necessary treatment; and d) HPV testing followed by Pap triage of HPV-positive women every 5 years (HPV-Pap), which requires 5 or more visits for screening and necessary treatment. CIN: cervical intraepithelial neoplasia; HPV: human papillomavirus; LEEP: loop electrosurgical excision procedure; VIA: visual inspection with acetic acid.

254x190mm (300 x 300 DPI)

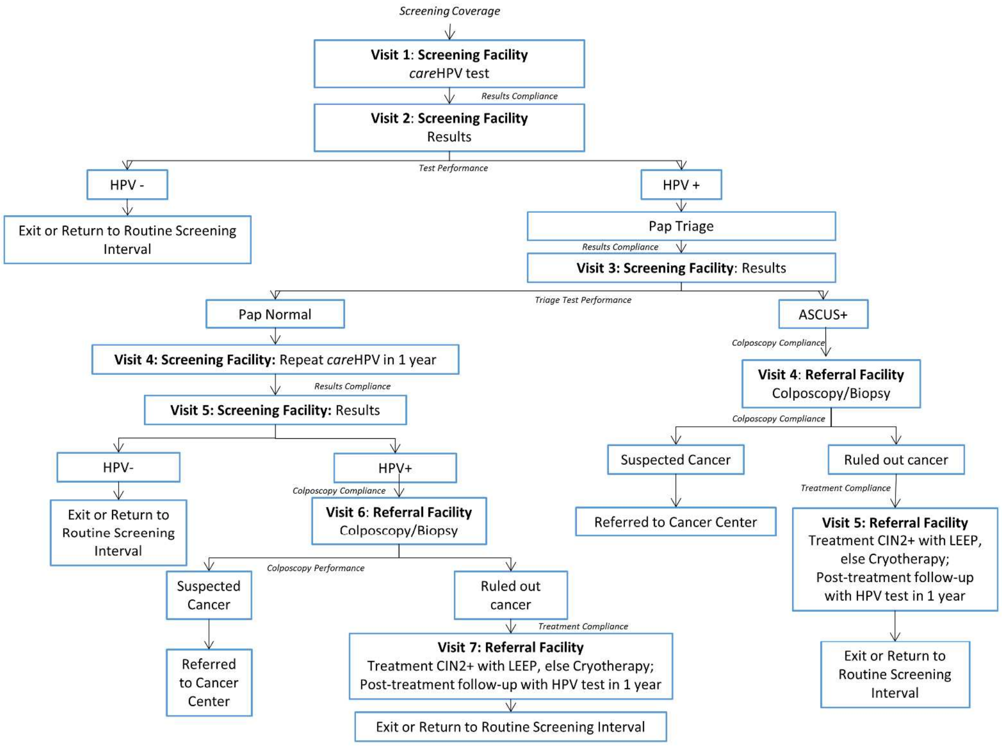

Figure 1D. Pathways of care, by screening strategy. Each diagram indicates the flow of screening-eligible women (i.e., women aged 30 to 59 years) through each point of contact in a screening episode, conditional on visit compliance and test results, for a) Pap testing every 3 years (Pap), which requires 4 visits for screening, diagnosis, and treatment; b) HPV screen-and-treat every 5 years (HPV-ST), which requires 3 or more visits for screening and necessary treatment; c) HPV testing followed by visual inspection with acetic acid (VIA) triage of HPV-positive women every 5 years (HPV-VIA), which requires 3 or more visits for screening and necessary treatment; and d) HPV testing followed by Pap triage of HPV-positive women every 5 years (HPV-Pap), which requires 5 or more visits for screening and necessary treatment. CIN: cervical intraepithelial neoplasia; HPV: human papillomavirus; LEEP: loop electrosurgical excision procedure; VIA: visual inspection with acetic acid.

254x190mm (300 x 300 DPI)

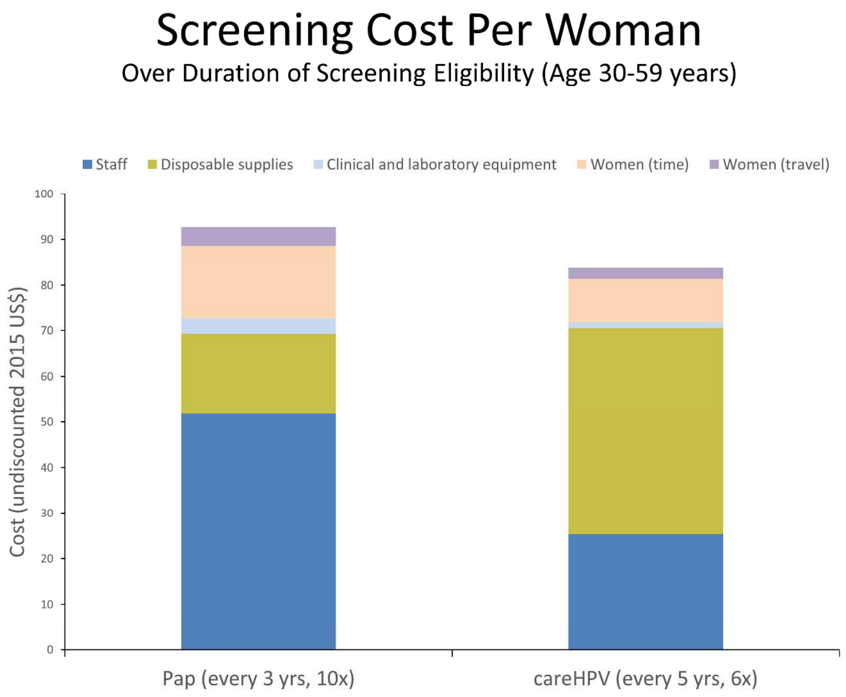

Figure 2. Cervical cancer screening cost per woman over duration of screening eligibility, by cost component: Pap testing (every 3 years) versus careHPV testing (every 5 years). Bars indicate the undiscounted cost (2015 US$) of screening with Pap testing (offered 10 times between ages 30 and 59 years) versus careHPV testing (offered 6 times between ages 30 and 59 years), by cost component. Only screening costs are shown; costs associated with recommended management following a positive screening test are not included. 6x: delivered 6 times over the course of screening eligible ages 30 to 59; 10x: delivered 10 times over the course of screening eligible ages.

254x190mm (300 x 300 DPI)

only

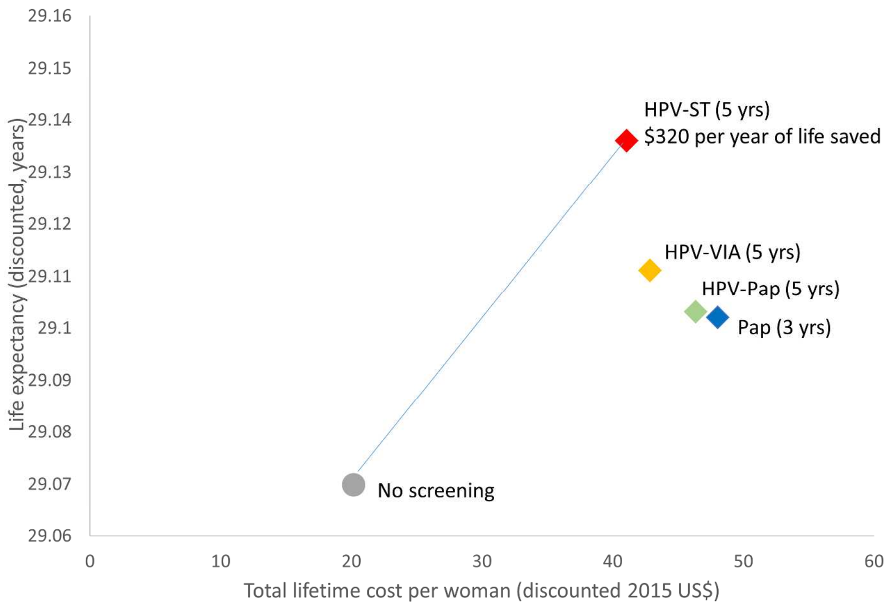

Figure 3. Cost-effectiveness analysis: Base case. The graph displays the discounted lifetime costs (x-axis; in 2015 US$) and life expectancy (y-axis) associated with each screening strategy (Pap testing every 3 years; careHPV screen-and-treat every 5 years [HPV-ST]; careHPV every 5 years with visual inspection with acetic acid [VIA] triage of HPV-positive women [HPV-VIA]; and careHPV every 5 years with Pap triage of HPV-positive women [HPV-Pap]), under base case assumptions. The cost-effectiveness associated with a change from one strategy to a more costly alternative is represented by the difference in cost divided by the difference in life expectancy associated with the two strategies. The curve indicates the strategies that are efficient because they are more effective and either 1) cost less; or 2) have a more attractive cost-effectiveness ratio than less effective options. The incremental cost-effectiveness ratio (ICER) is the reciprocal of the slope of the line connecting the two strategies under comparison. In the base case analysis, HPV-ST every 5 years was less costly and more effective than other screening strategies considered, and was thus a dominant strategy with an ICER of US$320 per year of life saved. HPV-Pap: HPV testing with Pap triage of HPV-positive women; HPV-ST: HPV screen-and-treat strategy; HPV-VIA: HPV testing with visual inspection with acetic acid (VIA) triage of HPV-positive women; yrs: years.

254x190mm (300 x 300 DPI)

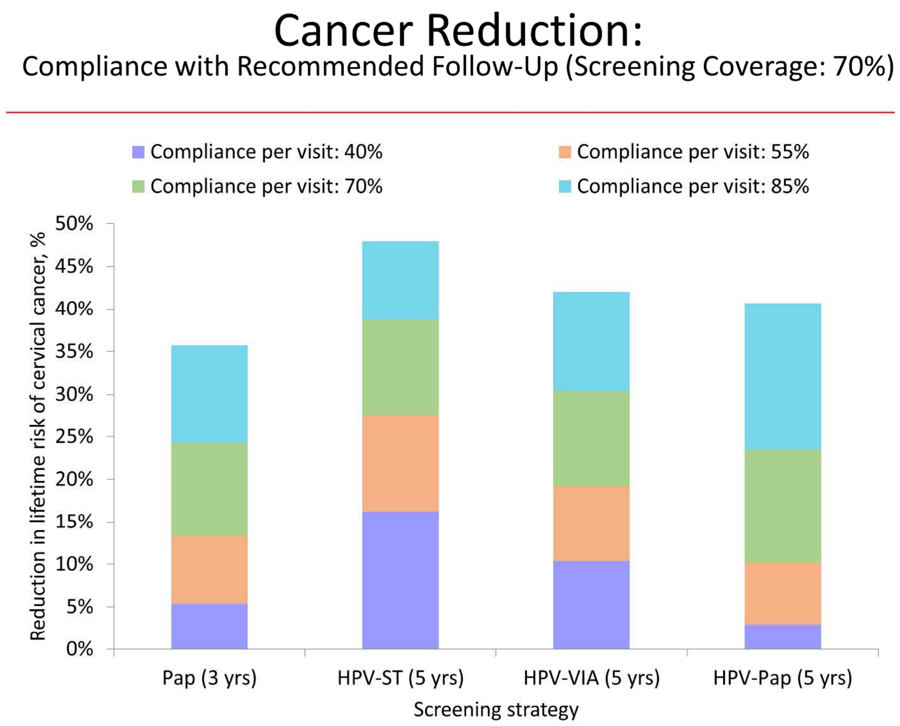

Figure 4. Reduction in lifetime risk of cervical cancer, by compliance level. Bars indicate the percent reduction in lifetime risk of cervical cancer for each screening strategy (Pap testing every 3 years; careHPV screen-and-treat every 5 years [HPV-ST]; careHPV every 5 years with visual inspection with acetic acid [VIA] triage of HPV-positive women [HPV-VIA]; and careHPV every 5 years with Pap triage of HPV-positive women [HPV-Pap]) as compliance per visit within a screening episode increases. Compliance is defined as the proportion of women who return for each clinical encounter, relative to the previous visit. Coverage of the target population is assumed to be 70%. While the base case analysis assumed 85% compliance for visits at screening facilities and 40% compliance for visits at referral facilities (for diagnosis and treatment), the graph displays cancer risk reduction assuming the specified compliance level at all visits, regardless of facility type.

254x190mm (300 x 300 DPI)

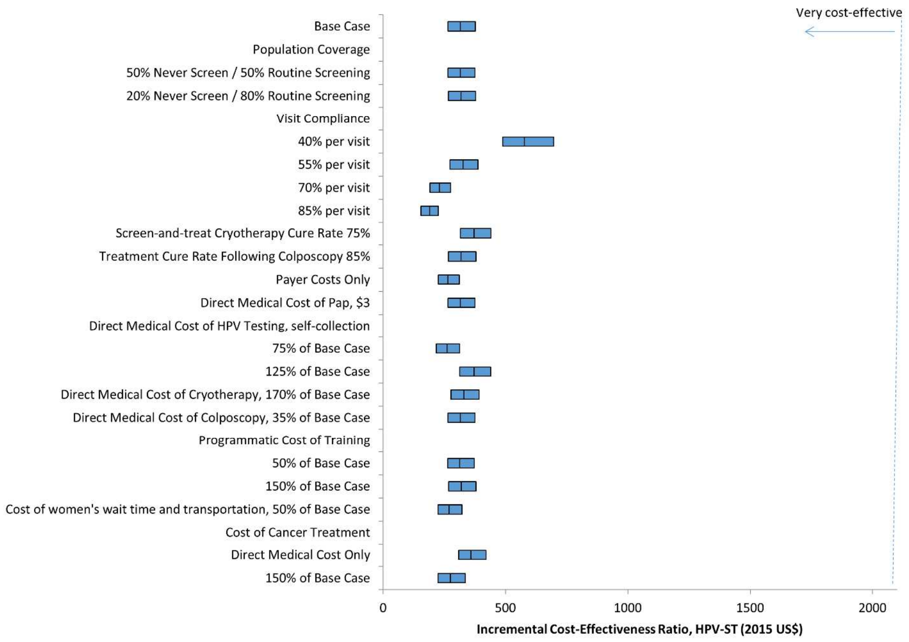

Figure 5. Base case and sensitivity analysis: Incremental cost-effectiveness ratios, HPV screen-and-treat (HPV-ST) strategy. Incremental cost-effectiveness ratios (ICERs) are presented (x-axis, 2015 US$ per year of life saved) for the base case and sensitivity analyses (y-axis). The blue bars represent the range of the ICERs for HPV-ST every 5 years across the 50 input parameter sets, with the ICER of the mean costs divided by the mean effects demarcated by a black line. The dashed blue line indicates Nicaragua’s per capita gross domestic product (GDP), at US$2,090, assuming this is the threshold that designates interventions as “very cost-effective”.

254x190mm (300 x 300 DPI)

only

Supplemental Appendix:
The Cost-Effectiveness of HPV-Based Screening in the Public Health System in Nicaragua

MODEL CALIBRATION

Overview of the calibration process

Details of model development for the U.S. and Nicaragua models, including initial parameterization and calibration, have been published elsewhere [1, 2]. Baseline HPV incidence rates, as a function of genotype and age, were derived from a published prospective study of sexually active women aged 15-85 years in Bogota, Colombia [3]. Because HPV incidence is not necessarily similar in Colombia and Nicaragua due to differences in sexual behavior, we considered age-specific HPV incidence and natural immunity following initial infection as candidate parameters for calibration. Type-specific data on CIN2 and CIN3 regression and progression are limited [4-9], so these highly uncertain parameters were also candidates for calibration. Time-dependent rates of HPV clearance and progression by genotype were informed by primary longitudinal data from the control arm of the Costa Rica Vaccine Trial [10], assuming that rates of HPV clearance and progression to precancer are similar across populations and vary only by time since infection and HPV genotype.

To calibrate the model, we selected empirical epidemiologic data from Nicaragua, including age-specific prevalence of high-risk HPV and age-specific cancer incidence (see below for further detail). We then set plausible search ranges around baseline model input values for age- and type-specific HPV incidence; natural immunity following initial infection; and progression and regression of CIN. We performed repeated model simulations in the absence of any preventive intervention (i.e., natural history simulations). For each simulation, we randomly selected a single value for each of the uncertain parameters from the identified search range, creating a unique vector of parameter values (i.e., parameter "set"). From over 1,475,000 repeated samplings, we identified the parameter sets with the highest correspondence to the empirical calibration target data from Nicaragua by calculating and aggregating the log-likelihood of model-projected outcomes. We used the 50 parameter sets with the highest likelihood scores (i.e., best overall fit to the empirical data from Nicaragua) for analysis to capture uncertainty in the model parameters as a form of probabilistic sensitivity analysis. We report results as a mean of outcomes across these top 50 parameter sets; incremental cost-effectiveness ratios are reported as the ratio of the mean costs divided by the mean effects of one strategy versus another across sets.

Calibration targets

We assessed model fit by comparing projected model outcomes of age-specific prevalence of high-risk HPV and age-specific cancer incidence relative to empirical data from Nicaragua. The scoring algorithm included age-specific prevalence of high-risk HPV and age-specific cervical cancer incidence.

Age-specific prevalence of high-risk HPV was drawn from the Screening Technologies to Advance Rapid Testing for Cervical Cancer Prevention–Utility and Program Planning (START-UP) project data on *care*HPV positivity using a cut-off ratio cut-point of 0.5 relative light units (**Table A.1**). For each age group, we derived a 95% binomial confidence interval around the point prevalence, which comprised the calibration target. The likelihood function for each age group was assumed to follow a binomial distribution.

Age-specific cancer incidence was drawn from Globocan due to the lack of a local cancer registry [11] (**Table A.2**). The likelihood function for each age group was assumed to follow a normal distribution.

For peer review only - http://bmjopen.bmj.com/site/about/guidelines.xhtml

Composite goodness-of-fit scores for each input parameter set were generated by summing the log likelihood of each model outcome (i.e., age-specific HPV prevalence, age-specific cancer incidence). The 50 input parameter sets with the highest goodness-of-fit scores yielded the model outputs that were simultaneously closest to all calibration targets, and these were selected for analysis. **Figures A.1** and **A.2** display model fit to epidemiologic data on age-specific prevalence of high-risk HPV and age-specific cancer incidence.

COST DATA

Direct Medical Costs: Screening, Diagnosis, and Treatment of Precancerous Lesions

The direct medical costs of screening, diagnosis, and treatment of precancerous lesions were drawn from the START-UP demonstration study in Masaya department and from the Scale-Up Nicaragua Project in the departments of Carazo, Chontales, and Chinandega. Direct medical costs included clinical staff time, clinical supplies, drugs, clinical equipment, laboratory staff time, laboratory supplies, and laboratory equipment. Costs from the START-UP study were collected in 2010 local currency units and inflated to year 2015 levels using Nicaragua GDP deflators and then converted to US$ using the official exchange rate [12]; the exception was for equipment, which was generally procured in the United States and was inflated to year 2015 levels using US GDP deflators. Costs from the Scale-Up Nicaragua project (including equipment) were collected in 2015 local currency units and converted to US$ using the official exchange rate [12].

Costs are reported in **Table A.3** (screening procedures) and **Table A.4** (precancer diagnostic and treatment procedures).

Women's Time and Transportation Costs

To account for the opportunity cost of women's time spent traveling to, waiting for, or receiving care, we valued women's time using Nicaragua's 2013 monthly minimum wage [13], converted to 2015 US$. While the minimum wage may be a conservative estimate for valuation of women's time if they are formally employed, it can serve as a proxy for the societal value of women's time spent working in the informal sector or at home. We converted this to an hourly wage rate assuming 171 hours of work per month (40 hours per week).

Estimates for time spent traveling to, waiting for, and receiving care were dependent upon the facility level where care was assumed to take place (**Table A.5**). While screening and delivery of primary screening results were assumed to take place at basic primary health facilities, we assumed cryotherapy and LEEP treatment were only provided at referral facilities. Estimates of women's time spent receiving a procedure were based on site-specific data from the START-UP demonstration projects, with staff time spent on the procedure (excluding preparation and registration time, which we assumed were built into patient waiting time) used as a proxy for women's procedure time. Estimates of women's wait time for screening and delivery of primary screening results was based on the average wait time for screening at facilities in the Chinandega department; wait time for cryotherapy, colposcopy, and LEEP was based on the average wait time at referral centers in the Chontales department. Round-trip transportation time was based on Scale-Up project staff estimates of the typical amount of time women spent traveling to screening or referral facilities, using a sample of 9 facilities the Carazo department and 10 facilities in the Chontales and Chinandega departments. We averaged across screening and referral facilities within each department to obtain a department average, and then averaged across departments to obtain

For peer review only - http://bmjopen.bmj.com/site/about/guidelines.xhtml

average round-trip transportation times based on facility level. Estimates of women's time and time costs are presented in **Table A.6.**

Women's transportation costs were based on Scale-Up project staff reports of the typical mode of transportation to a particular facility by women in the catchment area, using a sample of 9 facilities in Carazo and 10 facilities each in Chontales or Chinandega. We used the most commonly reported mode of transportation for each department (for screening facilities: walking for Carazo; walking for Chontales; bus for Chinandega; for referral facilities: bus in all three departments). The cost of a bus trip was based on typical bus fare for the distance to the health facility, as reported by Scale-Up project staff. We averaged the typical transportation costs across departments to obtain an estimate of women's round-trip transportation costs to and from a health facility, assuming the same mode of transport was used for the trip to and from the health facility. Women's transportation costs are presented in **Table A.7.**

Programmatic Costs

We included the programmatic cost of the following types of training sessions that were conducted during the Scale-Up project: 1) for health care personnel to offer careHPV screening and counsel women on results; 2) for laboratory technicians to run the careHPV assay; 3) for community outreach workers and auxiliary nurses to promote careHPV screening and offer self-collection of HPV samples; and 4) for health care providers to offer VIA and cryotherapy. We included the cost of trainer time and trainee time (valued using average monthly salaries converted to an hourly rate; volunteer outreach worker time was valued using the minimum wage), as well as the cost of supplies, materials, transportation, and refreshments. To derive an average health care personnel training cost per woman screened, we divided the total training cost for health care personnel by 100,000, corresponding to the number of women screened during the Scale-Up project. To derive an average laboratory technician training cost per woman screened, we divided the total training cost for laboratory technicians by 100,000. To derive an average community outreach worker training cost per woman screened, we divided the total training cost for outreach workers by 80,000, corresponding to the number of women who participated in self-collection of HPV samples. To derive an average health care provider training cost per woman receiving VIA and cryotherapy, we divided the total VIA/cryotherapy training cost for health care providers by 16,000, corresponding to the approximate number of women who tested HPV-positive in Scale-Up and would thus require visual assessment (to determine eligibility for cryotherapy) and, if eligible, cryotherapy.

All women screened with HPV testing incurred the per-woman training costs for health care personnel and laboratory technicians. Only women screened with HPV self-collection incurred the per-woman cost of training outreach workers to offer self-collection (due to lack of data on self-collection in a community setting, in the base case analysis we assumed self-collection took place at the clinic; however, we assumed outreach workers would be involved in mobilizing and educating women about self-collection, and conservatively included the cost of training so as to represent a known programmatic cost as self-collection efforts shift to also include community settings). Only women who received either VIA (as triage) or screen-and-treat cryotherapy incurred the per-woman cost of training health providers to perform VIA and cryotherapy. Training costs are presented in **Table A.8.**

Cost of Cancer Care by Stage

Costs associated with cancer care by stage (Local versus Regional or Distant), including direct medical costs, direct non-medical costs (i.e., transport to a cancer center, and meals and temporary housing during the course of treatment), and patient and support person time were derived from a previous

For peer review only - http://bmjopen.bmj.com/site/about/guidelines.xhtml

analysis in El Salvador [14]. Based on expert opinion in El Salvador, we assumed 50% of local cancer patients presented with FIGO stage 1a, requiring radical hysterectomy, and 50% of local cancer patients presented with FIGO stage 1b/2a, requiring cobalt therapy followed by 5 chemotherapy sessions followed by brachytherapy. We assumed all women presenting with FIGO stage 2b or higher received cobalt therapy followed by 5 chemotherapy sessions followed by brachytherapy.

To adjust the direct medical costs of cancer treatment in El Salvador to the setting of Nicaragua, we multiplied by the ratio of WHO-CHOICE inpatient bed-day costs at a teaching hospital in Nicaragua relative to El Salvador. We assumed each patient and a support person spent the same amount of time traveling, waiting for, and receiving care as in El Salvador, and valued this time at the 2013 minimum wage rate in Nicaragua (inflated and converted to 2015 US$). To adjust temporary housing and transportation costs to the setting of Nicaragua, we multiplied the Salvadoran costs by the ratio of GDP per capita in Nicaragua relative to El Salvador. Costs are reported in **Table A.9**.

SUPPLEMENTARY RESULTS

Additional results described in the main manuscript are presented in **Figures A3** to **A5.**

For peer review only - http://bmjopen.bmj.com/site/about/guidelines.xhtml

For peer review only

Table A.1. Age-specific prevalence of high-risk HPV, Nicaragua [15].[a]

Age group	Number of women	Number of women with high-risk HPV	Prevalence (95% CI)
30 – 34 years	1,693	310	0.18 (0.17, 0.20)
35 – 39 years	1,141	184	0.16 (0.14, 0.18)
40 – 44 years	933	125	0.13 (0.11, 0.16)
45 – 49 years	878	121	0.14 (0.12, 0.16)

[a] HPV positivity was based on a cut-off of 0.5 relative light units.

Table A.2. Age-specific cervical cancer incidence, Nicaragua (GLOBOCAN 2012) [11].

Age group	Cases	Rate per 100,000 women (95% CI)
40 – 44 years	123	78.7 (64.8, 92.6)
45 – 49 years	112	85.4 (69.6, 101.2)
50 – 54 years	102	88.4 (71.2, 105.6)
55 – 59 years	85	88.1 (69.4, 106.8)
60 – 64 years	51	84.0 (61.0, 107.1)
65 – 69 years	37	80.8 (54.8, 106.8)
70 – 74 years	30	74.6 (47.9, 101.3)
≥75 years	45	70.3 (49.8, 90.8)

For peer review only

For peer review only - http://bmjopen.bmj.com/site/about/guidelines.xhtml

Table A.3. Screening: Direct medical costs per procedure (2015 US$).[a]

Cost category	HPV test (provider-collection)[b]	HPV test (self-collection)[b]	Cytology	VIA triage	Source
Clinical staff	3.81	2.88	3.81	3.81	START-UP
Clinical supplies	6.72	6.72	0.82	0.36	Scale-Up (HPV);[c] START-UP (Cytology/VIA)
Clinical equipment	0.01	0.01	0.01	0.02	
Lab staff	0.41	0.41	1.37	0.00	Scale-Up (transport personnel);[d] START-UP (other)
Lab supplies	0.81	0.81	0.92	0.00	Scale-Up (transport fuel);[e] START-UP (other)
Lab equipment	0.21	0.21	0.32	0.00	START-UP
**Total direct medical cost**	**11.96**	**11.04**	**7.26**	**4.19**	

[a] HPV: human papillomavirus; Scale-Up: Scale-Up demonstration project (2015); START-UP: Screening Technologies to Advance Rapid Testing for Cervical Cancer Prevention–Utility and Program Planning demonstration project (2010); US$: United States dollars; VIA: visual inspection with acetic acid.

[b] HPV testing with both provider-collection of cervical samples and self-collection of vaginal samples was assumed to take place at the clinic. The difference in costs between collection modalities is due to reduced clinical staff time when a woman self-collects her own sample.

[c] The cost of clinical supplies for HPV testing was drawn from the Scale-Up project and includes the test kit, plate sealers, brush, collection medium, pipette tips (3 types), gloves, and towels to decant and dry.

[d] The cost of laboratory staff time was drawn from the START-UP project, with the exception of preparing and transporting samples to the laboratory, which was drawn from the Scale-Up project. Staff time spent preparing samples for transport in the Scale-Up project was based on project staff estimates in each of the three departments (Carazo, Chontales, and Chinandega), which ranged from 5 to 7 minutes per sample. Staff time for round-trip transport of samples was averaged across 9 to 10 facilities in each of the three departments, and valued based on the average monthly wage of the personnel responsible for transporting the samples in each department. We derived an average cost per sample prepared and transported for each department (assuming the cost of staff time per sample was equivalent for HPV and cytology specimens), and then averaged these costs across the three departments.

[e] For HPV testing, all lab supplies are bundled in the category of clinical supplies, with the exception of fuel for laboratory transport, which is included under lab supplies. For both HPV and cytology testing, we assumed the same cost of fuel per sample. Using Scale-Up project staff estimates of the number of samples transported per load, the distance to the lab from 9 to 10 facilities in each department, and the usual type of vehicle used in each department, we derived the average fuel cost per sample using the average fuel economy of major vehicle types [16] and the average gas price per liter in Nicaragua [12].

For peer review only - http://bmjopen.bmj.com/site/about/guidelines.xhtml

For peer review only

Table A.4. Diagnosis and Treatment of Precancer: Direct medical costs per procedure (2015 US$).[a]

Cost category	Colposcopy	Colposcopy and biopsy	Cryotherapy	LEEP	Source
Clinical staff	5.92	6.29	7.54	7.34	START-UP
Clinical supplies	0.38	1.39	3.54	36.05	Scale-Up (cryotherapy gas);[b] START-UP (other)
Drugs	0.13	0.13	0.30	0.10	START-UP
Clinical equipment	0.49	0.53	9.13	13.29	Scale-Up (cryotherapy unit);[c] START-UP (other)
Lab staff	0.00	4.09	0.00	4.09	START-UP
Lab supplies	0.00	3.35	0.00	3.35	START-UP
Lab equipment	0.00	4.12	0.00	4.12	START-UP
**Total direct medical cost**	**6.91**	**19.91**	**18.16**	**68.36**	

[a] LEEP: loop electrosurgical excision procedure; Scale-Up: Scale-Up demonstration project (2015); START-UP: Screening Technologies to Advance Rapid Testing for Cervical Cancer Prevention–Utility and Program Planning demonstration project (2010); US$: United States dollars.

[b] We used estimates from Scale-Up project staff to inform the cost of gas used per cryotherapy procedure, based on the cost of 1 cylinder and the number of women treated per tank. We did not include the cost of the tank deposit.

[c] To estimate the cost per procedure of a cryotherapy unit, we assumed this equipment item was a tradable good, and accordingly converted the purchase price from local currency units to US$ using the official exchange rate. We annualized the cost with a 3% interest rate and assumed an economic life of 5 years. To derive the number of procedures per machine per year across the three departments considered, we divided the annual number of cryotherapy procedures by the number of cryotherapy units. We then divided the annualized cryotherapy unit cost by the number of procedures per machine per year.

For peer review only - http://bmjopen.bmj.com/site/about/guidelines.xhtml

For peer review only

Table A.5. Location of service delivery for screening, diagnosis, and treatment of precancerous lesions and cancer.[a]

Procedure	Location of services[b]
HPV DNA test	Primary facility
Cytology test	Primary facility
VIA test	Primary facility
Colposcopy/biopsy	Referral facility
Cryotherapy	Referral facility
LEEP	Referral facility
Follow-up visits (after cryotherapy or LEEP)	Primary facility
Cancer treatment	Tertiary facility

[a] HPV: human papillomavirus; LEEP: loop electrosurgical excision procedure; VIA: visual inspection with acetic acid.

For peer review only

For peer review only - http://bmjopen.bmj.com/site/about/guidelines.xhtml

Tabe A.6. Women's time estimates.[a]

Visit	Minutes	Cost (2015 US$)[b]	Source
**Screening**[c]			
Wait time	33	0.48	Scale-Up
Procedure time	20	0.29	START-UP
Transport time (round-trip)	57	0.82	Scale-Up
**Results**[c]			
Wait time	33	0.48	Scale-Up
Procedure time	2	0.03	START-UP
Transport time (round-trip)	57	0.82	Scale-Up
**Cryotherapy**[c]			
Wait time	120	1.75	Scale-Up
Procedure time	35	0.51	START-UP
Transport time (round-trip)	213	3.10	Scale-Up
**Colposcopy/biopsy**			
Wait time	120	1.75	Scale-Up
Procedure time	37	0.54	START-UP
Transport time (round-trip)	213	3.10	Scale-Up
**LEEP**			
Wait time	120	1.75	Scale-Up
Procedure time	25	0.36	START-UP
Transport time (round-trip)	213	3.10	Scale-Up

[a] LEEP: loop electrosurgical excision procedure; Scale-Up: Scale-Up demonstration project (2015); START-UP: Screening Technologies to Advance Rapid Testing for Cervical Cancer Prevention–Utility and Program Planning demonstration project (2010); US$: United States dollars.

[b] Women's time was valued using Nicaragua's 2013 monthly minimum wage [13], converted to 2015 US$. We converted this to an hourly wage rate assuming 171 hours of work per month (40 hours per week).

[c] Wait and transport time costs were accrued for each visit to a health facility, but we did not double-count wait and transport times when multiple procedures were performed in the same visit (e.g., VIA triage of screen-positive women and cryotherapy of VIA+ women accrued the wait time and transport time costs associated with a single visit to a referral facility, but accrued the time costs of both procedures.)

For peer review only - http://bmjopen.bmj.com/site/about/guidelines.xhtml

Table A.7. Women's transportation costs.[a]

Facility[b]	Round-trip transportation cost (2015 US$)	Source
Screening facility	0.41	Scale-Up
Referral facility	2.81	Scale-Up

[a] Scale-Up: Scale-Up demonstration project (2015); US$: United States dollar.

[b] Screening with cytology or HPV testing was assumed to take place at a screening facility (i.e., a primary level health facility). Triage testing with visual inspection with acetic acid (VIA), cryotherapy, colposcopy/biopsy, and loop electrosurgical excision procedure (LEEP) were assumed to take place at referral facilities.

For peer review only

For peer review only - http://bmjopen.bmj.com/site/about/guidelines.xhtml

Table A.8. Programmatic training costs for HPV-based screening.[a]

Type of training	Total cost of supplies/materials[b] (2015 US$)	Total cost of personnel time[c] (2015 US$)	Per woman cost[d] (2015 US$)
Health care personnel offering careHPV	2,758	5,784	0.09
Laboratory technicians processing careHPV assays	3,503	526	0.04
Community outreach workers promoting and offering self-collection	2,662	3,618	0.08
Health care providers offering VIA and cryotherapy	8,103	16,069	1.51

[a] HPV: human papillomavirus; US$: United States dollars; VIA: visual inspection with acetic acid.

[b] The total cost of supplies, materials, transportation, and refreshments for all training sessions of each type was converted from 2015 local currency units using the official exchange rate.

[c] The total cost of personnel time includes time costs for both the trainer and the trainees, valued using average monthly salary rates of each type of personnel present (and the proportion of trainees of each personnel type) and based on the length of the training session.

[d] To derive an average health care personnel training cost per woman screened, we divided the total training cost for health care personnel by 100,000, corresponding to the number of women screened during the Scale-Up project. To derive an average laboratory technician training cost per woman screened, we divided the total training cost for laboratory technicians by 100,000. To derive an average community outreach worker training cost per woman screened with self-collection, we divided the total training cost for outreach workers by 80,000, corresponding to the number of women who participated in self-collection of HPV samples (due to lack of data on self-collection in a community setting, in the base case analysis we assumed self-collection took place at the clinic; however, we assumed outreach workers would be involved in mobilizing and educating women about self-collection, and conservatively included the cost of training so as to represent a known programmatic cost as self-collection efforts shift to also include community settings). To derive an average health care provider training cost per woman receiving VIA or cryotherapy, we divided the total VIA/cryotherapy training cost for health care providers by 16,000, corresponding to the approximate number of women who tested HPV-positive in Scale-Up and would thus require visual assessment and cryotherapy.

For peer review only - http://bmjopen.bmj.com/site/about/guidelines.xhtml

Table A.9. Cancer treatment costs (2015 US$).			
**Cancer stage**	**Local cancer**	**Regional cancer**	**Distant cancer**
Direct medical costs[a]	943.62	917.83	917.83
Woman and support person time	345.77	639.14	639.14
Transportation, temporary housing, and meals	196.78	389.50	389.50
**Total cancer treatment cost**	**1486**	**1946**	**1946**

[a] Direct medical costs are based on the premise that 50% of local cancer patients presented with FIGO stage 1a, requiring radical hysterectomy, and 50% of local cancer patients presented with FIGO stage 1b/2a, requiring cobalt therapy followed by 5 chemotherapy sessions followed by brachytherapy. We assumed all women presenting with FIGO stage 2b or higher received cobalt therapy followed by 5 chemotherapy sessions followed by brachytherapy.

[b] Patient and support person time costs value time using the minimum wage in Nicaragua, as described in the text.

[c] Direct non-medical costs include round-trip transportation for visits to a cancer center and meals and temporary housing during the course of treatment.

For peer review only - http://bmjopen.bmj.com/site/about/guidelines.xhtml

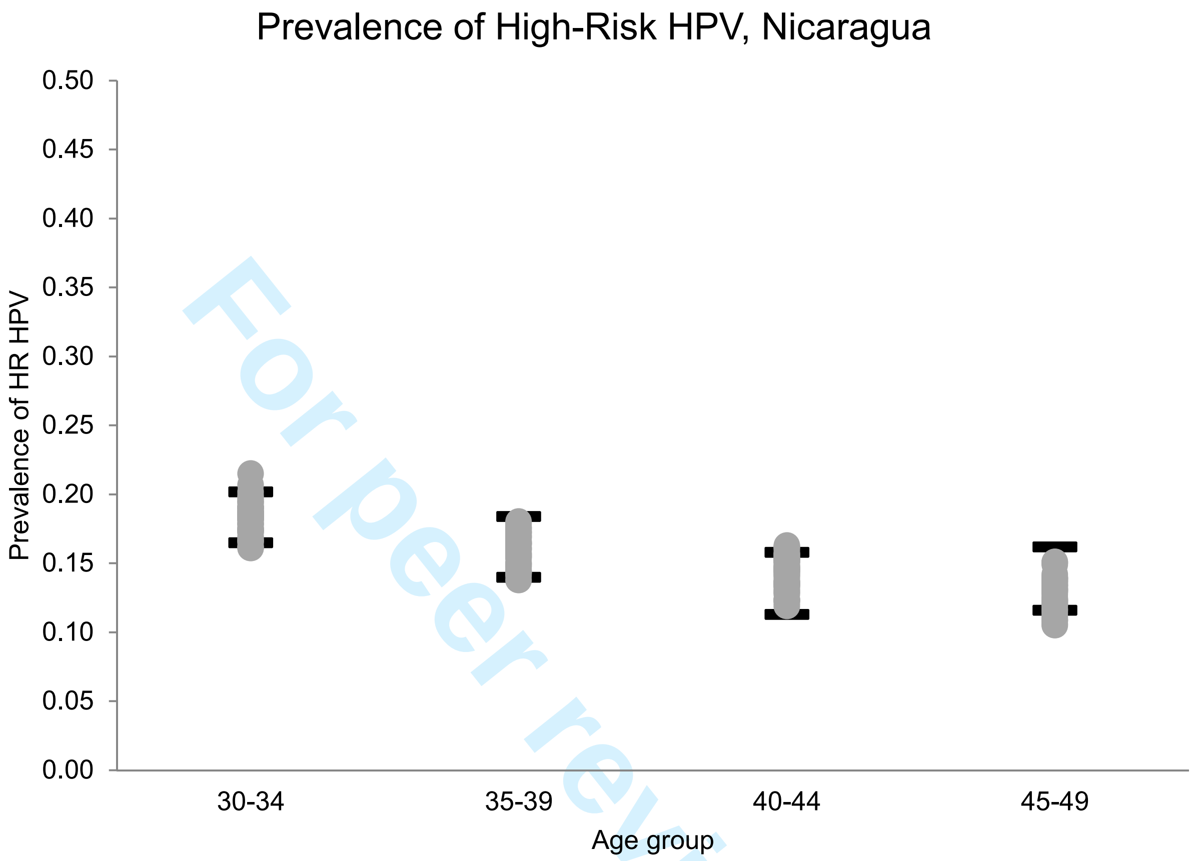

Figure A1. Selected model output from the top 50 input parameter sets compared with empirical data (i.e., calibration targets) on age-specific prevalence of high-risk HPV in Nicaragua, based on a relative light unit cut-off value of 0.5 in the START-UP studies [2, 15]. Bold lines represent the 95% confidence intervals around the empirical data, and gray circles represent model output from each of the top 50 input parameter sets.

For peer review only - http://bmjopen.bmj.com/site/about/guidelines.xhtml

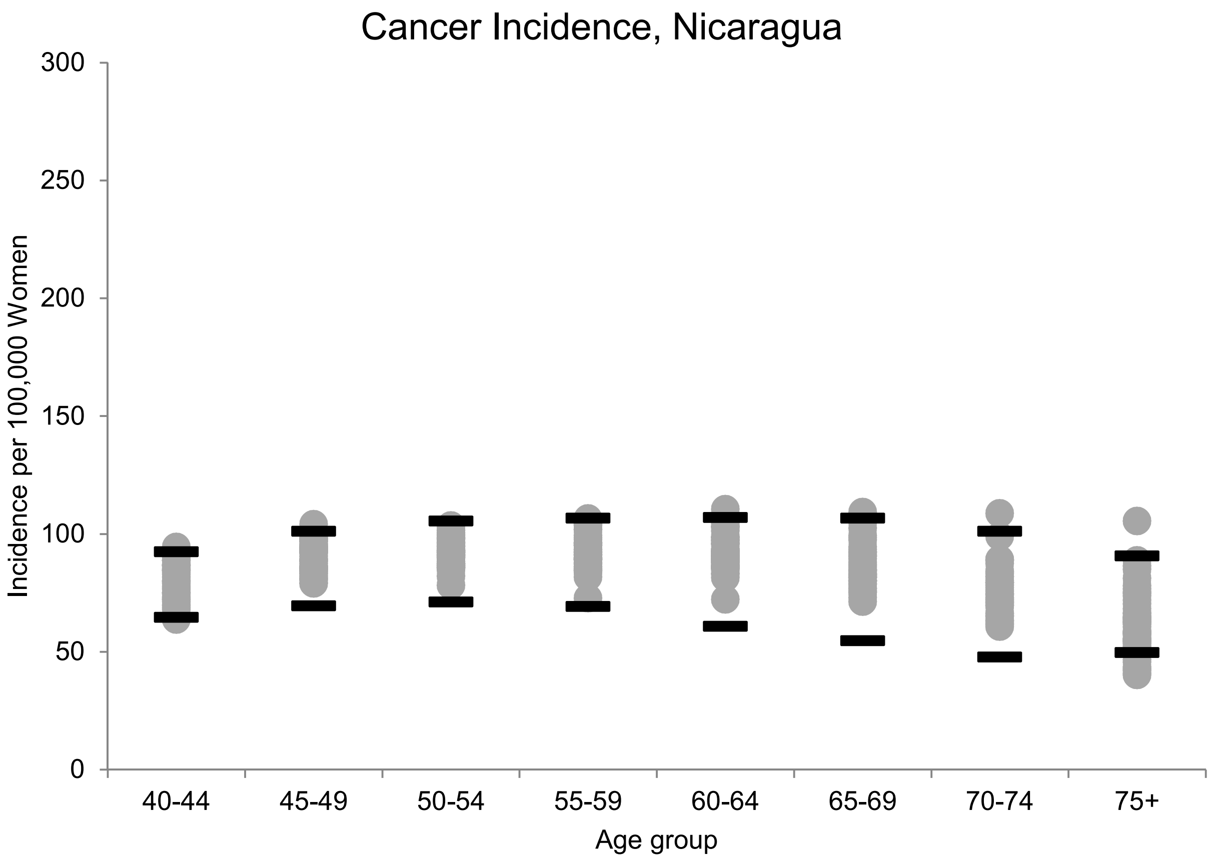

Figure A2. Selected model output from the top 50 input parameter sets compared with empirical data (i.e., calibration targets) on age-specific cancer incidence in Nicaragua [2, 11]. Bold lines represent the 95% confidence intervals around the empirical data, and gray circles represent model output from each of the top 50 input parameter sets.

For peer review only - http://bmjopen.bmj.com/site/about/guidelines.xhtml

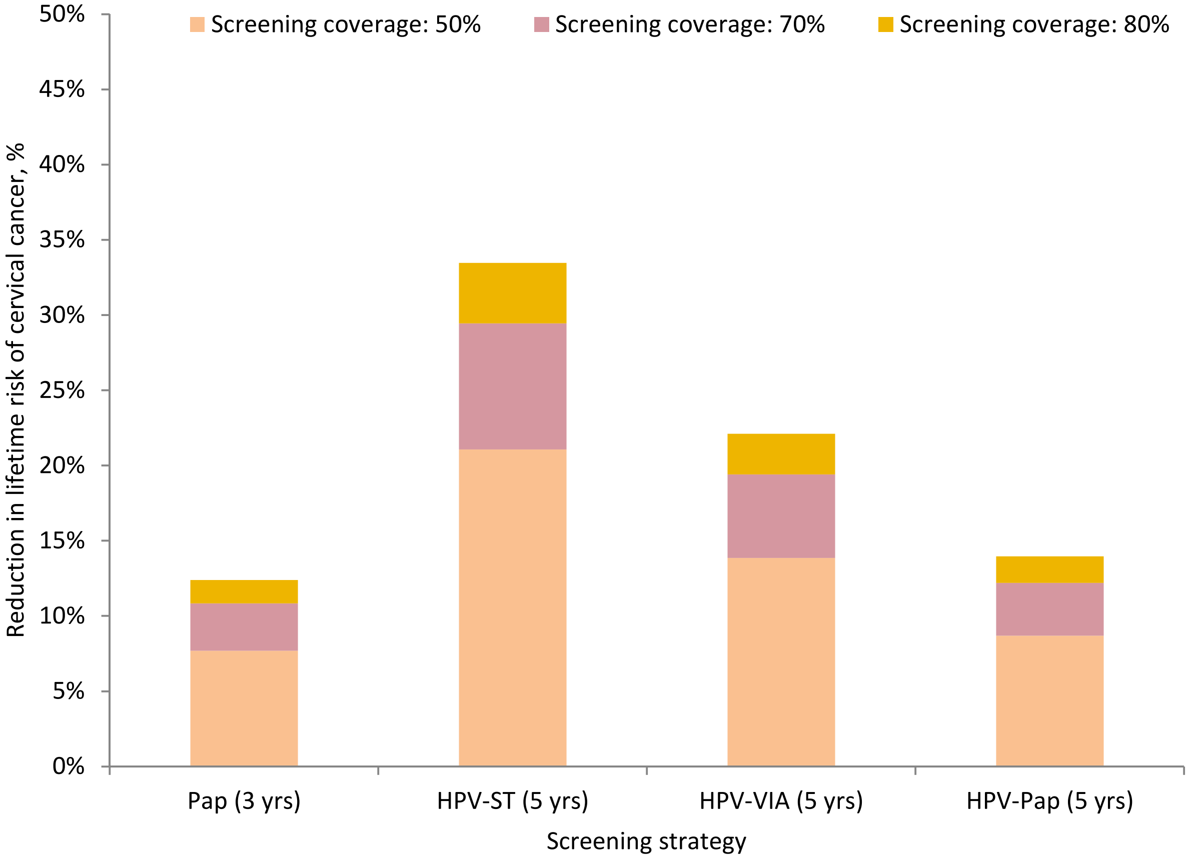

Figure A3. Reduction in lifetime risk of cervical cancer, by screening coverage level. Bars indicate the percent reduction in lifetime risk of cervical cancer for each screening strategy (Pap testing every 3 years; careHPV screen-and-treat every 5 years [HPV-ST]; careHPV every 5 years with visual inspection with acetic acid [VIA] triage of HPV-positive women [HPV-VIA]; and careHPV every 5 years with Pap triage of HPV-positive women [HPV-Pap]) as screening coverage of the target population increases.

For peer review only - http://bmjopen.bmj.com/site/about/guidelines.xhtml

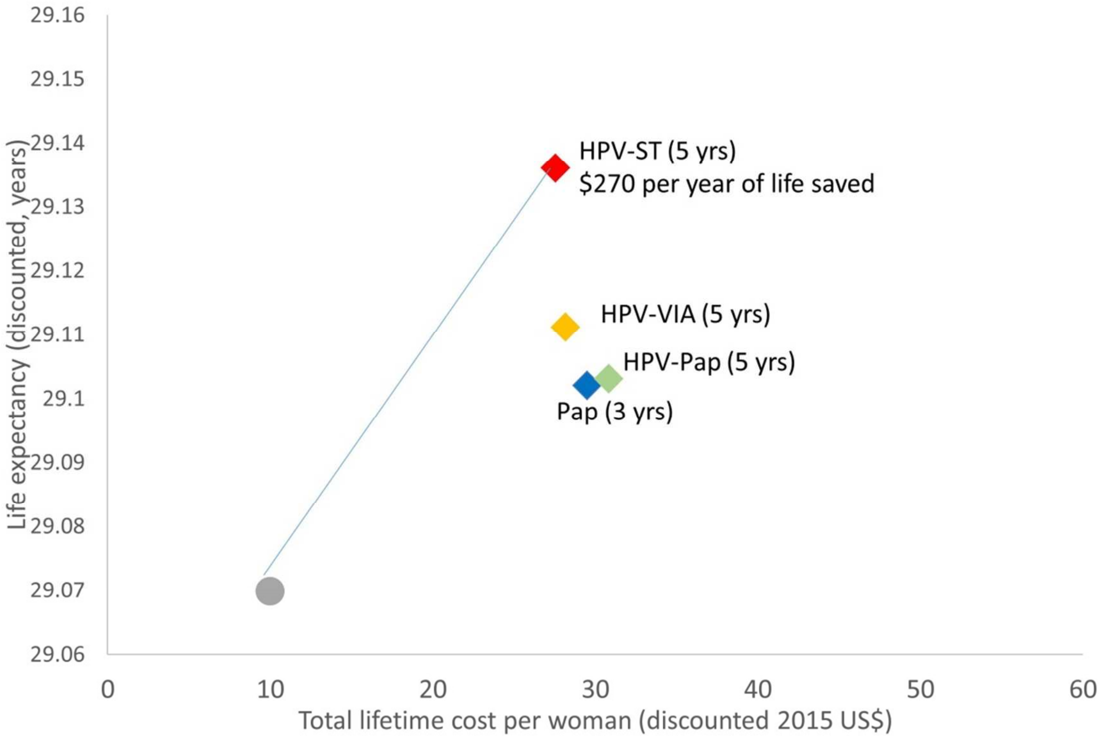

Figure A4. Cost-effectiveness analysis: Payer cost only. The graph displays the discounted lifetime costs (x-axis; in 2015 US$) and life expectancy (y-axis) associated with each screening strategy (Pap testing every 3 years; careHPV screen-and-treat every 5 years [HPV-ST]; careHPV every 5 years with visual inspection with acetic acid [VIA] triage of HPV-positive women [HPV-VIA]; and careHPV every 5 years with Pap triage of HPV-positive women [HPV-Pap]), when only costs to the payer are included (i.e., women's time and transportation costs are excluded). The cost-effectiveness associated with a change from one strategy to a more costly alternative is represented by the difference in cost divided by the difference in life expectancy associated with the two strategies. The curve indicates the strategies that are efficient because they are more effective and either 1) cost less; or 2) have a more attractive cost-effectiveness ratio than less effective options. The incremental cost-effectiveness ratio (ICER) is the reciprocal of the slope of the line connecting the two strategies under comparison. In this sensitivity analysis focused on costs to the payer, HPV-ST every 5 years was less costly and more effective than other screening strategies considered, and was thus a dominant strategy with an ICER of US$270 per year of life saved.

For peer review only - http://bmjopen.bmj.com/site/about/guidelines.xhtml

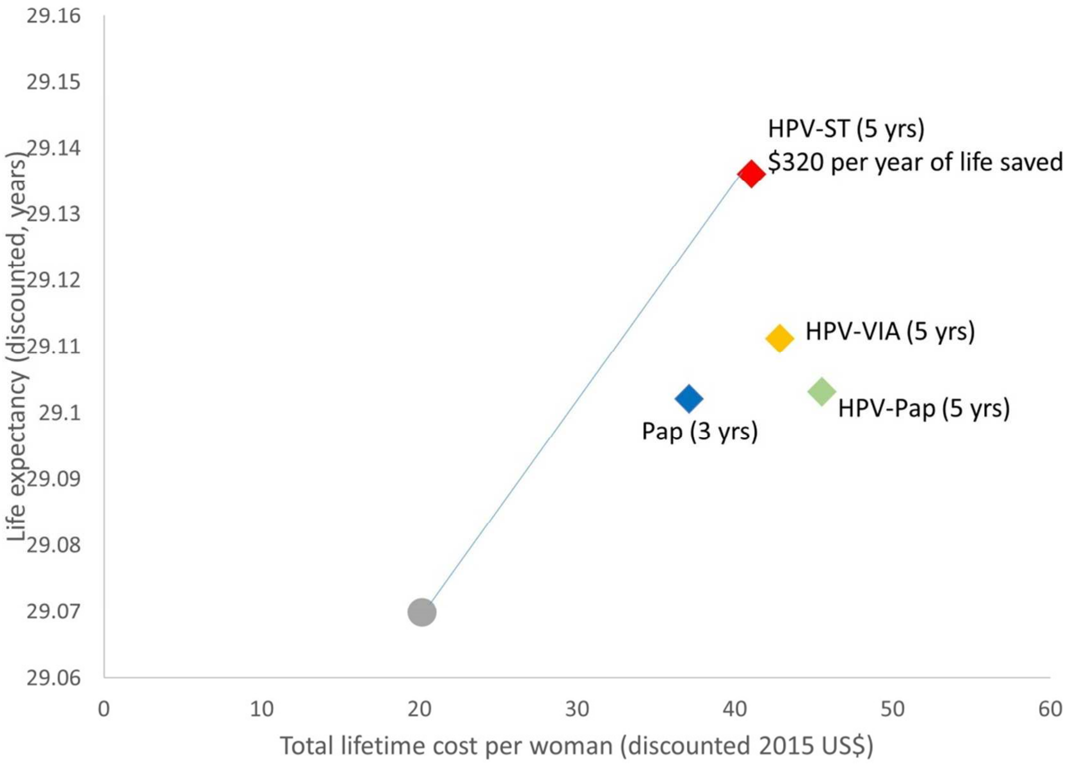

Figure A5. Cost-effectiveness analysis: Direct medical cost of Pap testing, US$3. The graph displays the discounted lifetime costs (x-axis; in 2015 US$) and life expectancy (y-axis) associated with each screening strategy (Pap testing every 3 years; careHPV screen-and-treat every 5 years [HPV-ST]; careHPV every 5 years with visual inspection with acetic acid [VIA] triage of HPV-positive women [HPV-VIA]; and careHPV every 5 years with Pap triage of HPV-positive women [HPV-Pap]), when the direct medical cost of Pap testing was assumed to be as low as US$3 (base case: US$7.26). The cost-effectiveness associated with a change from one strategy to a more costly alternative is represented by the difference in cost divided by the difference in life expectancy associated with the two strategies. The curve indicates the strategies that are efficient because they are more effective and either 1) cost less; or 2) have a more attractive cost-effectiveness ratio than less effective options. The incremental cost-effectiveness ratio (ICER) is the reciprocal of the slope of the line connecting the two strategies under comparison. In this sensitivity analysis examining the impact of a lower Pap test cost, HPV-ST every 5 years was more effective and had a more attractive cost-effectiveness ratio than Pap testing every 3 years; furthermore, HPV-ST was more effective and less costly than HPV-VIA and HPV-Pap, and was thus a dominant strategy with an ICER of US$320 per year of life saved.

For peer review only - http://bmjopen.bmj.com/site/about/guidelines.xhtml

References

1. Campos, N.G., et al., *An updated natural history model of cervical cancer: derivation of model parameters.* Am J Epidemiol, 2014. **180**(5): p. 545-55.
2. Campos, N.G., Tsu, V., Jeronimo, J., Mvundura, M., Lee, K., Kim, J.J., *When and how often to screen for cervical cancer in three low- and middle-income countries: A cost-effectiveness analysis.* Papillomavirus Research, 2015.
3. Munoz, N., et al., *Incidence, duration, and determinants of cervical human papillomavirus infection in a cohort of Colombian women with normal cytological results.* J Infect Dis, 2004. **190**(12): p. 2077-87.
4. McCredie, M.R., et al., *Natural history of cervical neoplasia and risk of invasive cancer in women with cervical intraepithelial neoplasia 3: a retrospective cohort study.* Lancet Oncol, 2008. **9**(5): p. 425-34.
5. Meyskens, F.L., Jr., et al., *Enhancement of regression of cervical intraepithelial neoplasia II (moderate dysplasia) with topically applied all-trans-retinoic acid: a randomized trial.* J Natl Cancer Inst, 1994. **86**(7): p. 539-43.
6. Keefe, K.A., et al., *A randomized, double blind, Phase III trial using oral beta-carotene supplementation for women with high-grade cervical intraepithelial neoplasia.* Cancer Epidemiol Biomarkers Prev, 2001. **10**(10): p. 1029-35.
7. Castle, P.E., et al., *Evidence for frequent regression of cervical intraepithelial neoplasia-grade 2.* Obstet Gynecol, 2009. **113**(1): p. 18-25.
8. Wang, S.M., et al., *Six-year regression and progression of cervical lesions of different human papillomavirus viral loads in varied histological diagnoses.* Int J Gynecol Cancer, 2013. **23**(4): p. 716-23.
9. Moscicki, A.B., et al., *Rate of and risks for regression of cervical intraepithelial neoplasia 2 in adolescents and young women.* Obstet Gynecol, 2010. **116**(6): p. 1373-80.
10. Herrero, R., et al., *Rationale and design of a community-based double-blind randomized clinical trial of an HPV 16 and 18 vaccine in Guanacaste, Costa Rica.* Vaccine, 2008. **26**(37): p. 4795-808.
11. Ferlay, J., et al., *GLOBOCAN 2012 v1.0, Cancer Incidence and Mortality Worldwide: IARC CancerBase No. 11 [Internet]*, 2013, International Agency for Research on Cancer: Lyon, France.
12. *World Development Indicators*, 2016, World Bank.
13. Database, I., *Country Profiles: Nicaragua*, 2016.
14. Campos, N.G., et al., *The comparative and cost-effectiveness of HPV-based cervical cancer screening algorithms in El Salvador.* Int J Cancer, 2015.
15. Jeronimo, J., et al., *A multicountry evaluation of careHPV testing, visual inspection with acetic acid, and papanicolaou testing for the detection of cervical cancer.* Int J Gynecol Cancer, 2014. **24**(3): p. 576-85.
16. Energy, U.S.D.o. *Average Fuel Economy of Major Vehicle Categories*. August 1, 2016]; Available from: http://www.afdc.energy.gov/data/10310.

For peer review only - http://bmjopen.bmj.com/site/about/guidelines.xhtml

CHEERS Checklist
Items to include when reporting economic evaluations of health interventions

The **ISPOR CHEERS Task Force Report**, *Consolidated Health Economic Evaluation Reporting Standards (CHEERS)—Explanation and Elaboration: A Report of the ISPOR Health Economic Evaluations Publication Guidelines Good Reporting Practices Task Force,* provides examples and further discussion of the 24-item CHEERS Checklist and the CHEERS Statement. It may be accessed via the *Value in Health* or via the ISPOR Health Economic Evaluation Publication Guidelines – CHEERS: Good Reporting Practices webpage: http://www.ispor.org/TaskForces/EconomicPubGuidelines.asp

Section/item	Item No	Recommendation	Reported on page No/ line No
**Title and abstract**			
Title	1	Identify the study as an economic evaluation or use more specific terms such as "cost-effectiveness analysis", and describe the interventions compared.	1/1
Abstract	2	Provide a structured summary of objectives, perspective, setting, methods (including study design and inputs), results (including base case and uncertainty analyses), and conclusions.	2/18
**Introduction**			
Background and objectives	3	Provide an explicit statement of the broader context for the study. Present the study question and its relevance for health policy or practice decisions.	6/112
**Methods**			
Target population and subgroups	4	Describe characteristics of the base case population and subgroups analysed, including why they were chosen.	7/119; 8/126
Setting and location	5	State relevant aspects of the system(s) in which the decision(s) need(s) to be made.	7/119; 8/126
Study perspective	6	Describe the perspective of the study and relate this to the costs being evaluated.	8/137
Comparators	7	Describe the interventions or strategies being compared and state why they were chosen.	10/170-190
Time horizon	8	State the time horizon(s) over which costs and consequences are being evaluated and say why appropriate.	8/129
Discount rate	9	Report the choice of discount rate(s) used for costs and outcomes and say why appropriate.	8/139
Choice of health outcomes	10	Describe what outcomes were used as the measure(s) of benefit in the evaluation and their relevance for the type of analysis performed.	8/129
Measurement of effectiveness	11a	*Single study-based estimates:* Describe fully the design features of the single effectiveness study and why the single study was a sufficient source of clinical effectiveness data.	NA

	11b	*Synthesis-based estimates:* Describe fully the methods used for identification of included studies and synthesis of clinical effectiveness data.	Table 1
Measurement and valuation of preference based outcomes	12	If applicable, describe the population and methods used to elicit preferences for outcomes.	NA
Estimating resources and costs	13a	*Single study-based economic evaluation:* Describe approaches used to estimate resource use associated with the alternative interventions. Describe primary or secondary research methods for valuing each resource item in terms of its unit cost. Describe any adjustments made to approximate to opportunity costs.	Appendix p.2-4/58-155
	13b	*Model-based economic evaluation:* Describe approaches and data sources used to estimate resource use associated with model health states. Describe primary or secondary research methods for valuing each resource item in terms of its unit cost. Describe any adjustments made to approximate to opportunity costs.	NA
Currency, price date, and conversion	14	Report the dates of the estimated resource quantities and unit costs. Describe methods for adjusting estimated unit costs to the year of reported costs if necessary. Describe methods for converting costs into a common currency base and the exchange rate.	Appendix p.2 /63-68
Choice of model	15	Describe and give reasons for the specific type of decision-analytical model used. Providing a figure to show model structure is strongly recommended.	8/143-167
Assumptions	16	Describe all structural or other assumptions underpinning the decision-analytical model.	Refs 17+18 (cited p.8/144)
Analytical methods	17	Describe all analytical methods supporting the evaluation. This could include methods for dealing with skewed, missing, or censored data; extrapolation methods; methods for pooling data; approaches to validate or make adjustments (such as half cycle corrections) to a model; and methods for handling population heterogeneity and uncertainty.	10 / 144-167; Appendix p.1-4
**Results**			
Study parameters	18	Report the values, ranges, references, and, if used, probability distributions for all parameters. Report reasons or sources for distributions used to represent uncertainty where appropriate. Providing a table to show the input values is strongly recommended.	Table 1
Incremental costs and outcomes	19	For each intervention, report mean values for the main categories of estimated costs and outcomes of interest, as well as mean differences between the comparator groups. If applicable, report incremental cost-effectiveness ratios.	Figure 3
Characterising uncertainty	20a	*Single study-based economic evaluation:* Describe the effects of sampling uncertainty for the estimated incremental cost and incremental effectiveness parameters, together with the impact	NA

		of methodological assumptions (such as discount rate, study perspective).	[illegible]
	20b	*Model-based economic evaluation:* Describe the effects on the results of uncertainty for all input parameters, and uncertainty related to the structure of the model and assumptions.	13-14/299-288
Characterising heterogeneity	21	If applicable, report differences in costs, outcomes, or cost-effectiveness that can be explained by variations between subgroups of patients with different baseline characteristics or other observed variability in effects that are not reducible by more information.	NA
**Discussion**			
Study findings, limitations, generalisability, and current knowledge	22	Summarise key study findings and describe how they support the conclusions reached. Discuss limitations and the generalisability of the findings and how the findings fit with current knowledge.	15-19/291-386
**Other**			
Source of funding	23	Describe how the study was funded and the role of the funder in the identification, design, conduct, and reporting of the analysis. Describe other non-monetary sources of support.	4 / 57-61
Conflicts of interest	24	Describe any potential for conflict of interest of study contributors in accordance with journal policy. In the absence of a journal policy, we recommend authors comply with International Committee of Medical Journal Editors recommendations.	4 / 62-66

For consistency, the CHEERS Statement checklist format is based on the format of the CONSORT statement checklist

The **ISPOR CHEERS Task Force Report** provides examples and further discussion of the 24-item CHEERS Checklist and the CHEERS Statement. It may be accessed via the *Value in Health* link or via the ISPOR Health Economic Evaluation Publication Guidelines – CHEERS: Good Reporting Practices webpage: http://www.ispor.org/TaskForces/EconomicPubGuidelines.asp

The citation for the CHEERS Task Force Report is:
Husereau D, Drummond M, Petrou S, et al. Consolidated health economic evaluation reporting standards (CHEERS)—Explanation and elaboration: A report of the ISPOR health economic evaluations publication guidelines good reporting practices task force. Value Health 2013;16:231-50.

BMJ Open

The Cost-Effectiveness of HPV-Based Cervical Cancer Screening in the Public Health System in Nicaragua

Journal:	*BMJ Open*
Manuscript ID	bmjopen-2016-015048.R1
Article Type:	Research
Date Submitted by the Author:	08-Feb-2017
Complete List of Authors:	Campos, Nicole; Harvard T.H. Chan School of Public Health, Health Policy and Management<br>Mvundura, Mercy; PATH, Devices and Tools Program<br>Jeronimo, Jose; PATH, Reproductive Global Health Program<br>Holme, Francesca; PATH, Reproductive Health Global Program<br>Vodicka, Elisabeth; University of Washington, School of Pharmacy<br>Kim, Jane; Harvard School of Public Health, Health Policy and Management
**Primary Subject Heading**:	Health economics
Secondary Subject Heading:	Global health, Health economics, Health policy, Obstetrics and gynaecology, Public health
Keywords:	HEALTH ECONOMICS, Epidemiology < ONCOLOGY, PUBLIC HEALTH

SCHOLARONE™
Manuscripts

For peer review only

The Cost-Effectiveness of HPV-Based Cervical Cancer Screening in the Public Health System in Nicaragua

Nicole G. Campos, PhD, [a] Mercy Mvundura, PhD,[b] Jose Jeronimo, MD,[c] Francesca Holme, MPH,[c] Elisabeth Vodicka, MHA,[d] Jane J. Kim, PhD[a]

[a] Center for Health Decision Science, Harvard T.H. Chan School of Public Health, 718 Huntington Avenue, Boston, Massachusetts, USA

[b] PATH, Devices and Tools Program, P.O. Box 900922, Seattle, Washington, USA

[c] PATH, Reproductive Health Global Program, P.O. Box 900922, Seattle, Washington, USA

[d] University of Washington, School of Pharmacy, Seattle, Washington, USA

Corresponding author: Nicole G. Campos, 718 Huntington Avenue, Boston, MA 02115; e-mail: ncampos@hsph.harvard.edu; phone: 617-432-2019; fax: 617-432-0190

Word count (abstract): 300

Word count (text): 4,527

For peer review only - http://bmjopen.bmj.com/site/about/guidelines.xhtml

Abstract

Objectives: To evaluate the cost-effectiveness of HPV DNA testing (versus Pap-based screening) for cervical cancer screening in Nicaragua.

Design: A previously developed Monte Carlo simulation model of the natural history of HPV infection and cervical cancer was calibrated to epidemiologic data from Nicaragua. Cost data inputs were derived using a micro-costing approach in Carazo, Chontales, and Chinandega departments; test performance data were from a demonstration project in Masaya department.

Setting: Nicaragua's public health sector facilities.

Participants: Women aged 30 to 59 years.

Interventions: Screening strategies included 1) Pap testing every 3 years, with referral to colposcopy for women with an ASCUS+ result ("Pap"); 2) HPV testing every 5 years, with referral to cryotherapy for HPV-positive eligible women (HPV cryotherapy, or "HPV-Cryo"); 3) HPV testing every 5 years, with referral to triage with visual inspection with acetic acid (VIA) for HPV-positive women ("HPV-VIA"); and 4) HPV testing every 5 years, with referral to Pap testing for HPV-positive women ("HPV-Pap").

Outcome measures: Reduction in lifetime risk of cancer; incremental cost-effectiveness ratios (ICER; 2015 US$ per year of life saved [YLS]).

Results: HPV-based screening strategies were more effective than Pap testing. HPV-Cryo was the least costly and most effective strategy, reducing lifetime cancer risk by 29.5% and outperforming HPV-VIA, HPV-Pap, and Pap only, which reduced cancer risk by 19.4%, 12.2%, and 10.8%, respectively. With an ICER of US$320/YLS, HPV-Cryo every 5 years would be very cost-effective using a threshold based on Nicaragua's per capita GDP of US$2,090. Findings were robust across sensitivity analyses on test performance, coverage, compliance, and cost parameters.

For peer review only - http://bmjopen.bmj.com/site/about/guidelines.xhtml

Conclusions: HPV testing is very cost-effective compared to Pap testing in Nicaragua, due to higher test sensitivity and the relatively lower number of visits required. Increasing compliance with recommended follow-up will further improve the health benefits and value for public health dollars.

For peer review only

For peer review only - http://bmjopen.bmj.com/site/about/guidelines.xhtml

Strengths and limitations of this study:

- Using implementation data from the Scale-Up project— which aims to facilitate institutionalization of HPV testing at the national level in Guatemala, Honduras, and Nicaragua— we estimated the cost-effectiveness of HPV testing in Nicaragua's public health system.
- The screening algorithms, as modeled, reflect the prototypical structure of a screening episode and the type of facility at which visits usually take place, but do not capture variation due to geography or health facility capacity.
- While we adopted a micro-costing approach to leverage data from implementation in Nicaragua, individual-level data for each woman were not available; furthermore, we did not have cost data associated with HPV self-collection in community settings, where most self-collection takes place.
- Despite these limitations, extensive sensitivity analyses on cost and screening assumptions indicate that HPV-Cryo is robustly the most efficient strategy.

Funding statement: This work was supported by the Bill & Melinda Gates Foundation.

Disclaimer: The findings and conclusions contained within are those of the authors and do not necessarily reflect positions or policies of the Bill & Melinda Gates Foundation. The funders had no role in study design; data collection, analysis, and interpretation; preparation of the manuscript; or decision to submit the article for publication.

Competing interests disclosed: All authors have completed the ICMJE uniform disclosure form at www.icmje.org/coi_disclosure.pdf and declare financial support from the Bill and Melinda Gates Foundation for the submitted work; JJ was the director of the START-UP demonstration projects and

For peer review only - http://bmjopen.bmj.com/site/about/guidelines.xhtml

received all tests used in the study as a donation from Qiagen; no other relationships or activities that could appear to have influenced the submitted work.

Contributors: All authors developed the analysis plan and interpreted the data. NC, MM, JJ, FH, and JK conceptualized the study. MM, JJ, FH, and EV collected implementation data. NC conducted data analysis and wrote the first draft of the report and revised subsequent drafts. JJ was the principal investigator of the START-UP and Scale-Up projects. JK was the principal investigator overseeing microsimulation model development. All authors contributed to and approved the final report.

Data sharing statement: PATH provides technical assistance to the government of Nicaragua and has access to screening indicators through partnership with Movicáncer, a local non-governmental organization partner in Nicaragua. Movicáncer designed the health information system used by the Ministry of Health to track screening and treatment of women's cancers. The Ministry of Health protects individually identifiable information; PATH received de-identified and consolidated data on visit compliance from Movicáncer. Costing data were collected by Movicáncer through consultation with Ministry of Health personnel, mostly via phone interviews. No individual identifiers were collected. These data are not available from any public source. A supplementary appendix describing the costing data and microsimulation model is available.

Acknowledgements: We gratefully acknowledge the efforts of Movicáncer of Nicaragua in gathering costing data.

For peer review only

For peer review only - http://bmjopen.bmj.com/site/about/guidelines.xhtml

Background

Cervical cancer is the leading cause of cancer among women in Nicaragua, with an estimated 934 cases and 424 deaths each year [1]. Yet cervical cancer is preventable through screening that allows for early detection and subsequent treatment of precancerous lesions caused by sexually transmitted infection with human papillomavirus (HPV). While most HPV infections clear spontaneously within 1 to 2 years, a persistent infection with one of approximately 15 oncogenic HPV genotypes may progress to precancer, which, if untreated, may become invasive cancer [2, 3].

In most high-income countries, routine screening with cervical cytology (i.e., Pap) testing has substantially reduced the incidence of cervical cancer [4]. However, due to its low sensitivity to detect precancer, Pap testing must be performed at regular frequent intervals in women of screening age. In low- and middle-income settings, where many women do not have access to routine primary health care and even fewer have access to higher-level facilities that offer diagnostic testing and treatment, Pap testing has not been effective at reducing cervical cancer incidence and mortality [5]. In Nicaragua, an estimated 31.5% of women aged 15 to 49 years have been screened within the last year, and nearly 30% of women in this age group have never been screened [6]. One recent survey found that 87% of women in León, Nicaragua, were informed of their Pap results, but of those who were referred to follow-up, only 67% received further care [7].

HPV DNA tests are highly sensitive to detect potentially oncogenic HPV infections and present an alternative to Pap-based screening. Because HPV-negative women are at very low risk for developing cervical cancer within the next 10 years [8], the interval between screenings can be extended to at least 5 years for this subset of women [9]. An additional advantage of HPV testing is that samples can be collected by a provider or by the woman herself, reducing the burden on health workers and time women spend seeking care and potentially increasing screening uptake [10-13]. Furthermore, a lower-cost HPV DNA test known as careHPV has been clinically validated [14, 15] and is now commercialized.

For peer review only - http://bmjopen.bmj.com/site/about/guidelines.xhtml

Given these potential benefits, the World Health Organization (WHO) recommends HPV testing for countries with sufficient resources [9].

In 2011, the Screening Technologies to Advance Rapid Testing for Cervical Cancer Prevention−Utility and Program Planning (START-UP) project in Nicaragua's Masaya department demonstrated that screening with careHPV could be effectively implemented in public sector health facilities [14]. The Ministry of Health of Nicaragua subsequently built on these initial efforts, incorporating HPV testing into public health care systems in three departments with technical assistance from PATH under the Scale-Up project. Adoption of HPV testing within Nicaragua's public health care system is taking place in three phases [16]. In Phase 1, partner organizations worked with the Ministry of Health to prepare for introduction of HPV screening into public health facilities by developing screening and treatment algorithms; creating educational materials; organizing training sessions for health workers and laboratory technicians; and bolstering referral and treatment systems for follow-up of screen-positive women. Phase 2 piloted screening with 10,000 HPV tests in order to identify and address barriers to implementation. Phase 3 will expand coverage to over 50,000 women within one year.

To inform decision makers considering the national adoption and scale-up of HPV testing within Nicaragua's public health sector, this study aimed to 1) estimate the economic cost of cervical cancer screening with careHPV testing; and 2) project the long-term health and economic impact and value (i.e., cost-effectiveness) of careHPV testing in Nicaragua relative to existing Pap-based screening.

For peer review only

Methods

Analytic overview

We used a micro-costing approach to measure and aggregate the cost of all resources used to provide cervical cancer screening at the level of the individual patient within the public health sector in Nicaragua. We considered direct medical costs (i.e., medical resources required for the intervention), direct non-medical costs (i.e., other resources consumed as part of the intervention, such as patient

For peer review only - http://bmjopen.bmj.com/site/about/guidelines.xhtml

transportation costs), and patient time costs (i.e., time spent traveling and waiting for or receiving care). These cost data were input into a previously developed Monte Carlo simulation model (programmed in C++) of the natural history of HPV infection and cervical cancer that was calibrated to epidemiologic data from Nicaragua [17, 18]. We then used the model to project the lifetime health and economic outcomes associated with careHPV testing, using three different algorithms for the management of women who test HPV-positive, and Pap-based screening for women aged 30 to 59 years.

Model outcomes included the lifetime risk of cervical cancer, total lifetime costs per woman (in 2015 United States dollars [US$]), and life expectancy. Incremental cost-effectiveness ratios (ICERs) were calculated by dividing the additional cost of a particular strategy by its additional health benefit, compared with the next most costly strategy. Dominated strategies (defined as more costly and either less effective or having a higher cost-effectiveness ratio than more effective strategies) were eliminated. There is no universal criterion that defines a threshold cost-effectiveness ratio, below which an intervention is considered good value for money; we considered an intervention with an ICER less than Nicaragua's 2015 per capita GDP of US$2,090 to be "very cost-effective", and an intervention with an ICER less than three times per capita GDP as "cost-effective" [19]. We followed guidelines for cost-effectiveness by adopting a societal perspective, including costs irrespective of the payer in order to capture the opportunity cost of resources used for the screening intervention. We discounted future costs and life-years at a rate of 3% per year to account for time preferences [20, 21].

Mathematical simulation model

Descriptions of the natural history model of HPV infection and cervical carcinogenesis and model parameterization process have been previously published [17, 18], but we summarize model features here. Individual girls enter the model at age 9 years, prior to initiating sexual activity, and face monthly transitions between mutually exclusive health states that reflect disease progression, including

For peer review only - http://bmjopen.bmj.com/site/about/guidelines.xhtml

type-specific HPV infection, grade of precancer (i.e., cervical intraepithelial neoplasia [CIN] grade 2 or 3), and stage of invasive cancer. Transition probabilities may vary by age, HPV type, duration of infection or precancerous lesion status, prior HPV infection, and exposure to screening and treatment of HPV or precancer. Cervical cancer can be detected through symptoms or screening. Death can occur from non-cervical causes or from cervical cancer after its onset. The model tracks each individual woman's health status, clinical events, and economic outcomes over her lifetime, and aggregates outcomes to estimate the expected costs and health outcomes over the lifetime of the cohort.

The model was calibrated to epidemiologic data on age-specific HPV prevalence and cervical cancer incidence from Nicaragua [1, 14, 17]. We estimated baseline "prior" input parameter values for natural history transitions using available longitudinal data, including age- and type-specific HPV incidence data from Colombia [22-25]. To reflect potential differences in parameters that may vary by setting (i.e., age- and type-specific HPV incidence, natural immunity following initial infection) and uncertainty in progression and regression of precancer, we set plausible bounds around these input values and performed repeated model simulations of disease natural history in the absence of any intervention. Each model simulation selected one random value within the bounds for each uncertain parameter, creating a unique natural history input parameter set. By summing the log-likelihood of model-projected outcomes for each parameter set relative to the epidemiologic data from Nicaragua, we computed a goodness-of-fit score. We selected the 50 top-fitting input parameter sets to use in analysis. Results are reported as the mean across the top 50 parameter sets, and ICERs are reported as the ratio of the mean costs divided by the mean effects of one strategy versus another across sets [26]. Further details on model parameterization, including calibration, are available in the **Appendix**.

Cervical cancer screening strategies

For peer review only - http://bmjopen.bmj.com/site/about/guidelines.xhtml

We considered the following screening strategies for women aged 30 to 59 years (**Figure 1**): 1) Pap testing every 3 years, with referral to colposcopy for all women with an atypical squamous cells of undetermined significance or worse (ASCUS+) result (hereafter referred to as "Pap"); 2) careHPV testing every 5 years, with referral to cryotherapy for all HPV-positive eligible women (HPV cryotherapy, or "HPV-Cryo"); 3) careHPV testing every 5 years, with referral to triage with visual inspection with acetic acid (VIA) for all HPV-positive women ("HPV-VIA"); and 4) careHPV testing every 5 years, with referral to Pap testing for all HPV-positive women ("HPV-Pap"). The pathway of care for each strategy was based on patterns of care in the Scale-Up project, national screening guidelines, and WHO recommendations. We optimistically assumed 70% of women had access to routine screening and attended an initial visit at a screening facility (i.e., a primary health care facility). Women could then return to receive screening results and recommendations for any necessary follow-up care. Follow-up could include colposcopy, cryotherapy, or triage testing at a referral (i.e., higher level) facility, with the exception of Pap triage testing, which was assumed to take place at the screening clinic. At each encounter after the initial screening visit, we assumed 85% of women complied with each subsequent visit to a screening facility, while 40% complied with each subsequent visit to a referral facility, consistent with data from Phase 2 of the Scale-Up project. The minimum number of visits required for treatment in a single screening episode was 4 for Pap, 3 for HPV-Cryo, 3 for HPV-VIA, and 5 for HPV-Pap. In the HPV-VIA and HPV-Pap strategies, women who were HPV-positive but negative on the selected triage test were referred to repeat HPV testing in 1 year. In the HPV-Cryo and HPV-VIA strategies, women who were not eligible for treatment with cryotherapy based on visual assessment were referred to colposcopy with biopsy to rule out cancer; in the absence of cancer, these women were referred to treatment with loop electrosurgical excision procedure (LEEP) or cryotherapy.

Screening and treatment parameters are presented in **Table 1** [6, 14, 17, 27-32]. Screening test performance data were drawn from the START-UP project in Nicaragua to reflect local test

For peer review only

For peer review only - http://bmjopen.bmj.com/site/about/guidelines.xhtml

characteristics. While the START-UP project did not evaluate VIA and Pap as triage tests, we used the VIA and Pap positivity rates in HPV-positive women, along with published studies of triage test performance, to inform triage test sensitivity and specificity [33-38]; in the base case, we optimistically assumed high sensitivity of triage testing.

For all HPV testing strategies, we assumed 20% of women received provider-collection of cervical specimens and 80% of women self-collected vaginal specimens, consistent with the proportions in the Scale-Up project to date. We weighted cost and health outcomes for provider- and self-collection accordingly when aggregating results for the HPV strategies.

Cost data

All costs were converted to 2015 United States dollars (US$) using GDP deflators and the official exchange rate [39]. The direct medical costs of screening, diagnosis, and treatment of precancer were drawn from the START-UP study (Masaya department) and the Scale-Up project (Carazo, Chontales, and Chinandega departments). Direct medical costs included clinical staff time, clinical supplies, drugs, clinical equipment, laboratory staff time, laboratory supplies, and laboratory equipment. Direct non-medical costs included women's round-trip transportation costs to health facilities, and were based on estimates provided by Scale-Up project staff to represent average transportation costs in the Carazo, Chontales, and Chinandega departments. To account for the opportunity cost of women's time spent traveling to, waiting for, or receiving care, we used time estimates from the START-UP and Scale-Up projects, and valued women's time using Nicaragua's monthly minimum wage to serve as a proxy for the societal value of women's time. **Figure 2** displays the categorical breakdown of undiscounted costs for the screening visits over the course of a woman's screening-eligible years, with Pap versus careHPV testing.

For peer review only - http://bmjopen.bmj.com/site/about/guidelines.xhtml

For peer review only

Data on programmatic costs are limited, but for HPV strategies we included the cost of training sessions for health providers offering HPV-based screening, outreach workers, laboratory technicians, and providers offering VIA and cryotherapy. While women who self-collected HPV specimens in the Scale-Up project primarily did so in a community setting, micro-costing data were not available for self-collection performed outside of the clinic, so we assumed clinic-based self-collection. However, we conservatively included the cost of training outreach workers to represent a known programmatic cost as self-collection efforts are shifting to community settings.

Data on the costs of treating cervical cancer were unavailable for Nicaragua, so we estimated direct medical, direct non-medical, and patient and support person time costs using data from El Salvador [29].

Selected cost data are presented in **Table 1.** Further details on cost data are provided in the **Appendix**.

Sensitivity analyses

We performed sensitivity analysis to examine the impact of independently varying uncertain parameters, including Pap test performance, triage test performance in HPV-positive women, colposcopy performance, screening coverage, visit compliance, eligibility for cryotherapy following a positive screening and triage test, treatment effectiveness, discount rate, and cost data. Ranges selected for sensitivity analysis are displayed in **Table 1**.

Scenario analysis

The base case and sensitivity analysis assumed the availability of all strategies (i.e., Pap; HPV-Cryo; HPV-VIA; and HPV-Pap). Additionally, we performed a scenario analysis in which we assumed HPV-

For peer review only - http://bmjopen.bmj.com/site/about/guidelines.xhtml

Cryo was not available for logistical and programmatic reasons (i.e., limited access to cryotherapy equipment and gas).

Results

Base case: Population-level health benefits and cost-effectiveness analysis

HPV-based screening strategies were more effective than Pap testing. Among the HPV strategies, HPV-Cryo (every 5 years) was the most effective strategy; under base case assumptions, it reduced the lifetime risk of cervical cancer by 29.5% on average (range: 25.2%−33.6%). HPV-VIA (every 5 years) reduced cancer risk by 19.4% (range: 16.2%−22.6%), while HPV-Pap reduced cancer risk by 12.2% (range: 10.2%−14.5%). Pap (every 3 years) reduced cancer risk by 10.8% (range: 8.7%−13.4%). Under base case assumptions, HPV-Cryo was both less costly and more effective than all other strategies, thus dominating HPV-VIA, HPV-Pap, and Pap alone. With an ICER of US$320 per year of life saved (YLS), HPV-Cryo every 5 years would be considered "very cost-effective" given Nicaragua's per capita GDP of US$2,090. The total discounted lifetime cost per woman and life expectancy associated with each screening strategy is presented in **Figure 3**.

Sensitivity analysis

While HPV-Cryo remained the most effective strategy across all sensitivity analyses, the magnitude of reduction in lifetime risk of cancer was dependent upon screening coverage of the target population and compliance with recommended follow-up. When coverage was 50% and all other parameters were held constant at base case values, HPV-Cryo reduced cancer risk by an average of 21.1%; HPV-VIA, HPV-Pap, and Pap yielded average cancer risk reductions of 13.9%, 8.7%, and 7.7%, respectively. As coverage increased to 80%, all else being equal, HPV-Cryo reduced cancer risk by an average of 33.5%, while HPV-VIA, HPV-Pap, and Pap yielded average cancer risk reductions of 22.1%,

For peer review only - http://bmjopen.bmj.com/site/about/guidelines.xhtml

14.0%, and 12.4%, respectively (**Appendix**). **Figure 4** displays the impact of visit compliance on lifetime risk of cancer. When compliance with visits to all facilities (i.e., for both screening and referral) was low at 40%, HPV-Cryo remained the most effective strategy, but only reduced cancer risk by 16.2%; Pap had little health impact at this level of compliance, reducing cancer risk by only 5.4%. As compliance at all facilities rose to 85%, HPV-Cryo reduced cancer risk by 47.9%; HPV-VIA, HPV-Pap, and Pap reduced cancer risk by 42.0%, 40.7%, and 35.7% respectively.

In addition to remaining the most effective strategy across all sensitivity analyses, HPV-Cryo remained the most efficient strategy as well. HPV-Cryo remained the least costly and most effective strategy with a stable ICER of US$320 per YLS when 1) Pap test performance (as a primary screening test) improved; 2) VIA and Pap triage test performance improved; 3) colposcopy was assumed to be perfect; and 4) the direct medical cost of LEEP was varied from 75% to 125% of the base case. Despite slight fluctuation in the ICER, HPV-Cryo also remained the least costly and most effective strategy as 1) screening coverage varied from 50% to 80%; 2) visit compliance varied from 40% to 85% per visit; 3) the screen-and-treat cryotherapy cure rate was reduced to 75%; 4) eligibility for cryotherapy was reduced to 75% or less; 5) the treatment cure rate following colposcopy was reduced to 85%; 6) women receiving treatment following colposcopy in the Pap and HPV-Pap strategies were assumed to receive cryotherapy instead of the more costly LEEP; 7) the discount rate was varied from 0% to 5%; 8) only payer costs were considered, excluding women's time and transportation costs; 9) the direct medical cost of HPV self-collection was varied from 75% to 125% of the base case; 10) the direct medical cost of cryotherapy was increased to 170% of the base case; 11) the direct medical cost of colposcopy was reduced to 35% of the base case; 12) programmatic costs associated with HPV-based screening were varied from 50% to 150% of the base case; 13) women's time and transportation costs were reduced to 50% of the base case; and 14) the costs of cancer treatment ranged from including only direct medical costs to 150% of the base case (**Figure 5**). Among variables considered, compliance per visit appears to have the greatest impact

For peer review only - http://bmjopen.bmj.com/site/about/guidelines.xhtml

on the ICER for HPV-Cryo, with 40% compliance yielding an ICER of US$580 per YLS and 85% compliance yielding an ICER of US$190 per YLS. Even when visit compliance is low, HPV-Cryo would be considered very cost-effective.

When only payer costs were considered (i.e., women's time and transportation costs were excluded), the total lifetime cost per woman was lower for all strategies, and Pap every 3 years was slightly less costly (although still slightly less effective) than HPV-Pap every 5 years. HPV-Cryo remained the most effective and efficient strategy, with an ICER of US$270 per YLS (**Appendix**).

The only scenario in which Pap testing every 3 years was the least costly strategy occurred when the direct medical cost of Pap testing was US$3 (base case: US$7.26), a value commonly cited for the cost of Pap in Nicaragua, though the source of this estimate is unknown. However, Pap remained the least effective strategy, and HPV-Cryo had a lower cost-effectiveness ratio, maintaining an ICER of US$320 per YLS (**Appendix**).

Scenario analysis: HPV-Cryo unavailable

When we assumed HPV-Cryo was not available as a screening strategy, HPV-VIA was the least costly and most effective strategy in the base case and most sensitivity analyses, with a base case ICER of US$550 per YLS (**Appendix**). Exceptions included the following circumstances: 1) when the direct medical cost of Pap was US$3, Pap alone had a more attractive ICER (US$530), albeit was less effective than HPV-VIA (US$630 per YLS); 2) Pap performance in the general screening population was improved and Pap alone became the most effective strategy, with an ICER of US$540 per YLS; 3) VIA test sensitivity in HPV-positive women was only 0.40, in which case HPV-VIA had an ICER of US$726 per YLS, but HPV-Pap was more effective with an ICER of US$3,260.

Discussion

For peer review only - http://bmjopen.bmj.com/site/about/guidelines.xhtml

Using implementation data from the Scale-Up project— which aims to facilitate institutionalization of HPV testing at the national level in Guatemala, Honduras, and Nicaragua— we estimated the long-term health impact and value of careHPV testing in Nicaragua's public health system. We found that screening algorithms consisting of HPV testing at 5-year intervals would be less costly and more effective than screening with Pap testing at 3-year intervals. Furthermore, HPV testing followed by treatment with cryotherapy for all eligible HPV-positive women would be less costly and more effective than HPV testing followed by triage testing with either VIA or Pap for HPV-positive women. A screen-and-treat HPV program would be a very cost-effective intervention in Nicaragua, with an ICER of US$320 per YLS under base case assumptions. These findings were robust across sensitivity analyses. The comparatively large health benefits and efficiency of HPV-Cryo can largely be attributed to the relatively low number of visits to health care facilities and the high sensitivity of the careHPV test to detect both CIN2+ and oncogenic HPV infections with the potential to develop into precancer.

We found that screening coverage of the target population had a considerable impact on achievable reductions in cervical cancer risk, with HPV-Cryo yielding the greatest risk reduction. Due to proportional increases in both costs and health benefits, the ICER for HPV-Cryo remained stable as coverage increased from 50% to 80%. Compliance with recommended follow-up was a key driver of both achievable reductions in cancer risk and the ICER of HPV-Cryo. As the proportion of women who returned for each clinical encounter (relative to the previous visit) increased from 40% to 85%, the effectiveness or cancer benefit associated with HPV-Cryo rose from 16.2% to 47.9% as more women were linked to treatment; the ICER fell from US$580 per YLS to US$190 per YLS as more cancers were averted. Thus, improved efforts to successfully navigate women to recommended follow-up will enhance screening program effectiveness and efficiency.

While substantially reducing the cost of Pap testing to US$3 (less than half of the base case value) made Pap the strategy with the lowest per-woman lifetime costs, the confluence of low test

For peer review only - http://bmjopen.bmj.com/site/about/guidelines.xhtml

sensitivity and the high number of health facility visits needed to complete screening, diagnostic follow-up, and treatment made Pap the least effective strategy since women are lost to follow-up with each additional required visit. Even in this low-cost Pap scenario, HPV-Cryo remained the most effective and cost-effective strategy.

When we assumed a scenario in which HPV-Cryo was not available for logistic and programming reasons, we found that HPV-VIA was usually the least costly and most effective of the remaining strategies. Although the ICER was less attractive than the ICER associated with HPV-Cryo in the main analysis, it was below Nicaragua's per capita GDP.

There are several limitations to this analysis. We did not consider alternative screening intervals or ages for each strategy, but rather restricted the analysis to the ages and intervals currently under consideration by the Nicaraguan Ministry of Health. It is likely that an increased screening interval and fewer lifetime screens will also be cost-effective, although health benefits may be reduced; we demonstrated in a previous analysis that screening once or three times in a woman's lifetime with careHPV would be very cost-effective in Nicaragua [17]. The screening algorithms, as modeled, reflect the prototypical structure of a screening episode and the type of facility at which visits usually take place, but do not capture variation due to geography or health facility capacity. Furthermore, the modeled screening algorithms do not entirely reflect the complex downstream follow-up of screen-positive women that is embodied in the Ministry of Health's screening guidelines. In simplifying the downstream follow-up for modeled strategies, we may have underestimated the costs and overestimated the benefits relative to the national guidelines, which call for additional follow-up prior to treatment. However, the modeled strategies would likely bias the analysis in favor of Pap and HPV triage strategies. We also did not consider a strategy in which women with ASCUS+ were referred directly to cryotherapy instead of colposcopy because that approach is not recommended by the WHO guidelines,

For peer review only - http://bmjopen.bmj.com/site/about/guidelines.xhtml

or by any professional medical society to the best of our knowledge. We did not consider quality of life impact or the potential disutilities or harms that might be associated with overtreatment.

While we adopted a micro-costing approach to leverage data from the START-UP and Scale-Up projects in Nicaragua, there remain limitations to our cost estimates. First, individual-level data for each woman were not available; thus, our estimates represent average costs in the project populations. Second, we did not have information on the costs associated with HPV self-collection in community settings, where most self-collection takes place. Instead, we assumed all self-collection took place at the clinic. Compared to clinic-based efforts, community-based self-collection may be associated with lower costs for women's time and travel, and higher direct medical and programmatic costs due to outreach worker involvement in facilitating screening and delivering results. Third, our estimates of programmatic costs were restricted to training sessions, and we did not have information on the costs of social mobilization and outreach, patient navigation and support, or infrastructural improvements that would be required to successfully scale-up a screening program. Fourth, we valued women's time based on the minimum wage in Nicaragua. This may be a conservative estimate if most women attending screening are formally employed; conversely, it may overestimate the societal value of women's time spent working in the informal sector or at home. Finally, we extrapolated the cost of cancer treatment using data from El Salvador [29]. Despite these limitations, extensive sensitivity analyses on cost components indicate that HPV-Cryo is robustly the most efficient strategy.

As implementation of HPV testing continues, particularly without triage testing, the health system's capacity to provide cryotherapy will likely need to increase. While the use of triage testing (either with Pap or HPV) reduces the number of cryotherapy procedures performed, we found that the lower sensitivity of triage testing (resulting in more false negatives) led to a decline in health benefits as fewer women with persistent HPV infection and precancer received treatment. The cost savings associated with fewer cryotherapy procedures were outweighed by increased costs of additional follow-

For peer review only - http://bmjopen.bmj.com/site/about/guidelines.xhtml

up and cancer treatment in triage-negative women. A sensitivity analysis on the cost of cryotherapy revealed that HPV-Cryo remained the dominant strategy even when costs increased to 170% of the base case. However, we did not explicitly consider the costs of increasing access to cryotherapy machines or the implications of gas stock-outs, which have been identified as barriers in some low- and middle-income countries [40]. New ablative technologies currently undergoing testing are smaller, portable, and do not require gas. Thermal-coagulation has been used in United Kingdom for more than 30 years, and now it is being used in several low- and middle-income countries, including as part of a "screen-and-treat" program in Malawi [41], and is currently undergoing testing in Latin America. If newer technologies demonstrate cure rates similar to cryotherapy, the cost-effectiveness of screen-and-treat algorithms may improve along with access to treatment.

In summary, using data from the Scale-Up implementation project in Nicaragua, we found that HPV testing followed by cryotherapy for eligible HPV-positive women (a "screen-and-treat" approach) was a very cost-effective intervention in Nicaragua. As the HPV-Cryo algorithm was not implemented in Phase 2 of the Scale-Up project, compliance and cost estimates may need to be further honed to reflect improvements in capacity for cryotherapy if HPV-Cryo is implemented going forward. While it is too early to assume that costs and health impact from Phase 2 of implementation are generalizable to other departments in Nicaragua or other settings in Central America, extensive sensitivity analyses indicate the robustness of findings. An HPV-based screening algorithm involving a similar "screen-and-treat" approach was recently found to be good value for public health dollars in El Salvador [29], where national scale-up is underway. It is important to note that a favorable cost-effectiveness profile does not guarantee that HPV-Cryo will be affordable or feasible in a lower-middle income country like Nicaragua. Both the cost-effectiveness ratio, budgetary impact, and health systems infrastructure need to be favorable for screening programs to be sustainable. We present these findings to inform evidence-based

For peer review only - http://bmjopen.bmj.com/site/about/guidelines.xhtml

decision making around national screening guidelines, program design and implementation, and budgeting for infrastructural improvements and procurement of HPV tests in Nicaragua.

For peer review only

For peer review only - http://bmjopen.bmj.com/site/about/guidelines.xhtml

References

1. Ferlay J, Soerjomataram I, Ervik M, et al. *GLOBOCAN 2012 v1.0, Cancer Incidence and Mortality Worldwide: IARC CancerBase No. 11 [Internet]*, 2013, International Agency for Research on Cancer: Lyon, France.
2. Walboomers JM, Jacobs MV, Manos MM, et al. Human papillomavirus is a necessary cause of invasive cervical cancer worldwide. *J Pathol* 1999;189(1):12-19.
3. Rodriguez AC, Schiffman M, Herrero R, et al. Longitudinal study of human papillomavirus persistence and cervical intraepithelial neoplasia grade 2/3: critical role of duration of infection. *J Natl Cancer Inst* 2010;102(5):315-324.
4. Kitchener HC, Castle PE, and Cox JT. Chapter 7: Achievements and limitations of cervical cytology screening. *Vaccine* 2006;24 Suppl 3:S3/63-70.
5. Denny L, Quinn M, and Sankaranarayanan R. Chapter 8: Screening for cervical cancer in developing countries. *Vaccine* 2006;24 Suppl 3:S3/71-77.
6. ICO. *HPV Information Centre*, 2014.
7. Vastbinder MB, Castillo C, and Bekkers RL. Barriers to follow-up of abnormal Papanicolaou smears among women in Leon, Nicaragua. *Trop Doct* 2010;40(1):22-26.
8. Khan MJ, Castle PE, Lorincz AT, et al. The elevated 10-year risk of cervical precancer and cancer in women with human papillomavirus (HPV) type 16 or 18 and the possible utility of type-specific HPV testing in clinical practice. *J Natl Cancer Inst* 2005;97(14):1072-1079.
9. WHO, in *WHO Guidelines for Screening and Treatment of Precancerous Lesions for Cervical Cancer Prevention*2013: Geneva.
10. Arrossi S, Thouyaret L, Herrero R, et al. Effect of self-collection of HPV DNA offered by community health workers at home visits on uptake of screening for cervical cancer (the EMA study): a population-based cluster-randomised trial. *Lancet Glob Health* 2015;3(2):e85-94.
11. Moses E, Pedersen HN, Mitchell SM, et al. Uptake of community-based, self-collected HPV testing vs. visual inspection with acetic acid for cervical cancer screening in Kampala, Uganda: preliminary results of a randomised controlled trial. *Trop Med Int Health* 2015;20(10):1355-1367.
12. Lazcano-Ponce E, Lorincz AT, Cruz-Valdez A, et al. Self-collection of vaginal specimens for human papillomavirus testing in cervical cancer prevention (MARCH): a community-based randomised controlled trial. *Lancet* 2011;378(9806):1868-1873.
13. Racey CS, Withrow DR, and Gesink D. Self-collected HPV testing improves participation in cervical cancer screening: a systematic review and meta-analysis. *Can J Public Health* 2013;104(2):e159-166.
14. Jeronimo J, Bansil P, Lim J, et al. A multicountry evaluation of careHPV testing, visual inspection with acetic acid, and papanicolaou testing for the detection of cervical cancer. *Int J Gynecol Cancer* 2014;24(3):576-585.
15. Qiao YL, Sellors JW, Eder PS, et al. A new HPV-DNA test for cervical-cancer screening in developing regions: a cross-sectional study of clinical accuracy in rural China. *Lancet Oncol* 2008;9(10):929-936.
16. Jeronimo J, Holme F, Slavkovsky R, et al. Implementation of HPV testing in Latin America. *J Clin Virol* 2016;76 Suppl 1:S69-73.
17. Campos NG, Tsu, V., Jeronimo, J., Mvundura, M., Lee, K., Kim, J.J. When and how often to screen for cervical cancer in three low- and middle-income countries: A cost-effectiveness analysis. *Papillomavirus Research* 2015;1:38-58.

For peer review only - http://bmjopen.bmj.com/site/about/guidelines.xhtml

18. Campos NG, Burger EA, Sy S, et al. An updated natural history model of cervical cancer: derivation of model parameters. *Am J Epidemiol* 2014;180(5):545-555.

19. WHO. *Macroeconomics and health: Investing in health for economic development: Report of the Commission on Macroeconomics and Health*, 2001, World Health Organization: Geneva.

20. Tan-Torres Edejer T, Baltussen, R., Adam, T., Hutubessy, R., Acharya, A., Evans, D.B., Murray, C.J.L., ed. *Making Choices in Health: WHO Guide to Cost-Effectiveness Analysis*. 2003, World Health Organization: Geneva, Switzerland.

21. Jamison DT, Breman, J.G., Measham, A.R., Alleyne, G., Claeson, M., Evans, D.B., Jha, P., Mills, A., Musgrove, P., ed. *Disease Control Priorities in Developing Countries*. Second edition ed. 2006, Oxford University Press and The World Bank: Washington, D.C.

22. Munoz N, Mendez F, Posso H, et al. Incidence, duration, and determinants of cervical human papillomavirus infection in a cohort of Colombian women with normal cytological results. *J Infect Dis* 2004;190(12):2077-2087.

23. Herrero R, Hildesheim A, Rodriguez AC, et al. Rationale and design of a community-based double-blind randomized clinical trial of an HPV 16 and 18 vaccine in Guanacaste, Costa Rica. *Vaccine* 2008;26(37):4795-4808.

24. Surveillance, Epidemiology, and End Results (SEER) Program. *SEER*Stat Database: Incidence - SEER 18 Regs Research Data + Hurricane Katrina Impacted Louisiana Cases Nov 2011 Sub (1973-2009 varying) – Linked to County Attributes – Total U.S., 1969-2010 Counties*. Bethesda, MD: National Cancer Institute, Division of Cancer Control and Population Sciences, SUrveillance Research Program, Surveillance Systems Branch; 2012.

25. Sankaranarayanan R, Swaminathan R, Brenner H, et al. Cancer survival in Africa, Asia, and Central America: a population-based study. *Lancet Oncol* 2010;11(2):165-173.

26. Stinnett AA and Paltiel AD. Estimating CE ratios under second-order uncertainty: the mean ratio versus the ratio of means. *Med Decis Making* 1997;17(4):483-489.

27. Mayrand MH, Duarte-Franco E, Rodrigues I, et al. Human papillomavirus DNA versus Papanicolaou screening tests for cervical cancer. *N Engl J Med* 2007;357(16):1579-1588.

28. Ronco G, Dillner J, Elfstrom KM, et al. Efficacy of HPV-based screening for prevention of invasive cervical cancer: follow-up of four European randomised controlled trials. *Lancet* 2014;383(9916):524-532.

29. Campos NG, Maza M, Alfaro K, et al. The comparative and cost-effectiveness of HPV-based cervical cancer screening algorithms in El Salvador. *Int J Cancer* 2015.

30. Chirenje ZM, Rusakaniko S, Kirumbi L, et al. Situation analysis for cervical cancer diagnosis and treatment in east, central and southern African countries. *Bull World Health Organ* 2001;79(2):127-132.

31. Cremer M, Bullard K, Maza M, et al. Cytology versus visual inspection with acetic acid among women treated previously with cryotherapy in a low-resource setting. *Int J Gynaecol Obstet* 2010;111(3):249-252.

32. Sauvaget C, Muwonge R, and Sankaranarayanan R. Meta-analysis of the effectiveness of cryotherapy in the treatment of cervical intraepithelial neoplasia. *Int J Gynaecol Obstet* 2013;120(3):218-223.

33. Bigoni J, Gundar M, Tebeu PM, et al. Cervical cancer screening in sub-Saharan Africa: a randomized trial of VIA versus cytology for triage of HPV-positive women. *Int J Cancer* 2015;137(1):127-134.

34. Catarino R, Vassilakos P, Scaringella S, et al. Smartphone Use for Cervical Cancer Screening in Low-Resource Countries: A Pilot Study Conducted in Madagascar. *PLoS One* 2015;10(7):e0134309.

For peer review only - http://bmjopen.bmj.com/site/about/guidelines.xhtml

35. Pan QJ, Hu SY, Guo HQ, et al. Liquid-based cytology and human papillomavirus testing: a pooled analysis using the data from 13 population-based cervical cancer screening studies from China. *Gynecol Oncol* 2014;133(2):172-179.

36. Qiao YL, Jeronimo J, Zhao FH, et al. Lower cost strategies for triage of human papillomavirus DNA-positive women. *Int J Cancer* 2014;134(12):2891-2901.

37. Tebeu PM, Fokom-Domgue J, Crofts V, et al. Effectiveness of a two-stage strategy with HPV testing followed by visual inspection with acetic acid for cervical cancer screening in a low-income setting. *Int J Cancer* 2015;136(6):E743-750.

38. Richardson LA, El-Zein M, Ramanakumar AV, et al. HPV DNA testing with cytology triage in cervical cancer screening: Influence of revealing HPV infection status. *Cancer Cytopathol* 2015;123(12):745-754.

39. *World Development Indicators*, 2016, World Bank.

40. Msyamboza KP, Phiri T, Sichali W, et al. Cervical cancer screening uptake and challenges in Malawi from 2011 to 2015: retrospective cohort study. *BMC Public Health* 2016;16(1):806.

41. Campbell C, Kafwafwa S, Brown H, et al. Use of thermo-coagulation as an alternative treatment modality in a 'screen and treat' programme of cervical screening in rural Malawi. *Int J Cancer* 2016.

42. Gage JC, Rodriguez AC, Schiffman M, et al. Treatability by cryotherapy in a screen-and-treat strategy. *J Low Genit Tract Dis* 2009;13(3):174-181.

43. Starks D, Arriba LN, Enerson CL, et al. Mexican Cervical Cancer Screening Study II: 6-month and 2-year follow-up of HR-HPV women treated with cryotherapy in a low-resource setting. *J Low Genit Tract Dis* 2014;18(4):333-337.

44. McClung EC and Blumenthal PD. Efficacy, safety, acceptability and affordability of cryotherapy: a review of current literature. *Minerva Ginecol* 2012;64(2):149-171.

For peer review only

For peer review only - http://bmjopen.bmj.com/site/about/guidelines.xhtml

Table 1. Baseline values and ranges for model variables.[a]

Test/Parameter	Base Case:	Sensitivity Analysis:
Screening/triage test performance (sensitivity/specificity to detect CIN2+)		
Pap (primary) [14, 27, 28]	0.41/0.94	Alternative sensitivity/specificity pair: 0.70/0.95
careHPV (primary), provider-collection of cervical samples **[14]**	0.78/0.89	--
careHPV (primary), self-collection of vaginal samples **[14]**	0.67/0.86	--
VIA (triage of HPV+) **[14, 33, 34, 36, 37]**	0.60/0.75	Alternative sensitivity/specificity pairs: 0.40/0.85 0.70/0.65
Pap (triage of HPV+) **[14, 33, 35, 38]**	0.85/0.55	Alternative sensitivity/specificity pairs: 0.40/0.85 0.90/0.50
Colposcopy performance (sensitivity/specificity to detect CIN1+) **[14, 17]**[b]		
Colposcopy (ASCUS+ women)	0.95/0.68	1.0/1.0
Colposcopy (HPV+ women ineligible for cryotherapy)	Upon ruling out cancer, all referred to treatment	0.95/0.68, with <CIN1 sent back to routine screening intervals
Colposcopy (HPV+/VIA+ women ineligible for ST cryotherapy)	Upon ruling out cancer, all referred to treatment	0.95/0.68, with <CIN1 sent back to routine screening intervals
Colposcopy (HPV+/Pap+ women)	Upon ruling out cancer, all referred to treatment	0.95/0.68, with <CIN1 sent back to routine screening intervals
Coverage and compliance[c]		
Access to routine screening, % of the target population **[6]**	70%	50% - 80%
Visit compliance, screening facility[d]	85%	40% - 85%
Visit compliance, referral facility[d]	40%	40% - 85%
Treatment eligibility and efficacy		
Eligibility for screen-and-treat cryotherapy **[29, 42]**	≤CIN1: 100% CIN2: 85%	≤CIN1: 75% CIN2: 60%

For peer review only

For peer review only - http://bmjopen.bmj.com/site/about/guidelines.xhtml

	CIN3: 75%	CIN3: 49%
Screen-and-treat cryotherapy cure rate (for HPV+ or HPV+/VIA+) **[30-32]**	92%	75%
Proportion of women maintaining an HPV infection following cryotherapy **[43]**	15%	--
Treatment cure rate following colposcopy (LEEP for CIN2+; else cryotherapy)**[30]**	96%	85%
Proportion of women maintaining an HPV infection following colposcopic diagnosis and treatment **[44]**	10%	--
Discount rate for costs and life-years	3%	0% - 5%
Direct medical costs, screening and treatment of precancer (2015 US$)**[14]**[d]		
Pap test	7.26	3
careHPV test (provider-collection)	11.96	--
careHPV test (self-collection)	11.04	75% - 125% of base case
VIA triage test	4.19	--
Colposcopy/biopsy	19.91	6.91[e]
Cryotherapy	18.16	30.54[f]
LEEP	68.36	75% - 125% of base case
Women's time and transportation costs (2015 US$)[d]		
Transportation to screening facility (round-trip)	0.41	0% to 50% of base case
Transportation to referral facility (round trip)	2.81	0% to 50% of base case
Wait time, screening facility	0.48	0% to 50% of base case
Wait time, referral facility	1.75	0% to 50% of base case
Transport time, screening facility	0.82	0% to 50% of base case
Transport time, referral facility	3.10	0% to 50% of base case
Programmatic costs, training (2015 US$)[d]		
Health care personnel (careHPV)	0.09 per woman screened with careHPV	50% - 150% of base case
Laboratory technicians (careHPV)	0.04 per woman screened with careHPV	50% - 150% of base case
Outreach workers/auxiliary nurses (careHPV self-collection)	0.08 per woman screened with careHPV (self-collection)	50% - 150% of base case
Health care providers (VIA and cryotherapy)	1.51 per woman receiving VIA and/or cryotherapy	50% - 150% of base case

For peer review only

For peer review only - http://bmjopen.bmj.com/site/about/guidelines.xhtml

Cost of cancer treatment (2015 US$) (rounded) **[29]**		
Local cancer		
- Direct medical	944	
- Direct non-medical[g]	197	
- Women's time[h]	346	
Total	1,486	944 - 2,229 (direct medical only; 150% of base case)
Regional and distant cancer		
- Direct medical	918	
- Direct non-medical[g]	390	
- Women's time[h]	640	
Total	1,946	918 - 2,920 (direct medical only; 150% of base case)

[a] ASCUS+: atypical squamous cells of undetermined significance or higher; CIN 1+: cervical intraepithelial neoplasia grade 1 or higher; CIN2+: cervical intraepithelial neoplasia grade 2 or higher; HPV: human papillomavirus; LEEP: loop electrosurgical excision procedure; ST: screen-and-treat; US$: 2015 United States dollars; VIA: visual inspection with acetic acid. Parameter values for sensitivity analysis were determined as follows: screening and triage test performance (cited literature); colposcopy performance (assumption); coverage and compliance (assumptions); treatment eligibility and efficacy (cited literature); discount rate (assumptions); direct medical costs (assumptions, with the exception of colposcopy and cryotherapy, which were based on ranges suggested by Scale-Up and START-UP data); women's costs (assumptions); programmatic costsw (assumptions); cancer costs (assumptions).

[b] Test performance characteristics of colposcopy in the START-UP demonstration project were derived from the worst diagnosis of the local pathologist relative to the worst diagnosis by a quality control pathologist (gold standard); we applied the treatment threshold of CIN1+, although this was not the treatment threshold in START-UP. To derive test performance of colposcopy, we excluded histological classifications that were inadequate or with a histological classification other than negative, CIN1, CIN2, CIN3, or cancer. Because CIN1 is not a true underlying health state in the microsimulation model, performance of colposcopy in the model is based on the underlying health states of no lesion, HPV infection, CIN2, or CIN3. For a treatment threshold of CIN1, we weighted sensitivity of colposcopy for women with HPV based on the country-specific prevalence of CIN1 among women with HPV infections in the START-UP studies.

[c] Compliance is defined as the proportion of women who return for each clinical encounter, relative to the previous visit.

[d] Unpublished data from the Scale-Up Nicaragua project. Further details on costing data are provided in the Appendix.

[e] In sensitivity analysis, we considered the direct medical cost of colposcopy to be equivalent to the cost of colposcopy alone, without biopsy.

[f] In sensitivity analysis, we considered the direct medical cost of cryotherapy to include the upper bound of cryotherapy equipment costs (assuming the lowest number of women treated per year per facility, in Carazo, Chontales, or Chinandega) and the upper bound of cryotherapy supply costs (assuming the lowest number of women treated per gas tank in any facility in Carazo, Chontales, or Chinandega).

[g] Direct non-medical costs include transportation to a tertiary facility, temporary housing, and meals.

[h] Includes woman's time an support person's time.

For peer review only

Figure legends.

Figure 1. Pathways of care, by screening strategy. Each diagram indicates the flow of screening-eligible women (i.e., women aged 30 to 59 years) through each point of contact in a screening episode, conditional on visit compliance and test results, for a) Pap testing every 3 years (Pap), which requires 4 visits for screening, diagnosis, and treatment; b) HPV screen-and-treat every 5 years (HPV-Cryo), which requires 3 or more visits for screening and necessary treatment; c) HPV testing followed by visual inspection with acetic acid (VIA) triage of HPV-positive women every 5 years (HPV-VIA), which requires 3 or more visits for screening and necessary treatment; and d) HPV testing followed by Pap triage of HPV-positive women every 5 years (HPV-Pap), which requires 5 or more visits for screening and necessary treatment. ASCUS+: Pap result of atypical squamous cells of undetermined significance or worse; CIN: cervical intraepithelial neoplasia; HPV: human papillomavirus; LEEP: loop electrosurgical excision procedure; VIA: visual inspection with acetic acid.

Figure 2. Cervical cancer screening cost per woman over duration of screening eligibility, by cost component: Pap testing (every 3 years) versus careHPV testing (every 5 years). Bars indicate the undiscounted cost (2015 US$) of screening with Pap testing (offered 10 times between ages 30 and 59 years) versus careHPV testing (offered 6 times between ages 30 and 59 years), by cost component. Only screening costs are shown; costs associated with recommended management following a positive screening test are not included. 6x: delivered 6 times over the course of screening eligible ages 30 to 59; 10x: delivered 10 times over the course of screening eligible ages.

Figure 3. Cost-effectiveness analysis: Base case results. The graph displays the discounted lifetime costs (x-axis; in 2015 US$) and life expectancy (y-axis) associated with each screening strategy (Pap testing every 3 years; careHPV screen-and-treat every 5 years [HPV-Cryo]; careHPV every 5 years with visual inspection with acetic acid [VIA] triage of HPV-positive women [HPV-VIA]; and careHPV every 5 years with Pap triage of HPV-positive women [HPV-Pap]), under base case assumptions. The cost-effectiveness

For peer review only - http://bmjopen.bmj.com/site/about/guidelines.xhtml

associated with a change from one strategy to a more costly alternative is represented by the difference in cost divided by the difference in life expectancy associated with the two strategies. The curve indicates the strategies that are efficient because they are more effective and either 1) cost less; or 2) have a more attractive cost-effectiveness ratio than less effective options. The incremental cost-effectiveness ratio (ICER) is the reciprocal of the slope of the line connecting the two strategies under comparison. In the base case analysis, HPV-Cryo every 5 years was less costly and more effective than other screening strategies considered, and was thus a dominant strategy with an ICER of US$320 per year of life saved. HPV-Pap: HPV testing with Pap triage of HPV-positive women; HPV-Cryo: HPV screen-and-treat strategy; HPV-VIA: HPV testing with visual inspection with acetic acid (VIA) triage of HPV-positive women; yrs: years.

Figure 4. Reduction in lifetime risk of cervical cancer, by compliance level. Bars indicate the percent reduction in lifetime risk of cervical cancer for each screening strategy (Pap testing every 3 years; careHPV screen-and-treat every 5 years [HPV-Cryo]; careHPV every 5 years with visual inspection with acetic acid [VIA] triage of HPV-positive women [HPV-VIA]; and careHPV every 5 years with Pap triage of HPV-positive women [HPV-Pap]) as compliance per visit within a screening episode increases. Compliance is defined as the proportion of women who return for each clinical encounter, relative to the previous visit. Coverage of the target population is assumed to be 70%. While the base case analysis assumed 85% compliance for visits at screening facilities and 40% compliance for visits at referral facilities (for diagnosis and treatment), the graph displays cancer risk reduction assuming the specified compliance level at all visits, regardless of facility type.

Figure 5. Base case and sensitivity analysis: Incremental cost-effectiveness ratios, HPV cryotherapy (HPV-Cryo) strategy. Incremental cost-effectiveness ratios (ICERs) are presented (x-axis, 2015 US$ per year of life saved) for the base case and sensitivity analyses (y-axis). The blue bars represent the range of the ICERs for HPV-Cryo every 5 years across the 50 input parameter sets, with the ICER of the mean

For peer review only - http://bmjopen.bmj.com/site/about/guidelines.xhtml

costs divided by the mean effects demarcated by a black line. The dashed blue line indicates Nicaragua's per capita gross domestic product (GDP), at US$2,090, assuming this is the threshold that designates interventions as "very cost-effective".

For peer review only

For peer review only - http://bmjopen.bmj.com/site/about/guidelines.xhtml

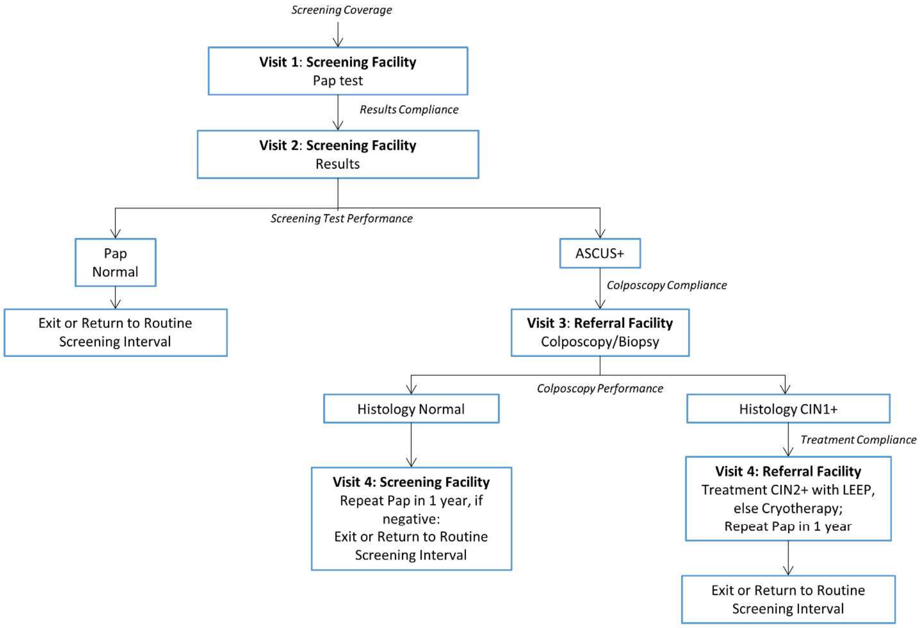

Figure 1. Pathways of care, by screening strategy. Each diagram indicates the flow of screening-eligible women (i.e., women aged 30 to 59 years) through each point of contact in a screening episode, conditional on visit compliance and test results, for a) Pap testing every 3 years (Pap), which requires 4 visits for screening, diagnosis, and treatment; b) HPV screen-and-treat every 5 years (HPV-STHPV-Cryo), which requires 3 or more visits for screening and necessary treatment; c) HPV testing followed by visual inspection with acetic acid (VIA) triage of HPV-positive women every 5 years (HPV-VIA), which requires 3 or more visits for screening and necessary treatment; and d) HPV testing followed by Pap triage of HPV-positive women every 5 years (HPV-Pap), which requires 5 or more visits for screening and necessary treatment. ASCUS+: Pap result of atypical squamous cells of undetermined significance or worse; CIN: cervical intraepithelial neoplasia; HPV: human papillomavirus; LEEP: loop electrosurgical excision procedure; VIA: visual inspection with acetic acid.

254x190mm (300 x 300 DPI)

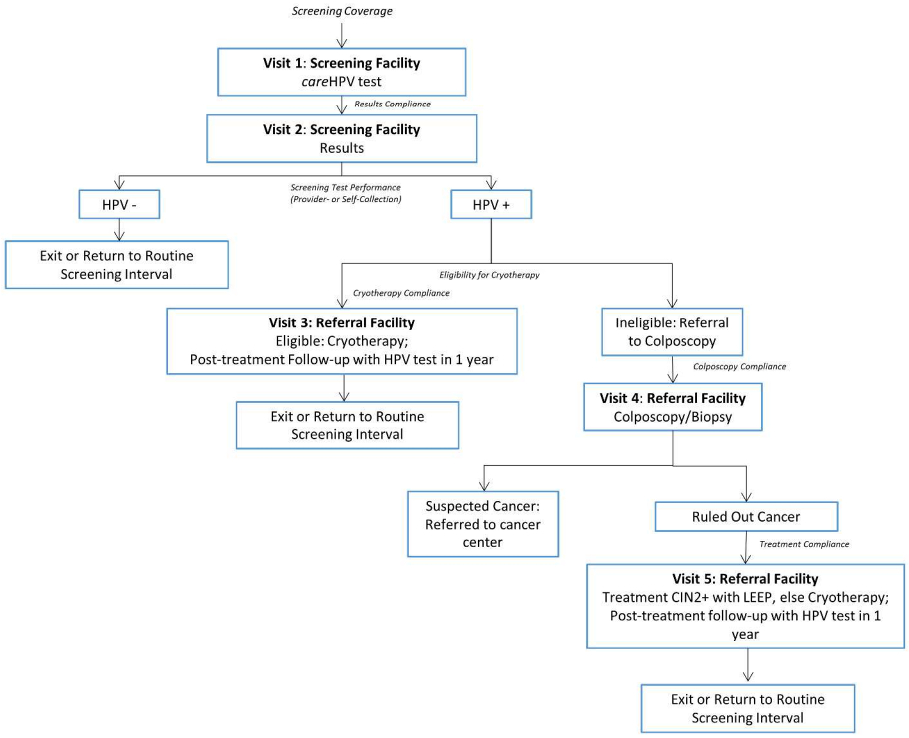

Figure 1. Pathways of care, by screening strategy. Each diagram indicates the flow of screening-eligible women (i.e., women aged 30 to 59 years) through each point of contact in a screening episode, conditional on visit compliance and test results, for a) Pap testing every 3 years (Pap), which requires 4 visits for screening, diagnosis, and treatment; b) HPV screen-and-treat every 5 years (HPV-Cryo), which requires 3 or more visits for screening and necessary treatment; c) HPV testing followed by visual inspection with acetic acid (VIA) triage of HPV-positive women every 5 years (HPV-VIA), which requires 3 or more visits for screening and necessary treatment; and d) HPV testing followed by Pap triage of HPV-positive women every 5 years (HPV-Pap), which requires 5 or more visits for screening and necessary treatment. ASCUS+: Pap result of atypical squamous cells of undetermined significance or worse; CIN: cervical intraepithelial neoplasia; HPV: human papillomavirus; LEEP: loop electrosurgical excision procedure; VIA: visual inspection with acetic acid.

254x190mm (300 x 300 DPI)

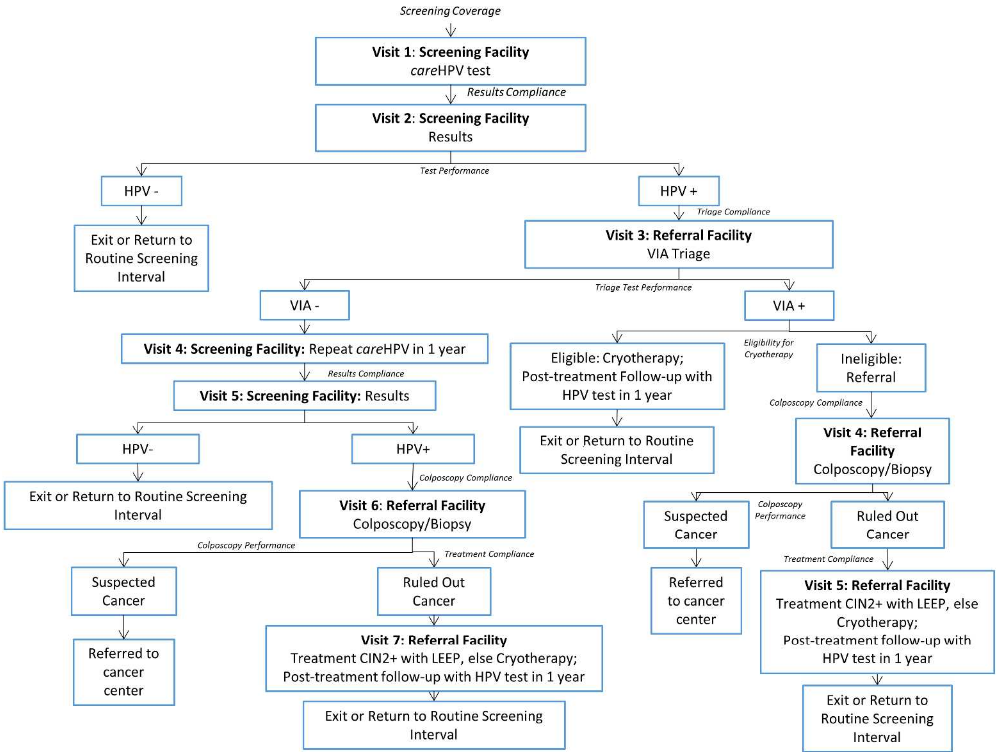

Figure 1. Pathways of care, by screening strategy. Each diagram indicates the flow of screening-eligible women (i.e., women aged 30 to 59 years) through each point of contact in a screening episode, conditional on visit compliance and test results, for a) Pap testing every 3 years (Pap), which requires 4 visits for screening, diagnosis, and treatment; b) HPV screen-and-treat every 5 years (HPV-Cryo), which requires 3 or more visits for screening and necessary treatment; c) HPV testing followed by visual inspection with acetic acid (VIA) triage of HPV-positive women every 5 years (HPV-VIA), which requires 3 or more visits for screening and necessary treatment; and d) HPV testing followed by Pap triage of HPV-positive women every 5 years (HPV-Pap), which requires 5 or more visits for screening and necessary treatment. ASCUS+: Pap result of atypical squamous cells of undetermined significance or worse; CIN: cervical intraepithelial neoplasia; HPV: human papillomavirus; LEEP: loop electrosurgical excision procedure; VIA: visual inspection with acetic acid.

254x190mm (300 x 300 DPI)

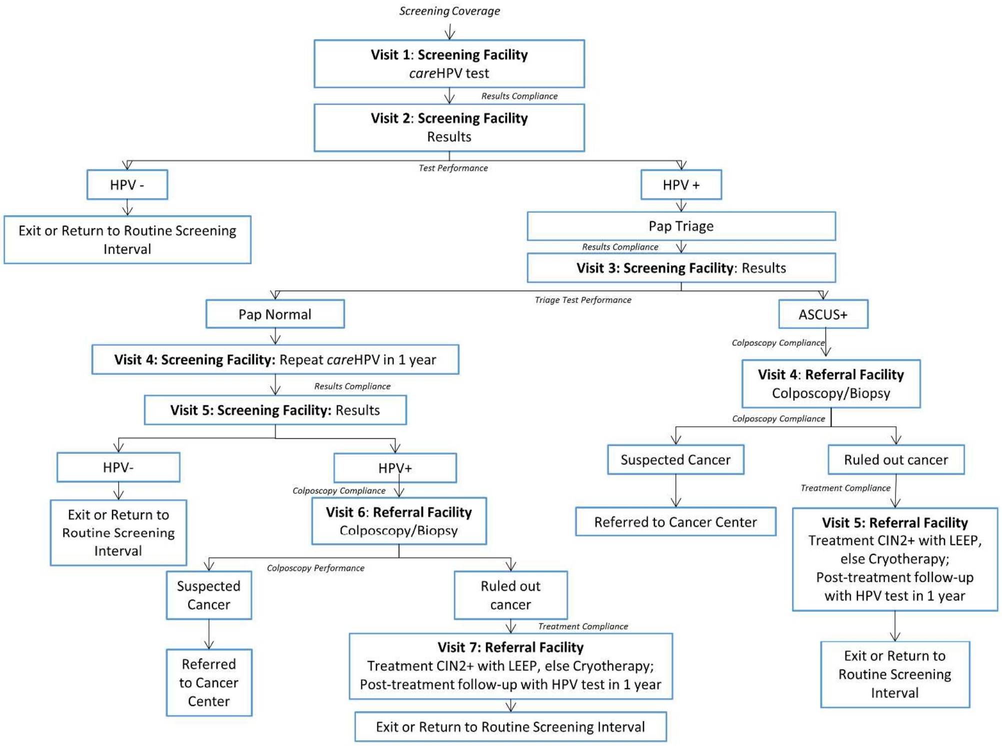

Figure 1. Pathways of care, by screening strategy. Each diagram indicates the flow of screening-eligible women (i.e., women aged 30 to 59 years) through each point of contact in a screening episode, conditional on visit compliance and test results, for a) Pap testing every 3 years (Pap), which requires 4 visits for screening, diagnosis, and treatment; b) HPV screen-and-treat every 5 years (HPV-Cryo), which requires 3 or more visits for screening and necessary treatment; c) HPV testing followed by visual inspection with acetic acid (VIA) triage of HPV-positive women every 5 years (HPV-VIA), which requires 3 or more visits for screening and necessary treatment; and d) HPV testing followed by Pap triage of HPV-positive women every 5 years (HPV-Pap), which requires 5 or more visits for screening and necessary treatment. ASCUS+: Pap result of atypical squamous cells of undetermined significance or worse; CIN: cervical intraepithelial neoplasia; HPV: human papillomavirus; LEEP: loop electrosurgical excision procedure; VIA: visual inspection with acetic acid.

254x190mm (300 x 300 DPI)

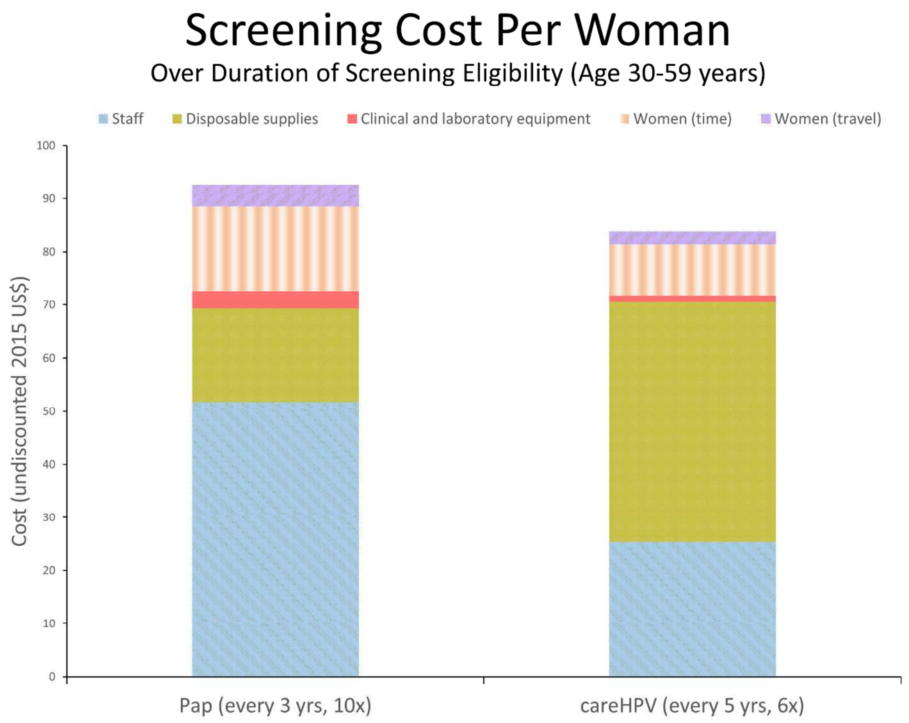

Figure 2. Cervical cancer screening cost per woman over duration of screening eligibility, by cost component: Pap testing (every 3 years) versus careHPV testing (every 5 years). Bars indicate the undiscounted cost (2015 US$) of screening with Pap testing (offered 10 times between ages 30 and 59 years) versus careHPV testing (offered 6 times between ages 30 and 59 years), by cost component. Only screening costs are shown; costs associated with recommended management following a positive screening test are not included. 6x: delivered 6 times over the course of screening eligible ages 30 to 59; 10x: delivered 10 times over the course of screening eligible ages.

254x190mm (300 x 300 DPI)

only

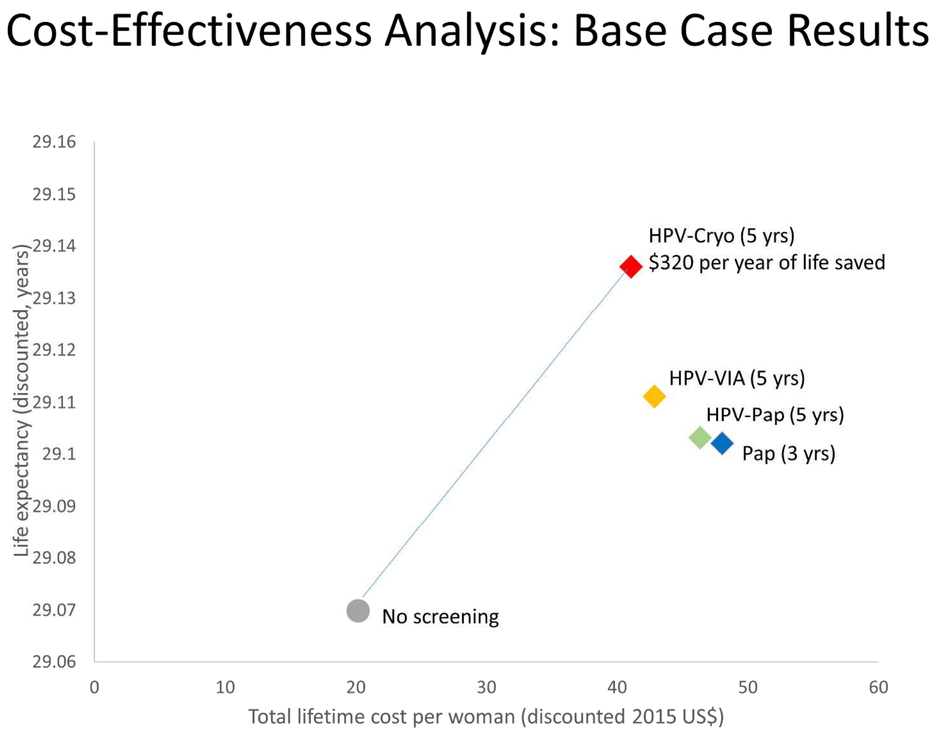

Figure 3. Cost-effectiveness analysis: Base case results. The graph displays the discounted lifetime costs (x-axis; in 2015 US$) and life expectancy (y-axis) associated with each screening strategy (Pap testing every 3 years; careHPV screen-and-treat every 5 years [HPV-Cryo]; careHPV every 5 years with visual inspection with acetic acid [VIA] triage of HPV-positive women [HPV-VIA]; and careHPV every 5 years with Pap triage of HPV-positive women [HPV-Pap]), under base case assumptions. The cost-effectiveness associated with a change from one strategy to a more costly alternative is represented by the difference in cost divided by the difference in life expectancy associated with the two strategies. The curve indicates the strategies that are efficient because they are more effective and either 1) cost less; or 2) have a more attractive cost-effectiveness ratio than less effective options. The incremental cost-effectiveness ratio (ICER) is the reciprocal of the slope of the line connecting the two strategies under comparison. In the base case analysis, HPV-Cryo every 5 years was less costly and more effective than other screening strategies considered, and was thus a dominant strategy with an ICER of US$320 per year of life saved. HPV-Pap: HPV testing with Pap triage of HPV-positive women; HPV-Cryo: HPV screen-and-treat strategy; HPV-VIA: HPV testing with visual inspection with acetic acid (VIA) triage of HPV-positive women; yrs: years.

254x190mm (300 x 300 DPI)

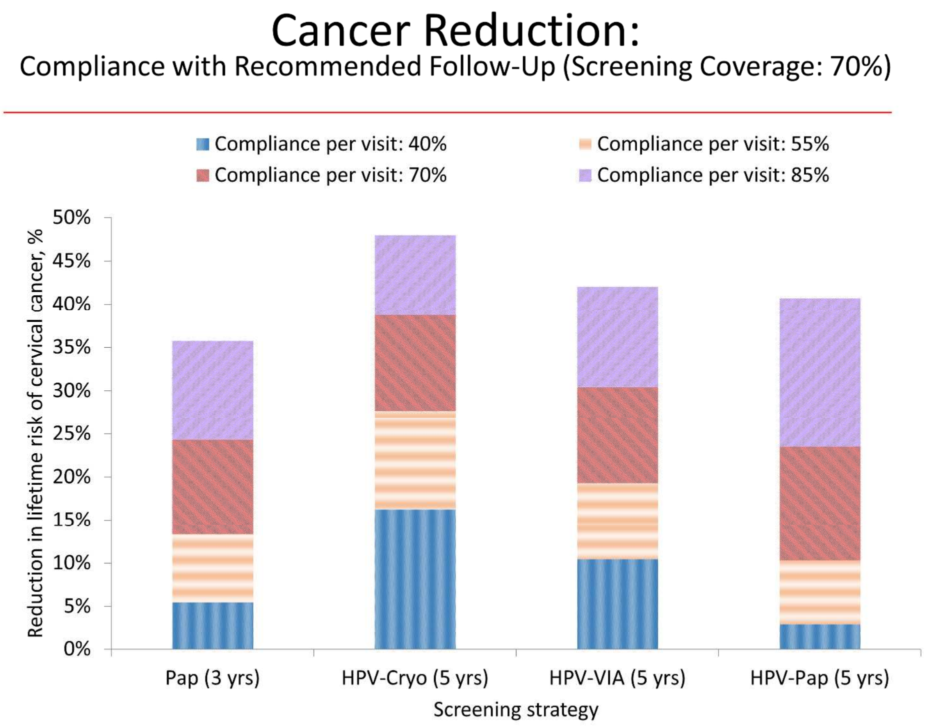

Figure 4. Reduction in lifetime risk of cervical cancer, by compliance level. Bars indicate the percent reduction in lifetime risk of cervical cancer for each screening strategy (Pap testing every 3 years; careHPV screen-and-treat every 5 years [HPV-Cryo]; careHPV every 5 years with visual inspection with acetic acid [VIA] triage of HPV-positive women [HPV-VIA]; and careHPV every 5 years with Pap triage of HPV-positive women [HPV-Pap]) as compliance per visit within a screening episode increases. Compliance is defined as the proportion of women who return for each clinical encounter, relative to the previous visit. Coverage of the target population is assumed to be 70%. While the base case analysis assumed 85% compliance for visits at screening facilities and 40% compliance for visits at referral facilities (for diagnosis and treatment), the graph displays cancer risk reduction assuming the specified compliance level at all visits, regardless of facility type.

254x190mm (300 x 300 DPI)

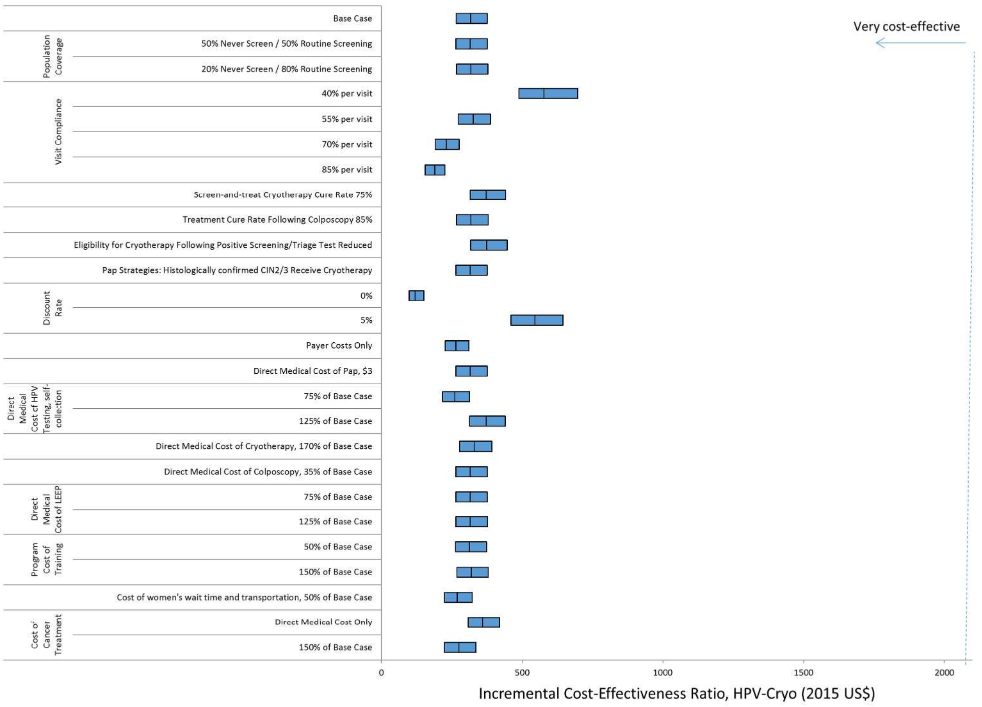

Figure 5. Base case and sensitivity analysis: Incremental cost-effectiveness ratios, HPV cryotherapy (HPV-Cryo) strategy. Incremental cost-effectiveness ratios (ICERs) are presented (x-axis, 2015 US$ per year of life saved) for the base case and sensitivity analyses (y-axis). The blue bars represent the range of the ICERs for HPV-Cryo every 5 years across the 50 input parameter sets, with the ICER of the mean costs divided by the mean effects demarcated by a black line. The dashed blue line indicates Nicaragua's per capita gross domestic product (GDP), at US$2,090, assuming this is the threshold that designates interventions as "very cost-effective".

254x190mm (300 x 300 DPI)

only

CHEERS Checklist

Section/topic	#	Recommendation	Reported on page #/ line #
**TITLE AND ABSTRACT**			
Title	1	Identify the study as an economic evaluation or use more specific terms such as “cost-effectiveness analysis”, and describe the interventions compared.	1/1
Abstract	2	Provide a structured summary of objectives, perspective, setting, methods (including study design and inputs), results (including base case and uncertainty analyses), and conclusions.	2/18-42
Abstract			
**INTRODUCTION**			
Background and objectives	3	Provide an explicit statement of the broader context for the study.<br>Present the study question and its relevance for health policy or practice decisions.	7/112
**METHODS**			
Target population and subgroups	4	Describe characteristics of the base case population and subgroups analysed, including why they were chosen.	7/119; 8/126
Setting and location	5	State relevant aspects of the system(s) in which the decision(s) need(s) to be made.	7/119; 8/126
Study perspective	6	Describe the perspective of the study and relate this to the costs being evaluated.	8/137
Comparators	7	Describe the interventions or strategies being compared and state why they were chosen.	10/170-190
Time horizon	8	State the time horizon(s) over which costs and consequences are being evaluated and say why appropriate.	8/129
Discount rate	9	Report the choice of discount rate(s) used for costs and outcomes and say why appropriate.	8/139
Choice of health outcomes	10	Describe what outcomes were used as the measure(s) of benefit in the evaluation and their relevance for the type of analysis performed.	8/129
Measurement of effectiveness	11a	*Single study-based estimates*: Describe fully the design features of the single effectiveness study and why the single study was a sufficient source of clinical effectiveness data	24/527
	11b	*Synthesis-based estimates*: Describe fully the methods used for identification of included studies and synthesis of clinical effectiveness data.	24/527
Measurement and valuation of preference based	12	If applicable, describe the population and methods used to elicit preferences for outcomes	NA

For peer review only - http://bmjopen.bmj.com/site/about/guidelines.xhtml

For peer review only

CHEERS Checklist

outcomes			

Section/topic	#	Checklist item	Reported on page #
Estimating resources and costs	13a	*Single study-based economic evaluation*: Describe approaches used to estimate resource use associated with the alternative interventions. Describe primary or secondary research methods for valuing each resource item in terms of its unit cost. Describe any adjustments made to approximate to opportunity costs.	NA
	13b	*Model-based economic evaluation*: Describe approaches and data sources used to estimate resource use associated with model health states. Describe primary or secondary research methods for valuing each resource item in terms of its unit cost. Describe any adjustments made to approximate to opportunity costs.	Appendix, p.2-4/ 58-155
Currency, price date, and conversion	14	Report the dates of the estimated resource quantities and unit costs. Describe methods for adjusting estimated unit costs to the year of reported costs if necessary. Describe methods for converting costs into a common currency base and the exchange rate.	Appendix, p.2/ 63-68
Choice of model	15	Describe and give reasons for the specific type of decision-analytical model used. Providing a figure to show model structure is strongly recommended.	8/143-167
Assumptions	16	Describe all structural or other assumptions underpinning the decision-analytical model.	8/143; Appendix p. 6/172
Analytical models	17	Describe all analytical methods supporting the evaluation. This could include methods for dealing with skewed, missing, or censored data; extrapolation methods; methods for pooling data; approaches to validate or make adjustments (such as half cycle corrections) to a model; and methods for handling population heterogeneity and uncertainty.	9/154-167 Appendix p. 1-4
**RESULTS**			
Study parameters	18	Report the values, ranges, references, and, if used, probability distributions for all parameters/ Report reasons or sources for distributions used to represent uncertainty where appropriate. Providing a table to show the input values is strongly recommended.	24/527-549
Incremental costs and outcomes	19	For each intervention, report mean values for the main categories of estimated costs and outcomes of interest, as well as mean differences between the comparator groups. If applicable, report incremental cost-effectiveness ratios.	27/569
Characterising uncertainty	20a	*Single study-based economic evaluation*: Describe the effects of sampling uncertainty for the estimated incremental cost and incremental effectiveness parameters, together with the impact of methodological assumptions (such as discount rate, study perspective).	NA
	20b	*Model-based economic evaluation*: Describe the effects on the results of uncertainty for all input parameters, and uncertainty related to the structure of the model and assumptions.	13-14/ 257-299

For peer review only

CHEERS Checklist

Section/topic	#	Checklist item	Reported on page #
Characterising heterogeneity	21	If applicable, report differences in costs, outcomes, or cost-effectiveness that can be explained by variations between subgroups of patients with different baseline characteristics or other observed variability in effects that are not reducible by more information.	NA
**Section/topic**	**#**	**Checklist item**	**Reported on page #**
**DISCUSSION**			
Study findings, limiations, generalisability, and current knowledge	22	Summarise key study findings and describe how they support the conclusions reached. Discuss limitations and the generalisability of the findings and how the findings fit with current knowledge.	16-20/ 312-407
**Other**			
Source of funding	23	Describe how the study was funded and the role of the funder in the identification, design, conduct, and reporting of the analysis. Describe other non-monetary sources of support.	4/ 57-62
Conflicts of interest	24	Describe any potential for conflict of interest of study contributors in accordance with journal policy. Int eh absence of a journal policy, we recommend authors comply with International Committee of Medical Journal Editors recommendations.	4/ 63-67

For peer review only

Supplemental Appendix:
The Cost-Effectiveness of HPV-Based Screening in the Public Health System in Nicaragua

MODEL CALIBRATION

Overview of the calibration process

Details of model development for the U.S. and Nicaragua models, including initial parameterization and calibration, have been published elsewhere [1, 2]. Baseline HPV incidence rates, as a function of genotype and age, were derived from a published prospective study of sexually active women aged 15-85 years in Bogota, Colombia [3]. Because HPV incidence is not necessarily similar in Colombia and Nicaragua due to differences in sexual behavior, we considered age-specific HPV incidence and natural immunity following initial infection as candidate parameters for calibration. Type-specific data on CIN2 and CIN3 regression and progression are limited [4-9], so these highly uncertain parameters were also candidates for calibration. Time-dependent rates of HPV clearance and progression by genotype were informed by primary longitudinal data from the control arm of the Costa Rica Vaccine Trial [10], assuming that rates of HPV clearance and progression to precancer are similar across populations and vary only by time since infection and HPV genotype.

To calibrate the model, we selected empirical epidemiologic data from Nicaragua, including age-specific prevalence of high-risk HPV and age-specific cancer incidence (see below for further detail). We then set plausible search ranges around baseline model input values for age- and type-specific HPV incidence; natural immunity following initial infection; and progression and regression of CIN. We performed repeated model simulations in the absence of any preventive intervention (i.e., natural history simulations). For each simulation, we randomly selected a single value for each of the uncertain parameters from the identified search range, creating a unique vector of parameter values (i.e., parameter "set"). From over 1,475,000 repeated samplings, we identified the parameter sets with the highest correspondence to the empirical calibration target data from Nicaragua by calculating and aggregating the log-likelihood of model-projected outcomes. We used the 50 parameter sets with the highest likelihood scores (i.e., best overall fit to the empirical data from Nicaragua) for analysis to capture uncertainty in the model parameters as a form of probabilistic sensitivity analysis. We report results as a mean of outcomes across these top 50 parameter sets; incremental cost-effectiveness ratios are reported as the ratio of the mean costs divided by the mean effects of one strategy versus another across sets.

Calibration targets

We assessed model fit by comparing projected model outcomes of age-specific prevalence of high-risk HPV and age-specific cancer incidence relative to empirical data from Nicaragua. The scoring algorithm included age-specific prevalence of high-risk HPV and age-specific cervical cancer incidence.

Age-specific prevalence of high-risk HPV was drawn from the Screening Technologies to Advance Rapid Testing for Cervical Cancer Prevention–Utility and Program Planning (START-UP) project data on *care*HPV positivity using a cut-off ratio cut-point of 0.5 relative light units (**Table A.1**). For each age group, we derived a 95% binomial confidence interval around the point prevalence, which comprised the calibration target. The likelihood function for each age group was assumed to follow a binomial distribution.

Age-specific cancer incidence was drawn from Globocan due to the lack of a local cancer registry [11] (**Table A.2**). The likelihood function for each age group was assumed to follow a normal distribution.

For peer review only - http://bmjopen.bmj.com/site/about/guidelines.xhtml

Composite goodness-of-fit scores for each input parameter set were generated by summing the log likelihood of each model outcome (i.e., age-specific HPV prevalence, age-specific cancer incidence). The 50 input parameter sets with the highest goodness-of-fit scores yielded the model outputs that were simultaneously closest to all calibration targets, and these were selected for analysis. **Figures A.1** and **A.2** display model fit to epidemiologic data on age-specific prevalence of high-risk HPV and age-specific cancer incidence.

Transition probabilities for the calibrated natural history model are presented in **Table A.3.**

COST DATA

Direct Medical Costs: Screening, Diagnosis, and Treatment of Precancerous Lesions

The direct medical costs of screening, diagnosis, and treatment of precancerous lesions were drawn from the START-UP demonstration study in Masaya department and from the Scale-Up Nicaragua Project in the departments of Carazo, Chontales, and Chinandega. Direct medical costs included clinical staff time, clinical supplies, drugs, clinical equipment, laboratory staff time, laboratory supplies, and laboratory equipment. Costs from the START-UP study were collected in 2010 local currency units and inflated to year 2015 levels using Nicaragua GDP deflators and then converted to US$ using the official exchange rate [12]; the exception was for equipment, which was generally procured in the United States and was inflated to year 2015 levels using US GDP deflators. Costs from the Scale-Up Nicaragua project (including equipment) were collected in 2015 local currency units and converted to US$ using the official exchange rate [12].

Costs are reported in **Table A.4** (screening procedures) and **Table A.5** (precancer diagnostic and treatment procedures).

Women's Time and Transportation Costs

To account for the opportunity cost of women's time spent traveling to, waiting for, or receiving care, we valued women's time using Nicaragua's 2013 monthly minimum wage [13], converted to 2015 US$. While the minimum wage may be a conservative estimate for valuation of women's time if they are formally employed, it can serve as a proxy for the societal value of women's time spent working in the informal sector or at home. We converted this to an hourly wage rate assuming 171 hours of work per month (40 hours per week).

Estimates for time spent traveling to, waiting for, and receiving care were dependent upon the facility level where care was assumed to take place (**Table A.6**). While screening and delivery of primary screening results were assumed to take place at basic primary health facilities, we assumed cryotherapy and LEEP treatment were only provided at referral facilities. Estimates of women's time spent receiving a procedure were based on site-specific data from the START-UP demonstration projects, with staff time spent on the procedure (excluding preparation and registration time, which we assumed were built into patient waiting time) used as a proxy for women's procedure time. Estimates of women's wait time for screening and delivery of primary screening results was based on the average wait time for screening at facilities in the Chinandega department; wait time for cryotherapy, colposcopy, and LEEP was based on the average wait time at referral centers in the Chontales department. Round-trip transportation time was based on Scale-Up project staff estimates of the typical amount of time women spent traveling to screening or referral facilities, using a sample of 9 facilities the Carazo department and 10 facilities in the Chontales and Chinandega departments. We averaged across screening and referral facilities within

For peer review only - http://bmjopen.bmj.com/site/about/guidelines.xhtml

each department to obtain a department average, and then averaged across departments to obtain average round-trip transportation times based on facility level. Estimates of women's time and time costs are presented in **Table A.7.**

Women's transportation costs were based on Scale-Up project staff reports of the typical mode of transportation to a particular facility by women in the catchment area, using a sample of 9 facilities in Carazo and 10 facilities each in Chontales or Chinandega. We used the most commonly reported mode of transportation for each department (for screening facilities: walking for Carazo; walking for Chontales; bus for Chinandega; for referral facilities: bus in all three departments). The cost of a bus trip was based on typical bus fare for the distance to the health facility, as reported by Scale-Up project staff. We averaged the typical transportation costs across departments to obtain an estimate of women's round-trip transportation costs to and from a health facility, assuming the same mode of transport was used for the trip to and from the health facility. Women's transportation costs are presented in **Table A.8.**

Programmatic Costs

We included the programmatic cost of the following types of training sessions that were conducted during the Scale-Up project: 1) for health care personnel to offer careHPV screening and counsel women on results; 2) for laboratory technicians to run the careHPV assay; 3) for community outreach workers and auxiliary nurses to promote careHPV screening and offer self-collection of HPV samples; and 4) for health care providers to offer VIA and cryotherapy. We included the cost of trainer time and trainee time (valued using average monthly salaries converted to an hourly rate; volunteer outreach worker time was valued using the minimum wage), as well as the cost of supplies, materials, transportation, and refreshments. To derive an average health care personnel training cost per woman screened, we divided the total training cost for health care personnel by 100,000, corresponding to the number of women screened during the Scale-Up project. To derive an average laboratory technician training cost per woman screened, we divided the total training cost for laboratory technicians by 100,000. To derive an average community outreach worker training cost per woman screened, we divided the total training cost for outreach workers by 80,000, corresponding to the number of women who participated in self-collection of HPV samples. To derive an average health care provider training cost per woman receiving VIA and cryotherapy, we divided the total VIA/cryotherapy training cost for health care providers by 16,000, corresponding to the approximate number of women who tested HPV-positive in Scale-Up and would thus require visual assessment (to determine eligibility for cryotherapy) and, if eligible, cryotherapy.

All women screened with HPV testing incurred the per-woman training costs for health care personnel and laboratory technicians. Only women screened with HPV self-collection incurred the per-woman cost of training outreach workers to offer self-collection (due to lack of data on self-collection in a community setting, in the base case analysis we assumed self-collection took place at the clinic; however, we assumed outreach workers would be involved in mobilizing and educating women about self-collection, and conservatively included the cost of training so as to represent a known programmatic cost as self-collection efforts shift to also include community settings). Only women who received either VIA (as triage) or screen-and-treat cryotherapy incurred the per-woman cost of training health providers to perform VIA and cryotherapy. Training costs are presented in **Table A.9.**

Cost of Cancer Care by Stage

Costs associated with cancer care by stage (Local versus Regional or Distant), including direct medical costs, direct non-medical costs (i.e., transport to a cancer center, and meals and temporary housing

For peer review only - http://bmjopen.bmj.com/site/about/guidelines.xhtml

during the course of treatment), and patient and support person time were derived from a previous analysis in El Salvador [14]. Based on expert opinion in El Salvador, we assumed 50% of local cancer patients presented with FIGO stage 1a, requiring radical hysterectomy, and 50% of local cancer patients presented with FIGO stage 1b/2a, requiring cobalt therapy followed by 5 chemotherapy sessions followed by brachytherapy. We assumed all women presenting with FIGO stage 2b or higher received cobalt therapy followed by 5 chemotherapy sessions followed by brachytherapy.

To adjust the direct medical costs of cancer treatment in El Salvador to the setting of Nicaragua, we multiplied by the ratio of WHO-CHOICE inpatient bed-day costs at a teaching hospital in Nicaragua relative to El Salvador. We assumed each patient and a support person spent the same amount of time traveling, waiting for, and receiving care as in El Salvador, and valued this time at the 2013 minimum wage rate in Nicaragua (inflated and converted to 2015 US$). To adjust temporary housing and transportation costs to the setting of Nicaragua, we multiplied the Salvadoran costs by the ratio of GDP per capita in Nicaragua relative to El Salvador. Costs are reported in **Table A.10**.

SUPPLEMENTARY RESULTS

Additional results described in the main manuscript are presented in **Figures A3** to **A6.**

For peer review only - http://bmjopen.bmj.com/site/about/guidelines.xhtml

Table A.1. Age-specific prevalence of high-risk HPV, Nicaragua [15].[a]

Age group	Number of women	Number of women with high-risk HPV	Prevalence (95% CI)
30 – 34 years	1,693	310	0.18 (0.17, 0.20)
35 – 39 years	1,141	184	0.16 (0.14, 0.18)
40 – 44 years	933	125	0.13 (0.11, 0.16)
45 – 49 years	878	121	0.14 (0.12, 0.16)

[a] HPV positivity was based on a cut-off of 0.5 relative light units.

Table A.2. Age-specific cervical cancer incidence, Nicaragua (GLOBOCAN 2012) [11].

Age group	Cases	Rate per 100,000 women (95% CI)
40 – 44 years	123	78.7 (64.8, 92.6)
45 – 49 years	112	85.4 (69.6, 101.2)
50 – 54 years	102	88.4 (71.2, 105.6)
55 – 59 years	85	88.1 (69.4, 106.8)
60 – 64 years	51	84.0 (61.0, 107.1)
65 – 69 years	37	80.8 (54.8, 106.8)
70 – 74 years	30	74.6 (47.9, 101.3)
≥75 years	45	70.3 (49.8, 90.8)

For peer review only

For peer review only - http://bmjopen.bmj.com/site/about/guidelines.xhtml

Table A.3. Monthly transition probabilities in the calibrated natural history model of HPV infection and cervical carcinogenesis.

HPV Infection

HPV Type and Age Group, years	Baseline Value		Range of Multiplier Values Among Top 50 Parameter Sets[a]	
	Min	Max	Min	Max
**HPV 16**				
<21	0	0.00186	3.15278	7.90861
21-24	0.000903	0.00125	0.670187	6.75291
25-29	0.000782	0.00087	0.604518	7.39154
30-49	0.000602	0.00078	0.677505	7.86645
>= 50	0.000217	0.00059	0.363714	8.9736
**HPV 18**				
<21	0	0.00116	0.365305	9.94514
21-24	0.0011	0.001173	0.304444	8.18583
25-29	0.0006	0.001	0.305103	8.23571
30-49	0.000301	0.00058	0.336658	9.81842
>= 50	0.000109	0.000295	0.347223	9.86684
**HPV 31**				
<21	0	0.001428	0.432486	7.54208
21-24	0.0009	0.001364	0.332414	5.86815
25-29	0.0006	0.000805	0.312983	6.63163
30-49	0.000301	0.00055	0.381602	7.86096
>= 50	0.000109	0.000295	0.445801	7.91394
**HPV 33**				
<21	0	0.000786	0.337263	5.98918
21-24	0.000412	0.00069	0.321392	5.21972
25-29	0.00025	0.00036	0.321163	4.52998
30-49	0.000131	0.00022	0.315971	5.91849
>= 50	0.000054	0.000128	0.341468	5.73381
**HPV 45**				
<21	0	0.001	0.838185	7.90205
21-24	0.00041	0.0008	0.303553	6.97879
25-29	0.000315	0.00037	0.373551	7.22544
30-49	0.000161	0.000305	0.320699	7.65339
>= 50	0.000054	0.00015	0.313646	7.97911

For peer review only

**HPV 52**				
<21	0	0.001186	0.339479	5.92479
21-24	0.00027	0.0008	0.328541	4.11892
25-29	0.000184	0.000254	0.301478	4.73227
30-49	0.00015	0.000173	0.365365	5.61621
>= 50	0.000054	0.000148	0.333456	11.406
**HPV 58**				
<21	0	0.001189	0.586648	7.78762
21-24	0.00059	0.0011	0.30793	7.02402
25-29	0.000465	0.00055	0.376855	6.55248
30-49	0.000244	0.00045	0.300592	7.20446
>= 50	0.000054	0.000239	0.42182	7.99349
**Other Oncogenic Types**				
<21	0	0.00247	0.324069	5.82435
21-24	0.001804	0.0023	0.301534	5.30746
25-29	0.0012	0.0017	0.311474	4.99753
30-49	0.000602	0.0011	0.318774	5.86776
>= 50	0.000217	0.00059	0.313403	5.86636
**Nononcogenic Types**				
<21	0	0.00261	0.361451	14.721
21-24	0.00255	0.00262	0.327572	10.6641
25-29	0.002	0.0025	0.327932	13.399
30-49	0.00085	0.00186	0.326549	14.4844
>= 50	0.000217	0.0008	0.324229	14.05

HPV clearance

HPV Type and time since infection, months	Baseline Value	Multiplier Value Among Top 50 Parameter Sets[a]
**HPV 16**		
1-15	0.041886	1
16-27	0.040754	1
28-39	0.033905	1
40-51	0.031888	1
52+	0.019846	1
**HPV 18**		
1-15	0.073342	1
16-27	0.063235	1
28-39	0.053605	1
40-51	0.020616	1

For peer review only

52+	0.020616	1
**HPV 31**		
1-15	0.063447	1
16-27	0.033826	1
28-39	0.033826	1
40-51	0.033826	1
52+	0.033826	1
**HPV 33**		
1-15	0.083452	1
16-27	0.044955	1
28-39	0.036156	1
40-51	0.036156	1
52+	0.036156	1
**HPV 45**		
1-15	0.078517	1
16-27	0.042579	1
28-39	0.041675	1
40-51	0.030133	1
52+	0.030133	1
**HPV 52**		
1-15	0.062999	1
16-27	0.044401	1
28-39	0.044401	1
40-51	0.039325	1
52+	0.039325	1
**HPV 58**		
1-15	0.065572	1
16-27	0.05443	1
28-39	0.053968	1
40-51	0.033319	1
52+	0.033319	1
**High Risk HPV**		
1-15	0.080766	1
16-27	0.066633	1
28-39	0.053972	1
40-51	0.049229	1
52+	0.005094	1

For peer review only

For peer review only - http://bmjopen.bmj.com/site/about/guidelines.xhtml

**Low Risk HPV**		
1-15	0.051888	1
16-27	0.050005	1
28-39	0.034649	1
40-51	0.034649	1
52-63	0.034649	1
64+	0.028608	1

HPV Progression to CIN2

HPV Type and time since infection, months	Baseline Value	Multiplier Value Among Top 50 Parameter Sets[a]
**HPV 16**		
1-15	0.001707	1
16-27	0.002422	1
28-39	0.002577	1
40-51	0.005518	1
52+	0.014998	1
**HPV 18**		
1-15	4.27E-05	1
16-27	0.000189	1
28-39	0.000189	1
40-51	0.007733	1
52+	0.007733	1
**HPV 31**		
1-15	0.000262	1
16-27	0.00278	1
28-39	0.003091	1
40-51	0.006931	1
52+	0.006931	1
**HPV 33**		
1-15	0.000719	1
16-27	0.000719	1
28-39	0.004939	1
40-51	0.004939	1
52+	0.004939	1
**HPV 45**		
1-15	0	1
16-27	0	1

For peer review only - http://bmjopen.bmj.com/site/about/guidelines.xhtml

28-39	0.002258	1
40-51	0.005333	1
52+	0.005333	1
**HPV 52**		
1-15	0.000884	1
16-27	0.001681	1
28-39	0.001681	1
40-51	0.001978	1
52+	0.005678	1
**HPV 58**		
1-15	0.000587	1
16-27	0.002468	1
28-39	0.002468	1
40-51	0.004617	1
52+	0.010246	1
**High Risk HPV**		
1-15	0.000126	1
16-27	0.000373	1
28-39	0.00196	1
40-51	0.00196	1
52+	0.00196	1
**Low Risk HPV**		
1-15	0.000205	1
16-27	0.000291	1
28-39	0.00031	1
40-51	0.000663	1
52+	0.000663	1

HPV progression to CIN3

**HPV Type and time since infection, months**	Baseline Value	Multiplier Value Among Top 50 Parameter Sets[a]
**HPV 16**		
1-15	0.000569	1
16-27	0.000808	1
28-39	0.00086	1
40-51	0.001843	1
52+	0.005024	1
**HPV 18**		

For peer review only - http://bmjopen.bmj.com/site/about/guidelines.xhtml

1-15	1.07E-05	1
16-27	4.72E-05	1
28-39	4.72E-05	1
40-51	0.001939	1
52+	0.001939	1
**HPV 31**		
1-15	6.55E-05	1
16-27	0.000696	1
28-39	0.000774	1
40-51	0.001737	1
52+	0.001737	1
**HPV 33**		
1-15	0.00018	1
16-27	0.00018	1
28-39	0.001237	1
40-51	0.001237	1
52+	0.001237	1
**HPV 45**		
1-15	0	1
16-27	0	1
28-39	0.000565	1
40-51	0.001336	1
52+	0.001336	1
**HPV 52**		
1-15	0.000221	1
16-27	0.000421	1
28-39	0.000421	1
40-51	0.000495	1
52+	0.001423	1
**HPV 58**		
1-15	0.000147	1
16-27	0.000618	1
28-39	0.000618	1
40-51	0.001156	1
52+	0.002571	1
**High Risk HPV**		
1-15	3.16E-05	1

For peer review only

16-27	9.32E-05	1
28-39	0.00049	1
40-51	0.00049	1
52+	0.00049	1
**Low Risk HPV**		
1-15	2.28E-05	1
16-27	3.23E-05	1
28-39	3.44E-05	1
40-51	7.37E-05	1
52+	7.37E-05	1

Regression of CIN2 to NL

HPV Type and time since infection, years	Baseline Value		Range of Multiplier Values Among Top 50 Parameter Sets[a]	
	Min	Max	Min	Max
**HPV 16**				
1-5	0.05	0.523578	0.500863	0.687389
6-10	0.035	0.523578	0.500863	0.687389
11-20	0.013	0.523578	0.500863	0.687389
21-29	0.0005	0.523578	0.500863	0.687389
30-39	0.0001	0.523578	0.500863	0.687389
40+	0.00005	0.523578	0.500863	0.687389
**All Other HPV Types**				
1-5	0.05	0.523578	0.527319	3.70898
6-10	0.035	0.523578	0.527319	3.70898
11-20	0.013	0.523578	0.527319	3.70898
21-29	0.0005	0.523578	0.527319	3.70898
30-39	0.0001	0.523578	0.527319	3.70898
40+	0.00005	0.523578	0.527319	3.70898

Regression of CIN3 to NL

HPV Type and time since infection, years	Baseline Value		Range of Multiplier Values Among Top 50 Parameter Sets[a]	
	Min	Max	Min	Max
**HPV 16**				
1-5	0.025	0.639221	0.50664	1.67845
6-10	0.0175	0.639221	0.50664	1.67845
11-20	0.0065	0.639221	0.50664	1.67845

For peer review only - http://bmjopen.bmj.com/site/about/guidelines.xhtml

21-29	0.00025	0.639221	0.50664	1.67845
30-39	0.00005	0.639221	0.50664	1.67845
40+	0.000025	0.639221	0.50664	1.67845
**All Other HPV Types**				
1-5	0.025	0.639221	0.577736	3.9781
6-10	0.0175	0.639221	0.577736	3.9781
11-20	0.0065	0.639221	0.577736	3.9781
21-29	0.00025	0.639221	0.577736	3.9781
30-39	0.00005	0.639221	0.577736	3.9781
40+	0.000025	0.639221	0.577736	3.9781

Progression of CIN2 to CA

HPV Type and time since infection, years	Baseline Value	Range of Multiplier Values Among Top 50 Parameter Sets[a]	
		Min	Max
**HPV16**			
1-5	3.29E-05	1.00901	1.49572
6-10	3.56E-05	1.00901	1.49572
11-20	0.000857	1.00901	1.49572
21-29	0.002506	1.00901	1.49572
30-34	0.005011	1.00901	1.49572
35-39	0.005429	1.00901	1.49572
40-44	0.011693	1.00901	1.49572
45-49	0.012528	1.00901	1.49572
50+	0.33408	1.00901	1.49572
**HPV 18**			
1-5	3.29E-05	1.00901	1.49572
6-10	3.56E-05	1.00901	1.49572
11-20	0.000571	1.00901	1.49572
21-29	0.002506	1.00901	1.49572
30-34	0.005011	1.00901	1.49572
35-39	0.005429	1.00901	1.49572
40-44	0.011693	1.00901	1.49572
45-49	0.012528	1.00901	1.49572
50+	0.33408	1.00901	1.49572
**HPV 31**			
1-5	2.2E-05	1.00901	1.49572
6-10	2.38E-05	1.00901	1.49572
11-20	0.000857	1.00901	1.49572

For peer review only

21-29	0.00167	1.00901	1.49572
30-34	0.003341	1.00901	1.49572
35-39	0.003619	1.00901	1.49572
40-44	0.007795	1.00901	1.49572
45-49	0.008352	1.00901	1.49572
50+	0.22272	1.00901	1.49572
**HPV 33**			
1-5	3.29E-05	1.00901	1.49572
6-10	3.56E-05	1.00901	1.49572
11-20	0.000571	1.00901	1.49572
21-29	0.002506	1.00901	1.49572
30-34	0.005011	1.00901	1.49572
35-39	0.005429	1.00901	1.49572
40-44	0.011693	1.00901	1.49572
45-49	0.012528	1.00901	1.49572
50+	0.33408	1.00901	1.49572
**HPV 45**			
1-5	2.2E-05	1.00901	1.49572
6-10	2.38E-05	1.00901	1.49572
11-20	0.000571	1.00901	1.49572
21-29	0.00167	1.00901	1.49572
30-34	0.003341	1.00901	1.49572
35-39	0.003619	1.00901	1.49572
40-44	0.007795	1.00901	1.49572
45-49	0.008352	1.00901	1.49572
50+	0.22272	1.00901	1.49572
**HPV 52**			
1-5	2.2E-05	1.00901	1.49572
6-10	2.38E-05	1.00901	1.49572
11-20	0.000571	1.00901	1.49572
21-29	0.00167	1.00901	1.49572
30-34	0.003341	1.00901	1.49572
35-39	0.003619	1.00901	1.49572
40-44	0.007795	1.00901	1.49572
45-49	0.008352	1.00901	1.49572
50+	0.22272	1.00901	1.49572
**HPV 58**			
1-5	2.2E-05	1.00901	1.49572
6-10	2.38E-05	1.00901	1.49572

For peer review only

11-20	0	1.00901	1.49572
21-29	0.00167	1.00901	1.49572
30-34	0.003341	1.00901	1.49572
35-39	0.003619	1.00901	1.49572
40-44	0.007795	1.00901	1.49572
45-49	0.008352	1.00901	1.49572
50+	0.22272	1.00901	1.49572
**High Risk HPV**			
1-5	2.2E-05	1.00901	1.49572
6-10	2.38E-05	1.00901	1.49572
11-20	0.000571	1.00901	1.49572
21-29	0.00167	1.00901	1.49572
30-34	0.003341	1.00901	1.49572
35-39	0.003619	1.00901	1.49572
40-44	0.007795	1.00901	1.49572
45-49	0.008352	1.00901	1.49572
50+	0.22272	1.00901	1.49572

Progression of CIN3 to CA

**HPV Type and time since infection, years**	Baseline Value	Range of Multiplier Values Among Top 50 Parameter Sets[a]	
		Min	Max
**HPV 16**			
1-5	0.000165	1.00199	1.48007
6-10	0.000178	1.00199	1.48007
11-20	0.004284	1.00199	1.48007
21-29	0.012528	1.00199	1.48007
30-34	0.025056	1.00199	1.48007
35-39	0.027144	1.00199	1.48007
40-44	0.058464	1.00199	1.48007
45-49	0.06264	1.00199	1.48007
50+	0.33408	1.00199	1.48007
**HPV 18**			
1-5	0.000165	1.00199	1.48007
6-10	0.000178	1.00199	1.48007
11-20	0.004284	1.00199	1.48007
21-29	0.012528	1.00199	1.48007
30-34	0.025056	1.00199	1.48007
35-39	0.027144	1.00199	1.48007
40-44	0.058464	1.00199	1.48007
45-49	0.06264	1.00199	1.48007

For peer review only - http://bmjopen.bmj.com/site/about/guidelines.xhtml

50+	0.33408	1.00199	1.48007
**HPV 31**			
1-5	0.00011	1.00199	1.48007
6-10	0.000119	1.00199	1.48007
11-20	0.002856	1.00199	1.48007
21-29	0.008352	1.00199	1.48007
30-34	0.016704	1.00199	1.48007
35-39	0.018096	1.00199	1.48007
40-44	0.038976	1.00199	1.48007
45-49	0.04176	1.00199	1.48007
50+	0.22272	1.00199	1.48007
**HPV 33**			
1-5	0.000165	1.00199	1.48007
6-10	0.000178	1.00199	1.48007
11-20	0.004284	1.00199	1.48007
21-29	0.012528	1.00199	1.48007
30-34	0.025056	1.00199	1.48007
35-39	0.027144	1.00199	1.48007
40-44	0.058464	1.00199	1.48007
45-49	0.06264	1.00199	1.48007
50+	0.33408	1.00199	1.48007
**HPV 45**			
1-5	0.00011	1.00199	1.48007
6-10	0.000119	1.00199	1.48007
11-20	0.002856	1.00199	1.48007
21-29	0.008352	1.00199	1.48007
30-34	0.016704	1.00199	1.48007
35-39	0.018096	1.00199	1.48007
40-44	0.038976	1.00199	1.48007
45-49	0.04176	1.00199	1.48007
50+	0.22272	1.00199	1.48007
**HPV 52**			
1-5	0.00011	1.00199	1.48007
6-10	0.000119	1.00199	1.48007
11-20	0.002856	1.00199	1.48007
21-29	0.008352	1.00199	1.48007
30-34	0.016704	1.00199	1.48007
35-39	0.018096	1.00199	1.48007
40-44	0.038976	1.00199	1.48007

For peer review only

45-49	0.04176	1.00199	1.48007
50+	0.22272	1.00199	1.48007
**HPV 58**			
1-5	0.00011	1.00199	1.48007
6-10	0.000119	1.00199	1.48007
11-20	0.002856	1.00199	1.48007
21-29	0.008352	1.00199	1.48007
30-34	0.016704	1.00199	1.48007
35-39	0.018096	1.00199	1.48007
40-44	0.038976	1.00199	1.48007
45-49	0.04176	1.00199	1.48007
50+	0.22272	1.00199	1.48007
**High Risk HPV**			
1-5	0.00011	1.00199	1.48007
6-10	0.000119	1.00199	1.48007
11-20	0.002856	1.00199	1.48007
21-29	0.008352	1.00199	1.48007
30-34	0.016704	1.00199	1.48007
35-39	0.018096	1.00199	1.48007
40-44	0.038976	1.00199	1.48007
45-49	0.04176	1.00199	1.48007
50+	0.22272	1.00199	1.48007

Progression of invasive cancer stages

	Baseline Value
Local to regional	0.02
Regional to distant	0.025

Invasive cancer mortality

Local	0.005876
Regional	0.015137
Distant	0.065519

Probability of symptom detection

Local	0.0039
Regional	0.1333
Distant	0.1746

For peer review only

[a] Multiplier values were drawn from the identified plausible search ranges and applied to baseline input probabilities; the values presented represent the multiplier values that yielded the 50 best fits to the epidemiologic calibration targets.

For peer review only - http://bmjopen.bmj.com/site/about/guidelines.xhtml

For peer review only

Table A.4. Screening: Direct medical costs per procedure (2015 US$).[a]

Cost category	HPV test (provider-collection)[b]	HPV test (self-collection)[b]	Cytology	VIA triage	Source
Clinical staff	3.81	2.88	3.81	3.81	START-UP
Clinical supplies	6.72	6.72	0.82	0.36	Scale-Up (HPV);[c] START-UP (Cytology/VIA)
Clinical equipment	0.01	0.01	0.01	0.02	
Lab staff	0.41	0.41	1.37	0.00	Scale-Up (transport personnel);[d] START-UP (other)
Lab supplies	0.81	0.81	0.92	0.00	Scale-Up (transport fuel);[e] START-UP (other)
Lab equipment	0.21	0.21	0.32	0.00	START-UP
**Total direct medical cost**	**11.96**	**11.04**	**7.26**	**4.19**	

[a] HPV: human papillomavirus; Scale-Up: Scale-Up demonstration project (2015); START-UP: Screening Technologies to Advance Rapid Testing for Cervical Cancer Prevention–Utility and Program Planning demonstration project (2010); US$: United States dollars; VIA: visual inspection with acetic acid.

[b] HPV testing with both provider-collection of cervical samples and self-collection of vaginal samples was assumed to take place at the clinic. The difference in costs between collection modalities is due to reduced clinical staff time when a woman self-collects her own sample.

[c] The cost of clinical supplies for HPV testing was drawn from the Scale-Up project and includes the test kit, plate sealers, brush, collection medium, pipette tips (3 types), gloves, and towels to decant and dry.

[d] The cost of laboratory staff time was drawn from the START-UP project, with the exception of preparing and transporting samples to the laboratory, which was drawn from the Scale-Up project. Staff time spent preparing samples for transport in the Scale-Up project was based on project staff estimates in each of the three departments (Carazo, Chontales, and Chinandega), which ranged from 5 to 7 minutes per sample. Staff time for round-trip transport of samples was averaged across 9 to 10 facilities in each of the three departments, and valued based on the average monthly wage of the personnel responsible for transporting the samples in each department. We derived an average cost per sample prepared and transported for each department (assuming the cost of staff time per sample was equivalent for HPV and cytology specimens), and then averaged these costs across the three departments.

[e] For HPV testing, all lab supplies are bundled in the category of clinical supplies, with the exception of fuel for laboratory transport, which is included under lab supplies. For both HPV and cytology testing, we assumed the same cost of fuel per sample. Using Scale-Up project staff estimates of the number of samples transported per load, the distance to the lab from 9 to 10 facilities in each department, and the usual type of vehicle used in each department, we derived the average fuel cost per sample using the average fuel economy of major vehicle types [16] and the average gas price per liter in Nicaragua [12].

For peer review only - http://bmjopen.bmj.com/site/about/guidelines.xhtml

Table A.5. Diagnosis and Treatment of Precancer: Direct medical costs per procedure (2015 US$).[a]

Cost category	Colposcopy	Colposcopy and biopsy	Cryotherapy	LEEP	Source
Clinical staff	5.92	6.29	7.54	7.34	START-UP
Clinical supplies	0.38	1.39	3.54	36.05	Scale-Up (cryotherapy gas);[b] START-UP (other)
Drugs	0.13	0.13	0.30	0.10	START-UP
Clinical equipment	0.49	0.53	9.13	13.29	Scale-Up (cryotherapy unit);[c] START-UP (other)
Lab staff	0.00	4.09	0.00	4.09	START-UP
Lab supplies	0.00	3.35	0.00	3.35	START-UP
Lab equipment	0.00	4.12	0.00	4.12	START-UP
**Total direct medical cost**	**6.91**	**19.91**	**18.16**	**68.36**	

[a] LEEP: loop electrosurgical excision procedure; Scale-Up: Scale-Up demonstration project (2015); START-UP: Screening Technologies to Advance Rapid Testing for Cervical Cancer Prevention–Utility and Program Planning demonstration project (2010); US$: United States dollars.

[b] We used estimates from Scale-Up project staff to inform the cost of gas used per cryotherapy procedure, based on the cost of 1 cylinder and the number of women treated per tank. We did not include the cost of the tank deposit.

[c] To estimate the cost per procedure of a cryotherapy unit, we assumed this equipment item was a tradable good, and accordingly converted the purchase price from local currency units to US$ using the official exchange rate. We annualized the cost with a 3% interest rate and assumed an economic life of 5 years. To derive the number of procedures per machine per year across the three departments considered, we divided the annual number of cryotherapy procedures by the number of cryotherapy units. We then divided the annualized cryotherapy unit cost by the number of procedures per machine per year.

For peer review only - http://bmjopen.bmj.com/site/about/guidelines.xhtml

Table A.6. Location of service delivery for screening, diagnosis, and treatment of precancerous lesions and cancer.[a]

Procedure	Location of services[b]
HPV DNA test	Primary facility
Cytology test	Primary facility
VIA test	Primary facility
Colposcopy/biopsy	Referral facility
Cryotherapy	Referral facility
LEEP	Referral facility
Follow-up visits (after cryotherapy or LEEP)	Primary facility
Cancer treatment	Tertiary facility

[a] HPV: human papillomavirus; LEEP: loop electrosurgical excision procedure; VIA: visual inspection with acetic acid.

For peer review only

For peer review only - http://bmjopen.bmj.com/site/about/guidelines.xhtml

Tabe A.7. Women's time estimates.[a]

Visit	Minutes	Cost (2015 US$)[b]	Source
**Screening**[c]			
Wait time	33	0.48	Scale-Up
Procedure time	20	0.29	START-UP
Transport time (round-trip)	57	0.82	Scale-Up
**Results**[c]			
Wait time	33	0.48	Scale-Up
Procedure time	2	0.03	START-UP
Transport time (round-trip)	57	0.82	Scale-Up
**Cryotherapy**[c]			
Wait time	120	1.75	Scale-Up
Procedure time	35	0.51	START-UP
Transport time (round-trip)	213	3.10	Scale-Up
**Colposcopy/biopsy**			
Wait time	120	1.75	Scale-Up
Procedure time	37	0.54	START-UP
Transport time (round-trip)	213	3.10	Scale-Up
**LEEP**			
Wait time	120	1.75	Scale-Up
Procedure time	25	0.36	START-UP
Transport time (round-trip)	213	3.10	Scale-Up

[a] LEEP: loop electrosurgical excision procedure; Scale-Up: Scale-Up demonstration project (2015); START-UP: Screening Technologies to Advance Rapid Testing for Cervical Cancer Prevention–Utility and Program Planning demonstration project (2010); US$: United States dollars.

[b] Women's time was valued using Nicaragua's 2013 monthly minimum wage [13], converted to 2015 US$. We converted this to an hourly wage rate assuming 171 hours of work per month (40 hours per week).

[c] Wait and transport time costs were accrued for each visit to a health facility, but we did not double-count wait and transport times when multiple procedures were performed in the same visit (e.g., VIA triage of screen-positive women and cryotherapy of VIA+ women accrued the wait time and transport time costs associated with a single visit to a referral facility, but accrued the time costs of both procedures.)

For peer review only - http://bmjopen.bmj.com/site/about/guidelines.xhtml

Table A.8. Women's transportation costs.[a]

Facility[b]	Round-trip transportation cost (2015 US$)	Source
Screening facility	0.41	Scale-Up
Referral facility	2.81	Scale-Up

[a] Scale-Up: Scale-Up demonstration project (2015); US$: United States dollar.

[b] Screening with cytology or HPV testing was assumed to take place at a screening facility (i.e., a primary level health facility). Triage testing with visual inspection with acetic acid (VIA), cryotherapy, colposcopy/biopsy, and loop electrosurgical excision procedure (LEEP) were assumed to take place at referral facilities.

For peer review only

For peer review only - http://bmjopen.bmj.com/site/about/guidelines.xhtml

Table A.9. Programmatic training costs for HPV-based screening.[a]

Type of training	Total cost of supplies/materials[b] (2015 US$)	Total cost of personnel time[c] (2015 US$)	Per woman cost[d] (2015 US$)
Health care personnel offering careHPV	2,758	5,784	0.09
Laboratory technicians processing careHPV assays	3,503	526	0.04
Community outreach workers promoting and offering self-collection	2,662	3,618	0.08
Health care providers offering VIA and cryotherapy	8,103	16,069	1.51

[a] HPV: human papillomavirus; US$: United States dollars; VIA: visual inspection with acetic acid.
[b] The total cost of supplies, materials, transportation, and refreshments for all training sessions of each type was converted from 2015 local currency units using the official exchange rate.
[c] The total cost of personnel time includes time costs for both the trainer and the trainees, valued using average monthly salary rates of each type of personnel present (and the proportion of trainees of each personnel type) and based on the length of the training session.
[d] To derive an average health care personnel training cost per woman screened, we divided the total training cost for health care personnel by 100,000, corresponding to the number of women screened during the Scale-Up project. To derive an average laboratory technician training cost per woman screened, we divided the total training cost for laboratory technicians by 100,000. To derive an average community outreach worker training cost per woman screened with self-collection, we divided the total training cost for outreach workers by 80,000, corresponding to the number of women who participated in self-collection of HPV samples (due to lack of data on self-collection in a community setting, in the base case analysis we assumed self-collection took place at the clinic; however, we assumed outreach workers would be involved in mobilizing and educating women about self-collection, and conservatively included the cost of training so as to represent a known programmatic cost as self-collection efforts shift to also include community settings). To derive an average health care provider training cost per woman receiving VIA or cryotherapy, we divided the total VIA/cryotherapy training cost for health care providers by 16,000, corresponding to the approximate number of women who tested HPV-positive in Scale-Up and would thus require visual assessment and cryotherapy.

For peer review only - http://bmjopen.bmj.com/site/about/guidelines.xhtml

Table A.10. Cancer treatment costs (2015 US$).

Cancer stage	Local cancer	Regional cancer	Distant cancer
Direct medical costs[a]	943.62	917.83	917.83
Woman and support person time	345.77	639.14	639.14
Transportation, temporary housing, and meals	196.78	389.50	389.50
**Total cancer treatment cost**	**1486**	**1946**	**1946**

[a] Direct medical costs are based on the premise that 50% of local cancer patients presented with FIGO stage 1a, requiring radical hysterectomy, and 50% of local cancer patients presented with FIGO stage 1b/2a, requiring cobalt therapy followed by 5 chemotherapy sessions followed by brachytherapy. We assumed all women presenting with FIGO stage 2b or higher received cobalt therapy followed by 5 chemotherapy sessions followed by brachytherapy.

[b] Patient and support person time costs value time using the minimum wage in Nicaragua, as described in the text.

[c] Direct non-medical costs include round-trip transportation for visits to a cancer center and meals and temporary housing during the course of treatment.

For peer review only - http://bmjopen.bmj.com/site/about/guidelines.xhtml

For peer review only

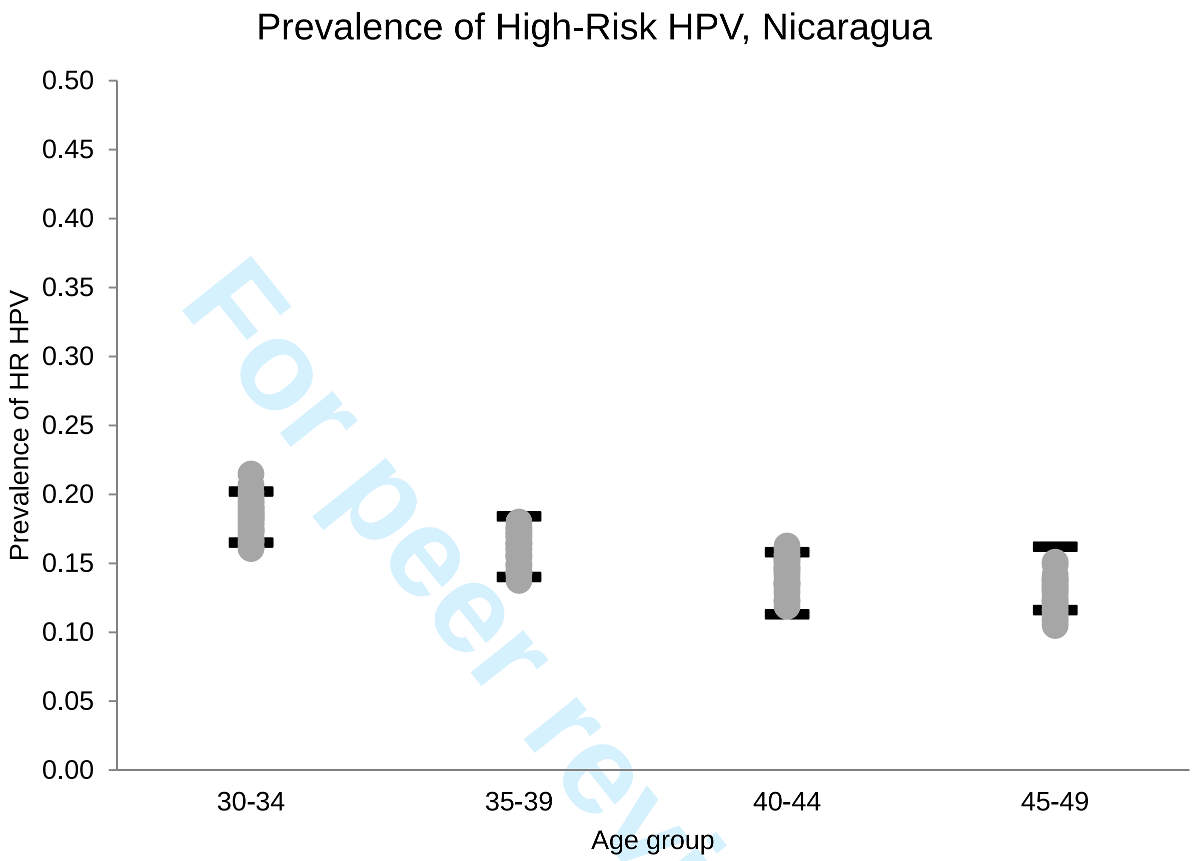

Figure A1. Selected model output from the top 50 input parameter sets compared with empirical data (i.e., calibration targets) on age-specific prevalence of high-risk HPV in Nicaragua, based on a relative light unit cut-off value of 0.5 in the START-UP studies [2, 15]. Bold lines represent the 95% confidence intervals around the empirical data, and gray circles represent model output from each of the top 50 input parameter sets.

For peer review only - http://bmjopen.bmj.com/site/about/guidelines.xhtml

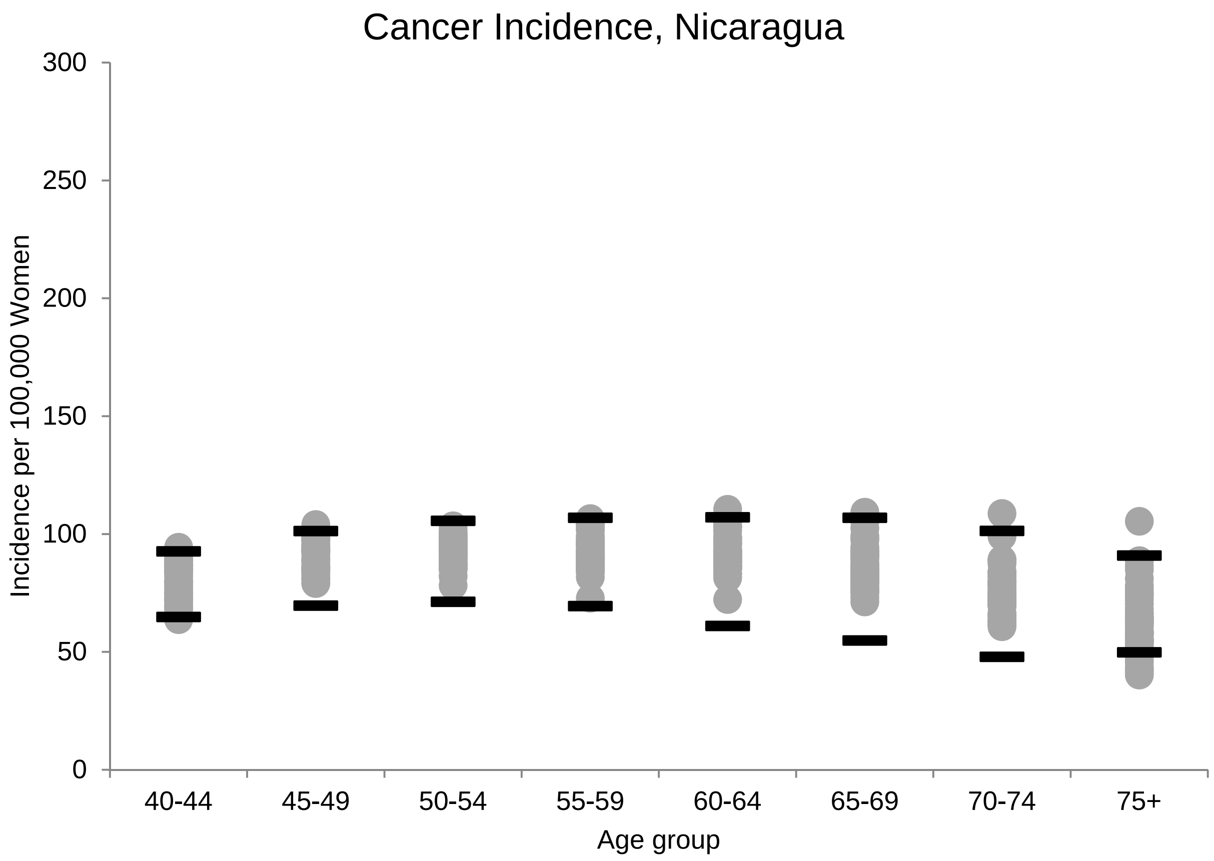

Figure A2. Selected model output from the top 50 input parameter sets compared with empirical data (i.e., calibration targets) on age-specific cancer incidence in Nicaragua [2, 11]. Bold lines represent the 95% confidence intervals around the empirical data, and gray circles represent model output from each of the top 50 input parameter sets.

For peer review only - http://bmjopen.bmj.com/site/about/guidelines.xhtml

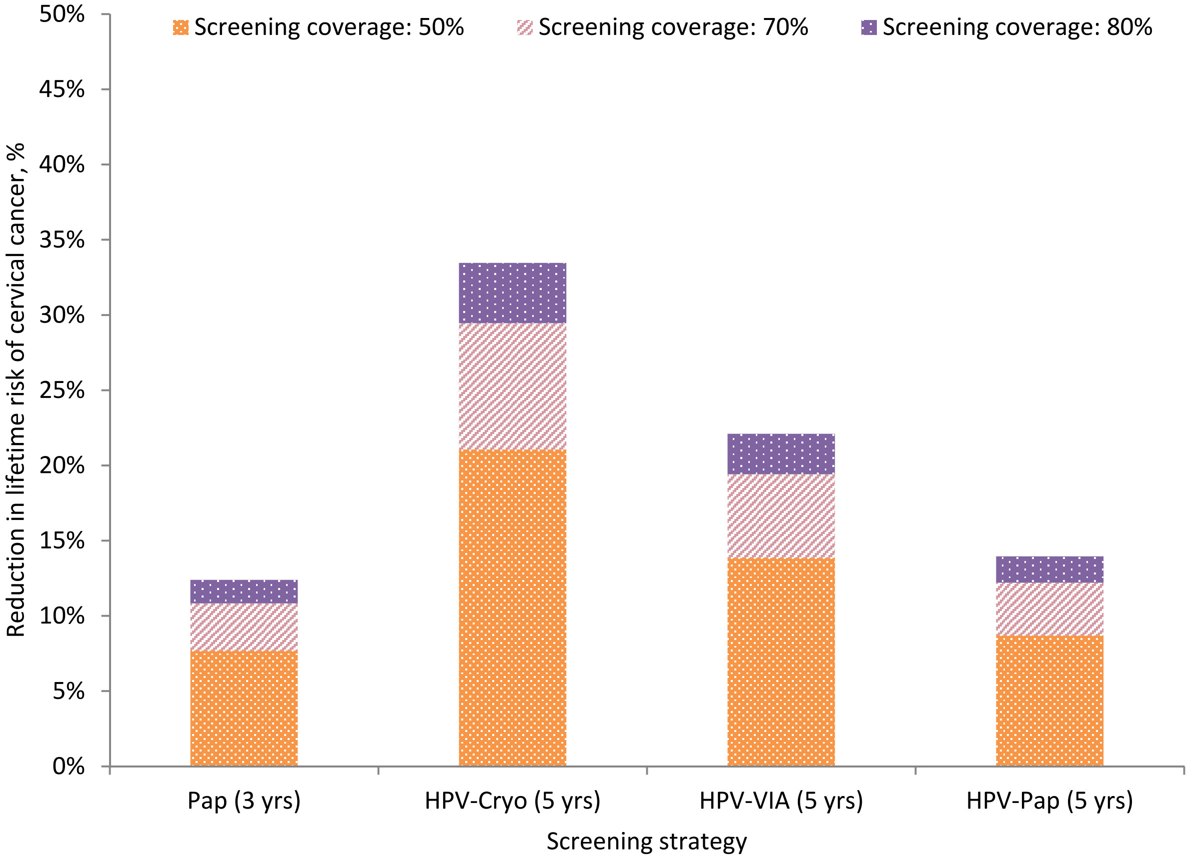

Figure A3. Reduction in lifetime risk of cervical cancer, by screening coverage level. Bars indicate the percent reduction in lifetime risk of cervical cancer for each screening strategy (Pap testing every 3 years; careHPV with cryotherapy for HPV-positive women every 5 years [HPV-Cryo]; careHPV every 5 years with visual inspection with acetic acid [VIA] triage of HPV-positive women [HPV-VIA]; and careHPV every 5 years with Pap triage of HPV-positive women [HPV-Pap]) as screening coverage of the target population increases.

view only

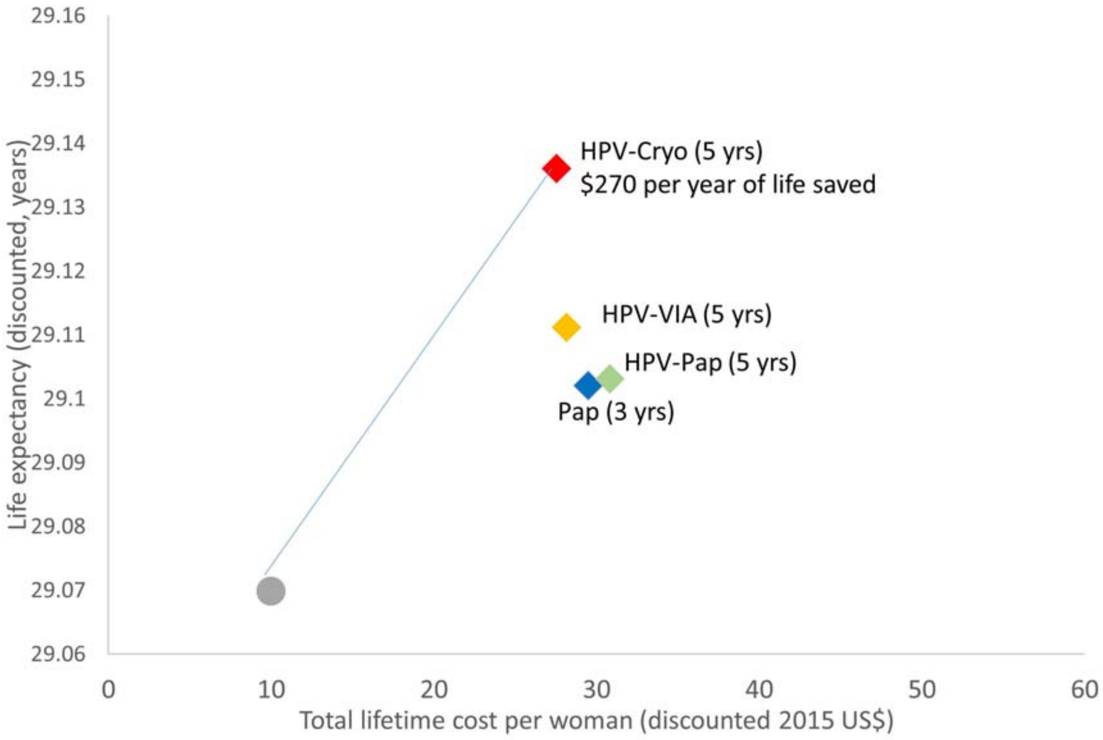

Figure A4. Cost-effectiveness analysis: Payer cost only. The graph displays the discounted lifetime costs (x-axis; in 2015 US$) and life expectancy (y-axis) associated with each screening strategy (Pap testing every 3 years; careHPV with cryotherapy for HPV-positive women every 5 years [HPV-Cryo]; careHPV every 5 years with visual inspection with acetic acid [VIA] triage of HPV-positive women [HPV-VIA]; and careHPV every 5 years with Pap triage of HPV-positive women [HPV-Pap]), when only costs to the payer are included (i.e., women's time and transportation costs are excluded). The cost-effectiveness associated with a change from one strategy to a more costly alternative is represented by the difference in cost divided by the difference in life expectancy associated with the two strategies. The curve indicates the strategies that are efficient because they are more effective and either 1) cost less; or 2) have a more attractive cost-effectiveness ratio than less effective options. The incremental cost-effectiveness ratio (ICER) is the reciprocal of the slope of the line connecting the two strategies under comparison. In this sensitivity analysis focused on costs to the payer, HPV-Cryo every 5 years was less costly and more effective than other screening strategies considered, and was thus a dominant strategy with an ICER of US$270 per year of life saved.

For peer review only - http://bmjopen.bmj.com/site/about/guidelines.xhtml

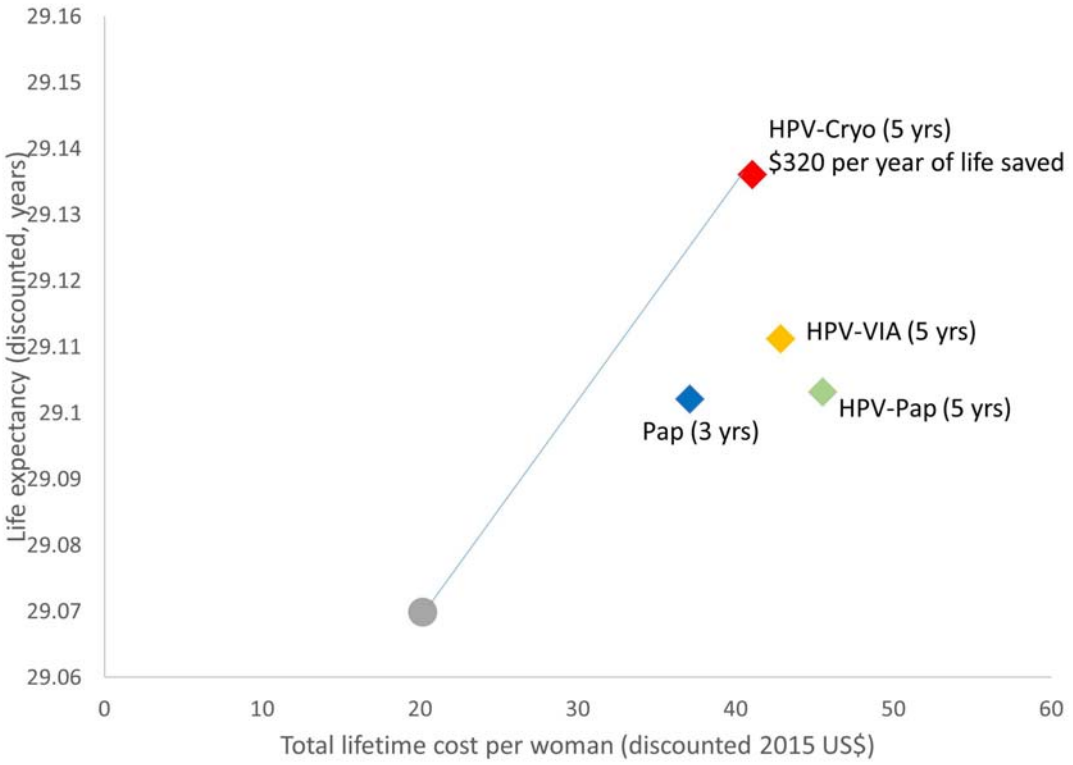

Figure A5. Cost-effectiveness analysis: Direct medical cost of Pap testing, US$3. The graph displays the discounted lifetime costs (x-axis; in 2015 US$) and life expectancy (y-axis) associated with each screening strategy (Pap testing every 3 years; careHPV with cryotherapy for HPV-positive women every 5 years [HPV-Cryo]; careHPV every 5 years with visual inspection with acetic acid [VIA] triage of HPV-positive women [HPV-VIA]; and careHPV every 5 years with Pap triage of HPV-positive women [HPV-Pap]), when the direct medical cost of Pap testing was assumed to be as low as US$3 (base case: US$7.26). The cost-effectiveness associated with a change from one strategy to a more costly alternative is represented by the difference in cost divided by the difference in life expectancy associated with the two strategies. The curve indicates the strategies that are efficient because they are more effective and either 1) cost less; or 2) have a more attractive cost-effectiveness ratio than less effective options. The incremental cost-effectiveness ratio (ICER) is the reciprocal of the slope of the line connecting the two strategies under comparison. In this sensitivity analysis examining the impact of a lower Pap test cost, HPV-Cryo every 5 years was more effective and had a more attractive cost-effectiveness ratio than Pap testing every 3 years; furthermore, HPV-Cryo was more effective and less costly than HPV-VIA and HPV-Pap, and was thus a dominant strategy with an ICER of US$320 per year of life saved.

For peer review only - http://bmjopen.bmj.com/site/about/guidelines.xhtml

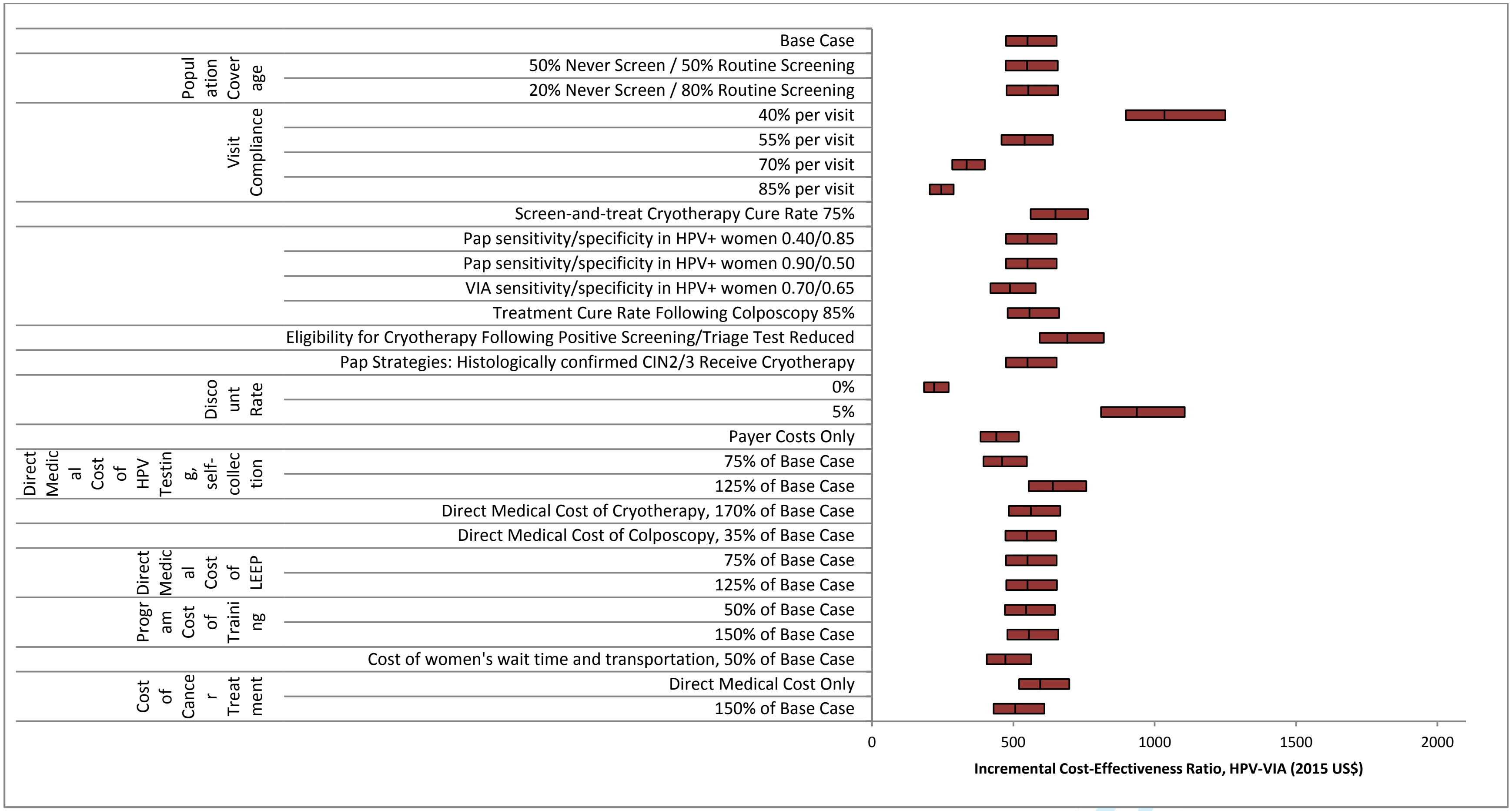

Figure A6. Base case and sensitivity analysis when HPV-Cryo is assumed to be unavailable: Incremental cost-effectiveness ratios, HPV-VIA strategy. Incremental cost-effectiveness ratios (ICERs) are presented (x-axis, 2015 US$ per year of life saved) for the base case and sensitivity analyses (y-axis), assuming HPV-Cryo is not available but Pap alone, HPV-VIA, and HPV-Pap are available. The orange bars represent the range of the ICERs for HPV-VIA every 5 years across the 50 input parameter sets, with the ICER of the mean costs divided by the mean effects demarcated by a black line. Nicaragua's per capita gross domestic product (GDP) is US$2,090, as a possible threshold that designates interventions as "very cost-effective".

For peer review only - http://bmjopen.bmj.com/site/about/guidelines.xhtml

References

1. Campos, N.G., et al., *An updated natural history model of cervical cancer: derivation of model parameters.* Am J Epidemiol, 2014. **180**(5): p. 545-55.
2. Campos, N.G., Tsu, V., Jeronimo, J., Mvundura, M., Lee, K., Kim, J.J., *When and how often to screen for cervical cancer in three low- and middle-income countries: A cost-effectiveness analysis.* Papillomavirus Research, 2015.
3. Munoz, N., et al., *Incidence, duration, and determinants of cervical human papillomavirus infection in a cohort of Colombian women with normal cytological results.* J Infect Dis, 2004. **190**(12): p. 2077-87.
4. McCredie, M.R., et al., *Natural history of cervical neoplasia and risk of invasive cancer in women with cervical intraepithelial neoplasia 3: a retrospective cohort study.* Lancet Oncol, 2008. **9**(5): p. 425-34.
5. Meyskens, F.L., Jr., et al., *Enhancement of regression of cervical intraepithelial neoplasia II (moderate dysplasia) with topically applied all-trans-retinoic acid: a randomized trial.* J Natl Cancer Inst, 1994. **86**(7): p. 539-43.
6. Keefe, K.A., et al., *A randomized, double blind, Phase III trial using oral beta-carotene supplementation for women with high-grade cervical intraepithelial neoplasia.* Cancer Epidemiol Biomarkers Prev, 2001. **10**(10): p. 1029-35.
7. Castle, P.E., et al., *Evidence for frequent regression of cervical intraepithelial neoplasia-grade 2.* Obstet Gynecol, 2009. **113**(1): p. 18-25.
8. Wang, S.M., et al., *Six-year regression and progression of cervical lesions of different human papillomavirus viral loads in varied histological diagnoses.* Int J Gynecol Cancer, 2013. **23**(4): p. 716-23.
9. Moscicki, A.B., et al., *Rate of and risks for regression of cervical intraepithelial neoplasia 2 in adolescents and young women.* Obstet Gynecol, 2010. **116**(6): p. 1373-80.
10. Herrero, R., et al., *Rationale and design of a community-based double-blind randomized clinical trial of an HPV 16 and 18 vaccine in Guanacaste, Costa Rica.* Vaccine, 2008. **26**(37): p. 4795-808.
11. Ferlay, J., et al., *GLOBOCAN 2012 v1.0, Cancer Incidence and Mortality Worldwide: IARC CancerBase No. 11 [Internet]*, 2013, International Agency for Research on Cancer: Lyon, France.
12. *World Development Indicators*, 2016, World Bank.
13. Database, I., *Country Profiles: Nicaragua*, 2016.
14. Campos, N.G., et al., *The comparative and cost-effectiveness of HPV-based cervical cancer screening algorithms in El Salvador.* Int J Cancer, 2015.
15. Jeronimo, J., et al., *A multicountry evaluation of careHPV testing, visual inspection with acetic acid, and papanicolaou testing for the detection of cervical cancer.* Int J Gynecol Cancer, 2014. **24**(3): p. 576-85.
16. Energy, U.S.D.o. *Average Fuel Economy of Major Vehicle Categories*. August 1, 2016]; Available from: http://www.afdc.energy.gov/data/10310.

For peer review only - http://bmjopen.bmj.com/site/about/guidelines.xhtml

BMJ Open

The Cost-Effectiveness of HPV-Based Cervical Cancer Screening in the Public Health System in Nicaragua

Journal:	*BMJ Open*
Manuscript ID	bmjopen-2016-015048.R2
Article Type:	Research
Date Submitted by the Author:	09-Mar-2017

Complete List of Authors:	Campos, Nicole; Harvard T.H. Chan School of Public Health, Health Policy and Management<br>Mvundura, Mercy; PATH, Devices and Tools Program<br>Jeronimo, Jose; PATH, Reproductive Global Health Program<br>Holme, Francesca; PATH, Reproductive Health Global Program<br>Vodicka, Elisabeth; University of Washington, School of Pharmacy<br>Kim, Jane; Harvard School of Public Health, Health Policy and Management

**Primary Subject Heading**:	Health economics
Secondary Subject Heading:	Global health, Health economics, Health policy, Obstetrics and gynaecology, Public health
Keywords:	HEALTH ECONOMICS, Epidemiology < ONCOLOGY, PUBLIC HEALTH

SCHOLARONE™
Manuscripts

For peer review only

The Cost-Effectiveness of HPV-Based Cervical Cancer Screening in the Public Health System in Nicaragua

Nicole G. Campos, PhD, [a] Mercy Mvundura, PhD,[b] Jose Jeronimo, MD,[c] Francesca Holme, MPH,[c] Elisabeth Vodicka, MHA,[d] Jane J. Kim, PhD[a]

[a] Center for Health Decision Science, Harvard T.H. Chan School of Public Health, 718 Huntington Avenue, Boston, Massachusetts, USA

[b] PATH, Devices and Tools Program, P.O. Box 900922, Seattle, Washington, USA

[c] PATH, Reproductive Health Global Program, P.O. Box 900922, Seattle, Washington, USA

[d] University of Washington, School of Pharmacy, Seattle, Washington, USA

Corresponding author: Nicole G. Campos, 718 Huntington Avenue, Boston, MA 02115; e-mail: ncampos@hsph.harvard.edu; phone: 617-432-2019; fax: 617-432-0190

Word count (abstract): 300

Word count (text): 4,527

For peer review only

Abstract

Objectives: To evaluate the cost-effectiveness of HPV DNA testing (versus Pap-based screening) for cervical cancer screening in Nicaragua.

Design: A previously developed Monte Carlo simulation model of the natural history of HPV infection and cervical cancer was calibrated to epidemiologic data from Nicaragua. Cost data inputs were derived using a micro-costing approach in Carazo, Chontales, and Chinandega departments; test performance data were from a demonstration project in Masaya department.

Setting: Nicaragua's public health sector facilities.

Participants: Women aged 30 to 59 years.

Interventions: Screening strategies included 1) Pap testing every 3 years, with referral to colposcopy for women with an ASCUS+ result ("Pap"); 2) HPV testing every 5 years, with referral to cryotherapy for HPV-positive eligible women (HPV cryotherapy, or "HPV-Cryo"); 3) HPV testing every 5 years, with referral to triage with visual inspection with acetic acid (VIA) for HPV-positive women ("HPV-VIA"); and 4) HPV testing every 5 years, with referral to Pap testing for HPV-positive women ("HPV-Pap").

Outcome measures: Reduction in lifetime risk of cancer; incremental cost-effectiveness ratios (ICER; 2015 US$ per year of life saved [YLS]).

Results: HPV-based screening strategies were more effective than Pap testing. HPV-Cryo was the least costly and most effective strategy, reducing lifetime cancer risk by 29.5% and outperforming HPV-VIA, HPV-Pap, and Pap only, which reduced cancer risk by 19.4%, 12.2%, and 10.8%, respectively. With an ICER of US$320/YLS, HPV-Cryo every 5 years would be very cost-effective using a threshold based on Nicaragua's per capita GDP of US$2,090. Findings were robust across sensitivity analyses on test performance, coverage, compliance, and cost parameters.

For peer review only - http://bmjopen.bmj.com/site/about/guidelines.xhtml

For peer review only

Conclusions: HPV testing is very cost-effective compared to Pap testing in Nicaragua, due to higher test sensitivity and the relatively lower number of visits required. Increasing compliance with recommended follow-up will further improve the health benefits and value for public health dollars.

For peer review only

For peer review only - http://bmjopen.bmj.com/site/about/guidelines.xhtml

Strengths and limitations of this study:

- Strengths of this study include the use of implementation data from the Scale-Up project to estimate the cost-effectiveness of HPV testing in Nicaragua's public health system. Findings were robust across extensive sensitivity and scenario analyses.
- A limitation of this study is that modeled screening algorithms do not entirely reflect the complex downstream management described by in-country guidelines. While the modeled algorithms reflect the prototypical structure of a screening episode and the type of facility at which visits usually take place, these do not capture variation due to geography or health facility capacity.
- An additional limitation is that while we adopted a micro-costing approach to leverage data from implementation in Nicaragua, individual-level data for each woman were not available; furthermore, we did not have cost data associated with HPV self-collection in community settings, where most self-collection takes place.

Funding statement: This work was supported by the Bill & Melinda Gates Foundation.

Disclaimer: The findings and conclusions contained within are those of the authors and do not necessarily reflect positions or policies of the Bill & Melinda Gates Foundation. The funders had no role in study design; data collection, analysis, and interpretation; preparation of the manuscript; or decision to submit the article for publication.

Competing interests disclosed: All authors have completed the ICMJE uniform disclosure form at www.icmje.org/coi_disclosure.pdf and declare financial support from the Bill and Melinda Gates Foundation for the submitted work; JJ was the director of the START-UP demonstration projects and

For peer review only - http://bmjopen.bmj.com/site/about/guidelines.xhtml

received all tests used in the study as a donation from Qiagen; no other relationships or activities that could appear to have influenced the submitted work.

Contributors: All authors developed the analysis plan and interpreted the data. NC, MM, JJ, FH, and JK conceptualized the study. MM, JJ, FH, and EV collected implementation data. NC conducted data analysis and wrote the first draft of the report and revised subsequent drafts. JJ was the principal investigator of the START-UP and Scale-Up projects. JK was the principal investigator overseeing microsimulation model development. All authors contributed to and approved the final report.

Data sharing statement: PATH provides technical assistance to the government of Nicaragua and has access to screening indicators through partnership with Movicáncer, a local non-governmental organization partner in Nicaragua. Movicáncer designed the health information system used by the Ministry of Health to track screening and treatment of women's cancers. The Ministry of Health protects individually identifiable information; PATH received de-identified and consolidated data on visit compliance from Movicáncer. Costing data were collected by Movicáncer through consultation with Ministry of Health personnel, mostly via phone interviews. No individual identifiers were collected. These data are not available from any public source. A supplementary appendix describing the costing data and microsimulation model is available.

Acknowledgements: We gratefully acknowledge the efforts of Movicáncer of Nicaragua in gathering costing data.

For peer review only

For peer review only - http://bmjopen.bmj.com/site/about/guidelines.xhtml

Background

Cervical cancer is the leading cause of cancer among women in Nicaragua, with an estimated 934 cases and 424 deaths each year [1]. Yet cervical cancer is preventable through screening that allows for early detection and subsequent treatment of precancerous lesions caused by sexually transmitted infection with human papillomavirus (HPV). While most HPV infections clear spontaneously within 1 to 2 years, a persistent infection with one of approximately 15 oncogenic HPV genotypes may progress to precancer, which, if untreated, may become invasive cancer [2, 3].

In most high-income countries, routine screening with cervical cytology (i.e., Pap) testing has substantially reduced the incidence of cervical cancer [4]. However, due to its low sensitivity to detect precancer, Pap testing must be performed at regular frequent intervals in women of screening age. In low- and middle-income settings, where many women do not have access to routine primary health care and even fewer have access to higher-level facilities that offer diagnostic testing and treatment, Pap testing has not been effective at reducing cervical cancer incidence and mortality [5]. In Nicaragua, an estimated 31.5% of women aged 15 to 49 years have been screened within the last year, and nearly 30% of women in this age group have never been screened [6]. One recent survey found that 87% of women in León, Nicaragua, were informed of their Pap results, but of those who were referred to follow-up, only 67% received further care [7].

HPV DNA tests are highly sensitive to detect potentially oncogenic HPV infections and present an alternative to Pap-based screening. Because HPV-negative women are at very low risk for developing cervical cancer within the next 10 years [8], the interval between screenings can be extended to at least 5 years for this subset of women [9]. An additional advantage of HPV testing is that samples can be collected by a provider or by the woman herself, reducing the burden on health workers and time women spend seeking care and potentially increasing screening uptake [10-13]. Furthermore, a lower-cost HPV DNA test known as careHPV has been clinically validated [14, 15] and is now commercialized.

For peer review only

For peer review only - http://bmjopen.bmj.com/site/about/guidelines.xhtml

Given these potential benefits, the World Health Organization (WHO) recommends HPV testing for countries with sufficient resources [9].

In 2011, the Screening Technologies to Advance Rapid Testing for Cervical Cancer Prevention−Utility and Program Planning (START-UP) project in Nicaragua's Masaya department demonstrated that screening with careHPV could be effectively implemented in public sector health facilities [14]. The Ministry of Health of Nicaragua subsequently built on these initial efforts, incorporating HPV testing into public health care systems in three departments with technical assistance from PATH under the Scale-Up project. Adoption of HPV testing within Nicaragua's public health care system is taking place in three phases [16]. In Phase 1, partner organizations worked with the Ministry of Health to prepare for introduction of HPV screening into public health facilities by developing screening and treatment algorithms; creating educational materials; organizing training sessions for health workers and laboratory technicians; and bolstering referral and treatment systems for follow-up of screen-positive women. Phase 2 piloted screening with 10,000 HPV tests in order to identify and address barriers to implementation. Phase 3 will expand coverage to over 50,000 women within one year.

To inform decision makers considering the national adoption and scale-up of HPV testing within Nicaragua's public health sector, this study aimed to 1) estimate the economic cost of cervical cancer screening with careHPV testing; and 2) project the long-term health and economic impact and value (i.e., cost-effectiveness) of careHPV testing in Nicaragua relative to existing Pap-based screening.

Methods

Analytic overview

We used a micro-costing approach to measure and aggregate the cost of all resources used to provide cervical cancer screening at the level of the individual patient within the public health sector in Nicaragua. We considered direct medical costs (i.e., medical resources required for the intervention), direct non-medical costs (i.e., other resources consumed as part of the intervention, such as patient

For peer review only - http://bmjopen.bmj.com/site/about/guidelines.xhtml

transportation costs), and patient time costs (i.e., time spent traveling and waiting for or receiving care). These cost data were input into a previously developed Monte Carlo simulation model (programmed in C++) of the natural history of HPV infection and cervical cancer that was calibrated to epidemiologic data from Nicaragua [17, 18]. We then used the model to project the lifetime health and economic outcomes associated with careHPV testing, using three different algorithms for the management of women who test HPV-positive, and Pap-based screening for women aged 30 to 59 years.

Model outcomes included the lifetime risk of cervical cancer, total lifetime costs per woman (in 2015 United States dollars [US$]), and life expectancy. Incremental cost-effectiveness ratios (ICERs) were calculated by dividing the additional cost of a particular strategy by its additional health benefit, compared with the next most costly strategy. Dominated strategies (defined as more costly and either less effective or having a higher cost-effectiveness ratio than more effective strategies) were eliminated. There is no universal criterion that defines a threshold cost-effectiveness ratio, below which an intervention is considered good value for money; we considered an intervention with an ICER less than Nicaragua’s 2015 per capita GDP of US$2,090 to be “very cost-effective”, and an intervention with an ICER less than three times per capita GDP as “cost-effective” [19]. We followed guidelines for cost-effectiveness by adopting a societal perspective, including costs irrespective of the payer in order to capture the opportunity cost of resources used for the screening intervention. We discounted future costs and life-years at a rate of 3% per year to account for time preferences [20, 21].

Mathematical simulation model

Descriptions of the natural history model of HPV infection and cervical carcinogenesis and model parameterization process have been previously published [17, 18], but we summarize model features here. Individual girls enter the model at age 9 years, prior to initiating sexual activity, and face monthly transitions between mutually exclusive health states that reflect disease progression, including

For peer review only

For peer review only - http://bmjopen.bmj.com/site/about/guidelines.xhtml

type-specific HPV infection, grade of precancer (i.e., cervical intraepithelial neoplasia [CIN] grade 2 or 3), and stage of invasive cancer. Transition probabilities may vary by age, HPV type, duration of infection or precancerous lesion status, prior HPV infection, and exposure to screening and treatment of HPV or precancer. Cervical cancer can be detected through symptoms or screening. Death can occur from non-cervical causes or from cervical cancer after its onset. The model tracks each individual woman's health status, clinical events, and economic outcomes over her lifetime, and aggregates outcomes to estimate the expected costs and health outcomes over the lifetime of the cohort.

The model was calibrated to epidemiologic data on age-specific HPV prevalence and cervical cancer incidence from Nicaragua [1, 14, 17]. We estimated baseline "prior" input parameter values for natural history transitions using available longitudinal data, including age- and type-specific HPV incidence data from Colombia [22-25]. To reflect potential differences in parameters that may vary by setting (i.e., age- and type-specific HPV incidence, natural immunity following initial infection) and uncertainty in progression and regression of precancer, we set plausible bounds around these input values and performed repeated model simulations of disease natural history in the absence of any intervention. Each model simulation selected one random value within the bounds for each uncertain parameter, creating a unique natural history input parameter set. By summing the log-likelihood of model-projected outcomes for each parameter set relative to the epidemiologic data from Nicaragua, we computed a goodness-of-fit score. We selected the 50 top-fitting input parameter sets to use in analysis. Results are reported as the mean across the top 50 parameter sets, and ICERs are reported as the ratio of the mean costs divided by the mean effects of one strategy versus another across sets [26]. Further details on model parameterization, including calibration, are available in the **Appendix**.

Cervical cancer screening strategies

For peer review only - http://bmjopen.bmj.com/site/about/guidelines.xhtml

We considered the following screening strategies for women aged 30 to 59 years (**Figure 1**): 1) Pap testing every 3 years, with referral to colposcopy for all women with an atypical squamous cells of undetermined significance or worse (ASCUS+) result (hereafter referred to as “Pap”); 2) careHPV testing every 5 years, with referral to cryotherapy for all HPV-positive eligible women (HPV cryotherapy, or “HPV-Cryo”); 3) careHPV testing every 5 years, with referral to triage with visual inspection with acetic acid (VIA) for all HPV-positive women (“HPV-VIA”); and 4) careHPV testing every 5 years, with referral to Pap testing for all HPV-positive women (“HPV-Pap”). The pathway of care for each strategy was based on patterns of care in the Scale-Up project, national screening guidelines, and WHO recommendations. We optimistically assumed 70% of women had access to routine screening and attended an initial visit at a screening facility (i.e., a primary health care facility). Women could then return to receive screening results and recommendations for any necessary follow-up care. Follow-up could include colposcopy, cryotherapy, or triage testing at a referral (i.e., higher level) facility, with the exception of Pap triage testing, which was assumed to take place at the screening clinic. At each encounter after the initial screening visit, we assumed 85% of women complied with each subsequent visit to a screening facility, while 40% complied with each subsequent visit to a referral facility, consistent with data from Phase 2 of the Scale-Up project. The minimum number of visits required for treatment in a single screening episode was 4 for Pap, 3 for HPV-Cryo, 3 for HPV-VIA, and 5 for HPV-Pap. In the HPV-VIA and HPV-Pap strategies, women who were HPV-positive but negative on the selected triage test were referred to repeat HPV testing in 1 year. In the HPV-Cryo and HPV-VIA strategies, women who were not eligible for treatment with cryotherapy based on visual assessment were referred to colposcopy with biopsy to rule out cancer; in the absence of cancer, these women were referred to treatment with loop electrosurgical excision procedure (LEEP) or cryotherapy.

Screening and treatment parameters are presented in **Table 1** [6, 14, 17, 27-32]. Screening test performance data were drawn from the START-UP project in Nicaragua to reflect local test

For peer review only - http://bmjopen.bmj.com/site/about/guidelines.xhtml

characteristics. While the START-UP project did not evaluate VIA and Pap as triage tests, we used the VIA and Pap positivity rates in HPV-positive women, along with published studies of triage test performance, to inform triage test sensitivity and specificity [33-38]; in the base case, we optimistically assumed high sensitivity of triage testing.

For all HPV testing strategies, we assumed 20% of women received provider-collection of cervical specimens and 80% of women self-collected vaginal specimens, consistent with the proportions in the Scale-Up project to date. We weighted cost and health outcomes for provider- and self-collection accordingly when aggregating results for the HPV strategies.

Cost data

All costs were converted to 2015 United States dollars (US$) using GDP deflators and the official exchange rate [39]. The direct medical costs of screening, diagnosis, and treatment of precancer were drawn from the START-UP study (Masaya department) and the Scale-Up project (Carazo, Chontales, and Chinandega departments). Direct medical costs included clinical staff time, clinical supplies, drugs, clinical equipment, laboratory staff time, laboratory supplies, and laboratory equipment. Direct non-medical costs included women's round-trip transportation costs to health facilities, and were based on estimates provided by Scale-Up project staff to represent average transportation costs in the Carazo, Chontales, and Chinandega departments. To account for the opportunity cost of women's time spent traveling to, waiting for, or receiving care, we used time estimates from the START-UP and Scale-Up projects, and valued women's time using Nicaragua's monthly minimum wage to serve as a proxy for the societal value of women's time. **Figure 2** displays the categorical breakdown of undiscounted costs for the screening visits over the course of a woman's screening-eligible years, with Pap versus careHPV testing.

For peer review only - http://bmjopen.bmj.com/site/about/guidelines.xhtml

Data on programmatic costs are limited, but for HPV strategies we included the cost of training sessions for health providers offering HPV-based screening, outreach workers, laboratory technicians, and providers offering VIA and cryotherapy. While women who self-collected HPV specimens in the Scale-Up project primarily did so in a community setting, micro-costing data were not available for self-collection performed outside of the clinic, so we assumed clinic-based self-collection. However, we conservatively included the cost of training outreach workers to represent a known programmatic cost as self-collection efforts are shifting to community settings.

Data on the costs of treating cervical cancer were unavailable for Nicaragua, so we estimated direct medical, direct non-medical, and patient and support person time costs using data from El Salvador [29].

Selected cost data are presented in **Table 1.** Further details on cost data are provided in the **Appendix**.

Sensitivity analyses

We performed sensitivity analysis to examine the impact of independently varying uncertain parameters, including Pap test performance, triage test performance in HPV-positive women, colposcopy performance, screening coverage, visit compliance, eligibility for cryotherapy following a positive screening and triage test, treatment effectiveness, discount rate, and cost data. Ranges selected for sensitivity analysis are displayed in **Table 1**.

Scenario analysis

The base case and sensitivity analysis assumed the availability of all strategies (i.e., Pap; HPV-Cryo; HPV-VIA; and HPV-Pap). Additionally, we performed a scenario analysis in which we assumed HPV-

For peer review only - http://bmjopen.bmj.com/site/about/guidelines.xhtml

Cryo was not available for logistical and programmatic reasons (i.e., limited access to cryotherapy equipment and gas).

Results

Base case: Population-level health benefits and cost-effectiveness analysis

HPV-based screening strategies were more effective than Pap testing. Among the HPV strategies, HPV-Cryo (every 5 years) was the most effective strategy; under base case assumptions, it reduced the lifetime risk of cervical cancer by 29.5% on average (range: 25.2%−33.6%). HPV-VIA (every 5 years) reduced cancer risk by 19.4% (range: 16.2%−22.6%), while HPV-Pap reduced cancer risk by 12.2% (range: 10.2%−14.5%). Pap (every 3 years) reduced cancer risk by 10.8% (range: 8.7%−13.4%). Under base case assumptions, HPV-Cryo was both less costly and more effective than all other strategies, thus dominating HPV-VIA, HPV-Pap, and Pap alone. With an ICER of US$320 per year of life saved (YLS), HPV-Cryo every 5 years would be considered "very cost-effective" given Nicaragua's per capita GDP of US$2,090. The total discounted lifetime cost per woman and life expectancy associated with each screening strategy is presented in **Figure 3**.

Sensitivity analysis

While HPV-Cryo remained the most effective strategy across all sensitivity analyses, the magnitude of reduction in lifetime risk of cancer was dependent upon screening coverage of the target population and compliance with recommended follow-up. When coverage was 50% and all other parameters were held constant at base case values, HPV-Cryo reduced cancer risk by an average of 21.1%; HPV-VIA, HPV-Pap, and Pap yielded average cancer risk reductions of 13.9%, 8.7%, and 7.7%, respectively. As coverage increased to 80%, all else being equal, HPV-Cryo reduced cancer risk by an average of 33.5%, while HPV-VIA, HPV-Pap, and Pap yielded average cancer risk reductions of 22.1%,

For peer review only - http://bmjopen.bmj.com/site/about/guidelines.xhtml

14.0%, and 12.4%, respectively (**Appendix**). **Figure 4** displays the impact of visit compliance on lifetime risk of cancer. When compliance with visits to all facilities (i.e., for both screening and referral) was low at 40%, HPV-Cryo remained the most effective strategy, but only reduced cancer risk by 16.2%; Pap had little health impact at this level of compliance, reducing cancer risk by only 5.4%. As compliance at all facilities rose to 85%, HPV-Cryo reduced cancer risk by 47.9%; HPV-VIA, HPV-Pap, and Pap reduced cancer risk by 42.0%, 40.7%, and 35.7% respectively.

In addition to remaining the most effective strategy across all sensitivity analyses, HPV-Cryo remained the most efficient strategy as well. HPV-Cryo remained the least costly and most effective strategy with a stable ICER of US$320 per YLS when 1) Pap test performance (as a primary screening test) improved; 2) VIA and Pap triage test performance improved; 3) colposcopy was assumed to be perfect; and 4) the direct medical cost of LEEP was varied from 75% to 125% of the base case. Despite slight fluctuation in the ICER, HPV-Cryo also remained the least costly and most effective strategy as 1) screening coverage varied from 50% to 80%; 2) visit compliance varied from 40% to 85% per visit; 3) the screen-and-treat cryotherapy cure rate was reduced to 75%; 4) eligibility for cryotherapy was reduced to 75% or less; 5) the treatment cure rate following colposcopy was reduced to 85%; 6) women receiving treatment following colposcopy in the Pap and HPV-Pap strategies were assumed to receive cryotherapy instead of the more costly LEEP; 7) the discount rate was varied from 0% to 5%; 8) only payer costs were considered, excluding women's time and transportation costs; 9) the direct medical cost of HPV self-collection was varied from 75% to 125% of the base case; 10) the direct medical cost of cryotherapy was increased to 170% of the base case; 11) the direct medical cost of colposcopy was reduced to 35% of the base case; 12) programmatic costs associated with HPV-based screening were varied from 50% to 150% of the base case; 13) women's time and transportation costs were reduced to 50% of the base case; and 14) the costs of cancer treatment ranged from including only direct medical costs to 150% of the base case (**Figure 5**). Among variables considered, compliance per visit appears to have the greatest impact

For peer review only - http://bmjopen.bmj.com/site/about/guidelines.xhtml

on the ICER for HPV-Cryo, with 40% compliance yielding an ICER of US$580 per YLS and 85% compliance yielding an ICER of US$190 per YLS. Even when visit compliance is low, HPV-Cryo would be considered very cost-effective.

When only payer costs were considered (i.e., women's time and transportation costs were excluded), the total lifetime cost per woman was lower for all strategies, and Pap every 3 years was slightly less costly (although still slightly less effective) than HPV-Pap every 5 years. HPV-Cryo remained the most effective and efficient strategy, with an ICER of US$270 per YLS (**Appendix**).

The only scenario in which Pap testing every 3 years was the least costly strategy occurred when the direct medical cost of Pap testing was US$3 (base case: US$7.26), a value commonly cited for the cost of Pap in Nicaragua, though the source of this estimate is unknown. However, Pap remained the least effective strategy, and HPV-Cryo had a lower cost-effectiveness ratio, maintaining an ICER of US$320 per YLS (**Appendix**).

Scenario analysis: HPV-Cryo unavailable

When we assumed HPV-Cryo was not available as a screening strategy, HPV-VIA was the least costly and most effective strategy in the base case and most sensitivity analyses, with a base case ICER of US$550 per YLS (**Appendix**). Exceptions included the following circumstances: 1) when the direct medical cost of Pap was US$3, Pap alone had a more attractive ICER (US$530), albeit was less effective than HPV-VIA (US$630 per YLS); 2) Pap performance in the general screening population was improved and Pap alone became the most effective strategy, with an ICER of US$540 per YLS; 3) VIA test sensitivity in HPV-positive women was only 0.40, in which case HPV-VIA had an ICER of US$726 per YLS, but HPV-Pap was more effective with an ICER of US$3,260.

Discussion

For peer review only - http://bmjopen.bmj.com/site/about/guidelines.xhtml

321 Using implementation data from the Scale-Up project— which aims to facilitate
322 institutionalization of HPV testing at the national level in Guatemala, Honduras, and Nicaragua— we
323 estimated the long-term health impact and value of careHPV testing in Nicaragua's public health system.
324 We found that screening algorithms consisting of HPV testing at 5-year intervals would be less costly and
325 more effective than screening with Pap testing at 3-year intervals. Furthermore, HPV testing followed by
326 treatment with cryotherapy for all eligible HPV-positive women would be less costly and more effective
327 than HPV testing followed by triage testing with either VIA or Pap for HPV-positive women. A screen-
328 and-treat HPV program would be a very cost-effective intervention in Nicaragua, with an ICER of US$320
329 per YLS under base case assumptions. These findings were robust across sensitivity analyses. The
330 comparatively large health benefits and efficiency of HPV-Cryo can largely be attributed to the relatively
331 low number of visits to health care facilities and the high sensitivity of the careHPV test to detect both
332 CIN2+ and oncogenic HPV infections with the potential to develop into precancer.

333 We found that screening coverage of the target population had a considerable impact on
334 achievable reductions in cervical cancer risk, with HPV-Cryo yielding the greatest risk reduction. Due to
335 proportional increases in both costs and health benefits, the ICER for HPV-Cryo remained stable as
336 coverage increased from 50% to 80%. Compliance with recommended follow-up was a key driver of
337 both achievable reductions in cancer risk and the ICER of HPV-Cryo. As the proportion of women who
338 returned for each clinical encounter (relative to the previous visit) increased from 40% to 85%, the
339 effectiveness or cancer benefit associated with HPV-Cryo rose from 16.2% to 47.9% as more women
340 were linked to treatment; the ICER fell from US$580 per YLS to US$190 per YLS as more cancers were
341 averted. Thus, improved efforts to successfully navigate women to recommended follow-up will
342 enhance screening program effectiveness and efficiency.

343 While substantially reducing the cost of Pap testing to US$3 (less than half of the base case
344 value) made Pap the strategy with the lowest per-woman lifetime costs, the confluence of low test

For peer review only - http://bmjopen.bmj.com/site/about/guidelines.xhtml

sensitivity and the high number of health facility visits needed to complete screening, diagnostic follow-up, and treatment made Pap the least effective strategy since women are lost to follow-up with each additional required visit. Even in this low-cost Pap scenario, HPV-Cryo remained the most effective and cost-effective strategy.

When we assumed a scenario in which HPV-Cryo was not available for logistic and programming reasons, we found that HPV-VIA was usually the least costly and most effective of the remaining strategies. Although the ICER was less attractive than the ICER associated with HPV-Cryo in the main analysis, it was below Nicaragua's per capita GDP.

There are several limitations to this analysis. We did not consider alternative screening intervals or ages for each strategy, but rather restricted the analysis to the ages and intervals currently under consideration by the Nicaraguan Ministry of Health. It is likely that an increased screening interval and fewer lifetime screens will also be cost-effective, although health benefits may be reduced; we demonstrated in a previous analysis that screening once or three times in a woman's lifetime with careHPV would be very cost-effective in Nicaragua [17]. The screening algorithms, as modeled, reflect the prototypical structure of a screening episode and the type of facility at which visits usually take place, but do not capture variation due to geography or health facility capacity. Furthermore, the modeled screening algorithms do not entirely reflect the complex downstream follow-up of screen-positive women that is embodied in the Ministry of Health's screening guidelines. In simplifying the downstream follow-up for modeled strategies, we may have underestimated the costs and overestimated the benefits relative to the national guidelines, which call for additional follow-up prior to treatment. However, the modeled strategies would likely bias the analysis in favor of Pap and HPV triage strategies. We also did not consider a strategy in which women with ASCUS+ were referred directly to cryotherapy instead of colposcopy because that approach is not recommended by the WHO guidelines,

For peer review only - http://bmjopen.bmj.com/site/about/guidelines.xhtml

or by any professional medical society to the best of our knowledge. We did not consider quality of life impact or the potential disutilities or harms that might be associated with overtreatment.

While we adopted a micro-costing approach to leverage data from the START-UP and Scale-Up projects in Nicaragua, there remain limitations to our cost estimates. First, individual-level data for each woman were not available; thus, our estimates represent average costs in the project populations. Second, we did not have information on the costs associated with HPV self-collection in community settings, where most self-collection takes place. Instead, we assumed all self-collection took place at the clinic. Compared to clinic-based efforts, community-based self-collection may be associated with lower costs for women's time and travel, and higher direct medical and programmatic costs due to outreach worker involvement in facilitating screening and delivering results. Third, our estimates of programmatic costs were restricted to training sessions, and we did not have information on the costs of social mobilization and outreach, patient navigation and support, or infrastructural improvements that would be required to successfully scale-up a screening program. Fourth, we valued women's time based on the minimum wage in Nicaragua. This may be a conservative estimate if most women attending screening are formally employed; conversely, it may overestimate the societal value of women's time spent working in the informal sector or at home. Finally, we extrapolated the cost of cancer treatment using data from El Salvador [29]. Despite these limitations, extensive sensitivity analyses on cost components indicate that HPV-Cryo is robustly the most efficient strategy.

As implementation of HPV testing continues, particularly without triage testing, the health system's capacity to provide cryotherapy will likely need to increase. While the use of triage testing (either with Pap or HPV) reduces the number of cryotherapy procedures performed, we found that the lower sensitivity of triage testing (resulting in more false negatives) led to a decline in health benefits as fewer women with persistent HPV infection and precancer received treatment. The cost savings associated with fewer cryotherapy procedures were outweighed by increased costs of additional follow-

For peer review only - http://bmjopen.bmj.com/site/about/guidelines.xhtml

up and cancer treatment in triage-negative women. A sensitivity analysis on the cost of cryotherapy revealed that HPV-Cryo remained the dominant strategy even when costs increased to 170% of the base case. However, we did not explicitly consider the costs of increasing access to cryotherapy machines or the implications of gas stock-outs, which have been identified as barriers in some low- and middle-income countries [40]. New ablative technologies currently undergoing testing are smaller, portable, and do not require gas. Thermal-coagulation has been used in United Kingdom for more than 30 years, and now it is being used in several low- and middle-income countries, including as part of a “screen-and-treat” program in Malawi [41], and is currently undergoing testing in Latin America. If newer technologies demonstrate cure rates similar to cryotherapy, the cost-effectiveness of screen-and-treat algorithms may improve along with access to treatment.

In summary, using data from the Scale-Up implementation project in Nicaragua, we found that HPV testing followed by cryotherapy for eligible HPV-positive women (a “screen-and-treat” approach) was a very cost-effective intervention in Nicaragua. As the HPV-Cryo algorithm was not implemented in Phase 2 of the Scale-Up project, compliance and cost estimates may need to be further honed to reflect improvements in capacity for cryotherapy if HPV-Cryo is implemented going forward. While it is too early to assume that costs and health impact from Phase 2 of implementation are generalizable to other departments in Nicaragua or other settings in Central America, extensive sensitivity analyses indicate the robustness of findings. An HPV-based screening algorithm involving a similar “screen-and-treat” approach was recently found to be good value for public health dollars in El Salvador [29], where national scale-up is underway. It is important to note that a favorable cost-effectiveness profile does not guarantee that HPV-Cryo will be affordable or feasible in a lower-middle income country like Nicaragua. Both the cost-effectiveness ratio, budgetary impact, and health systems infrastructure need to be favorable for screening programs to be sustainable. We present these findings to inform evidence-based

For peer review only - http://bmjopen.bmj.com/site/about/guidelines.xhtml

415 decision making around national screening guidelines, program design and implementation, and

416 budgeting for infrastructural improvements and procurement of HPV tests in Nicaragua.

For peer review only

For peer review only - http://bmjopen.bmj.com/site/about/guidelines.xhtml

References

1. Ferlay J, Soerjomataram I, Ervik M, et al. *GLOBOCAN 2012 v1.0, Cancer Incidence and Mortality Worldwide: IARC CancerBase No. 11 [Internet]*, 2013, International Agency for Research on Cancer: Lyon, France.
2. Walboomers JM, Jacobs MV, Manos MM, et al. Human papillomavirus is a necessary cause of invasive cervical cancer worldwide. *J Pathol* 1999;189(1):12-19.
3. Rodriguez AC, Schiffman M, Herrero R, et al. Longitudinal study of human papillomavirus persistence and cervical intraepithelial neoplasia grade 2/3: critical role of duration of infection. *J Natl Cancer Inst* 2010;102(5):315-324.
4. Kitchener HC, Castle PE, and Cox JT. Chapter 7: Achievements and limitations of cervical cytology screening. *Vaccine* 2006;24 Suppl 3:S3/63-70.
5. Denny L, Quinn M, and Sankaranarayanan R. Chapter 8: Screening for cervical cancer in developing countries. *Vaccine* 2006;24 Suppl 3:S3/71-77.
6. ICO. *HPV Information Centre*, 2014.
7. Vastbinder MB, Castillo C, and Bekkers RL. Barriers to follow-up of abnormal Papanicolaou smears among women in Leon, Nicaragua. *Trop Doct* 2010;40(1):22-26.
8. Khan MJ, Castle PE, Lorincz AT, et al. The elevated 10-year risk of cervical precancer and cancer in women with human papillomavirus (HPV) type 16 or 18 and the possible utility of type-specific HPV testing in clinical practice. *J Natl Cancer Inst* 2005;97(14):1072-1079.
9. WHO, in *WHO Guidelines for Screening and Treatment of Precancerous Lesions for Cervical Cancer Prevention*2013: Geneva.
10. Arrossi S, Thouyaret L, Herrero R, et al. Effect of self-collection of HPV DNA offered by community health workers at home visits on uptake of screening for cervical cancer (the EMA study): a population-based cluster-randomised trial. *Lancet Glob Health* 2015;3(2):e85-94.
11. Moses E, Pedersen HN, Mitchell SM, et al. Uptake of community-based, self-collected HPV testing vs. visual inspection with acetic acid for cervical cancer screening in Kampala, Uganda: preliminary results of a randomised controlled trial. *Trop Med Int Health* 2015;20(10):1355-1367.
12. Lazcano-Ponce E, Lorincz AT, Cruz-Valdez A, et al. Self-collection of vaginal specimens for human papillomavirus testing in cervical cancer prevention (MARCH): a community-based randomised controlled trial. *Lancet* 2011;378(9806):1868-1873.
13. Racey CS, Withrow DR, and Gesink D. Self-collected HPV testing improves participation in cervical cancer screening: a systematic review and meta-analysis. *Can J Public Health* 2013;104(2):e159-166.
14. Jeronimo J, Bansil P, Lim J, et al. A multicountry evaluation of careHPV testing, visual inspection with acetic acid, and papanicolaou testing for the detection of cervical cancer. *Int J Gynecol Cancer* 2014;24(3):576-585.
15. Qiao YL, Sellors JW, Eder PS, et al. A new HPV-DNA test for cervical-cancer screening in developing regions: a cross-sectional study of clinical accuracy in rural China. *Lancet Oncol* 2008;9(10):929-936.
16. Jeronimo J, Holme F, Slavkovsky R, et al. Implementation of HPV testing in Latin America. *J Clin Virol* 2016;76 Suppl 1:S69-73.
17. Campos NG, Tsu, V., Jeronimo, J., Mvundura, M., Lee, K., Kim, J.J. When and how often to screen for cervical cancer in three low- and middle-income countries: A cost-effectiveness analysis. *Papillomavirus Research* 2015;1:38-58.

For peer review only - http://bmjopen.bmj.com/site/about/guidelines.xhtml

18. Campos NG, Burger EA, Sy S, et al. An updated natural history model of cervical cancer: derivation of model parameters. *Am J Epidemiol* 2014;180(5):545-555.
19. WHO. *Macroeconomics and health: Investing in health for economic development: Report of the Commission on Macroeconomics and Health*, 2001, World Health Organization: Geneva.
20. Tan-Torres Edejer T, Baltussen, R., Adam, T., Hutubessy, R., Acharya, A., Evans, D.B., Murray, C.J.L., ed. *Making Choices in Health: WHO Guide to Cost-Effectiveness Analysis*. 2003, World Health Organization: Geneva, Switzerland.
21. Jamison DT, Breman, J.G., Measham, A.R., Alleyne, G., Claeson, M., Evans, D.B., Jha, P., Mills, A., Musgrove, P., ed. *Disease Control Priorities in Developing Countries*. Second edition ed. 2006, Oxford University Press and The World Bank: Washington, D.C.
22. Munoz N, Mendez F, Posso H, et al. Incidence, duration, and determinants of cervical human papillomavirus infection in a cohort of Colombian women with normal cytological results. *J Infect Dis* 2004;190(12):2077-2087.
23. Herrero R, Hildesheim A, Rodriguez AC, et al. Rationale and design of a community-based double-blind randomized clinical trial of an HPV 16 and 18 vaccine in Guanacaste, Costa Rica. *Vaccine* 2008;26(37):4795-4808.
24. Surveillance, Epidemiology, and End Results (SEER) Program. *SEER*Stat Database: Incidence - SEER 18 Regs Research Data + Hurricane Katrina Impacted Louisiana Cases Nov 2011 Sub (1973-2009 varying) – Linked to County Attributes – Total U.S., 1969-2010 Counties*. Bethesda, MD: National Cancer Institute, Division of Cancer Control and Population Sciences, SUrveillance Research Program, Surveillance Systems Branch; 2012.
25. Sankaranarayanan R, Swaminathan R, Brenner H, et al. Cancer survival in Africa, Asia, and Central America: a population-based study. *Lancet Oncol* 2010;11(2):165-173.
26. Stinnett AA and Paltiel AD. Estimating CE ratios under second-order uncertainty: the mean ratio versus the ratio of means. *Med Decis Making* 1997;17(4):483-489.
27. Mayrand MH, Duarte-Franco E, Rodrigues I, et al. Human papillomavirus DNA versus Papanicolaou screening tests for cervical cancer. *N Engl J Med* 2007;357(16):1579-1588.
28. Ronco G, Dillner J, Elfstrom KM, et al. Efficacy of HPV-based screening for prevention of invasive cervical cancer: follow-up of four European randomised controlled trials. *Lancet* 2014;383(9916):524-532.
29. Campos NG, Maza M, Alfaro K, et al. The comparative and cost-effectiveness of HPV-based cervical cancer screening algorithms in El Salvador. *Int J Cancer* 2015.
30. Chirenje ZM, Rusakaniko S, Kirumbi L, et al. Situation analysis for cervical cancer diagnosis and treatment in east, central and southern African countries. *Bull World Health Organ* 2001;79(2):127-132.
31. Cremer M, Bullard K, Maza M, et al. Cytology versus visual inspection with acetic acid among women treated previously with cryotherapy in a low-resource setting. *Int J Gynaecol Obstet* 2010;111(3):249-252.
32. Sauvaget C, Muwonge R, and Sankaranarayanan R. Meta-analysis of the effectiveness of cryotherapy in the treatment of cervical intraepithelial neoplasia. *Int J Gynaecol Obstet* 2013;120(3):218-223.
33. Bigoni J, Gundar M, Tebeu PM, et al. Cervical cancer screening in sub-Saharan Africa: a randomized trial of VIA versus cytology for triage of HPV-positive women. *Int J Cancer* 2015;137(1):127-134.
34. Catarino R, Vassilakos P, Scaringella S, et al. Smartphone Use for Cervical Cancer Screening in Low-Resource Countries: A Pilot Study Conducted in Madagascar. *PLoS One* 2015;10(7):e0134309.

For peer review only - http://bmjopen.bmj.com/site/about/guidelines.xhtml

35. Pan QJ, Hu SY, Guo HQ, et al. Liquid-based cytology and human papillomavirus testing: a pooled analysis using the data from 13 population-based cervical cancer screening studies from China. *Gynecol Oncol* 2014;133(2):172-179.
36. Qiao YL, Jeronimo J, Zhao FH, et al. Lower cost strategies for triage of human papillomavirus DNA-positive women. *Int J Cancer* 2014;134(12):2891-2901.
37. Tebeu PM, Fokom-Domgue J, Crofts V, et al. Effectiveness of a two-stage strategy with HPV testing followed by visual inspection with acetic acid for cervical cancer screening in a low-income setting. *Int J Cancer* 2015;136(6):E743-750.
38. Richardson LA, El-Zein M, Ramanakumar AV, et al. HPV DNA testing with cytology triage in cervical cancer screening: Influence of revealing HPV infection status. *Cancer Cytopathol* 2015;123(12):745-754.
39. *World Development Indicators*, 2016, World Bank.
40. Msyamboza KP, Phiri T, Sichali W, et al. Cervical cancer screening uptake and challenges in Malawi from 2011 to 2015: retrospective cohort study. *BMC Public Health* 2016;16(1):806.
41. Campbell C, Kafwafwa S, Brown H, et al. Use of thermo-coagulation as an alternative treatment modality in a 'screen and treat' programme of cervical screening in rural Malawi. *Int J Cancer* 2016.
42. Gage JC, Rodriguez AC, Schiffman M, et al. Treatability by cryotherapy in a screen-and-treat strategy. *J Low Genit Tract Dis* 2009;13(3):174-181.
43. Starks D, Arriba LN, Enerson CL, et al. Mexican Cervical Cancer Screening Study II: 6-month and 2-year follow-up of HR-HPV women treated with cryotherapy in a low-resource setting. *J Low Genit Tract Dis* 2014;18(4):333-337.
44. McClung EC and Blumenthal PD. Efficacy, safety, acceptability and affordability of cryotherapy: a review of current literature. *Minerva Ginecol* 2012;64(2):149-171.

For peer review only - http://bmjopen.bmj.com/site/about/guidelines.xhtml

Table 1. Baseline values and ranges for model variables.[a]

Test/Parameter	Base Case:	Sensitivity Analysis:
Screening/triage test performance (sensitivity/specificity to detect CIN2+)		
Pap (primary) [14, 27, 28]	0.41/0.94	Alternative sensitivity/specificity pair:<br>0.70/0.95
careHPV (primary), provider-collection of cervical samples **[14]**	0.78/0.89	--
careHPV (primary), self-collection of vaginal samples **[14]**	0.67/0.86	--
VIA (triage of HPV+) **[14, 33, 34, 36, 37]**	0.60/0.75	Alternative sensitivity/specificity pairs:<br>0.40/0.85<br>0.70/0.65
Pap (triage of HPV+) **[14, 33, 35, 38]**	0.85/0.55	Alternative sensitivity/specificity pairs:<br>0.40/0.85<br>0.90/0.50
Colposcopy performance (sensitivity/specificity to detect CIN1+) **[14, 17]**[b]		
Colposcopy (ASCUS+ women)	0.95/0.68	1.0/1.0
Colposcopy (HPV+ women ineligible for cryotherapy)	Upon ruling out cancer, all referred to treatment	0.95/0.68, with <CIN1 sent back to routine screening intervals
Colposcopy (HPV+/VIA+ women ineligible for ST cryotherapy)	Upon ruling out cancer, all referred to treatment	0.95/0.68, with <CIN1 sent back to routine screening intervals
Colposcopy (HPV+/Pap+ women)	Upon ruling out cancer, all referred to treatment	0.95/0.68, with <CIN1 sent back to routine screening intervals
Coverage and compliance[c]		
Access to routine screening, % of the target population **[6]**	70%	50% - 80%
Visit compliance, screening facility[d]	85%	40% - 85%
Visit compliance, referral facility[d]	40%	40% - 85%
Treatment eligibility and efficacy		
Eligibility for screen-and-treat cryotherapy **[29, 42]**	≤CIN1: 100%<br>CIN2: 85%	≤CIN1: 75%<br>CIN2: 60%

For peer review only

	CIN3: 75%	CIN3: 49%
Screen-and-treat cryotherapy cure rate (for HPV+ or HPV+/VIA+) **[30-32]**	92%	75%
Proportion of women maintaining an HPV infection following cryotherapy **[43]**	15%	--
Treatment cure rate following colposcopy (LEEP for CIN2+; else cryotherapy)**[30]**	96%	85%
Proportion of women maintaining an HPV infection following colposcopic diagnosis and treatment **[44]**	10%	--
Discount rate for costs and life-years	3%	0% - 5%
Direct medical costs, screening and treatment of precancer (2015 US$)**[14]**[d]		
Pap test	7.26	3
careHPV test (provider-collection)	11.96	--
careHPV test (self-collection)	11.04	75% - 125% of base case
VIA triage test	4.19	--
Colposcopy/biopsy	19.91	6.91[e]
Cryotherapy	18.16	30.54[f]
LEEP	68.36	75% - 125% of base case
Women's time and transportation costs (2015 US$)[d]		
Transportation to screening facility (round-trip)	0.41	0% to 50% of base case
Transportation to referral facility (round trip)	2.81	0% to 50% of base case
Wait time, screening facility	0.48	0% to 50% of base case
Wait time, referral facility	1.75	0% to 50% of base case
Transport time, screening facility	0.82	0% to 50% of base case
Transport time, referral facility	3.10	0% to 50% of base case
Programmatic costs, training (2015 US$)[d]		
Health care personnel (careHPV)	0.09 per woman screened with careHPV	50% - 150% of base case
Laboratory technicians (careHPV)	0.04 per woman screened with careHPV	50% - 150% of base case
Outreach workers/auxiliary nurses (careHPV self-collection)	0.08 per woman screened with careHPV (self-collection)	50% - 150% of base case
Health care providers (VIA and cryotherapy)	1.51 per woman receiving VIA and/or cryotherapy	50% - 150% of base case

For peer review only - http://bmjopen.bmj.com/site/about/guidelines.xhtml

Cost of cancer treatment (2015 US$) (rounded) **[29]**		
Local cancer		
- Direct medical	944	
- Direct non-medical[g]	197	
- Women's time[h]	346	
Total	1,486	944 - 2,229 (direct medical only; 150% of base case)
Regional and distant cancer		
- Direct medical	918	
- Direct non-medical[g]	390	
- Women's time[h]	640	
Total	1,946	918 - 2,920 (direct medical only; 150% of base case)

[a] ASCUS+: atypical squamous cells of undetermined significance or higher; CIN 1+: cervical intraepithelial neoplasia grade 1 or higher; CIN2+: cervical intraepithelial neoplasia grade 2 or higher; HPV: human papillomavirus; LEEP: loop electrosurgical excision procedure; ST: screen-and-treat; US$: 2015 United States dollars; VIA: visual inspection with acetic acid. Parameter values for sensitivity analysis were determined as follows: screening and triage test performance (cited literature); colposcopy performance (assumption); coverage and compliance (assumptions); treatment eligibility and efficacy (cited literature); discount rate (assumptions); direct medical costs (assumptions, with the exception of colposcopy and cryotherapy, which were based on ranges suggested by Scale-Up and START-UP data); women's costs (assumptions); programmatic costsw (assumptions); cancer costs (assumptions).

[b] Test performance characteristics of colposcopy in the START-UP demonstration project were derived from the worst diagnosis of the local pathologist relative to the worst diagnosis by a quality control pathologist (gold standard); we applied the treatment threshold of CIN1+, although this was not the treatment threshold in START-UP. To derive test performance of colposcopy, we excluded histological classifications that were inadequate or with a histological classification other than negative, CIN1, CIN2, CIN3, or cancer. Because CIN1 is not a true underlying health state in the microsimulation model, performance of colposcopy in the model is based on the underlying health states of no lesion, HPV infection, CIN2, or CIN3. For a treatment threshold of CIN1, we weighted sensitivity of colposcopy for women with HPV based on the country-specific prevalence of CIN1 among women with HPV infections in the START-UP studies.

[c] Compliance is defined as the proportion of women who return for each clinical encounter, relative to the previous visit.

[d] Unpublished data from the Scale-Up Nicaragua project. Further details on costing data are provided in the Appendix.

[e] In sensitivity analysis, we considered the direct medical cost of colposcopy to be equivalent to the cost of colposcopy alone, without biopsy.

[f] In sensitivity analysis, we considered the direct medical cost of cryotherapy to include the upper bound of cryotherapy equipment costs (assuming the lowest number of women treated per year per facility, in Carazo, Chontales, or Chinandega) and the upper bound of cryotherapy supply costs (assuming the lowest number of women treated per gas tank in any facility in Carazo, Chontales, or Chinandega).

[g] Direct non-medical costs include transportation to a tertiary facility, temporary housing, and meals.

[h] Includes woman's time an support person's time.

For peer review only - http://bmjopen.bmj.com/site/about/guidelines.xhtml

Figure legends.

Figure 1. Pathways of care, by screening strategy. Each diagram indicates the flow of screening-eligible women (i.e., women aged 30 to 59 years) through each point of contact in a screening episode, conditional on visit compliance and test results, for a) Pap testing every 3 years (Pap), which requires 4 visits for screening, diagnosis, and treatment; b) HPV screen-and-treat every 5 years (HPV-Cryo), which requires 3 or more visits for screening and necessary treatment; c) HPV testing followed by visual inspection with acetic acid (VIA) triage of HPV-positive women every 5 years (HPV-VIA), which requires 3 or more visits for screening and necessary treatment; and d) HPV testing followed by Pap triage of HPV-positive women every 5 years (HPV-Pap), which requires 5 or more visits for screening and necessary treatment. ASCUS+: Pap result of atypical squamous cells of undetermined significance or worse; CIN: cervical intraepithelial neoplasia; HPV: human papillomavirus; LEEP: loop electrosurgical excision procedure; VIA: visual inspection with acetic acid.

Figure 2. Cervical cancer screening cost per woman over duration of screening eligibility, by cost component: Pap testing (every 3 years) versus careHPV testing (every 5 years). Bars indicate the undiscounted cost (2015 US$) of screening with Pap testing (offered 10 times between ages 30 and 59 years) versus careHPV testing (offered 6 times between ages 30 and 59 years), by cost component. Only screening costs are shown; costs associated with recommended management following a positive screening test are not included. 6x: delivered 6 times over the course of screening eligible ages 30 to 59; 10x: delivered 10 times over the course of screening eligible ages.

Figure 3. Cost-effectiveness analysis: Base case results. The graph displays the discounted lifetime costs (x-axis; in 2015 US$) and life expectancy (y-axis) associated with each screening strategy (Pap testing every 3 years; careHPV screen-and-treat every 5 years [HPV-Cryo]; careHPV every 5 years with visual inspection with acetic acid [VIA] triage of HPV-positive women [HPV-VIA]; and careHPV every 5 years with Pap triage of HPV-positive women [HPV-Pap]), under base case assumptions. The cost-effectiveness

For peer review only - http://bmjopen.bmj.com/site/about/guidelines.xhtml

associated with a change from one strategy to a more costly alternative is represented by the difference in cost divided by the difference in life expectancy associated with the two strategies. The curve indicates the strategies that are efficient because they are more effective and either 1) cost less; or 2) have a more attractive cost-effectiveness ratio than less effective options. The incremental cost-effectiveness ratio (ICER) is the reciprocal of the slope of the line connecting the two strategies under comparison. In the base case analysis, HPV-Cryo every 5 years was less costly and more effective than other screening strategies considered, and was thus a dominant strategy with an ICER of US$320 per year of life saved. HPV-Pap: HPV testing with Pap triage of HPV-positive women; HPV-Cryo: HPV screen-and-treat strategy; HPV-VIA: HPV testing with visual inspection with acetic acid (VIA) triage of HPV-positive women; yrs: years.

Figure 4. Reduction in lifetime risk of cervical cancer, by compliance level. Bars indicate the percent reduction in lifetime risk of cervical cancer for each screening strategy (Pap testing every 3 years; careHPV screen-and-treat every 5 years [HPV-Cryo]; careHPV every 5 years with visual inspection with acetic acid [VIA] triage of HPV-positive women [HPV-VIA]; and careHPV every 5 years with Pap triage of HPV-positive women [HPV-Pap]) as compliance per visit within a screening episode increases. Compliance is defined as the proportion of women who return for each clinical encounter, relative to the previous visit. Coverage of the target population is assumed to be 70%. While the base case analysis assumed 85% compliance for visits at screening facilities and 40% compliance for visits at referral facilities (for diagnosis and treatment), the graph displays cancer risk reduction assuming the specified compliance level at all visits, regardless of facility type.

Figure 5. Base case and sensitivity analysis: Incremental cost-effectiveness ratios, HPV cryotherapy (HPV-Cryo) strategy. Incremental cost-effectiveness ratios (ICERs) are presented (x-axis, 2015 US$ per year of life saved) for the base case and sensitivity analyses (y-axis). The blue bars represent the range of the ICERs for HPV-Cryo every 5 years across the 50 input parameter sets, with the ICER of the mean

For peer review only - http://bmjopen.bmj.com/site/about/guidelines.xhtml

costs divided by the mean effects demarcated by a black line. The dashed blue line indicates Nicaragua's per capita gross domestic product (GDP), at US$2,090, assuming this is the threshold that designates interventions as "very cost-effective".

For peer review only

For peer review only - http://bmjopen.bmj.com/site/about/guidelines.xhtml

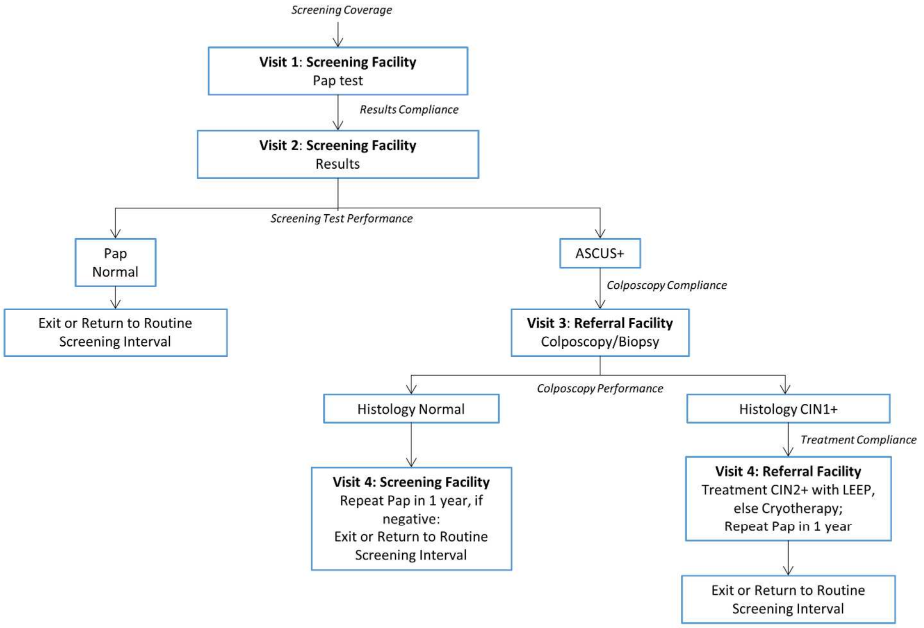

Figure 1. Pathways of care, by screening strategy. Each diagram indicates the flow of screening-eligible women (i.e., women aged 30 to 59 years) through each point of contact in a screening episode, conditional on visit compliance and test results, for a) Pap testing every 3 years (Pap), which requires 4 visits for screening, diagnosis, and treatment; b) HPV screen-and-treat every 5 years (HPV-STHPV-Cryo), which requires 3 or more visits for screening and necessary treatment; c) HPV testing followed by visual inspection with acetic acid (VIA) triage of HPV-positive women every 5 years (HPV-VIA), which requires 3 or more visits for screening and necessary treatment; and d) HPV testing followed by Pap triage of HPV-positive women every 5 years (HPV-Pap), which requires 5 or more visits for screening and necessary treatment. ASCUS+: Pap result of atypical squamous cells of undetermined significance or worse; CIN: cervical intraepithelial neoplasia; HPV: human papillomavirus; LEEP: loop electrosurgical excision procedure; VIA: visual inspection with acetic acid.

254x190mm (300 x 300 DPI)

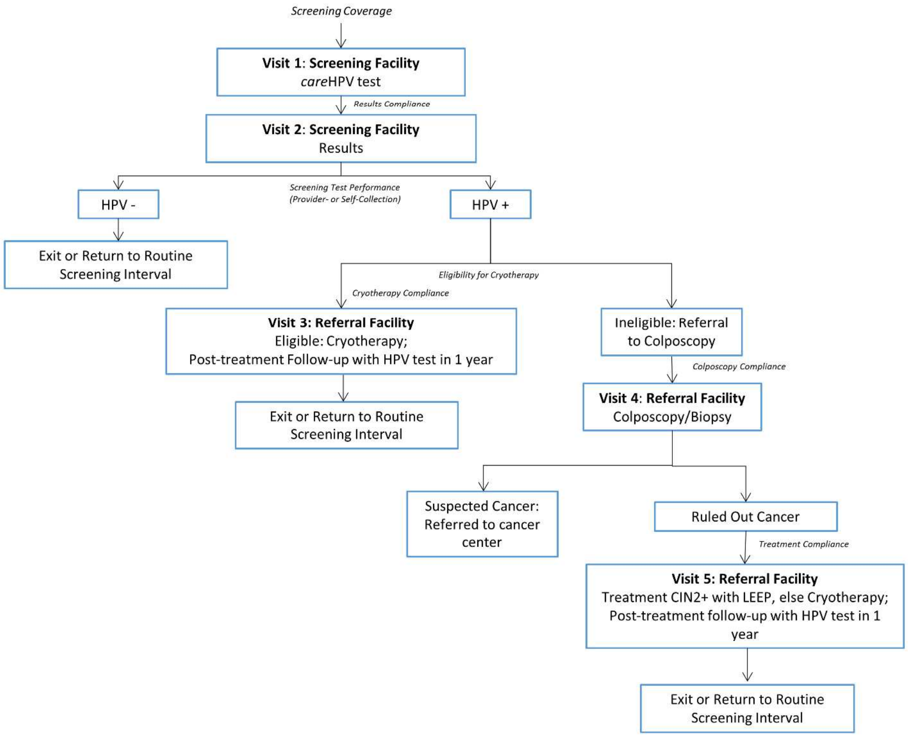

Figure 1. Pathways of care, by screening strategy. Each diagram indicates the flow of screening-eligible women (i.e., women aged 30 to 59 years) through each point of contact in a screening episode, conditional on visit compliance and test results, for a) Pap testing every 3 years (Pap), which requires 4 visits for screening, diagnosis, and treatment; b) HPV screen-and-treat every 5 years (HPV-Cryo), which requires 3 or more visits for screening and necessary treatment; c) HPV testing followed by visual inspection with acetic acid (VIA) triage of HPV-positive women every 5 years (HPV-VIA), which requires 3 or more visits for screening and necessary treatment; and d) HPV testing followed by Pap triage of HPV-positive women every 5 years (HPV-Pap), which requires 5 or more visits for screening and necessary treatment. ASCUS+: Pap result of atypical squamous cells of undetermined significance or worse; CIN: cervical intraepithelial neoplasia; HPV: human papillomavirus; LEEP: loop electrosurgical excision procedure; VIA: visual inspection with acetic acid.

254x190mm (300 x 300 DPI)

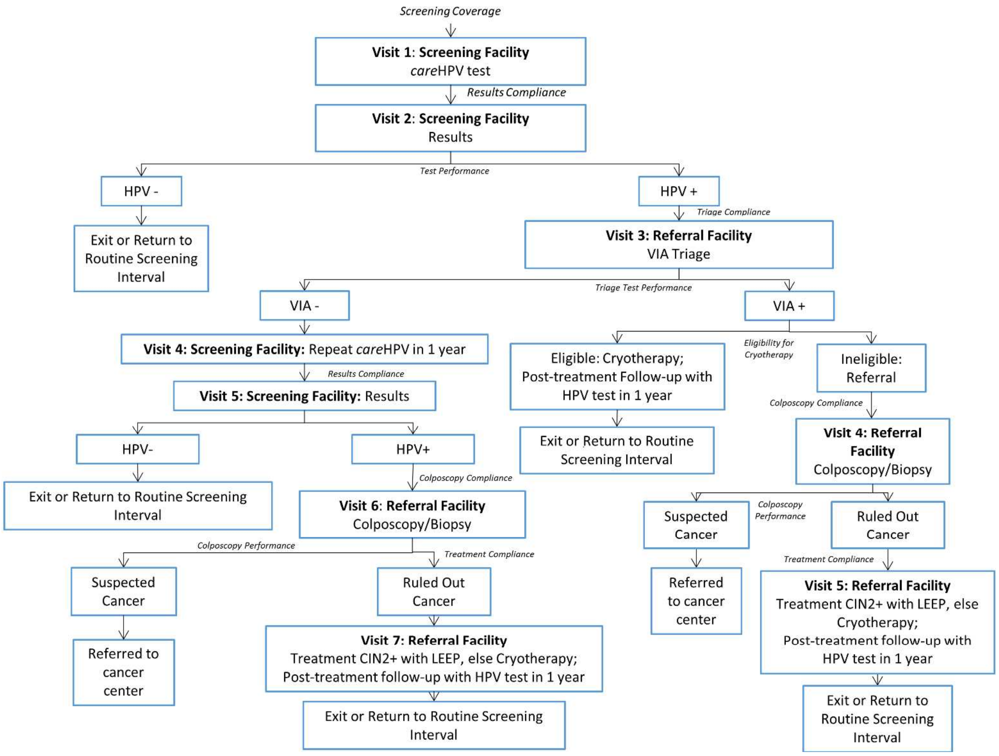

Figure 1. Pathways of care, by screening strategy. Each diagram indicates the flow of screening-eligible women (i.e., women aged 30 to 59 years) through each point of contact in a screening episode, conditional on visit compliance and test results, for a) Pap testing every 3 years (Pap), which requires 4 visits for screening, diagnosis, and treatment; b) HPV screen-and-treat every 5 years (HPV-Cryo), which requires 3 or more visits for screening and necessary treatment; c) HPV testing followed by visual inspection with acetic acid (VIA) triage of HPV-positive women every 5 years (HPV-VIA), which requires 3 or more visits for screening and necessary treatment; and d) HPV testing followed by Pap triage of HPV-positive women every 5 years (HPV-Pap), which requires 5 or more visits for screening and necessary treatment. ASCUS+: Pap result of atypical squamous cells of undetermined significance or worse; CIN: cervical intraepithelial neoplasia; HPV: human papillomavirus; LEEP: loop electrosurgical excision procedure; VIA: visual inspection with acetic acid.

254x190mm (300 x 300 DPI)

For peer review only - http://bmjopen.bmj.com/site/about/guidelines.xhtml

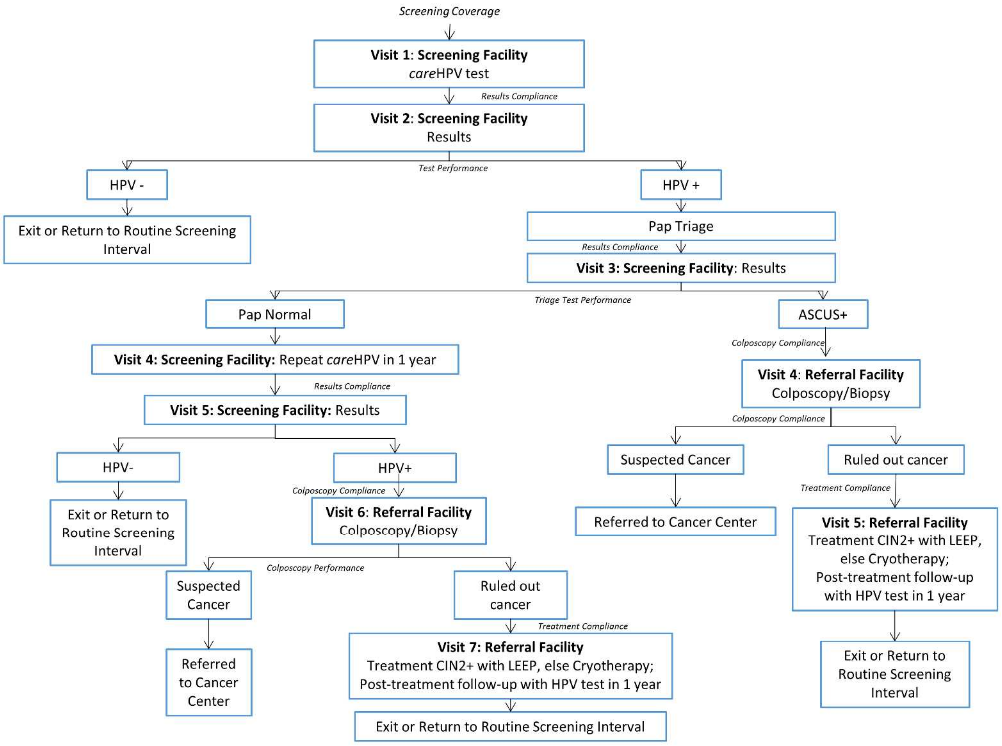

Figure 1. Pathways of care, by screening strategy. Each diagram indicates the flow of screening-eligible women (i.e., women aged 30 to 59 years) through each point of contact in a screening episode, conditional on visit compliance and test results, for a) Pap testing every 3 years (Pap), which requires 4 visits for screening, diagnosis, and treatment; b) HPV screen-and-treat every 5 years (HPV-Cryo), which requires 3 or more visits for screening and necessary treatment; c) HPV testing followed by visual inspection with acetic acid (VIA) triage of HPV-positive women every 5 years (HPV-VIA), which requires 3 or more visits for screening and necessary treatment; and d) HPV testing followed by Pap triage of HPV-positive women every 5 years (HPV-Pap), which requires 5 or more visits for screening and necessary treatment. ASCUS+: Pap result of atypical squamous cells of undetermined significance or worse; CIN: cervical intraepithelial neoplasia; HPV: human papillomavirus; LEEP: loop electrosurgical excision procedure; VIA: visual inspection with acetic acid.

254x190mm (300 x 300 DPI)

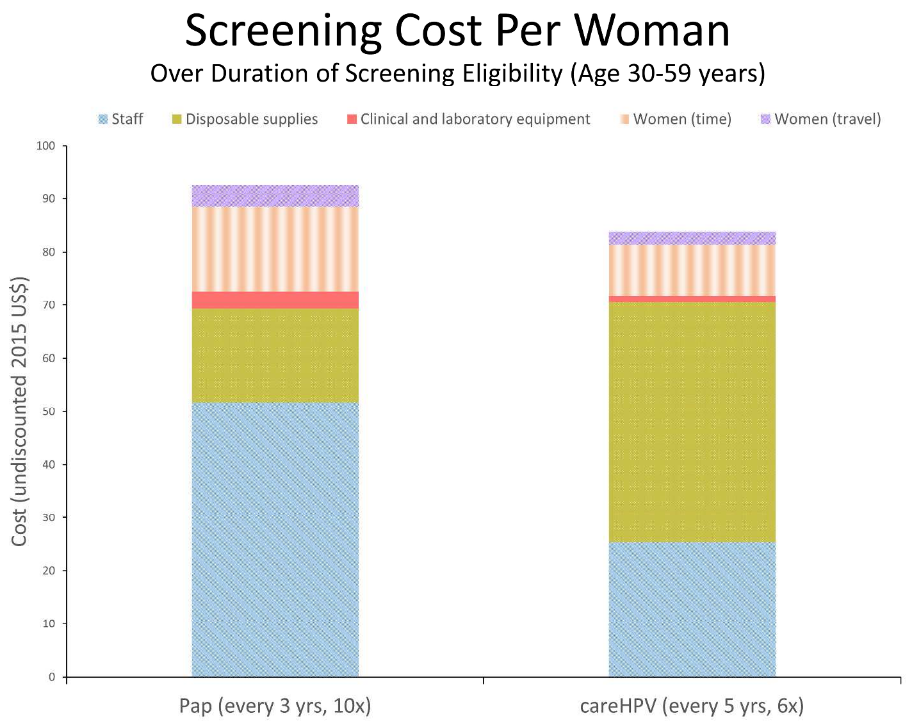

254x190mm (300 x 300 DPI)

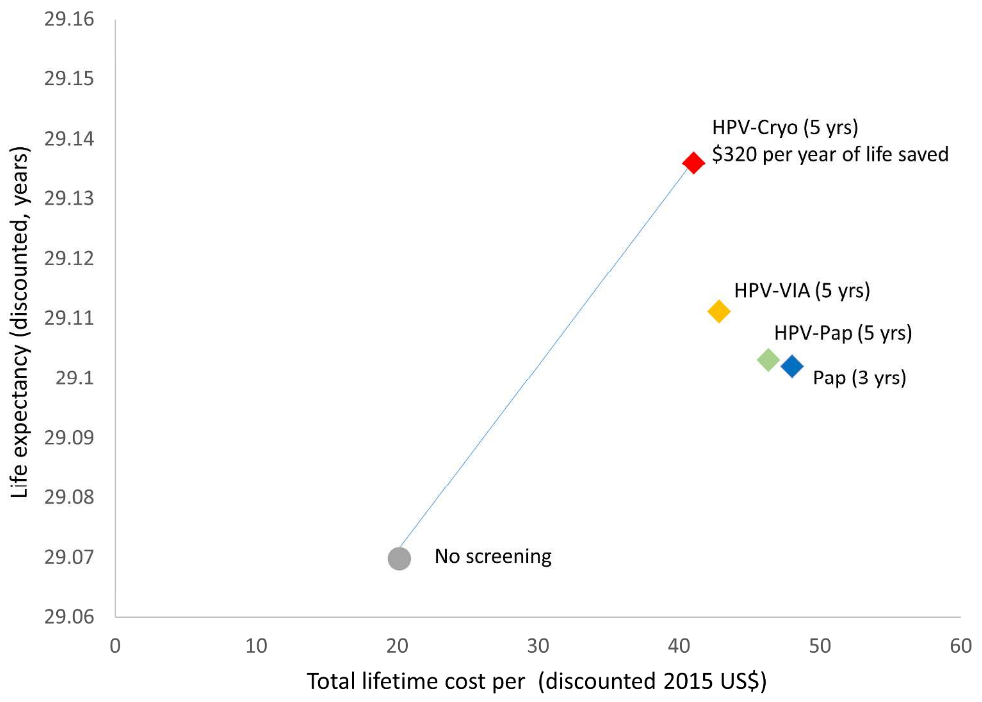

254x190mm (300 x 300 DPI)

For peer review only

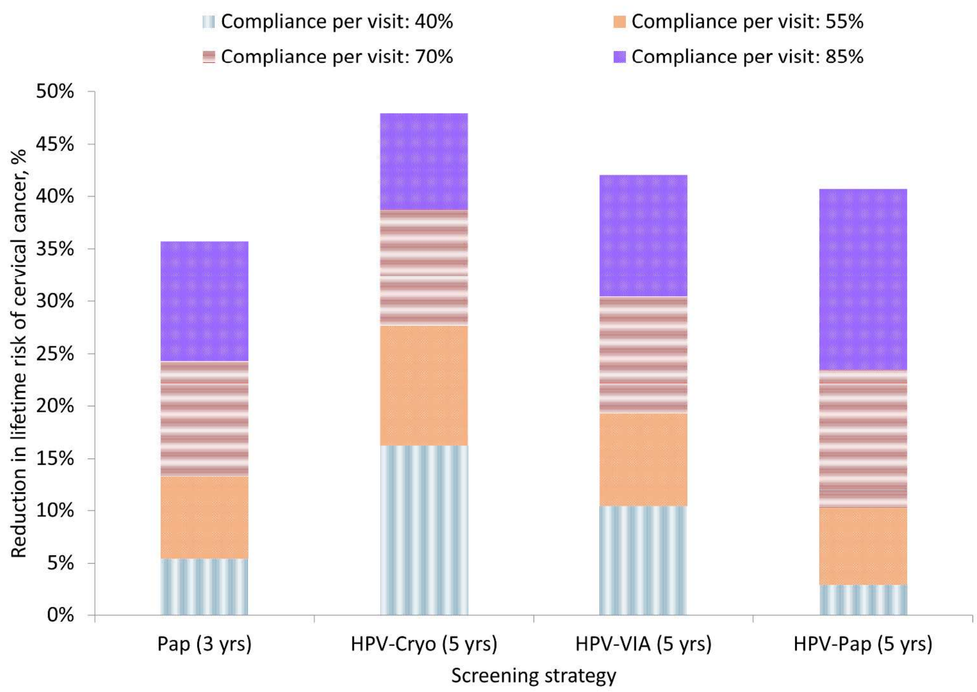

254x190mm (300 x 300 DPI)

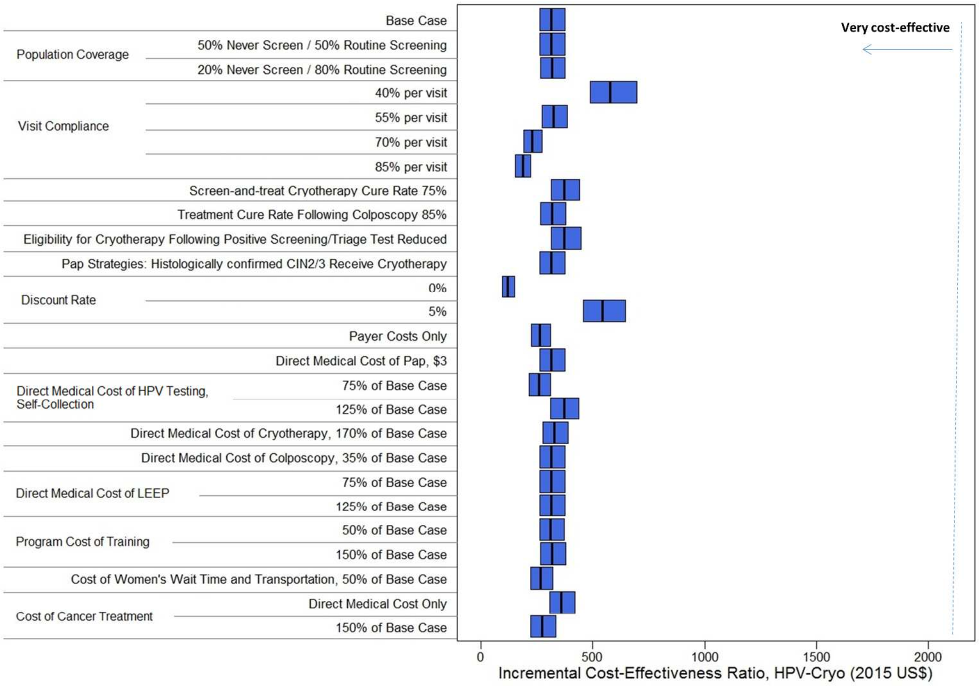

254x190mm (300 x 300 DPI)

Supplemental Appendix:
The Cost-Effectiveness of HPV-Based Screening in the Public Health System in Nicaragua

MODEL CALIBRATION

Overview of the calibration process

Details of model development for the U.S. and Nicaragua models, including initial parameterization and calibration, have been published elsewhere [1, 2]. Baseline HPV incidence rates, as a function of genotype and age, were derived from a published prospective study of sexually active women aged 15-85 years in Bogota, Colombia [3]. Because HPV incidence is not necessarily similar in Colombia and Nicaragua due to differences in sexual behavior, we considered age-specific HPV incidence and natural immunity following initial infection as candidate parameters for calibration. Type-specific data on CIN2 and CIN3 regression and progression are limited [4-9], so these highly uncertain parameters were also candidates for calibration. Time-dependent rates of HPV clearance and progression by genotype were informed by primary longitudinal data from the control arm of the Costa Rica Vaccine Trial [10], assuming that rates of HPV clearance and progression to precancer are similar across populations and vary only by time since infection and HPV genotype.

To calibrate the model, we selected empirical epidemiologic data from Nicaragua, including age-specific prevalence of high-risk HPV and age-specific cancer incidence (see below for further detail). We then set plausible search ranges around baseline model input values for age- and type-specific HPV incidence; natural immunity following initial infection; and progression and regression of CIN. We performed repeated model simulations in the absence of any preventive intervention (i.e., natural history simulations). For each simulation, we randomly selected a single value for each of the uncertain parameters from the identified search range, creating a unique vector of parameter values (i.e., parameter "set"). From over 1,475,000 repeated samplings, we identified the parameter sets with the highest correspondence to the empirical calibration target data from Nicaragua by calculating and aggregating the log-likelihood of model-projected outcomes. We used the 50 parameter sets with the highest likelihood scores (i.e., best overall fit to the empirical data from Nicaragua) for analysis to capture uncertainty in the model parameters as a form of probabilistic sensitivity analysis. We report results as a mean of outcomes across these top 50 parameter sets; incremental cost-effectiveness ratios are reported as the ratio of the mean costs divided by the mean effects of one strategy versus another across sets.

Calibration targets

We assessed model fit by comparing projected model outcomes of age-specific prevalence of high-risk HPV and age-specific cancer incidence relative to empirical data from Nicaragua. The scoring algorithm included age-specific prevalence of high-risk HPV and age-specific cervical cancer incidence.

Age-specific prevalence of high-risk HPV was drawn from the Screening Technologies to Advance Rapid Testing for Cervical Cancer Prevention–Utility and Program Planning (START-UP) project data on *care*HPV positivity using a cut-off ratio cut-point of 0.5 relative light units (**Table A.1**). For each age group, we derived a 95% binomial confidence interval around the point prevalence, which comprised the calibration target. The likelihood function for each age group was assumed to follow a binomial distribution.

Age-specific cancer incidence was drawn from Globocan due to the lack of a local cancer registry [11] (**Table A.2**). The likelihood function for each age group was assumed to follow a normal distribution.

For peer review only - http://bmjopen.bmj.com/site/about/guidelines.xhtml

Composite goodness-of-fit scores for each input parameter set were generated by summing the log likelihood of each model outcome (i.e., age-specific HPV prevalence, age-specific cancer incidence). The 50 input parameter sets with the highest goodness-of-fit scores yielded the model outputs that were simultaneously closest to all calibration targets, and these were selected for analysis. **Figures A.1** and **A.2** display model fit to epidemiologic data on age-specific prevalence of high-risk HPV and age-specific cancer incidence.

Transition probabilities for the calibrated natural history model are presented in **Table A.3.**

COST DATA

Direct Medical Costs: Screening, Diagnosis, and Treatment of Precancerous Lesions

The direct medical costs of screening, diagnosis, and treatment of precancerous lesions were drawn from the START-UP demonstration study in Masaya department and from the Scale-Up Nicaragua Project in the departments of Carazo, Chontales, and Chinandega. Direct medical costs included clinical staff time, clinical supplies, drugs, clinical equipment, laboratory staff time, laboratory supplies, and laboratory equipment. Costs from the START-UP study were collected in 2010 local currency units and inflated to year 2015 levels using Nicaragua GDP deflators and then converted to US$ using the official exchange rate [12]; the exception was for equipment, which was generally procured in the United States and was inflated to year 2015 levels using US GDP deflators. Costs from the Scale-Up Nicaragua project (including equipment) were collected in 2015 local currency units and converted to US$ using the official exchange rate [12].

Costs are reported in **Table A.4** (screening procedures) and **Table A.5** (precancer diagnostic and treatment procedures).

Women's Time and Transportation Costs

To account for the opportunity cost of women's time spent traveling to, waiting for, or receiving care, we valued women's time using Nicaragua's 2013 monthly minimum wage [13], converted to 2015 US$. While the minimum wage may be a conservative estimate for valuation of women's time if they are formally employed, it can serve as a proxy for the societal value of women's time spent working in the informal sector or at home. We converted this to an hourly wage rate assuming 171 hours of work per month (40 hours per week).

Estimates for time spent traveling to, waiting for, and receiving care were dependent upon the facility level where care was assumed to take place (**Table A.6**). While screening and delivery of primary screening results were assumed to take place at basic primary health facilities, we assumed cryotherapy and LEEP treatment were only provided at referral facilities. Estimates of women's time spent receiving a procedure were based on site-specific data from the START-UP demonstration projects, with staff time spent on the procedure (excluding preparation and registration time, which we assumed were built into patient waiting time) used as a proxy for women's procedure time. Estimates of women's wait time for screening and delivery of primary screening results was based on the average wait time for screening at facilities in the Chinandega department; wait time for cryotherapy, colposcopy, and LEEP was based on the average wait time at referral centers in the Chontales department. Round-trip transportation time was based on Scale-Up project staff estimates of the typical amount of time women spent traveling to screening or referral facilities, using a sample of 9 facilities the Carazo department and 10 facilities in the Chontales and Chinandega departments. We averaged across screening and referral facilities within

For peer review only - http://bmjopen.bmj.com/site/about/guidelines.xhtml

each department to obtain a department average, and then averaged across departments to obtain average round-trip transportation times based on facility level. Estimates of women's time and time costs are presented in **Table A.7.**

Women's transportation costs were based on Scale-Up project staff reports of the typical mode of transportation to a particular facility by women in the catchment area, using a sample of 9 facilities in Carazo and 10 facilities each in Chontales or Chinandega. We used the most commonly reported mode of transportation for each department (for screening facilities: walking for Carazo; walking for Chontales; bus for Chinandega; for referral facilities: bus in all three departments). The cost of a bus trip was based on typical bus fare for the distance to the health facility, as reported by Scale-Up project staff. We averaged the typical transportation costs across departments to obtain an estimate of women's round-trip transportation costs to and from a health facility, assuming the same mode of transport was used for the trip to and from the health facility. Women's transportation costs are presented in **Table A.8.**

Programmatic Costs

We included the programmatic cost of the following types of training sessions that were conducted during the Scale-Up project: 1) for health care personnel to offer careHPV screening and counsel women on results; 2) for laboratory technicians to run the careHPV assay; 3) for community outreach workers and auxiliary nurses to promote careHPV screening and offer self-collection of HPV samples; and 4) for health care providers to offer VIA and cryotherapy. We included the cost of trainer time and trainee time (valued using average monthly salaries converted to an hourly rate; volunteer outreach worker time was valued using the minimum wage), as well as the cost of supplies, materials, transportation, and refreshments. To derive an average health care personnel training cost per woman screened, we divided the total training cost for health care personnel by 100,000, corresponding to the number of women screened during the Scale-Up project. To derive an average laboratory technician training cost per woman screened, we divided the total training cost for laboratory technicians by 100,000. To derive an average community outreach worker training cost per woman screened, we divided the total training cost for outreach workers by 80,000, corresponding to the number of women who participated in self-collection of HPV samples. To derive an average health care provider training cost per woman receiving VIA and cryotherapy, we divided the total VIA/cryotherapy training cost for health care providers by 16,000, corresponding to the approximate number of women who tested HPV-positive in Scale-Up and would thus require visual assessment (to determine eligibility for cryotherapy) and, if eligible, cryotherapy.

All women screened with HPV testing incurred the per-woman training costs for health care personnel and laboratory technicians. Only women screened with HPV self-collection incurred the per-woman cost of training outreach workers to offer self-collection (due to lack of data on self-collection in a community setting, in the base case analysis we assumed self-collection took place at the clinic; however, we assumed outreach workers would be involved in mobilizing and educating women about self-collection, and conservatively included the cost of training so as to represent a known programmatic cost as self-collection efforts shift to also include community settings). Only women who received either VIA (as triage) or screen-and-treat cryotherapy incurred the per-woman cost of training health providers to perform VIA and cryotherapy. Training costs are presented in **Table A.9.**

Cost of Cancer Care by Stage

Costs associated with cancer care by stage (Local versus Regional or Distant), including direct medical costs, direct non-medical costs (i.e., transport to a cancer center, and meals and temporary housing

For peer review only - http://bmjopen.bmj.com/site/about/guidelines.xhtml

during the course of treatment), and patient and support person time were derived from a previous analysis in El Salvador [14]. Based on expert opinion in El Salvador, we assumed 50% of local cancer patients presented with FIGO stage 1a, requiring radical hysterectomy, and 50% of local cancer patients presented with FIGO stage 1b/2a, requiring cobalt therapy followed by 5 chemotherapy sessions followed by brachytherapy. We assumed all women presenting with FIGO stage 2b or higher received cobalt therapy followed by 5 chemotherapy sessions followed by brachytherapy.

To adjust the direct medical costs of cancer treatment in El Salvador to the setting of Nicaragua, we multiplied by the ratio of WHO-CHOICE inpatient bed-day costs at a teaching hospital in Nicaragua relative to El Salvador. We assumed each patient and a support person spent the same amount of time traveling, waiting for, and receiving care as in El Salvador, and valued this time at the 2013 minimum wage rate in Nicaragua (inflated and converted to 2015 US$). To adjust temporary housing and transportation costs to the setting of Nicaragua, we multiplied the Salvadoran costs by the ratio of GDP per capita in Nicaragua relative to El Salvador. Costs are reported in **Table A.10**.

SUPPLEMENTARY RESULTS

Additional results described in the main manuscript are presented in **Figures A3** to **A6.**

For peer review only

Table A.1. Age-specific prevalence of high-risk HPV, Nicaragua [15].[a]

Age group	Number of women	Number of women with high-risk HPV	Prevalence (95% CI)
30 – 34 years	1,693	310	0.18 (0.17, 0.20)
35 – 39 years	1,141	184	0.16 (0.14, 0.18)
40 – 44 years	933	125	0.13 (0.11, 0.16)
45 – 49 years	878	121	0.14 (0.12, 0.16)

[a] HPV positivity was based on a cut-off of 0.5 relative light units.

Table A.2. Age-specific cervical cancer incidence, Nicaragua (GLOBOCAN 2012) [11].

Age group	Cases	Rate per 100,000 women (95% CI)
40 – 44 years	123	78.7 (64.8, 92.6)
45 – 49 years	112	85.4 (69.6, 101.2)
50 – 54 years	102	88.4 (71.2, 105.6)
55 – 59 years	85	88.1 (69.4, 106.8)
60 – 64 years	51	84.0 (61.0, 107.1)
65 – 69 years	37	80.8 (54.8, 106.8)
70 – 74 years	30	74.6 (47.9, 101.3)
≥75 years	45	70.3 (49.8, 90.8)

For peer review only

For peer review only - http://bmjopen.bmj.com/site/about/guidelines.xhtml

Table A.3. Monthly transition probabilities in the calibrated natural history model of HPV infection and cervical carcinogenesis.

HPV Infection

HPV Type and Age Group, years	Baseline Value		Range of Multiplier Values Among Top 50 Parameter Sets[a]	
	Min	Max	Min	Max
**HPV 16**				
<21	0	0.00186	3.15278	7.90861
21-24	0.000903	0.00125	0.670187	6.75291
25-29	0.000782	0.00087	0.604518	7.39154
30-49	0.000602	0.00078	0.677505	7.86645
>= 50	0.000217	0.00059	0.363714	8.9736
**HPV 18**				
<21	0	0.00116	0.365305	9.94514
21-24	0.0011	0.001173	0.304444	8.18583
25-29	0.0006	0.001	0.305103	8.23571
30-49	0.000301	0.00058	0.336658	9.81842
>= 50	0.000109	0.000295	0.347223	9.86684
**HPV 31**				
<21	0	0.001428	0.432486	7.54208
21-24	0.0009	0.001364	0.332414	5.86815
25-29	0.0006	0.000805	0.312983	6.63163
30-49	0.000301	0.00055	0.381602	7.86096
>= 50	0.000109	0.000295	0.445801	7.91394
**HPV 33**				
<21	0	0.000786	0.337263	5.98918
21-24	0.000412	0.00069	0.321392	5.21972
25-29	0.00025	0.00036	0.321163	4.52998
30-49	0.000131	0.00022	0.315971	5.91849
>= 50	0.000054	0.000128	0.341468	5.73381
**HPV 45**				
<21	0	0.001	0.838185	7.90205
21-24	0.00041	0.0008	0.303553	6.97879
25-29	0.000315	0.00037	0.373551	7.22544
30-49	0.000161	0.000305	0.320699	7.65339
>= 50	0.000054	0.00015	0.313646	7.97911

For peer review only - http://bmjopen.bmj.com/site/about/guidelines.xhtml

For peer review only

**HPV 52**				
<21	0	0.001186	0.339479	5.92479
21-24	0.00027	0.0008	0.328541	4.11892
25-29	0.000184	0.000254	0.301478	4.73227
30-49	0.00015	0.000173	0.365365	5.61621
>= 50	0.000054	0.000148	0.333456	11.406
**HPV 58**				
<21	0	0.001189	0.586648	7.78762
21-24	0.00059	0.0011	0.30793	7.02402
25-29	0.000465	0.00055	0.376855	6.55248
30-49	0.000244	0.00045	0.300592	7.20446
>= 50	0.000054	0.000239	0.42182	7.99349
**Other Oncogenic Types**				
<21	0	0.00247	0.324069	5.82435
21-24	0.001804	0.0023	0.301534	5.30746
25-29	0.0012	0.0017	0.311474	4.99753
30-49	0.000602	0.0011	0.318774	5.86776
>= 50	0.000217	0.00059	0.313403	5.86636
**Nononcogenic Types**				
<21	0	0.00261	0.361451	14.721
21-24	0.00255	0.00262	0.327572	10.6641
25-29	0.002	0.0025	0.327932	13.399
30-49	0.00085	0.00186	0.326549	14.4844
>= 50	0.000217	0.0008	0.324229	14.05

HPV clearance

HPV Type and time since infection, months	Baseline Value	Multiplier Value Among Top 50 Parameter Sets[a]
**HPV 16**		
1-15	0.041886	1
16-27	0.040754	1
28-39	0.033905	1
40-51	0.031888	1
52+	0.019846	1
**HPV 18**		
1-15	0.073342	1
16-27	0.063235	1
28-39	0.053605	1
40-51	0.020616	1

52+	0.020616	1
**HPV 31**		
1-15	0.063447	1
16-27	0.033826	1
28-39	0.033826	1
40-51	0.033826	1
52+	0.033826	1
**HPV 33**		
1-15	0.083452	1
16-27	0.044955	1
28-39	0.036156	1
40-51	0.036156	1
52+	0.036156	1
**HPV 45**		
1-15	0.078517	1
16-27	0.042579	1
28-39	0.041675	1
40-51	0.030133	1
52+	0.030133	1
**HPV 52**		
1-15	0.062999	1
16-27	0.044401	1
28-39	0.044401	1
40-51	0.039325	1
52+	0.039325	1
**HPV 58**		
1-15	0.065572	1
16-27	0.05443	1
28-39	0.053968	1
40-51	0.033319	1
52+	0.033319	1
**High Risk HPV**		
1-15	0.080766	1
16-27	0.066633	1
28-39	0.053972	1
40-51	0.049229	1
52+	0.005094	1

For peer review only

For peer review only - http://bmjopen.bmj.com/site/about/guidelines.xhtml

**Low Risk HPV**		
1-15	0.051888	1
16-27	0.050005	1
28-39	0.034649	1
40-51	0.034649	1
52-63	0.034649	1
64+	0.028608	1

HPV Progression to CIN2

**HPV Type and time since infection, months**	Baseline Value	Multiplier Value Among Top 50 Parameter Sets[a]
**HPV 16**		
1-15	0.001707	1
16-27	0.002422	1
28-39	0.002577	1
40-51	0.005518	1
52+	0.014998	1
**HPV 18**		
1-15	4.27E-05	1
16-27	0.000189	1
28-39	0.000189	1
40-51	0.007733	1
52+	0.007733	1
**HPV 31**		
1-15	0.000262	1
16-27	0.00278	1
28-39	0.003091	1
40-51	0.006931	1
52+	0.006931	1
**HPV 33**		
1-15	0.000719	1
16-27	0.000719	1
28-39	0.004939	1
40-51	0.004939	1
52+	0.004939	1
**HPV 45**		
1-15	0	1
16-27	0	1

For peer review only

For peer review only - http://bmjopen.bmj.com/site/about/guidelines.xhtml

28-39	0.002258	1
40-51	0.005333	1
52+	0.005333	1
**HPV 52**		
1-15	0.000884	1
16-27	0.001681	1
28-39	0.001681	1
40-51	0.001978	1
52+	0.005678	1
**HPV 58**		
1-15	0.000587	1
16-27	0.002468	1
28-39	0.002468	1
40-51	0.004617	1
52+	0.010246	1
**High Risk HPV**		
1-15	0.000126	1
16-27	0.000373	1
28-39	0.00196	1
40-51	0.00196	1
52+	0.00196	1
**Low Risk HPV**		
1-15	0.000205	1
16-27	0.000291	1
28-39	0.00031	1
40-51	0.000663	1
52+	0.000663	1

HPV progression to CIN3

HPV Type and time since infection, months	Baseline Value	Multiplier Value Among Top 50 Parameter Sets[a]
**HPV 16**		
1-15	0.000569	1
16-27	0.000808	1
28-39	0.00086	1
40-51	0.001843	1
52+	0.005024	1
**HPV 18**		

For peer review only

1-15	1.07E-05	1
16-27	4.72E-05	1
28-39	4.72E-05	1
40-51	0.001939	1
52+	0.001939	1
**HPV 31**		
1-15	6.55E-05	1
16-27	0.000696	1
28-39	0.000774	1
40-51	0.001737	1
52+	0.001737	1
**HPV 33**		
1-15	0.00018	1
16-27	0.00018	1
28-39	0.001237	1
40-51	0.001237	1
52+	0.001237	1
**HPV 45**		
1-15	0	1
16-27	0	1
28-39	0.000565	1
40-51	0.001336	1
52+	0.001336	1
**HPV 52**		
1-15	0.000221	1
16-27	0.000421	1
28-39	0.000421	1
40-51	0.000495	1
52+	0.001423	1
**HPV 58**		
1-15	0.000147	1
16-27	0.000618	1
28-39	0.000618	1
40-51	0.001156	1
52+	0.002571	1
**High Risk HPV**		
1-15	3.16E-05	1

For peer review only

For peer review only - http://bmjopen.bmj.com/site/about/guidelines.xhtml

16-27	9.32E-05	1
28-39	0.00049	1
40-51	0.00049	1
52+	0.00049	1
**Low Risk HPV**		
1-15	2.28E-05	1
16-27	3.23E-05	1
28-39	3.44E-05	1
40-51	7.37E-05	1
52+	7.37E-05	1

Regression of CIN2 to NL

**HPV Type and time since infection, years**	Baseline Value		Range of Multiplier Values Among Top 50 Parameter Sets[a]	
	Min	Max	Min	Max
**HPV 16**				
1-5	0.05	0.523578	0.500863	0.687389
6-10	0.035	0.523578	0.500863	0.687389
11-20	0.013	0.523578	0.500863	0.687389
21-29	0.0005	0.523578	0.500863	0.687389
30-39	0.0001	0.523578	0.500863	0.687389
40+	0.00005	0.523578	0.500863	0.687389
**All Other HPV Types**				
1-5	0.05	0.523578	0.527319	3.70898
6-10	0.035	0.523578	0.527319	3.70898
11-20	0.013	0.523578	0.527319	3.70898
21-29	0.0005	0.523578	0.527319	3.70898
30-39	0.0001	0.523578	0.527319	3.70898
40+	0.00005	0.523578	0.527319	3.70898

Regression of CIN3 to NL

**HPV Type and time since infection, years**	Baseline Value		Range of Multiplier Values Among Top 50 Parameter Sets[a]	
	Min	Max	Min	Max
**HPV 16**				
1-5	0.025	0.639221	0.50664	1.67845
6-10	0.0175	0.639221	0.50664	1.67845
11-20	0.0065	0.639221	0.50664	1.67845

For peer review only

For peer review only - http://bmjopen.bmj.com/site/about/guidelines.xhtml

21-29	0.00025	0.639221	0.50664	1.67845
30-39	0.00005	0.639221	0.50664	1.67845
40+	0.000025	0.639221	0.50664	1.67845
**All Other HPV Types**				
1-5	0.025	0.639221	0.577736	3.9781
6-10	0.0175	0.639221	0.577736	3.9781
11-20	0.0065	0.639221	0.577736	3.9781
21-29	0.00025	0.639221	0.577736	3.9781
30-39	0.00005	0.639221	0.577736	3.9781
40+	0.000025	0.639221	0.577736	3.9781

Progression of CIN2 to CA

**HPV Type and time since infection, years**	Baseline Value	Range of Multiplier Values Among Top 50 Parameter Sets[a]	
		Min	Max
**HPV16**			
1-5	3.29E-05	1.00901	1.49572
6-10	3.56E-05	1.00901	1.49572
11-20	0.000857	1.00901	1.49572
21-29	0.002506	1.00901	1.49572
30-34	0.005011	1.00901	1.49572
35-39	0.005429	1.00901	1.49572
40-44	0.011693	1.00901	1.49572
45-49	0.012528	1.00901	1.49572
50+	0.33408	1.00901	1.49572
**HPV 18**			
1-5	3.29E-05	1.00901	1.49572
6-10	3.56E-05	1.00901	1.49572
11-20	0.000571	1.00901	1.49572
21-29	0.002506	1.00901	1.49572
30-34	0.005011	1.00901	1.49572
35-39	0.005429	1.00901	1.49572
40-44	0.011693	1.00901	1.49572
45-49	0.012528	1.00901	1.49572
50+	0.33408	1.00901	1.49572
**HPV 31**			
1-5	2.2E-05	1.00901	1.49572
6-10	2.38E-05	1.00901	1.49572
11-20	0.000857	1.00901	1.49572

For peer review only

21-29	0.00167	1.00901	1.49572
30-34	0.003341	1.00901	1.49572
35-39	0.003619	1.00901	1.49572
40-44	0.007795	1.00901	1.49572
45-49	0.008352	1.00901	1.49572
50+	0.22272	1.00901	1.49572
**HPV 33**			
1-5	3.29E-05	1.00901	1.49572
6-10	3.56E-05	1.00901	1.49572
11-20	0.000571	1.00901	1.49572
21-29	0.002506	1.00901	1.49572
30-34	0.005011	1.00901	1.49572
35-39	0.005429	1.00901	1.49572
40-44	0.011693	1.00901	1.49572
45-49	0.012528	1.00901	1.49572
50+	0.33408	1.00901	1.49572
**HPV 45**			
1-5	2.2E-05	1.00901	1.49572
6-10	2.38E-05	1.00901	1.49572
11-20	0.000571	1.00901	1.49572
21-29	0.00167	1.00901	1.49572
30-34	0.003341	1.00901	1.49572
35-39	0.003619	1.00901	1.49572
40-44	0.007795	1.00901	1.49572
45-49	0.008352	1.00901	1.49572
50+	0.22272	1.00901	1.49572
**HPV 52**			
1-5	2.2E-05	1.00901	1.49572
6-10	2.38E-05	1.00901	1.49572
11-20	0.000571	1.00901	1.49572
21-29	0.00167	1.00901	1.49572
30-34	0.003341	1.00901	1.49572
35-39	0.003619	1.00901	1.49572
40-44	0.007795	1.00901	1.49572
45-49	0.008352	1.00901	1.49572
50+	0.22272	1.00901	1.49572
**HPV 58**			
1-5	2.2E-05	1.00901	1.49572
6-10	2.38E-05	1.00901	1.49572

For peer review only

11-20	0	1.00901	1.49572
21-29	0.00167	1.00901	1.49572
30-34	0.003341	1.00901	1.49572
35-39	0.003619	1.00901	1.49572
40-44	0.007795	1.00901	1.49572
45-49	0.008352	1.00901	1.49572
50+	0.22272	1.00901	1.49572
**High Risk HPV**			
1-5	2.2E-05	1.00901	1.49572
6-10	2.38E-05	1.00901	1.49572
11-20	0.000571	1.00901	1.49572
21-29	0.00167	1.00901	1.49572
30-34	0.003341	1.00901	1.49572
35-39	0.003619	1.00901	1.49572
40-44	0.007795	1.00901	1.49572
45-49	0.008352	1.00901	1.49572
50+	0.22272	1.00901	1.49572

Progression of CIN3 to CA

HPV Type and time since infection, years	Baseline Value	Range of Multiplier Values Among Top 50 Parameter Sets[a]	
		Min	Max
**HPV 16**			
1-5	0.000165	1.00199	1.48007
6-10	0.000178	1.00199	1.48007
11-20	0.004284	1.00199	1.48007
21-29	0.012528	1.00199	1.48007
30-34	0.025056	1.00199	1.48007
35-39	0.027144	1.00199	1.48007
40-44	0.058464	1.00199	1.48007
45-49	0.06264	1.00199	1.48007
50+	0.33408	1.00199	1.48007
**HPV 18**			
1-5	0.000165	1.00199	1.48007
6-10	0.000178	1.00199	1.48007
11-20	0.004284	1.00199	1.48007
21-29	0.012528	1.00199	1.48007
30-34	0.025056	1.00199	1.48007
35-39	0.027144	1.00199	1.48007
40-44	0.058464	1.00199	1.48007
45-49	0.06264	1.00199	1.48007

For peer review only

For peer review only - http://bmjopen.bmj.com/site/about/guidelines.xhtml

50+	0.33408	1.00199	1.48007
**HPV 31**			
1-5	0.00011	1.00199	1.48007
6-10	0.000119	1.00199	1.48007
11-20	0.002856	1.00199	1.48007
21-29	0.008352	1.00199	1.48007
30-34	0.016704	1.00199	1.48007
35-39	0.018096	1.00199	1.48007
40-44	0.038976	1.00199	1.48007
45-49	0.04176	1.00199	1.48007
50+	0.22272	1.00199	1.48007
**HPV 33**			
1-5	0.000165	1.00199	1.48007
6-10	0.000178	1.00199	1.48007
11-20	0.004284	1.00199	1.48007
21-29	0.012528	1.00199	1.48007
30-34	0.025056	1.00199	1.48007
35-39	0.027144	1.00199	1.48007
40-44	0.058464	1.00199	1.48007
45-49	0.06264	1.00199	1.48007
50+	0.33408	1.00199	1.48007
**HPV 45**			
1-5	0.00011	1.00199	1.48007
6-10	0.000119	1.00199	1.48007
11-20	0.002856	1.00199	1.48007
21-29	0.008352	1.00199	1.48007
30-34	0.016704	1.00199	1.48007
35-39	0.018096	1.00199	1.48007
40-44	0.038976	1.00199	1.48007
45-49	0.04176	1.00199	1.48007
50+	0.22272	1.00199	1.48007
**HPV 52**			
1-5	0.00011	1.00199	1.48007
6-10	0.000119	1.00199	1.48007
11-20	0.002856	1.00199	1.48007
21-29	0.008352	1.00199	1.48007
30-34	0.016704	1.00199	1.48007
35-39	0.018096	1.00199	1.48007
40-44	0.038976	1.00199	1.48007

For peer review only - http://bmjopen.bmj.com/site/about/guidelines.xhtml

45-49	0.04176	1.00199	1.48007
50+	0.22272	1.00199	1.48007
**HPV 58**			
1-5	0.00011	1.00199	1.48007
6-10	0.000119	1.00199	1.48007
11-20	0.002856	1.00199	1.48007
21-29	0.008352	1.00199	1.48007
30-34	0.016704	1.00199	1.48007
35-39	0.018096	1.00199	1.48007
40-44	0.038976	1.00199	1.48007
45-49	0.04176	1.00199	1.48007
50+	0.22272	1.00199	1.48007
**High Risk HPV**			
1-5	0.00011	1.00199	1.48007
6-10	0.000119	1.00199	1.48007
11-20	0.002856	1.00199	1.48007
21-29	0.008352	1.00199	1.48007
30-34	0.016704	1.00199	1.48007
35-39	0.018096	1.00199	1.48007
40-44	0.038976	1.00199	1.48007
45-49	0.04176	1.00199	1.48007
50+	0.22272	1.00199	1.48007

Progression of invasive cancer stages

	Baseline Value
Local to regional	0.02
Regional to distant	0.025

Invasive cancer mortality

Local	0.005876
Regional	0.015137
Distant	0.065519

Probability of symptom detection

Local	0.0039
Regional	0.1333
Distant	0.1746

3

For peer review only - http://bmjopen.bmj.com/site/about/guidelines.xhtml

[a] Multiplier values were drawn from the identified plausible search ranges and applied to baseline input probabilities; the values presented represent the multiplier values that yielded the 50 best fits to the epidemiologic calibration targets.

For peer review only

For peer review only - http://bmjopen.bmj.com/site/about/guidelines.xhtml

Table A.4. Screening: Direct medical costs per procedure (2015 US$).[a]

Cost category	HPV test (provider-collection)[b]	HPV test (self-collection)[b]	Cytology	VIA triage	Source
Clinical staff	3.81	2.88	3.81	3.81	START-UP
Clinical supplies	6.72	6.72	0.82	0.36	Scale-Up (HPV);[c] START-UP (Cytology/VIA)
Clinical equipment	0.01	0.01	0.01	0.02	
Lab staff	0.41	0.41	1.37	0.00	Scale-Up (transport personnel);[d] START-UP (other)
Lab supplies	0.81	0.81	0.92	0.00	Scale-Up (transport fuel);[e] START-UP (other)
Lab equipment	0.21	0.21	0.32	0.00	START-UP
**Total direct medical cost**	**11.96**	**11.04**	**7.26**	**4.19**	

[a] HPV: human papillomavirus; Scale-Up: Scale-Up demonstration project (2015); START-UP: Screening Technologies to Advance Rapid Testing for Cervical Cancer Prevention–Utility and Program Planning demonstration project (2010); US$: United States dollars; VIA: visual inspection with acetic acid.

[b] HPV testing with both provider-collection of cervical samples and self-collection of vaginal samples was assumed to take place at the clinic. The difference in costs between collection modalities is due to reduced clinical staff time when a woman self-collects her own sample.

[c] The cost of clinical supplies for HPV testing was drawn from the Scale-Up project and includes the test kit, plate sealers, brush, collection medium, pipette tips (3 types), gloves, and towels to decant and dry.

[d] The cost of laboratory staff time was drawn from the START-UP project, with the exception of preparing and transporting samples to the laboratory, which was drawn from the Scale-Up project. Staff time spent preparing samples for transport in the Scale-Up project was based on project staff estimates in each of the three departments (Carazo, Chontales, and Chinandega), which ranged from 5 to 7 minutes per sample. Staff time for round-trip transport of samples was averaged across 9 to 10 facilities in each of the three departments, and valued based on the average monthly wage of the personnel responsible for transporting the samples in each department. We derived an average cost per sample prepared and transported for each department (assuming the cost of staff time per sample was equivalent for HPV and cytology specimens), and then averaged these costs across the three departments.

[e] For HPV testing, all lab supplies are bundled in the category of clinical supplies, with the exception of fuel for laboratory transport, which is included under lab supplies. For both HPV and cytology testing, we assumed the same cost of fuel per sample. Using Scale-Up project staff estimates of the number of samples transported per load, the distance to the lab from 9 to 10 facilities in each department, and the usual type of vehicle used in each department, we derived the average fuel cost per sample using the average fuel economy of major vehicle types [16] and the average gas price per liter in Nicaragua [12].

For peer review only - http://bmjopen.bmj.com/site/about/guidelines.xhtml

Table A.5. Diagnosis and Treatment of Precancer: Direct medical costs per procedure (2015 US$).[a]

Cost category	Colposcopy	Colposcopy and biopsy	Cryotherapy	LEEP	Source
Clinical staff	5.92	6.29	7.54	7.34	START-UP
Clinical supplies	0.38	1.39	3.54	36.05	Scale-Up (cryotherapy gas);[b] START-UP (other)
Drugs	0.13	0.13	0.30	0.10	START-UP
Clinical equipment	0.49	0.53	9.13	13.29	Scale-Up (cryotherapy unit);[c] START-UP (other)
Lab staff	0.00	4.09	0.00	4.09	START-UP
Lab supplies	0.00	3.35	0.00	3.35	START-UP
Lab equipment	0.00	4.12	0.00	4.12	START-UP
**Total direct medical cost**	**6.91**	**19.91**	**18.16**	**68.36**	

[a] LEEP: loop electrosurgical excision procedure; Scale-Up: Scale-Up demonstration project (2015); START-UP: Screening Technologies to Advance Rapid Testing for Cervical Cancer Prevention–Utility and Program Planning demonstration project (2010); US$: United States dollars.

[b] We used estimates from Scale-Up project staff to inform the cost of gas used per cryotherapy procedure, based on the cost of 1 cylinder and the number of women treated per tank. We did not include the cost of the tank deposit.

[c] To estimate the cost per procedure of a cryotherapy unit, we assumed this equipment item was a tradable good, and accordingly converted the purchase price from local currency units to US$ using the official exchange rate. We annualized the cost with a 3% interest rate and assumed an economic life of 5 years. To derive the number of procedures per machine per year across the three departments considered, we divided the annual number of cryotherapy procedures by the number of cryotherapy units. We then divided the annualized cryotherapy unit cost by the number of procedures per machine per year.

For peer review only

Table A.6. Location of service delivery for screening, diagnosis, and treatment of precancerous lesions and cancer.[a]

Procedure	Location of services
HPV DNA test	Screening facility
Cytology test	Screening facility
VIA test	Referral facility
Colposcopy/biopsy	Referral facility
Cryotherapy	Referral facility
LEEP	Referral facility
Follow-up visits (after cryotherapy or LEEP)	Primary facility
Cancer treatment	Tertiary facility

[a] HPV: human papillomavirus; LEEP: loop electrosurgical excision procedure; VIA: visual inspection with acetic acid.

For peer review only

Tabe A.7. Women's time estimates.[a]

Visit	Minutes	Cost (2015 US$)[b]	Source
**Screening**[c]			
Wait time	33	0.48	Scale-Up
Procedure time	20	0.29	START-UP
Transport time (round-trip)	57	0.82	Scale-Up
**Results**[c]			
Wait time	33	0.48	Scale-Up
Procedure time	2	0.03	START-UP
Transport time (round-trip)	57	0.82	Scale-Up
**Cryotherapy**[c]			
Wait time	120	1.75	Scale-Up
Procedure time	35	0.51	START-UP
Transport time (round-trip)	213	3.10	Scale-Up
**Colposcopy/biopsy**			
Wait time	120	1.75	Scale-Up
Procedure time	37	0.54	START-UP
Transport time (round-trip)	213	3.10	Scale-Up
**LEEP**			
Wait time	120	1.75	Scale-Up
Procedure time	25	0.36	START-UP
Transport time (round-trip)	213	3.10	Scale-Up

[a] LEEP: loop electrosurgical excision procedure; Scale-Up: Scale-Up demonstration project (2015); START-UP: Screening Technologies to Advance Rapid Testing for Cervical Cancer Prevention–Utility and Program Planning demonstration project (2010); US$: United States dollars.

[b] Women's time was valued using Nicaragua's 2013 monthly minimum wage [13], converted to 2015 US$. We converted this to an hourly wage rate assuming 171 hours of work per month (40 hours per week).

[c] Wait and transport time costs were accrued for each visit to a health facility, but we did not double-count wait and transport times when multiple procedures were performed in the same visit (e.g., VIA triage of screen-positive women and cryotherapy of VIA+ women accrued the wait time and transport time costs associated with a single visit to a referral facility, but accrued the time costs of both procedures.)

For peer review only

Table A.8. Women's transportation costs.[a]

**Facility**[b]	**Round-trip transportation cost (2015 US$)**	**Source**
Screening facility	0.41	Scale-Up
Referral facility	2.81	Scale-Up

[a] Scale-Up: Scale-Up demonstration project (2015); US$: United States dollar.

[b] Screening with cytology or HPV testing was assumed to take place at a screening facility (i.e., a primary level health facility). Triage testing with visual inspection with acetic acid (VIA), cryotherapy, colposcopy/biopsy, and loop electrosurgical excision procedure (LEEP) were assumed to take place at referral facilities.

For peer review only

Table A.9. Programmatic training costs for HPV-based screening.[a]

Type of training	Total cost of supplies/materials[b] (2015 US$)	Total cost of personnel time[c] (2015 US$)	Per woman cost[d] (2015 US$)
Health care personnel offering careHPV	2,758	5,784	0.09
Laboratory technicians processing careHPV assays	3,503	526	0.04
Community outreach workers promoting and offering self-collection	2,662	3,618	0.08
Health care providers offering VIA and cryotherapy	8,103	16,069	1.51

[a] HPV: human papillomavirus; US$: United States dollars; VIA: visual inspection with acetic acid.

[b] The total cost of supplies, materials, transportation, and refreshments for all training sessions of each type was converted from 2015 local currency units using the official exchange rate.

[c] The total cost of personnel time includes time costs for both the trainer and the trainees, valued using average monthly salary rates of each type of personnel present (and the proportion of trainees of each personnel type) and based on the length of the training session.

[d] To derive an average health care personnel training cost per woman screened, we divided the total training cost for health care personnel by 100,000, corresponding to the number of women screened during the Scale-Up project. To derive an average laboratory technician training cost per woman screened, we divided the total training cost for laboratory technicians by 100,000. To derive an average community outreach worker training cost per woman screened with self-collection, we divided the total training cost for outreach workers by 80,000, corresponding to the number of women who participated in self-collection of HPV samples (due to lack of data on self-collection in a community setting, in the base case analysis we assumed self-collection took place at the clinic; however, we assumed outreach workers would be involved in mobilizing and educating women about self-collection, and conservatively included the cost of training so as to represent a known programmatic cost as self-collection efforts shift to also include community settings). To derive an average health care provider training cost per woman receiving VIA or cryotherapy, we divided the total VIA/cryotherapy training cost for health care providers by 16,000, corresponding to the approximate number of women who tested HPV-positive in Scale-Up and would thus require visual assessment and cryotherapy.

For peer review only

Table A.10. Cancer treatment costs (2015 US$).

Cancer stage	Local cancer	Regional cancer	Distant cancer
Direct medical costs[a]	943.62	917.83	917.83
Woman and support person time	345.77	639.14	639.14
Transportation, temporary housing, and meals	196.78	389.50	389.50
**Total cancer treatment cost**	**1486**	**1946**	**1946**

[a] Direct medical costs are based on the premise that 50% of local cancer patients presented with FIGO stage 1a, requiring radical hysterectomy, and 50% of local cancer patients presented with FIGO stage 1b/2a, requiring cobalt therapy followed by 5 chemotherapy sessions followed by brachytherapy. We assumed all women presenting with FIGO stage 2b or higher received cobalt therapy followed by 5 chemotherapy sessions followed by brachytherapy.

[b] Patient and support person time costs value time using the minimum wage in Nicaragua, as described in the text.

[c] Direct non-medical costs include round-trip transportation for visits to a cancer center and meals and temporary housing during the course of treatment.

For peer review only

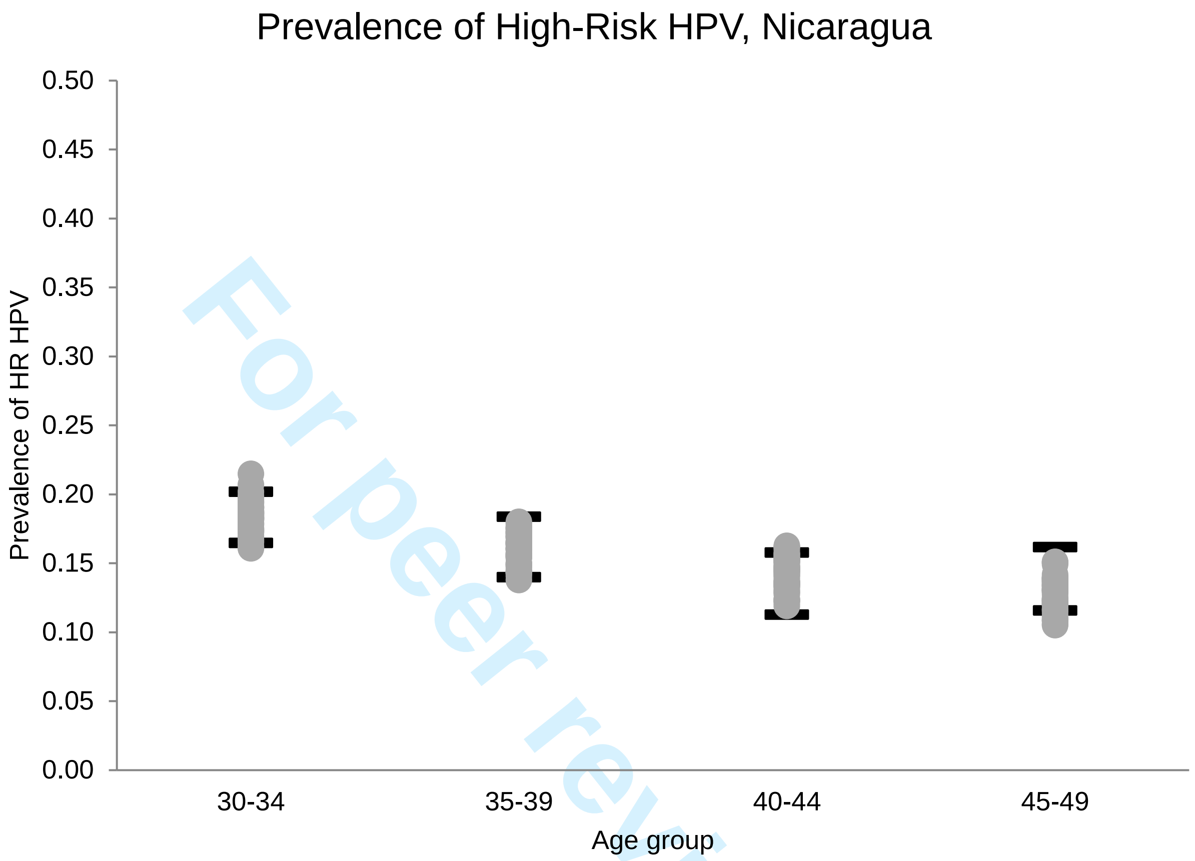

Figure A1. Selected model output from the top 50 input parameter sets compared with empirical data (i.e., calibration targets) on age-specific prevalence of high-risk HPV in Nicaragua, based on a relative light unit cut-off value of 0.5 in the START-UP studies [2, 15]. Bold lines represent the 95% confidence intervals around the empirical data, and gray circles represent model output from each of the top 50 input parameter sets.

For peer review only - http://bmjopen.bmj.com/site/about/guidelines.xhtml

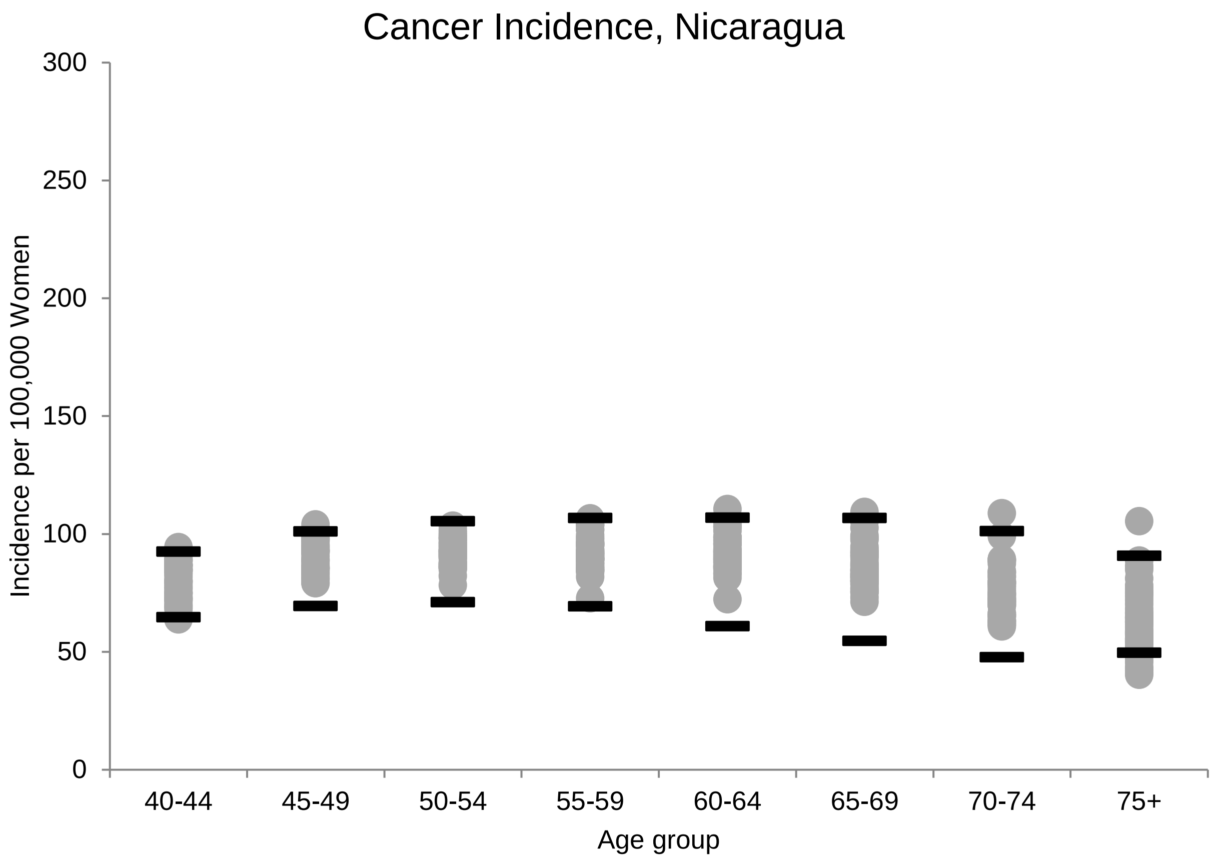

Figure A2. Selected model output from the top 50 input parameter sets compared with empirical data (i.e., calibration targets) on age-specific cancer incidence in Nicaragua [2, 11]. Bold lines represent the 95% confidence intervals around the empirical data, and gray circles represent model output from each of the top 50 input parameter sets.

For peer review only - http://bmjopen.bmj.com/site/about/guidelines.xhtml

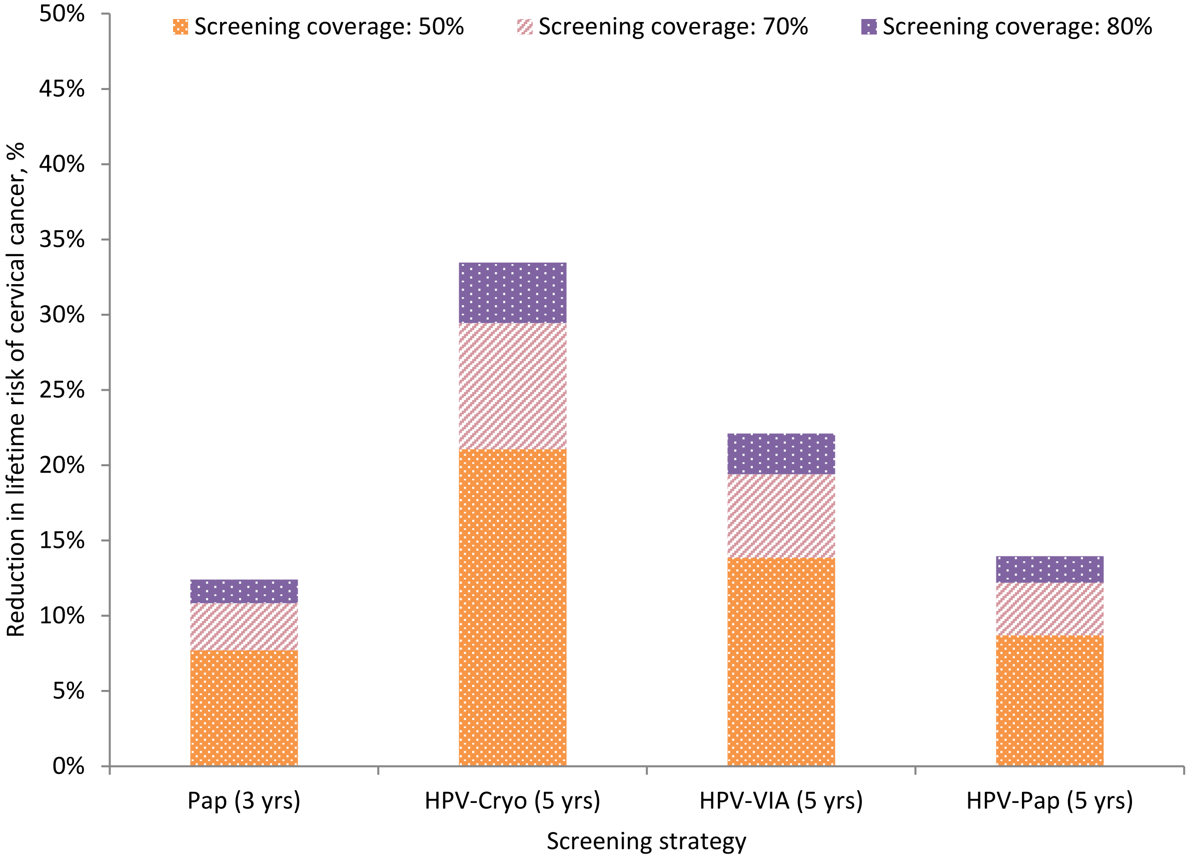

Figure A3. Reduction in lifetime risk of cervical cancer, by screening coverage level. Bars indicate the percent reduction in lifetime risk of cervical cancer for each screening strategy (Pap testing every 3 years; careHPV with cryotherapy for HPV-positive women every 5 years [HPV-Cryo]; careHPV every 5 years with visual inspection with acetic acid [VIA] triage of HPV-positive women [HPV-VIA]; and careHPV every 5 years with Pap triage of HPV-positive women [HPV-Pap]) as screening coverage of the target population increases.

For peer review only - http://bmjopen.bmj.com/site/about/guidelines.xhtml

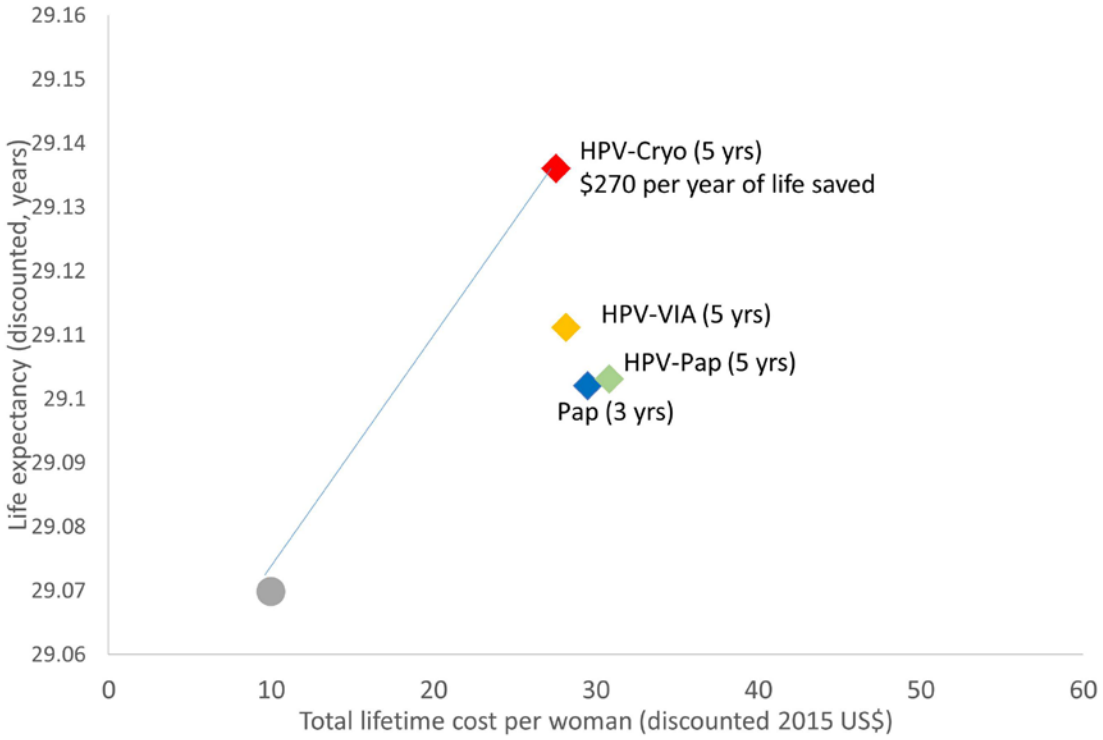

Figure A4. Cost-effectiveness analysis: Payer cost only. The graph displays the discounted lifetime costs (x-axis; in 2015 US$) and life expectancy (y-axis) associated with each screening strategy (Pap testing every 3 years; careHPV with cryotherapy for HPV-positive women every 5 years [HPV-Cryo]; careHPV every 5 years with visual inspection with acetic acid [VIA] triage of HPV-positive women [HPV-VIA]; and careHPV every 5 years with Pap triage of HPV-positive women [HPV-Pap]), when only costs to the payer are included (i.e., women's time and transportation costs are excluded). The cost-effectiveness associated with a change from one strategy to a more costly alternative is represented by the difference in cost divided by the difference in life expectancy associated with the two strategies. The curve indicates the strategies that are efficient because they are more effective and either 1) cost less; or 2) have a more attractive cost-effectiveness ratio than less effective options. The incremental cost-effectiveness ratio (ICER) is the reciprocal of the slope of the line connecting the two strategies under comparison. In this sensitivity analysis focused on costs to the payer, HPV-Cryo every 5 years was less costly and more effective than other screening strategies considered, and was thus a dominant strategy with an ICER of US$270 per year of life saved.

For peer review only - http://bmjopen.bmj.com/site/about/guidelines.xhtml

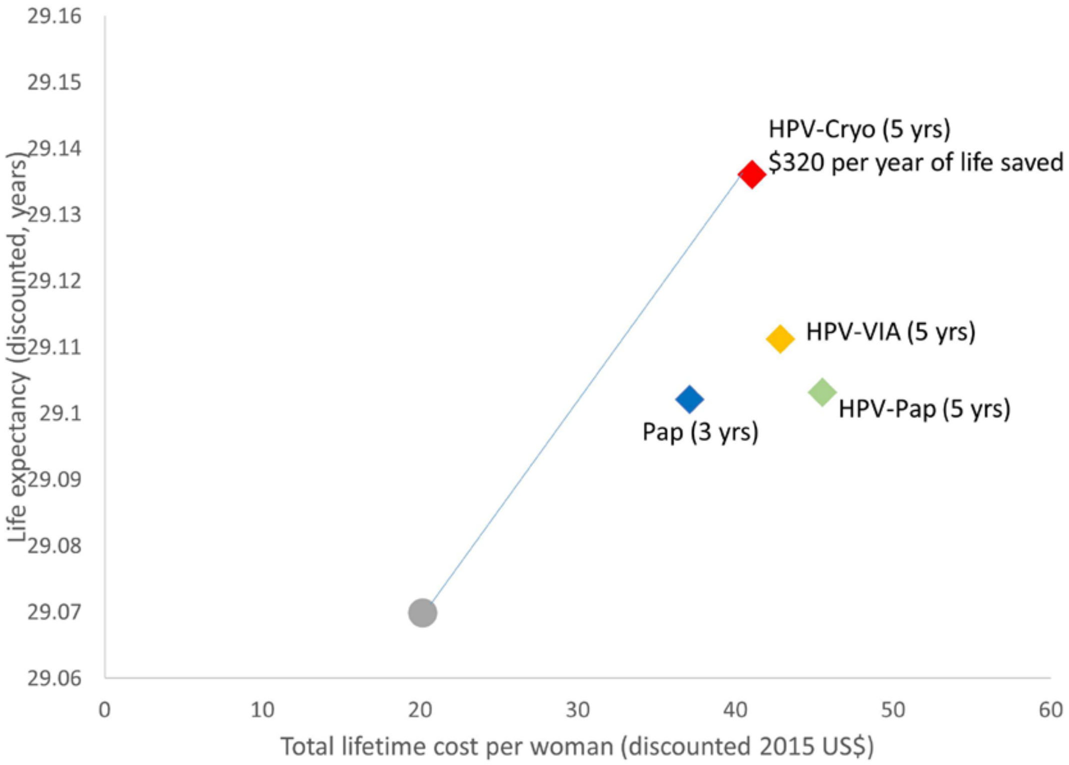

Figure A5. Cost-effectiveness analysis: Direct medical cost of Pap testing, US$3. The graph displays the discounted lifetime costs (x-axis; in 2015 US$) and life expectancy (y-axis) associated with each screening strategy (Pap testing every 3 years; careHPV with cryotherapy for HPV-positive women every 5 years [HPV-Cryo]; careHPV every 5 years with visual inspection with acetic acid [VIA] triage of HPV-positive women [HPV-VIA]; and careHPV every 5 years with Pap triage of HPV-positive women [HPV-Pap]), when the direct medical cost of Pap testing was assumed to be as low as US$3 (base case: US$7.26). The cost-effectiveness associated with a change from one strategy to a more costly alternative is represented by the difference in cost divided by the difference in life expectancy associated with the two strategies. The curve indicates the strategies that are efficient because they are more effective and either 1) cost less; or 2) have a more attractive cost-effectiveness ratio than less effective options. The incremental cost-effectiveness ratio (ICER) is the reciprocal of the slope of the line connecting the two strategies under comparison. In this sensitivity analysis examining the impact of a lower Pap test cost, HPV-Cryo every 5 years was more effective and had a more attractive cost-effectiveness ratio than Pap testing every 3 years; furthermore, HPV-Cryo was more effective and less costly than HPV-VIA and HPV-Pap, and was thus a dominant strategy with an ICER of US$320 per year of life saved.

For peer review only - http://bmjopen.bmj.com/site/about/guidelines.xhtml

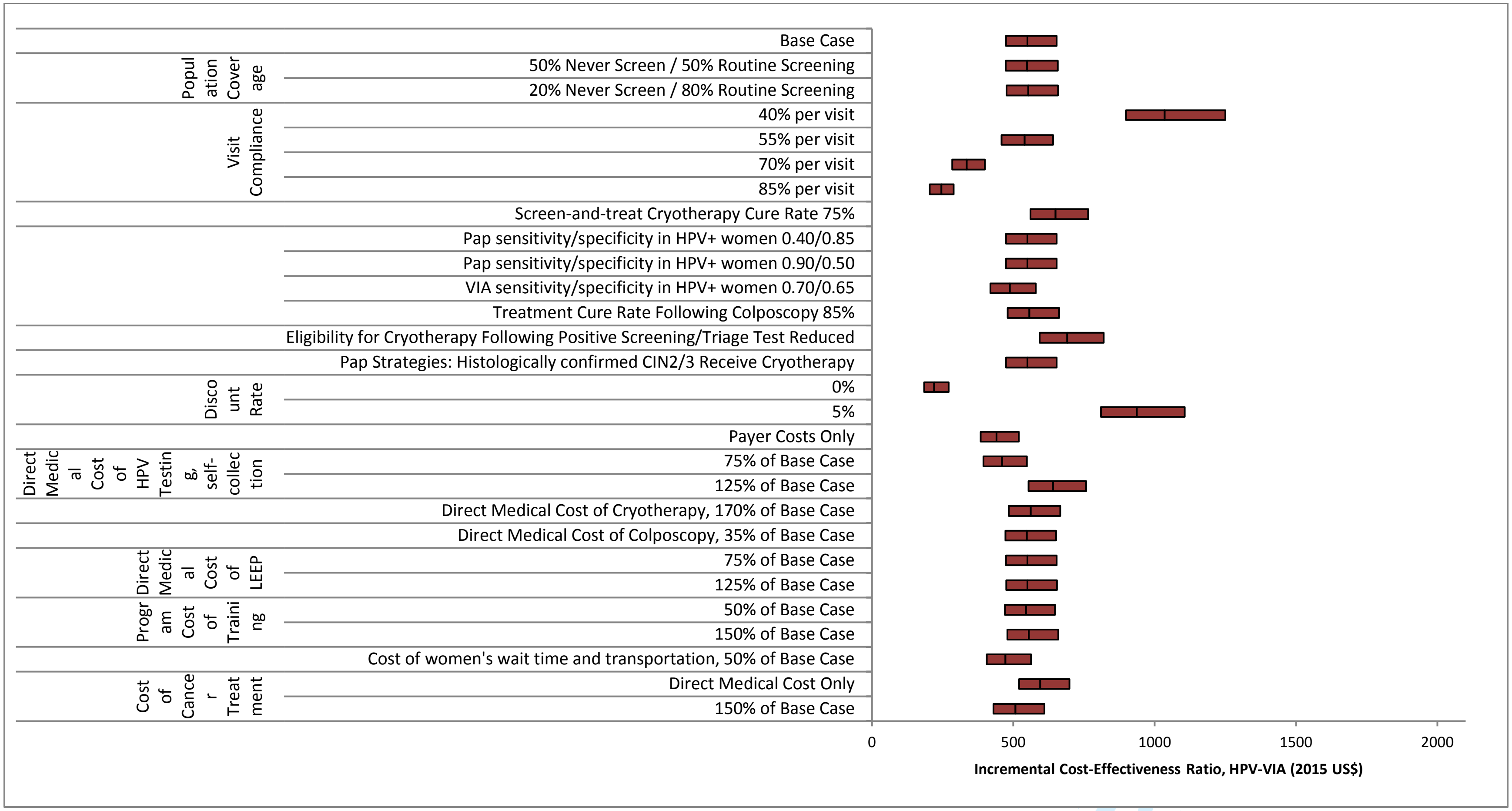

Figure A6. Base case and sensitivity analysis when HPV-Cryo is assumed to be unavailable: Incremental cost-effectiveness ratios, HPV-VIA strategy. Incremental cost-effectiveness ratios (ICERs) are presented (x-axis, 2015 US$ per year of life saved) for the base case and sensitivity analyses (y-axis), assuming HPV-Cryo is not available but Pap alone, HPV-VIA, and HPV-Pap are available. The orange bars represent the range of the ICERs for HPV-VIA every 5 years across the 50 input parameter sets, with the ICER of the mean costs divided by the mean effects demarcated by a black line. Nicaragua’s per capita gross domestic product (GDP) is US$2,090, as a possible threshold that designates interventions as “very cost-effective”.

For peer review only - http://bmjopen.bmj.com/site/about/guidelines.xhtml

References

1. Campos, N.G., et al., *An updated natural history model of cervical cancer: derivation of model parameters.* Am J Epidemiol, 2014. **180**(5): p. 545-55.
2. Campos, N.G., Tsu, V., Jeronimo, J., Mvundura, M., Lee, K., Kim, J.J., *When and how often to screen for cervical cancer in three low- and middle-income countries: A cost-effectiveness analysis.* Papillomavirus Research, 2015.
3. Munoz, N., et al., *Incidence, duration, and determinants of cervical human papillomavirus infection in a cohort of Colombian women with normal cytological results.* J Infect Dis, 2004. **190**(12): p. 2077-87.
4. McCredie, M.R., et al., *Natural history of cervical neoplasia and risk of invasive cancer in women with cervical intraepithelial neoplasia 3: a retrospective cohort study.* Lancet Oncol, 2008. **9**(5): p. 425-34.
5. Meyskens, F.L., Jr., et al., *Enhancement of regression of cervical intraepithelial neoplasia II (moderate dysplasia) with topically applied all-trans-retinoic acid: a randomized trial.* J Natl Cancer Inst, 1994. **86**(7): p. 539-43.
6. Keefe, K.A., et al., *A randomized, double blind, Phase III trial using oral beta-carotene supplementation for women with high-grade cervical intraepithelial neoplasia.* Cancer Epidemiol Biomarkers Prev, 2001. **10**(10): p. 1029-35.
7. Castle, P.E., et al., *Evidence for frequent regression of cervical intraepithelial neoplasia-grade 2.* Obstet Gynecol, 2009. **113**(1): p. 18-25.
8. Wang, S.M., et al., *Six-year regression and progression of cervical lesions of different human papillomavirus viral loads in varied histological diagnoses.* Int J Gynecol Cancer, 2013. **23**(4): p. 716-23.
9. Moscicki, A.B., et al., *Rate of and risks for regression of cervical intraepithelial neoplasia 2 in adolescents and young women.* Obstet Gynecol, 2010. **116**(6): p. 1373-80.
10. Herrero, R., et al., *Rationale and design of a community-based double-blind randomized clinical trial of an HPV 16 and 18 vaccine in Guanacaste, Costa Rica.* Vaccine, 2008. **26**(37): p. 4795-808.
11. Ferlay, J., et al., *GLOBOCAN 2012 v1.0, Cancer Incidence and Mortality Worldwide: IARC CancerBase No. 11 [Internet]*, 2013, International Agency for Research on Cancer: Lyon, France.
12. *World Development Indicators*, 2016, World Bank.
13. International Labour Organization. *Country Profiles: Nicaragua*, 2016.
14. Campos, N.G., et al., *The comparative and cost-effectiveness of HPV-based cervical cancer screening algorithms in El Salvador.* Int J Cancer, 2015.
15. Jeronimo, J., et al., *A multicountry evaluation of careHPV testing, visual inspection with acetic acid, and papanicolaou testing for the detection of cervical cancer.* Int J Gynecol Cancer, 2014. **24**(3): p. 576-85.
16. United States Department of Energy. *Average Fuel Economy of Major Vehicle Categories*. August 1, 2016]; Available from: http://www.afdc.energy.gov/data/10310.

For peer review only - http://bmjopen.bmj.com/site/about/guidelines.xhtml

CHEERS Checklist

Section/topic	#	Recommendation	Reported on page #/ line #
**TITLE AND ABSTRACT**			
Title	1	Identify the study as an economic evaluation or use more specific terms such as "cost-effectiveness analysis", and describe the interventions compared.	1/1
Abstract	2	Provide a structured summary of objectives, perspective, setting, methods (including study design and inputs), results (including base case and uncertainty analyses), and conclusions.	2/18-42
Abstract			
**INTRODUCTION**			
Background and objectives	3	Provide an explicit statement of the broader context for the study. Present the study question and its relevance for health policy or practice decisions.	7/112
**METHODS**			
Target population and subgroups	4	Describe characteristics of the base case population and subgroups analysed, including why they were chosen.	7/119; 8/126
Setting and location	5	State relevant aspects of the system(s) in which the decision(s) need(s) to be made.	7/119; 8/126
Study perspective	6	Describe the perspective of the study and relate this to the costs being evaluated.	8/137
Comparators	7	Describe the interventions or strategies being compared and state why they were chosen.	10/170-190
Time horizon	8	State the time horizon(s) over which costs and consequences are being evaluated and say why appropriate.	8/129
Discount rate	9	Report the choice of discount rate(s) used for costs and outcomes and say why appropriate.	8/139
Choice of health outcomes	10	Describe what outcomes were used as the measure(s) of benefit in the evaluation and their relevance for the type of analysis performed.	8/129
Measurement of effectiveness	11a	*Single study-based estimates*: Describe fully the design features of the single effectiveness study and why the single study was a sufficient source of clinical effectiveness data	24/527
	11b	*Synthesis-based estimates*: Describe fully the methods used for identification of included studies and synthesis of clinical effectiveness data.	24/527
Measurement and valuation of preference based	12	If applicable, describe the population and methods used to elicit preferences for outcomes	NA

For peer review only - http://bmjopen.bmj.com/site/about/guidelines.xhtml

CHEERS Checklist

outcomes			

Section/topic	#	Checklist item	Reported on page #
Estimating resources and costs	13a	*Single study-based economic evaluation*: Describe approaches used to estimate resource use associated with the alternative interventions. Describe primary or secondary research methods for valuing each resource item in terms of its unit cost. Describe any adjustments made to approximate to opportunity costs.	NA
	13b	*Model-based economic evaluation*: Describe approaches and data sources used to estimate resource use associated with model health states. Describe primary or secondary research methods for valuing each resource item in terms of its unit cost. Describe any adjustments made to approximate to opportunity costs.	Appendix, p.2-4/ 58-155
Currency, price date, and conversion	14	Report the dates of the estimated resource quantities and unit costs. Describe methods for adjusting estimated unit costs to the year of reported costs if necessary. Describe methods for converting costs into a common currency base and the exchange rate.	Appendix, p.2/ 63-68
Choice of model	15	Describe and give reasons for the specific type of decision-analytical model used. Providing a figure to show model structure is strongly recommended.	8/143-167
Assumptions	16	Describe all structural or other assumptions underpinning the decision-analytical model.	8/143;<br>Appendix p. 6/172
Analytical models	17	Describe all analytical methods supporting the evaluation. This could include methods for dealing with skewed, missing, or censored data; extrapolation methods; methods for pooling data; approaches to validate or make adjustments (such as half cycle corrections) to a model; and methods for handling population heterogeneity and uncertainty.	9/154-167 Appendix p. 1-4
**RESULTS**			
Study parameters	18	Report the values, ranges, references, and, if used, probability distributions for all parameters/ Report reasons or sources for distributions used to represent uncertainty where appropriate. Providing a table to show the input values is strongly recommended.	24/527-549
Incremental costs and outcomes	19	For each intervention, report mean values for the main categories of estimated costs and outcomes of interest, as well as mean differences between the comparator groups. If applicable, report incremental cost-effectiveness ratios.	27/569
Characterising uncertainty	20a	*Single study-based economic evaluation*: Describe the effects of sampling uncertainty for the estimated incremental cost and incremental effectiveness parameters, together with the impact of methodological assumptions (such as discount rate, study perspective).	NA
	20b	*Model-based economic evaluation*: Describe the effects on the results of uncertainty for all input parameters, and uncertainty related to the structure of the model and assumptions.	13-14/ 257-299

For peer review only

CHEERS Checklist

Section/topic	#	Checklist item	Reported on page #
Characterising heterogeneity	21	If applicable, report differences in costs, outcomes, or cost-effectiveness that can be explained by variations between subgroups of patients with different baseline characteristics or other observed variability in effects that are not reducible by more information.	NA
**Section/topic**	**#**	**Checklist item**	**Reported on page #**
**DISCUSSION**			
Study findings, limiations, generalisability, and current knowledge	22	Summarise key study findings and describe how they support the conclusions reached. Discuss limitations and the generalisability of the findings and how the findings fit with current knowledge.	16-20/ 312-407
**Other**			
Source of funding	23	Describe how the study was funded and the role of the funder in the identification, design, conduct, and reporting of the analysis. Describe other non-monetary sources of support.	4/ 57-62
Conflicts of interest	24	Describe any potential for conflict of interest of study contributors in accordance with journal policy. Int eh absence of a journal policy, we recommend authors comply with International Committee of Medical Journal Editors recommendations.	4/ 63-67

For peer review only
